# C_sp2_–H Amination Reactions Mediated by Metastable Pseudo-*O*_*h*_ Masked Aryl-Co^III^-nitrene Species

**DOI:** 10.1021/acs.inorgchem.2c02111

**Published:** 2022-08-23

**Authors:** Lorena Capdevila, Marc Montilla, Oriol Planas, Artur Brotons, Pedro Salvador, Vlad Martin-Diaconescu, Teodor Parella, Josep M. Luis, Xavi Ribas

**Affiliations:** † Institut de Química Computacional i Catàlisi (IQCC) and Departament de Química, Universitat de Girona, Campus Montilivi, Girona, E-17003, Catalonia, Spain; ‡ ALBA Synchrotron, Cerdanyola del Vallès, E-08290, Catalonia, Spain; § Servei de RMN, Facultat de Ciències, Universitat Autònoma de Barcelona, Campus UAB, Bellaterra, E-08193 Catalonia, Spain

## Abstract

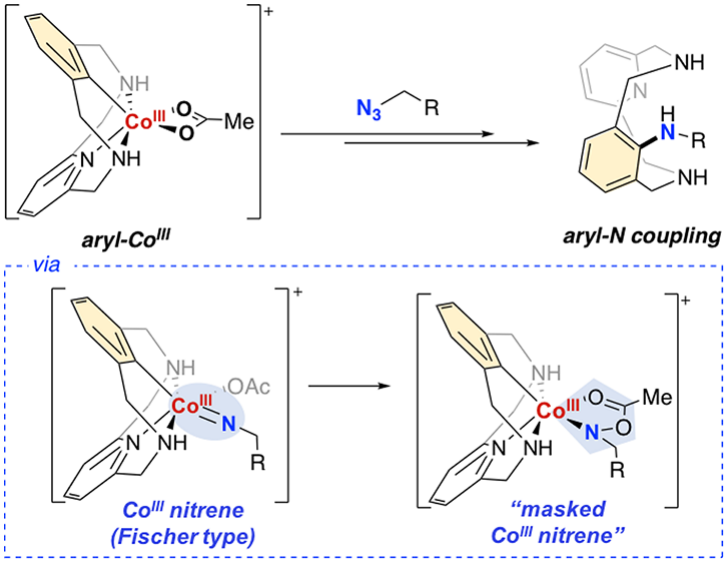

Cobalt-catalyzed C–H amination via M-nitrenoid species is spiking the interest of the research community. Understanding this process at a molecular level is a challenging task, and here we report a well-defined macrocyclic system featuring a pseudo-*O*_*h*_ aryl-Co^III^ species that reacts with aliphatic azides to effect intramolecular C_sp2_–N bond formation. Strikingly, a putative aryl-Co=NR nitrenoid intermediate species is formed and is rapidly trapped by a carboxylate ligand to form a carboxylate masked-nitrene, which functions as a shortcut to stabilize and guide the reaction to productive intramolecular C_sp2_–N bond formation. On one hand, several intermediate species featuring the C_sp2_–N bond formed have been isolated and structurally characterized, and the essential role of the carboxylate ligand has been proven. Complementarily, a thorough density functional theory study of the C_sp2_–N bond formation mechanism explains at the molecular level the key role of the carboxylate-masked nitrene species, which is essential to tame the metastability of the putative aryl-Co^III^=NR nitrene species to effectively yield the C_sp2_–N products. The solid molecular mechanistic scheme determined for the C_sp2_–N bond forming reaction is fully supported by both experimental and computation complementary studies.

## Abstract 2

A well-defined pseudo-*O*_*h*_ aryl-Co^III^ species reacts with aliphatic azides to effect intramolecular C_sp2_−N bond formation via a carboxylate masked-Co^III^-nitrene, which serves as a shortcut to guide the reaction to productive C_sp2_−N bond formation.

## Introduction

The introduction of nitrogen functionalities into organic frameworks has attracted considerable interest in the development of new methodologies, given their ubiquitous occurrence in pharmaceuticals and natural products.^1^ A powerful strategy to achieve the construction of C–N bonds is based on the direct functionalization of C–H bonds, which has been widely studied in the last few decades.^2−8^ This field has been mainly dominated by the use of noble metal catalysis; yet, the development of more sustainable methodologies using M-nitrenoid species with first-row transition metals has recently become a hot topic.^9^

**Figure 1:**
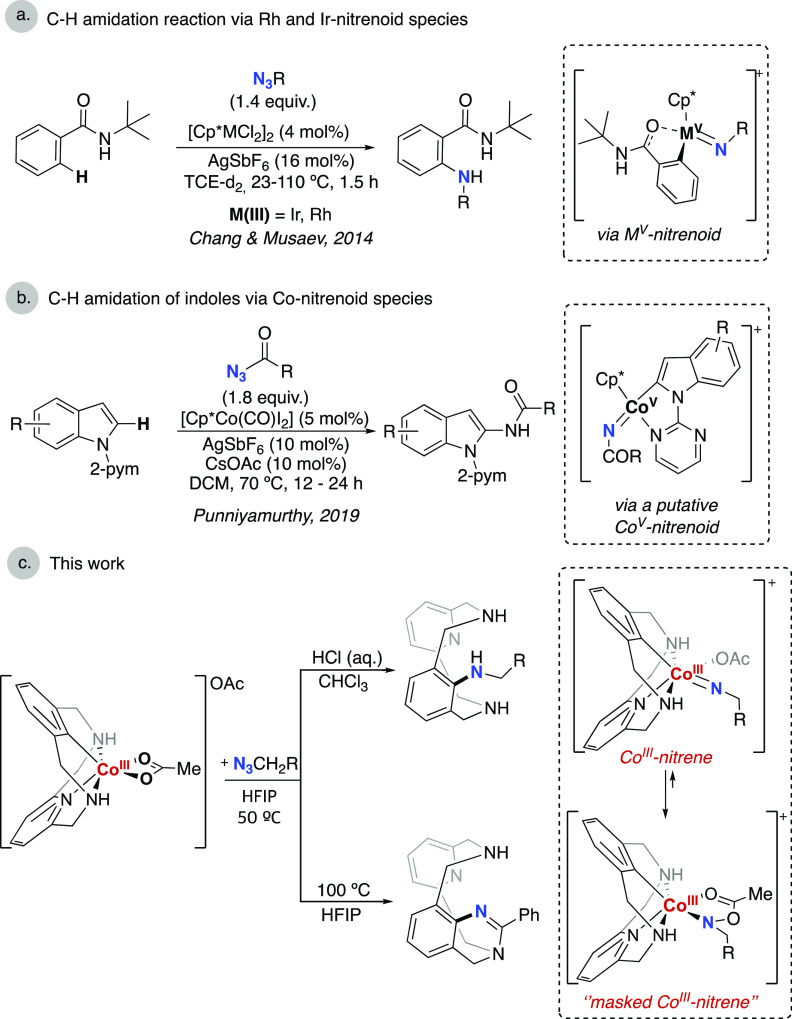
(a, b) Cp*-M-nitrenoid species proposed for the C–H amination reactions (M = Ir, Rh, Co); (c) this work.

M-Nitrenoid species are rare and unstable species for late transition metals. For Group 8 M-nitrenoids, a prominent example is the relatively stable octahedral iron(IV) terminal imido complex [Fe^IV^(N4Py)(NTs)]^2+^ reported by Que and co-workers,^10^ with *S* = 1 and a half-life of 3 h at room temperature.^11,12^ For transition metals in Group 9 and beyond, the common instability of *O*_*h*_ M-nitrenoid species may be overcome by changing the spin state or the geometry of the complex. In particular, the isolation of Group 9 Co-nitrene species has been achieved by lowering the symmetry and coordination number of the complex,^13−15^ highlighting four-coordinated complexes featuring tetrahedral geometry.^16,17^ Among all aminating reagents used to forge C–N bonds with cobalt catalysis, organic azides constitute an attractive N-source due to its 2e-oxidant character with concomitant extrusion of inert N_2_.^9,18,19^ The latter, together with the low-symmetry requirement, forces the design of low oxidation state Co^I^ species that form isolable Co^III^-imido multiple-bonded species upon reaction with N_3_-R.^16,17^ Although this chemistry is dominated by the use of low valent cobalt systems, few examples are reported on direct C_sp2_–N bond formation through C–H activation involving putative high-valent Co platforms.^20,21^ Indeed, highly unstable octahedral high valent M^V^=NR species with Group 9 metals are proposed as key intermediate species in C_sp2_–N bond forming processes. Remarkably, their relevance has been clearly pointed out formally in *O*_*h*_ M^V^ Group 9 complexes bearing a Cp* ligand.^9,22^ Reaction of cyclometalated Cp*Rh^III^ and Cp*Ir^III^ complexes with N_3_-R render the proposed Cp*M^V^=NR intermediate species,^23,24^ which are essential for the inner-sphere C_sp2_–N bond forming step with the cyclometalated ligand (Figure 1a). Regarding the analogous cobalt chemistry bearing a Cp* ligand, Matsunaga and Kanai demonstrated the ability of Cp*Co^III^ catalysts to perform the C_sp2_–H amidation of indoles using sulfonyl azides and phosphoryl azides.^25,26^ The C_sp2_–H amidation of indoles using acyl azides has also been reported using Cp*Co^III^ by Punniyamurthy and co-workers (Figure 1b)^27^ and using phenyl azidoformates by Chang and co-workers.^28^ Also, isoquinolone synthesis via Cp*Co^V^ cobaltacycles using N-chloroamides was reported by Zhu and co-workers.^29^ Despite these incipient reports, the high valent approach in Co-catalyzed C_sp2_–H amination is still in its infancy.

The examination of structure and electronic properties of key intermediate species is foremost for unveiling the mechanistic intricacies of inner-sphere N atom transfer chemistry. To this end, our group has been interested in the elucidation of transient intermediates involved in several C_sp2_–H functionalization reactions. In 2016, we reported a set of aryl-Co^III^ complexes synthesized through C_sp2_–H activation which were catalytically competent in alkyne and diazoacetate annulation reactions.^30−34^ Thanks to the stability offered by the 12-membered macrocyclic model substrate employed, we were able to isolate an unprecedented C-metalated *cis*-aryl-Co^III^-alkyl enolate complex, i.e., a masked-carbene species, which was demonstrated to be an on-cycle intermediate in the catalytic formation of the final C_sp2_–C products.^30,31^ Because of the extra stabilization offered by these model platforms, we hypothesized that they could offer a suitable electronic and geometric environment for studying the reactivity of pseudo-*O*_*h*_ aryl-Co^III^ organometallic complexes toward organic azides.

Herein, we report the N atom transfer reactivity of organic azides with well-defined aryl-Co^III^ complexes (Figure 1c), focusing on the step-by-step reactivity of intermediate species to unravel key mechanistic details of the C_sp2_–N bond formation. Aliphatic azides were found to efficiently effect the C_sp2_–N bond products. With a combination of experimental and density functional theory (DFT) studies, the full reconstruction of the N atom transfer process was revealed. Several intermediate species featuring the C_sp2_–N bond formed have been isolated and structurally characterized. The essential role of carboxylate-masked nitrenoid species to tame the metastability of the putative Co-nitrenoid was confirmed both experimentally and theoretically, affording a solid mechanistic picture of the C_sp2_-N bond forming process. The Co-nitrenoid is clearly described as an pseudo-*O*_*h*_ aryl-Co^III^-nitrene based on molecular orbital and electron density analyses, in contrast to the previously reported *O*_*h*_ Cp*Co^V^=NR imido species (Figure 1a,b).^25,26^

## Results and Discussion

The reactivity of the well-defined aryl-Co^III^ complex (**1-OAc**) with organic azides as nitrene precursors started by examining its reaction with *p*-NO_2_-phenyl azide. Unfortunately, the use of aromatic azides led to decomposition and formation of unidentified products. On the other hand, positive results were obtained with aliphatic azides. We started with the addition of benzyl azide (**a**) to **1-OAc** complex using fluorinated alcohols as solvent (TFE or HFIP) at 50 °C, affording the aryl-amine coupling complex **2a-OAc** in 46% yield (Scheme 2). This complex was structurally characterized by 2D NMR studies, where a diagnostic HMBC peak between the benzylic −CH_2_ of the formal azide and the quaternary carbon of the aryl moiety was observed, proving the formation of a new C_sp2_-NH bond. The coordinatively saturated complex **2a-OAc** slowly evolved at room temperature to a more stable dinuclear species, **3a-OAc**, in quantitative yield (see ^1^H NMR time-evolution in Figure S1). Crystals of **3a-OAc** were obtained from slow evaporation from a CH_2_Cl_2_ solution (DCM/pentane) at −4 °C, allowing for an unambiguous characterization of this dimeric species. Compound **3a-OAc** features the new C_sp2_–NH bond, and each Co^III^ center presents a distorted octahedral geometry, with coordination to N_py_, NH_L_, and NH_azide_ as well as one OAc and two μ-hydroxo bridging ligands. Independent blank experiments exposing **2a-OAc** crude mixture to H_2_O or O_2_ clearly suggested that the origin of the hydroxo groups in **3a-OAc** is O_2_.

**Scheme 1:**
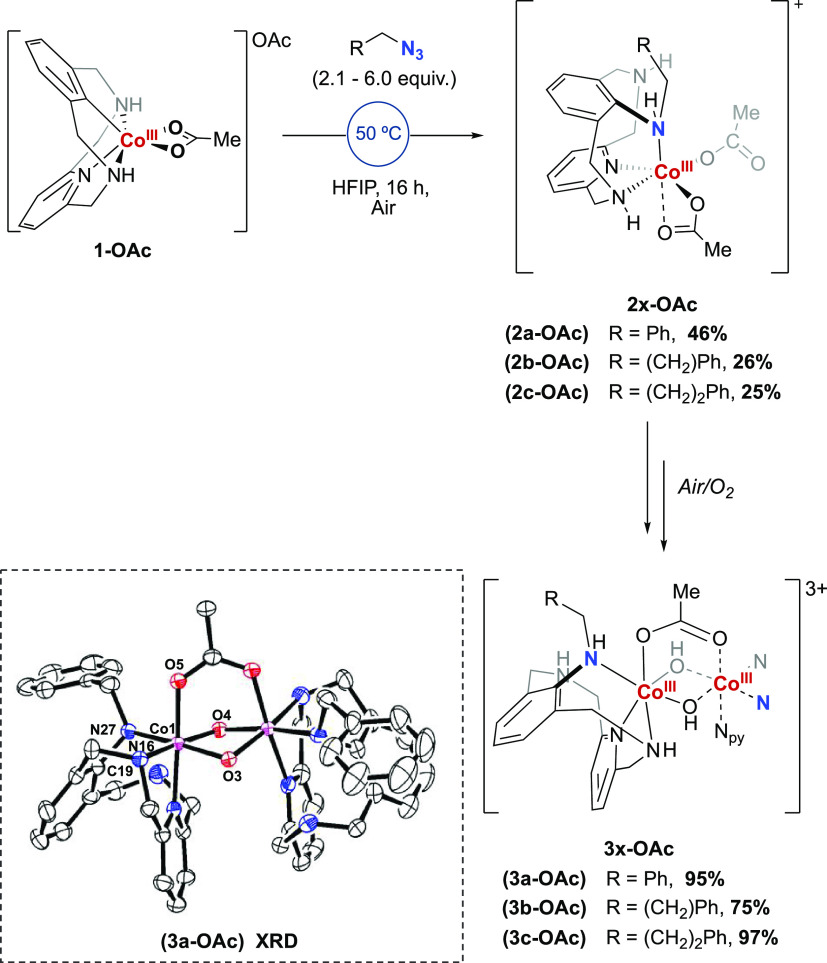
Reactivity of the Aryl-Co^III^ (**1-OAc**) with Organic Azides to Afford Complexes **2x-OAc** and **3x-OAc** NMR yields of **3x-OAc** are based on **2x-OAc**. Selected bond distances for [Å] and angles [deg]: C(19)–N(27) 1.441(16), N(27)–Co(1) 2.009(11), C(19)–N(27)–Co(1) 110.3(9), Co(1)–O(5) 1.924(9), Co(1)–N(16) 1.955(12), Co(1)–O(3) 1.932(9), Co(1)–O(4) 1.930 (9). Hydrogen atoms, anions, and solvents molecules have been omitted for clarity.

**Scheme 2:**
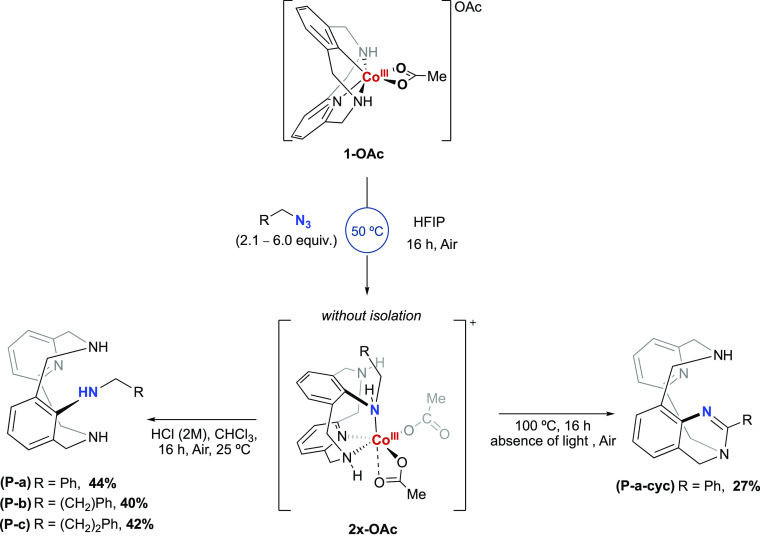
Thermal Decomposition and Acidic Work-up Affording the Corresponding Aminated Product **P-a**, **P-b**, **P-c**, and **P-a-cyc** Isolated yields shown.

Encouraged by these results, we explored the reactivity of **1-OAc** with (2-azidoethyl)benzene (**b**) and (3-azidopropyl)benzene (**c**) (Scheme 1). Using an excess of the azide **b** and **c** (6 equiv), the corresponding inserted complex (**2b-OAc** and **2c-OAc**) was obtained in 26% and 25% yield, respectively. Both complexes led to the quantitative formation of the corresponding dimer **3b-OAc** and **3c-OAc**, analogous to complex **3a-OAc** (vide supra).

The better yields observed for **2a-OAc** after treatment of **1-OAc** with benzyl azide prompted us to scrutinize the demetalation step. On the basis of previous reports,^24^ the protodemetalation step to render the aminated product was predicted to be kinetically and thermodynamically disfavored. Thus, to favor this step, we designed alternative strategies based on the use of strong acids and thermolysis (Scheme 2). First, HCl (2 M) was added to a solution of **2a-OAc** in CHCl_3_, and after 16 h the crude mixture was basified and extracted, affording the aminated product **P-a** in 44% isolated yield. The analogous reaction using **2b-OAc** and **2c-OAc** afforded the corresponding aminated product **P-b** and **P-c** in 40% and 42% respectively. On the other hand, heating **2a-OAc** to 100 °C in HFIP furnished the cyclized product **P-a-cyc** in 27% yield. The analogous cyclic products using **2b-OAc** and **2c-OAc** were not formed under the same conditions, which highlights the importance of the benzylic position for the formation of cyclized product (see mechanistic proposal for **P-a-cyc** formation in Scheme S7).

**Scheme 3:**
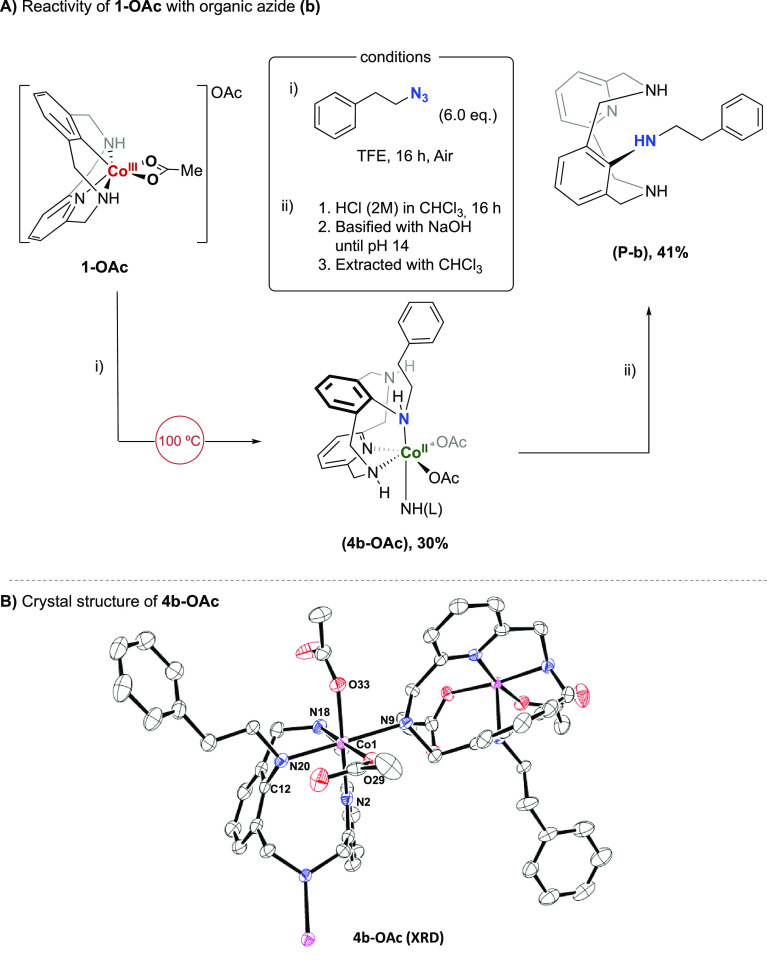
(A) Reactivity of Aryl-Co^III^ (**1-OAc**) with Organic Azide (**b**) at 100 °C and (B) Crystal Structure of **4b-OAc** Complex NMR yield of **P-b** is based on **4b-OAc**. Selected bond distances for [Å] and angles [deg]: C(12)–N(20) 1.439(5), N(20)–Co(1) 2.176(4), C(12)–N(20)–Co(1) 109.7(2), Co(1)–N(18) 2.127(4), Co(1)–N(9) 2.259(4), Co(1)–N(2) 2.255(4), Co(1)–O(33) 2.075(3), Co(1)–O(29) 2.056(3). Hydrogen atoms and solvent molecules have been omitted for clarity. NH(L) refers to the coordination of the Co(II) center to another ligand moiety (depicted as N9 in the crystal structure).

The absence of an analogous cyclic product from **2b-OAc** and **2c-OAc** led us to investigate in depth the reactivity of these azides under different thermal conditions (Scheme 3). When **1-OAc** was mixed with an excess of azide **b** at 100 °C in TFE, a new paramagnetic species appeared and was stable under inert atmosphere. XRD analysis showed a Co^II^ complex with distorted octahedral geometry bearing the phenylethan-1-amine moiety inserted (**4b-OAc**), which under acid conditions forms the product **P-b** in 41% NMR yield with respect to the **4b-OAc** complex.

Moreover, X-ray absorption spectroscopy (XAS) was conducted for **4b-OAc**, clearly confirming the Co^II^ oxidation state for the metal center (Table S2, Figure S22, Panels S1–S3) compared to Co^III^ species **1-OAc** and the newly synthesized aryl-Co^III^-benzylamine complex (**5-OAc**, see Scheme S13). The Co-ligand bond distances in the crystal structure of **4b-OAc** (>2.1 Å) suggest a high spin Co^II^–d^7^ electronic configuration, which was supported by the μ_eff_ calculated using Evans method in CD_2_Cl_2_. The obtained value of μ_eff_ = 4.22 MB is in agreement with the presence of three unpaired electrons. We hypothesized that the Co^II^ complex **4b-OAc** stemmed from reductive elimination of an in situ aryl-Co^IV^-imido, although more investigations are needed to shed some light on the detailed mechanism of the formation of **4b-OAc**.

The nature of the carboxylate ligand was also investigated, and we prepared the analogous **1-(OOCR)** complex bearing a substituted benzoate instead of the initial acetate (see Figures S23–S24 for the XRD of **1-(OBz-CF**_**3**_**)** and **1-(OBz-OMe)**). The use of EWG and EDG substituents did not affect the formation of the inserted **2a-OBz-X** complex (Scheme S9). Subjecting the mixture to acidic conditions led the formation of **P-a** product in similar yields. Moreover, the direct formation of **P-a-cyc** product was achieved by reacting several **1-(OBz-X)** with benzyl azide (**a**) under thermal conditions. The most coordinating *p*-OMe-benzoate affords a 42% yield, whereas the least coordinating *p*-NO_2_-benzoate affords only 16% yield, thus following the expected trend (see Scheme S11). However, the coordinating *p*-Me-benzoate drops to 17%, and the *p*-Cl-benzoate affords 49%. Therefore, the use of EWG and EDG substituents did not affect the formation of either complex **2a-OBz-X** complex or the final organic product **P-a-cyc**.

### Mechanistic Investigations

**Figure 2:**
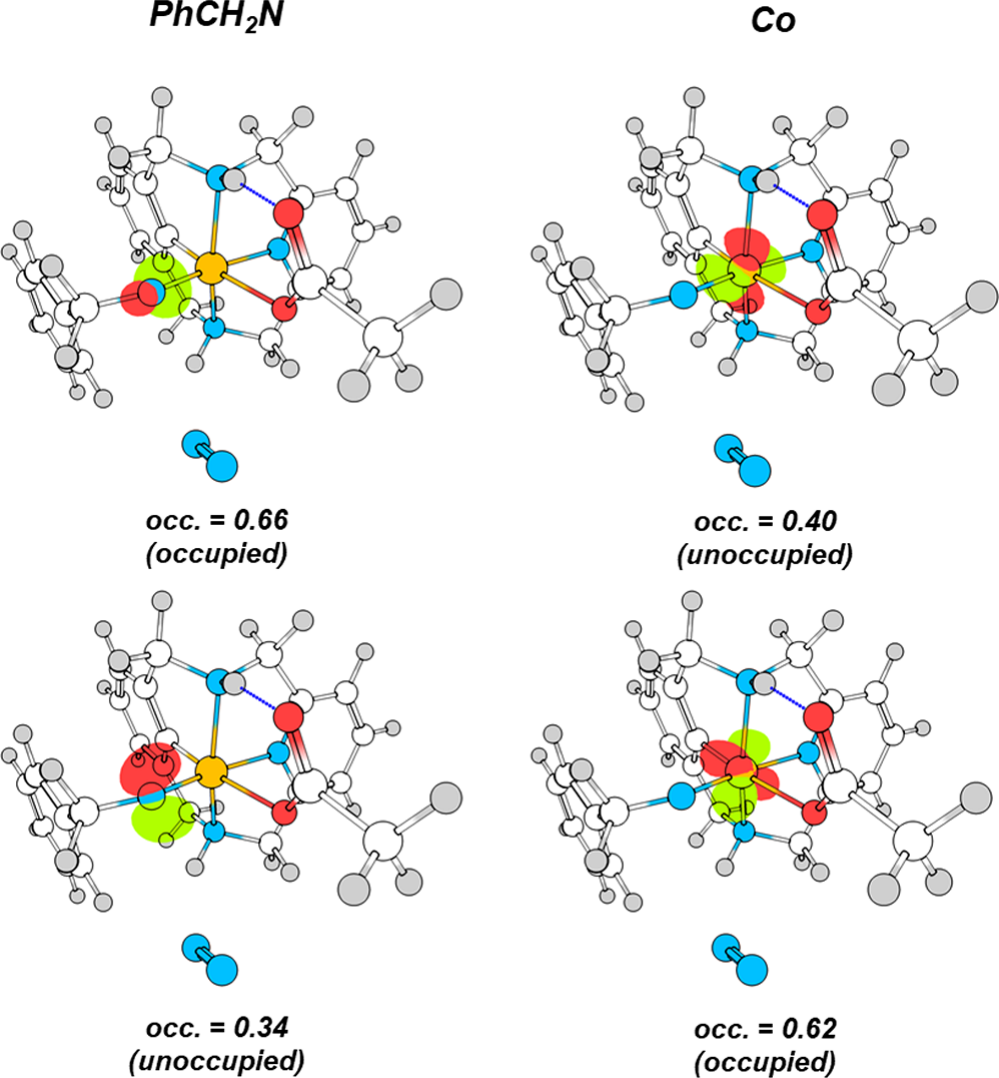
Effective fragment orbitals (EFOs) graphical representation and occupations—in the [0,1] range—associated with the σ (top) and π (bottom) interaction between the PhCH_2_N ligand and the Co center.

**Figure 3:**
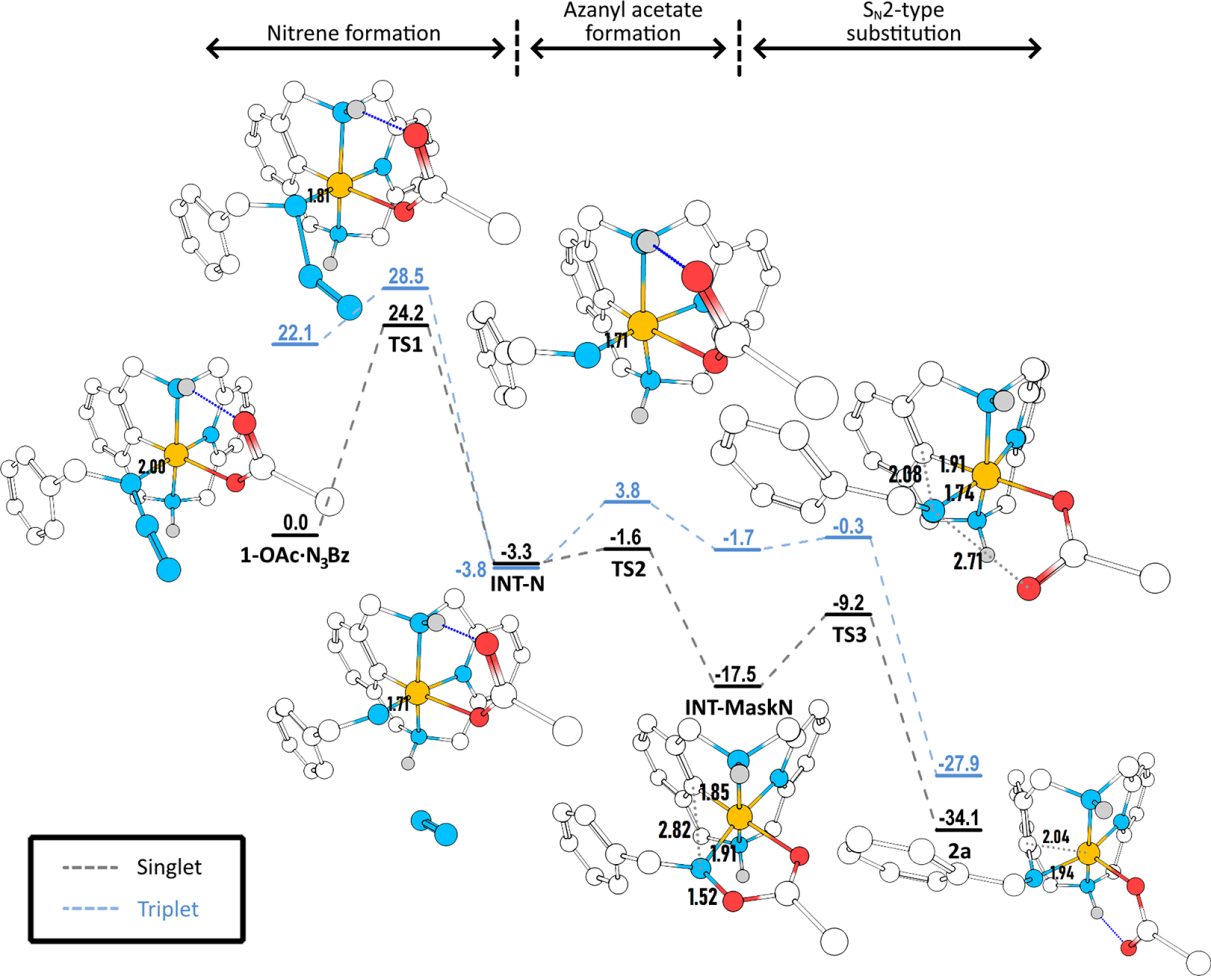
RevTPSS-D3BJ/Def2TZVP//BP86-D3BJ/Def2SVP free energy profile for the studied reaction mechanism. Gibbs free energies (*G*, in kcal·mol^–1^) are relative to **1-OAc·N**_**3**_**Bz**. The pathway in black corresponds to the singlet species (*S* = 0), while the blue pathway corresponds to the triplet species (*S* = 1). Geometries for all *S* = 0 intermediates and transition states are shown (nitrogen atoms are represented in blue, oxygens in red, cobalt in orange, carbon in white, and hydrogens in gray. Note that hydrogens bonded to carbon have been hidden for clarity). Relevant distances have also been included (in Å).

To gain more mechanistic insights of the C_sp2_–N bond formation, additional tests were performed. By adding TEMPO radical at 50 °C, MS peaks matching with a Co^II^ complex bearing the formed C_sp2_–N bond were detected, whereas at 100 °C the yield of **P-a-cyc** dropped from 27% to 7%. These results are not conclusive for either a radical or nonradical pathway since C_sp2_–N coupling is occurring, although in lower yields. Therefore, on the basis of all experimental evidence, a thorough computational DFT study was mandatory to unravel the precise mechanism for the intriguing C_sp2_–N bond forming step using benzyl azide and **1-OAc** (Figure 2 and Figure 3). The calculations were performed at the revTPSS-D3BJ/Def2TZVP//BP86-D3BJ/Def2SVP level of theory (see Supporting Information for full computational details and benchmark study). The rate-determining step of the reaction corresponds to the N_2_ extrusion from **1-OAc·N**_**3**_**Bz** to yield the short-lived species **INT-N**. Wave function analysis of **INT-N** indicated without a doubt that **INT-N** is best described as an aryl-Co^III^=N-R (R = −CH_2_Ph) nitrene species (Fischer-type) with σ and π bonds between the Co and the N atoms (bond order of 1.51 and bond length of 1.71 Å). Effective oxidation state (EOS) analysis in **INT-N** dissects the N–Co σ and π bonds into two contributions from the ligand and the metal, as shown in Figure 2 (see also Supporting Information). Considering the corresponding occupations of the effective fragment orbitals (EFOs), the EOS analysis assigns the two electrons of the N–Co σ bond to the nitrene, whereas the two electrons of the N–Co π bond are assigned to the Co. Therefore, **INT-N** may be described as an aryl Co^III^-nitrene with significant back-donation from Co to N. Qualitative analysis of the relative contributions of N and Co to the π and π* canonic molecular orbitals also characterize **INT-N** as a aryl Co^III^-nitrene (Figure S33 and Table S10), ruling out an aryl-Co^V^-imido species (Schrock-type).^35^

The EOS analysis of the *S* = 1 spin state of **INT-N** indicates that the triplet **INT-N** can also be described as a Co^III^-nitrene with a Co–N bond length of 1.73 Å. As it can be seen by the occupation and shape of the effective fragment orbitals (EFOs) depicted in Figure S36, in the singlet–triplet transition, the *S* = 0 beta electron of the lone-pair of the N is transferred to a p-type EFO of the N, resulting in a triplet state with two alpha p-type nonbonding electrons on the N. In addition, the remaining two beta electrons form two Co–N one-electron π bonds polarized toward the Co (see Figure S37). This analysis agrees with the fact that the major contribution of the spin density (i.e., electron density of alpha electrons minus the electron density of the beta electrons, which indicates the localization of the unpaired electrons) of the *S* = 1 state of **INT-N** is localized in the N (see Figure S34 and Table S12) and that the singlet → triplet spin-crossing does not cause significant change in the Co–N bond distance or in the formal oxidation state of the Co.

This intermediate species rapidly evolves overcoming a very low barrier (<2 kcal/mol, **TS2**) to a 14 kcal/mol more stable **INT-MaskN** species by formation of a five-member acetoxy(benzyl)amide ring via carboxylate attack to the N atom, formally defined as a masked aryl-Co-nitrene. Wave function analysis describes **INT-MaskN** as a masked Co^III^ nitrene with a single σ bond between the Co and the N. The lack of a Co=N π bond is also evidenced by the increased bond distance of 1.91 Å (1.71 Å for **INT-N**) and a decreased bond order of 0.78.

The masked aryl-Co^III^-nitrene (or “nitrenoid”)^36^ is not sufficiently stabilized to be experimentally trapped since it allows the formal nucleophilic attack of the aryl moiety to the N atom of the masked nitrene to finally achieve the C_sp2_–N coupling through a barrier lower than 9 kcal/mol, **TS3**. The partial atomic charge of the N atom in **INT-MaskN** (−0.51) vs **INT-N** (−0.74) explains the enhanced electrophilic character of the former, induced by the formation of the acetoxy(benzyl)amide. Moreover, EOS analysis also reveals a larger occupation of the aryl sigma contribution in **INT-MaskN** compared to **INT-N**, which favors the S_N_2 attack, and is an indication of the enhanced nucleophilic character of the aryl in **INT-MaskN** (see Figure S32).

The reaction profile has also been evaluated for *S* = 2 and *S* = 1 spin states. The energies of the *S* = 2 state of all intermediates and transition states involved in the reaction mechanism are far higher than the singlet, and therefore the quintuplet states play no role in the studied reaction mechanism (see Table S11). The triplet states does not play a key role either. The *S* = 1 state of the initial complex interacting with the benzyl azide is 22.1 kcal/mol above the singlet (Figure 3). The Gibbs energy of *S* = 1 states for **TS1**, **TS2**, **INT-MaskN**, **TS3**, and **2a** are also clearly higher than their *S* = 0 counterparts. The only exception is Co-nitrene intermediate **INT-N**, for which the triplet state is only 0.5 kcal/mol more stable than the singlet. However, because of the important electron reorganization that takes places on the N in the singlet–triplet transition, the probability of a spin-crossing between the singlet and the triplet Gibbs energy surfaces is strongly reduced. Thus, all of the computational evidence indicates that the reaction profile undergoes a singlet species.

We have performed several DFT relaxed PES scans to explore the stability of aryl-Co^III^-nitrene complex **INT-N** upon distortion or disconnection of one of the coordinating N.^17^ However, all of the calculations confirm that the tight coordination environment imposed by the macrocyclic ligand in the aryl-Co^III^-nitrene complex **INT-N** is mandatory for its stabilization. In addition, we have tried to locate the transition state that corresponds to the direct formation of the C–N bond (species **2a**) from intermediate **INT-N**. However, all of our attempts lead to transition state **TS2** or intermediate **INT-MaskN**.

The role of carboxylate anions was experimentally confirmed by using the acetate-free organometallic [aryl-Co^III^-(CH_3_CN)_2_]^2+^ complex (**1-CH**_**3**_**CN**) (Scheme S14). Applying reaction conditions at 50 and 100 °C using benzyl azide (**a**), neither the aryl-amine coupling complex (analogous to **2a-OAc**) (50 °C) nor final organic product **P-a-cyc** (100 °C) was detected. Furthermore, the presence of benzylaldehyde as a side product suggested the degradation of benzyl azide, pointing out the importance of the formation of the masked-aryl-Co-nitrene species **INT-MaskN** toward the C_sp2_–N coupling.

Previously, some of us studied the mechanism of C_sp2_–H functionalization with diazo esters catalyzed with a aryl-Co^III^-carboxylate compound,^31^ in which the key role of a carboxylate-masked aryl-Co^III^-carbene was proven. Analogously, the formation of the aryl-Co-nitrene, the facile evolution of the nitrene through a low-lying transition state to form a five-member acetoxy(benzyl)amide ring, as well as the final S_N_2-type substitution of the aryl-Co to the masked nitrene are reminiscent of the mechanism of the cobalt-catalyzed C_sp2_-H functionalization with diazo esters. The key difference between both mechanisms is the stability of the masked carbene and the masked nitrene. Whereas the masked-carbene could be isolated and fully experimentally characterized because the final nucleophilic attack is the rate-determining step of the reaction, the analogous masked nitrene could not be isolated due to the barrier to form the coupling product being much smaller than the barrier for the formation of the aryl-Co^III^-nitrene. Experimentally, we conducted UV–vis monitoring analyses at variable temperature, which allows us to determine that the release of N_2_ toward the formation of the nitrene species **INT-N** is indeed the rate-determining step (rds) of the reaction (Δ*G*^‡^ = 23.9 kcal/mol, Eyring plot in Figure S20), which nicely agrees with the DFT Gibbs energy profile of the reaction mechanism presented in Figure 3. MS analysis after mixing time agrees with the accumulation of **1-OAc·N**_**3**_**Bz** species (Figures S19 and S20).

**Scheme 4:**
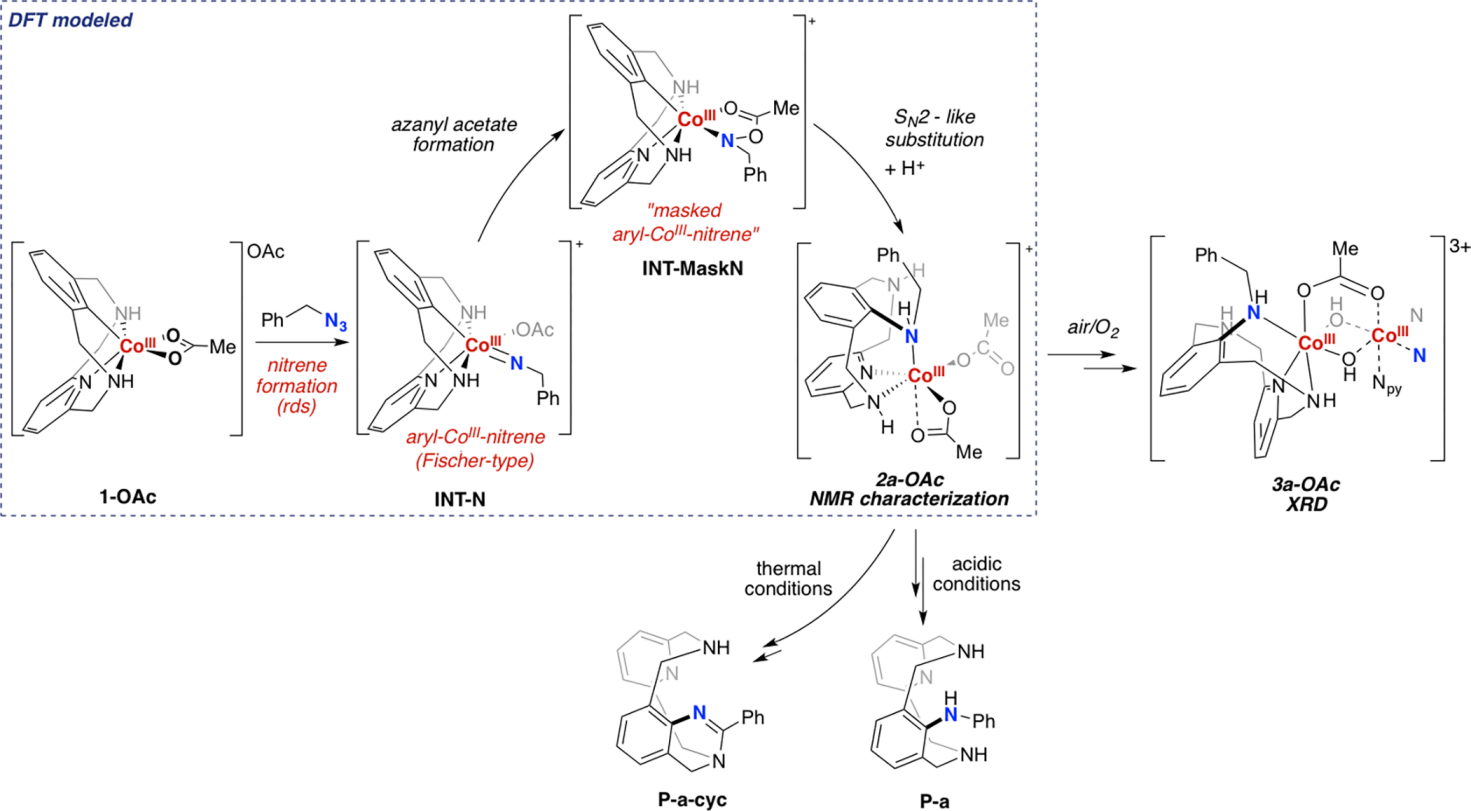
Proposed Mechanism for the Reaction of **1-OAc** with Benzylazide (**a**)

The proposed general mechanism is shown in Scheme 4. This study demonstrates the stabilizing masking effect of the carboxylate group to the Co-nitrene moiety, to tame the extraordinary reactivity and elusiveness of Co-nitrene species.

### Intermolecular Nitrene-Transfer Attempts

Additionally, nitrene transfer was attempted by adding xanthene (2 equiv) to the mixture of **1-OAc** and benzyl azide (**a**), but no intermolecular C_sp2_–N coupling product with xanthene was detected, and intramolecular **2a-OAc** (20%) was formed (see Scheme S15).

### Comparing the Reactivity of Organometallic Co^III^ and Rh^III^ Complexes

To gain more insight into the mechanism, we explored the reactivity of benzyl azide using an analogous aryl-Rh^III^ complex. However, attempts to isolate the aryl-Rh^III^ analogous to **1-OAc** using **L-H** were unsuccessful. Thus, we attempted the formation of **P-a-cyc** by reacting the **L-H** ligand with benzyl azide (**a**) and stoichiometric amounts of Rh(OAc)_3_ in TFE at 100 °C. In contrast to the aryl-Co^III^ complex, **P-a-cyc** cyclic product was not formed, and we only detected the formation of hydrazine. Therefore, since the aryl-Rh^III^ complex was not isolable with **L-H**, we attempted successfully the comparison of both aryl-Co^III^ (**1**_**Me**_**-OAc**) and aryl-Rh^III^ (**6**_**Me**_**-OAc**) synthesized with the **L-Me** ligand. Co^III^ complex **1**_**Me**_**-OAc** was mixed with benzylazide, but no reaction was observed. By reacting complex **6**_**Me**_**-OAc** with benzylazide, only aryl-Rh^III^-imine species (**7x**_**Me**_**-OAc**) was observed, with no trace of C_sp2_-N bond-formed species (Scheme S18). The contrasting reactivity of Co versus Rh analogues highlights the uniqueness of Co reactivity, its versatility to stabilize metastable species via carboxylate masking, and the value of studying in depth the role of first row metals in C–N formation.

## Conclusions

In summary, we have studied the reactivity of well-defined pseudo-*O*_*h*_ aryl-Co^III^ species (**1-OAc**) with different azides, with successful intramolecular C_sp2_–N bond formation with aliphatic azides. In brief, when benzyl azide is used, we are able to trap the just-formed C_sp2_–N bond species, consisting of a Co^III^ complex (**2a-OAc**) that tends to dimerize to form complex **3a-OAc**. Analogous reactivity is found for (2-azidoethyl)benzene (**b**) and (3-azidopropyl)benzene (**c**). However, only **2a-OAc** evolves to an intramolecular cyclization to obtain the organic product **P-a-cyc**, whereas **1-OAc** reacts with **b** under thermal treatment to afford a well-defined Co^II^ complex featuring the already formed C_sp2_–N bond (**4b-OAc**). The thorough DFT study performed demonstrates the stabilizing masking effect of the carboxylate group to tame the extraordinary reactivity and elusiveness of an aryl-Co^III^=N-R nitrene species (**INT-N**). First, the Gibbs energy barrier of the rate-determining step of the reaction, which corresponds to the N_2_ extrusion, is in agreement with the Gibbs energy barrier extracted from the Eyring plot and the mild experimental conditions applied (50 °C). More importantly, detailed wave function analysis of the masked aryl-Co^III^-nitrene species **INT-MaskN** clearly shows an increase of electrophilicity on N and an increase of nucleophilicity on the C_sp2_-aryl compared to **INT-N**, thus promoting the facile S_N_2-like attack to effect the C_sp2_–N coupling. This is in line with the fact that the S_N_2-like barrier is far lower than the barrier for the formation of the aryl-Co^III^-nitrene and with the fact that these masked species could not be trapped as in the case of the masked carbene.^31^ The key role of carboxylate anions in the formation of masked aryl-Co^III^-nitrene species, fully supported by both experimental and computation studies, culminated in a solid mechanistic picture of the C_sp2_–N bond forming amination process, which is thought to be valuable for the future development of catalytic C_sp2_–N methodologies via Co=NR species. Indeed, Co^III^ masked nitrene species have been crystallographically isolated very recently,^37^ further supporting the validity of our study. Moreover, the occurrence of other transition metal masked nitrene species (Ru) is also proposed in chiral α-amino acid synthesis using carbamate derivatives.^38^ Interestingly, this in situ masking strategy is a straightforward alternative to the use of stabilized nitrene sources such as dioxazolones (CO_2_-evolving reagent), which focus the scope on amidation reactions.^39−41^

## Experimental Section

### Formation of **2x-OAc** and **3x-OAc** Complexes

**1-OAc** (0.048 mmol) and organic azides (**a–c**) with 1 mL of HFIP were mixed in a 2 mL vial and sealed. The mixture was heated at 50 °C overnight. Then, the crude mixture was concentrated under a vacuum line until the initial volume was reduced to two-thirds observing the formation of **2x-OAc** intermediate complex by ^1^H NMR (CDCl_3_) and HRMS. The corresponding dimeric species **3x-OAc** were slowly formed by recrystallization with CHCl_3_ layered with pentane under air.

### Synthesis of **P-x** Products

Once the **2x-OAc** were formed, each crude mixture was dissolved in CHCl_3_, and HCl (3 mmol, 2 M) was added and stirred overnight. The crude was basified until pH 14 and extracted with CHCl_3_. The products were purified by column chromatography using neutral alumina (CHCl_3_, then CHCl_3_/MeOH 8:2), giving the corresponding C_sp2_–N coupling products (**P-a**, **P-b**, and **P-c**).

### Synthesis of **P-a-cyc** Product

**1-OAc** (0.048 mmol) and benzyl azide (**a**) (2.1 equiv) were mixed in HFIP (1 mL) in a 2 mL vial and sealed. The crude was heated at 100 °C overnight in the absence of light. The solvent was then removed, and the cyclic product was purified by column chromatography using neutral alumina (CHCl_3_, then CHCl_3_/MeOH. 8:2).

### Formation of **4b-OAc**

**1-OAc** (0.048 mmol) and (2-azidoethyl)benzene (**b**) (6.0 equiv) were mixed in TFE (1 mL) in a 2 mL vial and sealed. The crude was heated at 100 °C, and after 16 h the solvent was removed. Pentane diffusion in a concentrated solution of CH_2_Cl_2_ anhydrous under inert atmosphere yields the **4b-OAc** complex.

### Computational Details

All DFT calculations were carried out using Gaussian16 program. Geometry optimizations have been performed without any symmetry restrictions, considering the effect of the HFIP solvent via the Self-Consistent Reaction Field method using the SMD solvation model^42^ and taking into account dispersion effects with Grimme and co-workers DFT-D3BJ correction,^43,44^ at the BP86-D3BJ(SMD)/Def2SVP level of theory.^45−48^ The HFIP solvent is not implemented in GAUSSIAN16, so we performed those calculations using the *Solvent = Generic,Read* options for the *SCRF* keyword (see Supporting Information for further details). All geometry optimized structures were characterized by analytical frequency calculations, which also afforded enthalpy and entropy corrections at 298.15 K. All points in the reaction pathway were connected via IRC calculations. Single point calculations on the equilibrium geometries, including the solvent and dispersion effects (*E*_sp_), were carried out at the revTPSS-D3BJ(SMD)/Def2TZVP level of theory.^49^ Then, the total Gibbs energy values (*G*) are given by
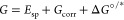
1where the Gibbs energy correction (*G*_corr._) was obtained from the thermodynamical analysis at the optimization level of theory but corrected using the GoodVibes code^50^ so that frequencies below 100 are not treated with the Harmonic Approximation, but rather with the Quasi-Harmonic Approximation as described by Grimme.^51^ Finally, the additional correction term Δ*G*^°/*^ accounts for the transition from the standard state concentration (gas phase, pressure of 1 atm) to the concentrations used experimentally.

Metal and ligands oxidation states (OS) were assigned with the effective oxidation states (EOS) analysis, which relies on Mayer’s effective fragment orbitals (EFOs) and their occupations. The EFOs are sorted by decreasing occupation number, and individual electrons (or pairs for closed-shell singlets) are assigned to those EFOs with higher occupations. This leads to an effective configuration of the atoms/ligands within the molecule, which directly determines their OS. EOS analysis was performed at the revTPSS-D3BJ/Def2TZVP level of theory with the in-house developed program APOST-3D,^52^ using the Topological Fuzzy Voronoi Cells (TFVC) atomic definition and a 40 × 146 atomic grid for the required numerical integrations.

## Supporting Information Available

Additional experimental details, materials, instrumentation, spectroscopic characterization of all compounds, computational details and X-ray crystallography details of **1-OBzCF**_**3**_ (CCDC 2097542), **1-OBzOMe** (CCDC 2097543), **3a-OAc** (CCDC 2097546), **4b-OAc** (CCDC 2097544), and **5-OAc** (CCDC 2097545) (PDF)

## References

[ref1] Hili R.; Yudin A. K. Making carbon-nitrogen bonds in biological and chemical synthesis. Nat. Chem. Biol. 2006, 2, 284–287. 10.1038/nchembio0606-284. 16710330

[ref2] Davies H. M. L.; Long M. S. Recent Advances in Catalytic Intramolecular C-H Aminations. Angew. Chem., Int. Ed. 2005, 44, 3518–3520. 10.1002/anie.200500554. 15887207

[ref3] Halfen J. A. Recent Advances in Metal-Mediated Carbon-Nitrogen Bond Formation Reactions: Aziridination and Amidation. Curr. Org. Chem. 2005, 9, 657–669. 10.2174/1385272053765024.

[ref4] Davies H. M. L.; Manning J. R. Catalytic C-H functionalization by metal carbenoid and nitrenoid insertion. Nature 2008, 451, 417–424. 10.1038/nature06485. 18216847 PMC3033428

[ref5] Collet F.; Dodd R. H.; Dauban P. Catalytic C-H amination: recent progress and future directions. Chem. Commun. 2009, 5061–5074. 10.1039/b905820f. 20448953

[ref6] Zalatan D. N.; Bois J. D. Metal-Catalyzed Oxidations of C-H to C-N Bonds. In C-H Activation; Yu J.-Q.; Shi Z., Eds.; Springer Berlin Heidelberg: Berlin, Heidelberg, 2010; pp 347–378. 10.1007/128_2009_19 21500412

[ref7] Müller P.; Fruit C. Enantioselective Catalytic Aziridinations and Asymmetric Nitrene Insertions into CH Bonds. Chem. Rev. 2003, 103, 2905–2920. 10.1021/cr020043t. 12914485

[ref8] Louillat M.-L.; Patureau F. W. Oxidative C-H amination reactions. Chem. Soc. Rev. 2014, 43, 901–910. 10.1039/C3CS60318K. 24217419

[ref9] Shin K.; Kim H.; Chang S. Transition-Metal-Catalyzed C-N Bond Forming Reactions Using Organic Azides as the Nitrogen Source: A Journey for the Mild and Versatile C-H Amination. Acc. Chem. Res. 2015, 48, 1040–1052. 10.1021/acs.accounts.5b00020. 25821998

[ref10] Klinker E. J.; Jackson T. A.; Jensen M. P.; Stubna A.; Juhász G.; Bominaar E. L.; Münck E.; Que Jr L. A Tosylimido Analogue of a Nonheme Oxoiron(IV) Complex. Angew. Chem., Int. Ed. 2006, 45, 7394–7397. 10.1002/anie.200602799. 17039556

[ref11] Kumar S.; Faponle A. S.; Barman P.; Vardhaman A. K.; Sastri C. V.; Kumar D.; de Visser S. P. Long-Range Electron Transfer Triggers Mechanistic Differences between Iron(IV)-Oxo and Iron(IV)-Imido Oxidants. J. Am. Chem. Soc. 2014, 136, 17102–17115. 10.1021/ja508403w. 25392052

[ref12] Coin G.; Patra R.; Rana S.; Biswas J. P.; Dubourdeaux P.; Clémancey M.; de Visser S. P.; Maiti D.; Maldivi P.; Latour J.-M. Fe-Catalyzed Aziridination Is Governed by the Electron Affinity of the Active Imido-Iron Species. ACS Catal. 2020, 10, 10010–10020. 10.1021/acscatal.0c01427.

[ref13] Zhang L.; Liu Y.; Deng L. Three-Coordinate Cobalt(IV) and Cobalt(V) Imido Complexes with N-Heterocyclic Carbene Ligation: Synthesis, Structure, and Their Distinct Reactivity in C-H Bond Amination. J. Am. Chem. Soc. 2014, 136, 15525–15528. 10.1021/ja509731z. 25330361

[ref14] Reckziegel A.; Pietzonka C.; Kraus F.; Werncke C. G. C-H Bond Activation by an Imido Cobalt(III) and the Resulting Amido Cobalt(II) Complex. Angew. Chem., Int. Ed. 2020, 59, 8527–8531. 10.1002/anie.201914718. PMC7318117 32119164

[ref15] Grünwald A.; Anjana S. S.; Munz D. Terminal Imido Complexes of the Groups 9–11: Electronic Structure and Developments in the Last Decade. Eur. J. Inorg. Chem. 2021, 2021, 4147–4166. 10.1002/ejic.202100410.

[ref16] Baek Y.; Das A.; Zheng S.-L.; Reibenspies J. H.; Powers D. C.; Betley T. A. C-H Amination Mediated by Cobalt Organoazide Adducts and the Corresponding Cobalt Nitrenoid Intermediates. J. Am. Chem. Soc. 2020, 142, 11232–11243. 10.1021/jacs.0c04252. 32456423 PMC9340662

[ref17] Mao W.; Fehn D.; Heinemann F. W.; Scheurer A.; Munz D.; Meyer K. A Pair of Cobalt(III/IV) Terminal Imido Complexes. Angew. Chem., Int. Ed. 2021, 60, 16480–16486. 10.1002/anie.202103170. PMC8362208 33847448

[ref18] Dequirez G.; Pons V.; Dauban P. Nitrene Chemistry in Organic Synthesis: Still in Its Infancy?. Angew. Chem., Int. Ed. 2012, 51, 7384–7395. 10.1002/anie.201201945. 22730346

[ref19] Park Y.; Semproni S. P.; Zhong H.; Chirik P. J. Synthesis, Electronic Structure, and Reactivity of a Planar Four-Coordinate, Cobalt-Imido Complex. Angew. Chem., Int. Ed. 2021, 60, 14376–14380. 10.1002/anie.202104320. 33876539

[ref20] Du C.; Li P.-X.; Zhu X.; Han J.-N.; Niu J.-L.; Song M.-P. Cobalt-Catalyzed Oxidative C-H/N-H Cross-Coupling: Selective and Facile Access to Triarylamines. ACS Catal. 2017, 7, 2810–2814. 10.1021/acscatal.7b00262.

[ref21] Jia Q.; Kong L.; Li X. Cobalt(iii)-catalyzed C-H amidation of weakly coordinating sulfoxonium ylides and α-benzoylketene dithioacetals. Org. Chem. Front. 2019, 6, 741–745. 10.1039/C8QO01270A.

[ref22] Sau Y.-K.; Yi X.-Y.; Chan K.-W.; Lai C.-S.; Williams I. D.; Leung W.-H. Insertion of nitrene and chalcogenolate groups into the Ir-C σ bond in a cyclometalated iridium(III) complex. J. Organomet. Chem. 2010, 695, 1399–1404. 10.1016/j.jorganchem.2010.02.002.

[ref23] Park S. H.; Kwak J.; Shin K.; Ryu J.; Park Y.; Chang S. Mechanistic Studies of the Rhodium-Catalyzed Direct C-H Amination Reaction Using Azides as the Nitrogen Source. J. Am. Chem. Soc. 2014, 136, 2492–2502. 10.1021/ja411072a. 24450395

[ref24] Figg T. M.; Park S.; Park J.; Chang S.; Musaev D. G. Comparative Investigations of Cp*-Based Group 9 Metal-Catalyzed Direct C-H Amination of Benzamides. Organometallics 2014, 33, 4076–4085. 10.1021/om5005868.

[ref25] Sun B.; Yoshino T.; Matsunaga S.; Kanai M. Air-Stable Carbonyl(pentamethylcyclopentadienyl)cobalt Diiodide Complex as a Precursor for Cationic (Pentamethylcyclopentadienyl)cobalt(III) Catalysis: Application for Directed C-2 Selective C-H Amidation of Indoles. Adv. Synth. Catal. 2014, 356, 1491–1495. 10.1002/adsc.201301110.

[ref26] Sun B.; Yoshino T.; Matsunaga S.; Kanai M. A Cp*CoI2-dimer as a precursor for cationic Co(iii)-catalysis: application to C-H phosphoramidation of indoles. Chem. Commun. 2015, 51, 4659–4661. 10.1039/C4CC10284C. 25690436

[ref27] Shah T. A.; De P. B.; Pradhan S.; Banerjee S.; Punniyamurthy T. Cp*Co(III)-Catalyzed Regioselective C2 Amidation of Indoles Using Acyl Azides. J. Org. Chem. 2019, 84, 16278–16285. 10.1021/acs.joc.9b02244. 31771331

[ref28] Lee J.; Lee J.; Jung H.; Kim D.; Park J.; Chang S. Versatile Cp*Co(III)(LX) Catalyst System for Selective Intramolecular C-H Amidation Reactions. J. Am. Chem. Soc. 2020, 142, 12324–12332. 10.1021/jacs.0c04448. 32551631

[ref29] Yu X.; Chen K.; Guo S.; Shi P.; Song C.; Zhu J. Direct Access to Cobaltacycles via C-H Activation: N-Chloroamide-Enabled Room-Temperature Synthesis of Heterocycles. Org. Lett. 2017, 19, 5348–5351. 10.1021/acs.orglett.7b02632. 28926268

[ref30] Planas O.; Roldán-Gómez S.; Martin-Diaconescu V.; Luis J. M.; Company A.; Ribas X. Mechanistic insights into the SN2-type reactivity of aryl-Co(iii) masked-carbenes for C-C bond forming transformations. Chem. Sci. 2018, 9, 5736–5746. 10.1039/C8SC00851E. 30079183 PMC6050605

[ref31] Planas O.; Roldán-Gómez S.; Martin-Diaconescu V.; Parella T.; Luis J. M.; Company A.; Ribas X. Carboxylate-Assisted Formation of Aryl-Co(III) Masked-Carbenes in Cobalt-Catalyzed C-H Functionalization with Diazo Esters. J. Am. Chem. Soc. 2017, 139, 14649–14655. 10.1021/jacs.7b07880. 28920682

[ref32] Planas O.; Whiteoak C. J.; Martin-Diaconescu V.; Gamba I.; Luis J. M.; Parella T.; Company A.; Ribas X. Isolation of Key Organometallic Aryl-Co(III) Intermediates in Cobalt-Catalyzed C(sp2)-H Functionalizations and New Insights into Alkyne Annulation Reaction Mechanisms. J. Am. Chem. Soc. 2016, 138, 14388–14397. 10.1021/jacs.6b08593. 27723326

[ref33] Planas O.; Whiteoak C. J.; Ribas X. Recent Advances in Cobalt-Catalyzed Cross-coupling Reactions. In Non-Noble Metal Catalysis; Gebbink R. J. M. K.; Moret M. E., Eds.; Wiley: 2019; pp 297–328.

[ref34] Planas O.; Chirila P. G.; Whiteoak C. J.; Ribas X. Chapter Four - Current Mechanistic Understanding of Cobalt-Catalyzed C-H Functionalization. In Adv. Organomet. Chem.; Pérez P. J., Ed.; Academic Press: 2018; Vol. 69, pp 209–282.

[ref35] Kuijpers P. F.; van der Vlugt J. I.; Schneider S.; de Bruin B. Nitrene Radical Intermediates in Catalytic Synthesis. Chem.—Eur. J. 2017, 23, 13819–13829. 10.1002/chem.201702537. 28675476 PMC5656926

[ref36] Caballero A.; Pérez P. J. Dimensioning the Term Carbenoid. Chem.—Eur. J. 2017, 23, 14389–14393. 10.1002/chem.201702392. 28640943

[ref37] Zhang H.; Sun M.-C.; Yang D.; Li T.; Song M.-P.; Niu J.-L. Cobalt(II)-Catalyzed Activation of C(sp3)-H Bonds: Organic Oxidant Enabled Selective Functionalization. ACS Catal. 2022, 12, 1650–1656. 10.1021/acscatal.1c05250.

[ref38] Ye C.-X.; Shen X.; Chen S.; Meggers E. Stereocontrolled 1,3-nitrogen migration to access chiral α-amino acids. Nat. Chem. 2022, 14, 566–573. 10.1038/s41557-022-00895-3. 35379900 PMC7612692

[ref39] Hong S. Y.; Hwang Y.; Lee M.; Chang S. Mechanism-Guided Development of Transition-Metal-Catalyzed C-N Bond-Forming Reactions Using Dioxazolones as the Versatile Amidating Source. Acc. Chem. Res. 2021, 54, 2683–2700. 10.1021/acs.accounts.1c00198. 33979133

[ref40] Lee S.; Rovis T. Rh(III)-Catalyzed Three-Component Syn-Carboamination of Alkenes Using Arylboronic Acids and Dioxazolones. ACS Catal. 2021, 11, 8585–8590. 10.1021/acscatal.1c02406. 34745710 PMC8570580

[ref41] Sunny S.; Karvembu R. Recent Advances in Cobalt-Catalyzed, Directing-Group-Assisted C-H Bond Amidation Reactions. Adv. Synth. Catal. 2021, 363, 4309–4331. 10.1002/adsc.202100558.

[ref42] Marenich A. V.; Cramer C. J.; Truhlar D. G. Universal solvation model based on solute electron density and on a continuum model of the solvent defined by the bulk dielectric constant and atomic surface tensions. J. Phys. Chem. B 2009, 113, 6378–6396. 10.1021/jp810292n. 19366259

[ref43] Grimme S.; Ehrlich S.; Goerigk L. Effect of the damping function in dispersion corrected density functional theory. J. Comput. Chem. 2011, 32, 1456–1465. 10.1002/jcc.21759. 21370243

[ref44] Grimme S.; Antony J.; Ehrlich S.; Krieg H. A consistent and accurate ab initio parametrization of density functional dispersion correction (DFT-D) for the 94 elements H-Pu. J. Chem. Phys. 2010, 132, 154104 10.1063/1.3382344. 20423165

[ref45] Becke A. D. Density-functional exchange-energy approximation with correct asymptotic behavior. Phys. Rev. A 1988, 38, 3098–3100. 10.1103/PhysRevA.38.3098. 9900728

[ref46] Perdew J. P. Density-functional approximation for the correlation energy of the inhomogeneous electron gas. Phys. Rev. B 1986, 33, 8822–8824. 10.1103/PhysRevB.33.8822. 9938299

[ref47] Schäfer A.; Huber C.; Ahlrichs R. Fully optimized contracted Gaussian basis sets of triple zeta valence quality for atoms Li to Kr. J. Chem. Phys. 1994, 100, 5829–5835. 10.1063/1.467146.

[ref48] Weigend F.; Ahlrichs R. Balanced basis sets of split valence, triple zeta valence and quadruple zeta valence quality for H to Rn: Design and assessment of accuracy. Phys. Chem. Chem. Phys. 2005, 7, 3297–3305. 10.1039/b508541a. 16240044

[ref49] Perdew J. P.; Ruzsinszky A.; Csonka G. I.; Constantin L. A.; Sun J. Workhorse Semilocal Density Functional for Condensed Matter Physics and Quantum Chemistry. Phys. Rev. Lett. 2009, 103, 026403 10.1103/PhysRevLett.103.026403. 19659225

[ref50] Luchini G.; Alegre-Requena J.; Funes-Ardoiz I.; Paton R. GoodVibes: automated thermochemistry for heterogeneous computational chemistry data [version 1; peer review: 2 approved with reservations]. F1000Research 2020, 9, 291 10.12688/f1000research.22758.1.

[ref51] Grimme S. Supramolecular Binding Thermodynamics by Dispersion-Corrected Density Functional Theory. Chem.—Eur. J. 2012, 18, 9955–9964. 10.1002/chem.201200497. 22782805

[ref52] Ramos-Cordoba E.; Postils V.; Salvador P. Oxidation States from Wave Function Analysis. J. Chem. Theory Computat. 2015, 11, 1501–1508. 10.1021/ct501088v. 26574361

