# Supplementary material for: Csp2–H Amination Reactions Mediated by Metastable Pseudo-Oh Masked Aryl-CoIII-nitrene Species
Source: Inorg Chem. 2022 Aug 23;61(35):14075–85. doi: 10.1021/acs.inorgchem.2c02111 (PMC9455280; doi:10.1021/acs.inorgchem.2c02111)
Supplement: Supplementary file 1 — ic2c02111_si_001.pdf [file ic2c02111_si_001.pdf]

Supporting information for

C_{sp2}-H amination reactions mediated by metastable pseudo-O_h masked aryl-Co^{III}-nitrene species

Lorena Capdevila,[†] Marc Montilla,[†] Oriol Planas,[†,ʃ] Artur Brotons,[†] Pedro Salvador,[†] Vlad Martin-Diaconescu,[∫] Teodor Parella,[º] Josep M. Luis,[†,*] Xavi Ribas[†,*]

[†] Institut de Química Computacional i Catàlisi (IQCC) and Departament de Química, Universitat de Girona, Campus Montilivi, Girona, E-17003, Catalonia, Spain.
[∫] ALBA Synchrotron, Cerdanyola del Vallès, E-08290, Catalonia, Spain.
[º] Servei de RMN, Facultat de Ciències, Universitat Autònoma de Barcelona, Campus UAB, Bellaterra, E-08193 Catalonia, Spain.
[ʃ] Current address: Queen Mary University of London, Mile End Road, London E1 4NS, UK.

Table of Contents

1. General considerations......S2
2. Synthesis of aryl-Co^{III}-X complexes (**1-OOCR**)......S4
3. Detection and isolation of reaction intermediates using **1-OAc** and organic azides (**a**-**c**)......S5
   3.1 Characterization of **2x-OAc**......S6
   3.2 Characterization of **3x-OAc**......S12
4. C-N bond formation reactions......S14
   4.1 Reactivity of **1-OAc** with organic azides at 50 ºC......S14
   4.2 Reactivity of **1-OAc** with organic azides at 100 ºC......S15
   4.3 Evaluation of different carboxylate anions......S18
5. Mechanistic insight......S21
   5.1 Inhibitory effect with TEMPO in **1-OAc** and benzyl azide reaction......S21
   5.2 Reactivity of **1-OAc** with benzyl amine to form **5-OAc**......S22
   5.3 Reactivity of **1-CH_3CN** with benzyl azide (**a**)......S22
   5.4 Kinetic Measurements......S23
   5.5 Reactivity of **1-OAc** and benzyl azide with xanthene......S24
   5.6 Evaluation of demetallation sources......S24
6. Synthesis of aryl-Rh^{III}-X complexes (**6_{Me}-OAc**)......S25
7. Reactivity of **1_{Me}-OAc** (Co^{III}) and **6_{Me}-OAc** (Rh^{III}) complex with organic azides......S25
8. XAS analysis of **1-OAc**, **4b-OAc** and **5-OAc** complexes......S28
9. Crystallographic data information......S31
10. Computational studies......S36
   10.1 EOS (Effective oxidation state) analysis......S36
   10.2 Analysis of the EFOs (Effective fragment orbitals)......S36
   10.3 Analysis of the MO-LCAO coefficients for nitrene (**INT-N**)......S38
   10.4 Study of the S=1 and S=2 states......S39
   10.5 Analysis of the triplet Nitrene (**INT-N**)......S39
   10.6 Attempts to find a stable penta-coordinated intermediate for Nitrene (**INT-N**)......S42
11. DFT XYZ coordinates of geometry optimized structures......S45

12. Original NMR data........S46

13. References........S90

1. General Considerations

Materials and methods

All reagents and solvents were purchased from Sigma Aldrich, Fisher Scientific or Fluorochem and used without further purification.^{1}H, ^{13}C{1H} and ^{19}F{H}-NMR spectra were recorded on Bruker 400 or 500 AVANCE spectrometer in the corresponding deuterated solvent ($CDCl_3$ or dmso d_6) and calibrated relative to the residual protons of the solvent. Quantification of reaction yields through integration of peaks was performed using an internal reference (1,3,5-trimethoxybenzene). High resolution mass spectra (HRMS) were recorded on a Bruker MicroTOF-Q IITM instrument using ESI source at Serveis Tècnics de Recerca, University of Girona. IR Spectra (FTIR) were recorded on a FT-IR Alpha spectrometer from Bruker with a PLATINUM-ATR attachment using OPUS software to process the data. UV-vis spectroscopy was performed with an Agilent 50 Scan (Varian) UV-vis spectrophotometer with 2 mm quartz cells. The ligands **L-H**, **L-Me** and the **1-CH_3CN** complex have been synthesized according to the procedure described in the literature.[1-3]

XAS Data Acquisition and processing

Samples were run as solid powders diluted in boron nitride at the ALBA synchrotron CLAESS beamline. Data was collected in transmission mode, at liquid nitrogen temperatures (80K), using a Si311 double crystal monochromator. Data was averaged; normalized and calibrated using the Athena software.[4] The energy was calibrated to the first inflection point of Co foil taken as 7709.5 eV. The autobk algorithm was used for EXAFS spectra extraction having a spline in the 1 to 13 Å-1 region with an Rbkg of 1.1 for **1-OAc** and **4b-OAc** and R_{bkg} of 1 for **5-OAc**. The FEFF6 code[5-6] was used for scattering path generation, and k^3-weighted fits of the data were carried out in r-space over an r-range of 1-3.0 Å and a k-range of 2-12.0 Å^{-1} unless otherwise specified, using the Artemis software.[6] The S_0^2 value was set to 0.9, and a global E_0 was employed with the initial E_0 value set to the inflection point of the rising edge. Single scattering paths were fit in terms of a Δr_{eff} and σ^2 as previously described. To assess the goodness of the fits both the R_{factor} (%R) and the reduced χ^2 (χ^2_v) were minimized, ensuring that the data was not over-fit. Pre-edge features were fit using a Gaussian-Lorentzian sum function with 50% Gaussian character.

Computational details

All DFT calculations have been carried out using the GAUSSIAN16 program.[7] Geometry optimizations have been performed without any symmetry restrictions, considering the effect of the HFIP solvent (via the Self-Consistent Reaction Field –SCRF– method using the SMD solvation model,[8] and also taking into account dispersion effects with the Grimme and coworkers DFT-D3BJ correction[9-10] at the BP86-D3BJ(SMD)/Def2SVP level of theory.[11-14]

Note that the HFIP solvent is not implemented in GAUSSIAN16, so we performed those calculations using the *Solvent=Generic,Read* options for the *SCRF* keyword. The *Minnesota Solvent Descriptor Database*[15] explains that, using the universal solvation model as described by Cramer et al.,[16] one needs a set of descriptors which must be provided in order to use a custom/non-parameterized solvent using Gaussian's SCRF. The required descriptors are:

n	index of refraction at optical frequencies at 293K. Sometimes called n_{20}^{D}. Note that for this descriptor, the GAUSSIAN input requires the user to enter the square of this value, rather than the value itself.
α	Abraham's hydrogen bond acidity. In Abraham's notation: $\sum \alpha_2^H$
β	Abraham's hydrogen bond basicity. In Abraham's notation: $\sum \beta_2^H$
γ	Macroscopic surface tension at a liquid-air interface at 298K. In cal·mol^{-1}·Å^{-2}, but dimensionless. Note that 1 dyne/cm = 1.43932 cal·mol^{-1}·Å^{-2}.
ε	Dielectric constant at 298K.
ϕ	Aromaticity: out of all non-hydrogen atoms, fraction which are aromatic carbons.
ψ	Electronegative halogenicity: out of all non-hydrogen atoms, fraction which are F, Cl, or Br.
n_{25}	same as n, but at 298K.

And the corresponding values for HPIF are:

n	1.275 [17]
α	0.77 [18]
β	0.10 [19]
γ	23.23 [20]
ε	16.7 [21]
ϕ	0.000
ψ	0.600
n_{25}	-

After optimization of the geometries with this procedure, analytical frequency calculations have been performed at the same level of theory, to evaluate enthalpy and entropy corrections at 298.15K, and to ensure that all frequencies were positive for the minima, while having only one negative frequency for the transition states (corresponding with the expected atom displacement for the chemical process). All points in the reaction pathway have been connected via IRC.

Single point calculations on the equilibrium geometries, including the solvent and dispersion effects (E_{sp}), have been carried out at the revTPSS-D3BJ(SMD)/Def2TZVP level of theory.[22] Note that the D3BJ parameters for the revTPSS functional are not currently implemented in GAUSSIAN16, so they were included using the corresponding IOp keywords: *IOp(3/174=1000000) IOp(3/175=1402300) IOp(3/177=442600) IOp(3/178=4472300).*[21]
Then, the total Gibbs Energy values (G) are given by:

$$G = E_{sp} + G_{corr.} + \Delta G^{\circ/*} \qquad (1)$$

where the Gibbs Energy correction ($G_{corr.}$) has been obtained from the thermodynamical analysis at the optimization level of theory, but corrected using the GoodVibes code[23] so that frequencies below 100 are not treated with the Harmonic Approximation, but rather with the Quasi-Harmonic Approximation as described by Grimme.[24] Finally, the additional correction term $\Delta G^{\circ/*}$ accounts for the transition from the standard state concentration (gas phase, pressure of 1 atm) to the concentrations used experimentally.[25] The Gibbs energy correction obtained performing a frequency calculation with the GAUSSIAN code is based on the statistical thermodynamics formula for the Gibbs energy of a molecule in the gas phase at the standard pressure of 1 atm. From the ideal gas approximation, and at 298.15 K, these standard conditions equate to a volume of 24.465 L, and thus a concentration of 0.041 M. However, the studied experimental reactions are carried out in the liquid phase with the reactants and catalysts at some different specific concentration. The $\Delta G^{\circ/*}$ term accounts for the Gibbs energy change due to the change in the entropy due to the isothermal expansion/compression from 0.041 M to a final volume that corresponds to the experimental concentration of the different reactants and catalysts. For instance, for a hypothetical experimental concentration of 1 M, the $\Delta G^{\circ/*}$correction is 1.894 kcal/mol. In practice, this correction is calculated for each of the species depending on their experimental concentrations using Eq. (2). For example, the benzyl azide used in the reaction has an experimental concentration of 0.1 M, and thus the correction is $\Delta G^{\circ/*} = 0.530$ kcal/mol.

$$\Delta G^{\circ/*} = RT \ln(V^*/V^0) \quad (2)$$

In the above equation, R is the universal gas constant, T is the temperature, V^o is the volume occupied by one mol of an ideal gas at a pressure of 1 atm (i.e., 24.465 L), and V^* is the volume occupied by an ideal gas at the concentration of x mol/L (i.e., $V^* = 1/x$ L).

Benchmark for the choice of density functional approximation to perform the Single-Point Calculations

We performed a benchmark study of Gibbs energy barriers of the nitrene formation step (i.e., **TS1** barrier) using two different basis sets and nine different DFAs. The equilibrium geometries of **1-OAc·NBz** and **TS1** were optimized at the BP86-D3BJ/Def2SVP level, and the "level of theory" of the table indicates the method used in the single point calculations required to compute the Gibbs energy barriers presented in the table below. The Gibbs energy values were computed using Eq. (1).

**Level of theory**	**TS1 Barrier (Gibbs Free Energy, kcal/mol)**
BP86-D3BJ/Def2SVP	26.0
BP86-D3BJ/Def2TZVP	24.2
TPSS-D3BJ/Def2TZVP	24.7
revTPSS-D3BJ/Def2TZVP	24.2
M06L-D3/Def2TZVP	32.6
B3LYP-D3BJ/Def2SVP	37.2
TPSSh-D3BJ/Def2TZVP	31.0
MN15-D3BJ/Def2TZVP	35.6
ωB97XD/Def2TZVP	40.8
ωB97XD/Def2SVP	43.4
M11/Def2TZVP	44.7

The relatively small energy difference between the barriers obtained with BP86-D3BJ/Def2-SVP and BP86-D3BJ/Def2-TZVP basis sets suggests that the latter basis set is flexible enough to determine the Gibbs energy profile of the mechanism of the studied reaction.

The single point calculations of the nitrene formation barrier show stronger dependence on the choice of the DFA. However, the metaGGA rev-TPSS functional used to perform the single-point calculations of the profile shown in Figure 3 of the main manuscript and GGA BP86 used to optimize the geometries lead to the same value for the nitrene formation barrier (i.e., 24.2 kcal/mol). The barrier obtained with the TPSS has also a similar value. Conversely, the hybrid functionals such as B3LYP,

TPSSh, or MN15, and the range-separated functionals like ωB97XD or M11 lead to too high barriers with respect to the experimental data. The results obtained suggest that all the DFAs that include Exact Hartree-Fock Exchange give too high barriers for this step.

Effective oxidation state (EOS) analysis

The concept of oxidation state (OS) is unavoidably related to the electron distribution around the atoms, which can nowadays be described to an unprecedented precision with modern electronic structure methods. The effective oxidation state (EOS) analysis introduced by Ramos-Cordoba et al. is formally applicable to any molecular system and wavefunction on equal footing.[26] The scheme uses Mayer's effective fragment orbitals (EFOs) and their occupation numbers (λ), obtained independently for each atom/ligand and for each spin case. These spin-resolved EFOs are sorted by decreasing occupation number and individual electrons are assigned to them until the total number of electrons is reached. This procedure leads to an effective configuration of each ligand/atom, and hence its OS. The difference in the occupation between the last occupied (LO) and first unoccupied (FU) EFOs indicates to which extent the electron distribution can be pictured as a discrete ionic model. A simple reliability index, R (%) = min (R_α, R_β), can be introduced for each spin case σ as

$$R_\sigma(\%) = 100 \cdot \min(1, max(0, \lambda_{LO}^{\sigma} - \lambda_{FU}^{\sigma} + 1/2)). \quad (2)$$

The OS assignment is considered as undisputable when the difference in occupation of the frontier EFOs exceeds half electron, leading to R = 100. When the two frontier EFOs from different fragments exhibit the same occupation number, two different equally plausible OS distributions are present with R = 50.

Besides the OS assignation and the associated R value, the visual inspection of the EFOs is also useful in order to determine which atom/ligand orbitals hold the individual electrons (or electron pairs). For each formal bond between two fragments, one finds one (and only one) pair of complementary EFOs on each of them representing the dangling valence of the split bond with occupations that add approximately to one for each spin channel.

In order to obtain the EFOs, an underlying atomic partitioning scheme is necessary. The shape of the EFOs is rather insensitive to partitioning, while the occupation numbers may vary. The partial ionic character of the bonds is better captured by schemes such as QTAIM or topological fuzzy Voronoi cells (TFVC), as compared to Mulliken's or Hirshfeld's schemes, and therefore QTAIM and TFVC schemes are better suited for OS assignation.

2. Synthesis of aryl-Co^{III}-X complexes (1-OOCR)

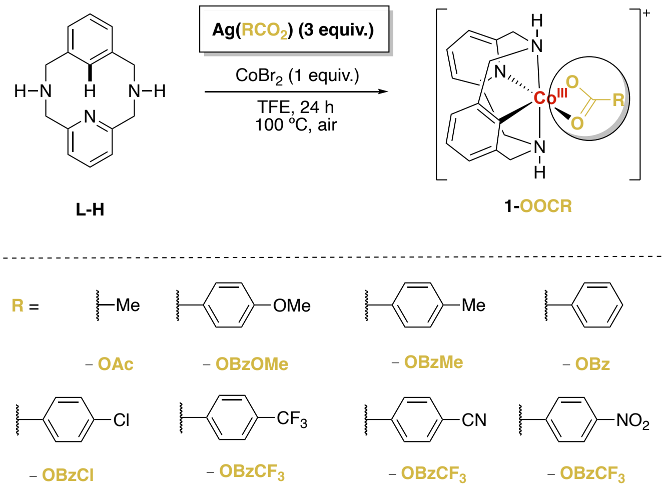

Scheme S1. Synthesis of aryl-Co^{III}-X complexes (**1-OOCR**).

The **1-OOCR** complexes have been synthesized according to the procedure described in the literature.[2-3] To a solution of **L-H** ligand (50 mg, 0.21 mmol) in a 10 mL vial, Ag(RCOO$_2$) (0.63 mmol) and CoBr$_2$ (45.9 mg, 0.21 mmol) were mixed in TFE (2.5 mL). The vial was sealed with a septum and warmed up to 100 °C. After 24 h, the solvent was removed, and the mixture was dissolved in CHCl$_3$ and layered with pentane at 4 °C. The resulting oil was dried under vacuum during 6 h affording the corresponding **1-OOCR** organometallic complex. Recrystallization with CHCl$_3$ layered with pentane gave the corresponding complex **1-OOCR**, which was characterized by NMR and X-Ray spectroscopy.
The NMR data of **1-OAc, 1-OBzOMe, 1-OBzMe, 1-OBz, 1-OBzCl, 1-OBzCN** and **1-OBzNO$_2$** was in agreement with the previous reports.[2-3]

- *Aryl-Co^{III}-(OBz-CF$_3$)$_2$ – **(1-OBzCF$_3$)***

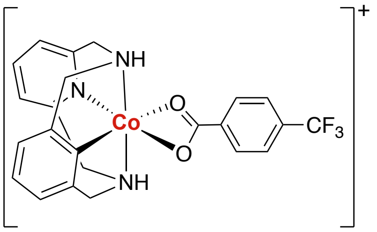

Red foam (74%, 105.2 mg). **^{1}H NMR** (400 MHz, CHCl$_3$, ppm): 8.24 (bs, 2H), 7.73 (d, ^3J = 7.7 Hz, 2H), 7.52 (m, 3H), 7.36 (d, ^3J = 7.7 Hz, 2H), 7.19 (bs, 2H), 7.06 (t, ^3J = 7.0 Hz, 1H), 7.00 – 6.94 (m, 4H), 5.01 (dd, ^2J = 16.7 Hz, ^3J = 7.0 Hz, 2H), 4.92 (dd, ^2J = 15.4 Hz, ^3J = 6.7 Hz, 2H), 3.95 – 3.88 (m, 4H). **^{13}C {1H} NMR** (100 MHz, CHCl$_3$, ppm): δ 174.7 (1C), 169.4 (1C), 161.7 (2C), 148.5 (2C), 138.8 (1C), 137.5 (1C), 131.5 (1C), 129.2 (2C), 124.6 (1C), 124.4 (2C), 124.3 (CF3), 120.7 (2C), 118.7 (2C), 62.5 (2C), 62.2(2C). **^{19}F-NMR** (400 MHz, CDCl3, ppm): -62.6, -62.8. **HRMS** (ESI) calc. for $C_{23}H_{20}F_3CoN_3O_2^+$ [M – (OBzCF$_3$)]$^+$: 486.0834; found 486.0828. **EA:** $C_{31}H_{24}F_6CoN_3O_4$ · 0.5(HOBzCF$_3$)·1(H_2O): calc. C 53.85 N 5.38 H 3.68%, exp. C 53.83 N 5.43 H 3.67%.

3. Detection and isolation of reaction intermediates using 1-OAc and organic azides (a-c)

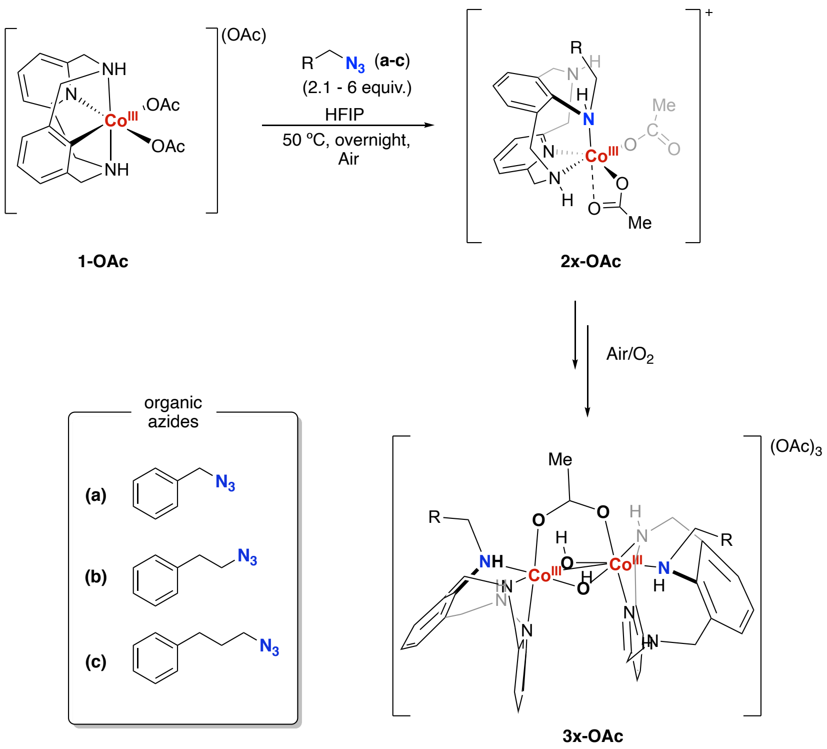

Scheme S2. Detection of **2x-OAc** and isolation of the corresponding **3x-OAc**.

In a 2 mL vial, **1-OAc** (20 mg, 0,048 mmol) and organic azide **a-c** (2.1 - 6 equiv.) were mixed in HFIP (1 mL) and the vial was sealed. The mixture was heated at 50 °C overnight. The crude was concentrated under vacuum line until the initial volume was reduced to two-thirds. The crude mixture was analyzed by ^{1}H-NMR (CDCl$_3$) and HRMS observing the formation of the reaction intermediates (**2x-OAc**). Then, recrystallization with CHCl$_3$ layered with pentane under air the corresponding dimeric species (**3x-OAc**) was slowly formed which were characterized by NMR.

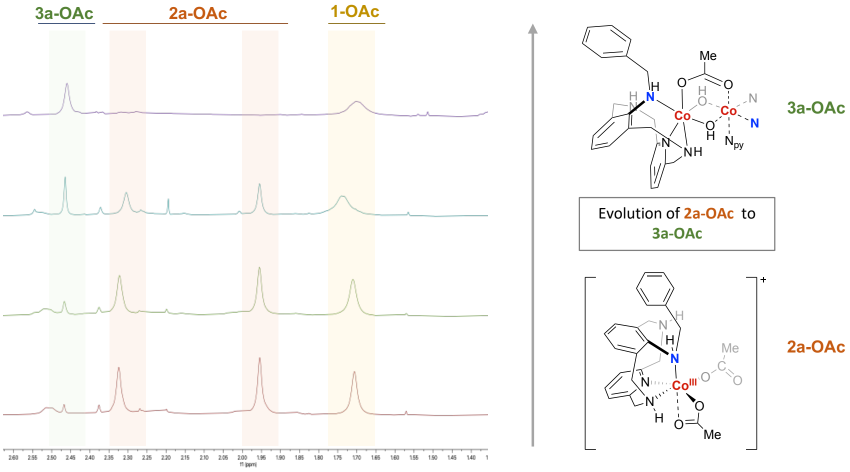

Figure S1. ^{1}H-NMR spectra (acetate region) of **2a-OAc** crude mixture yielding the **3a-OAc** complex.

*3.1. Characterization of **2x-OAc***

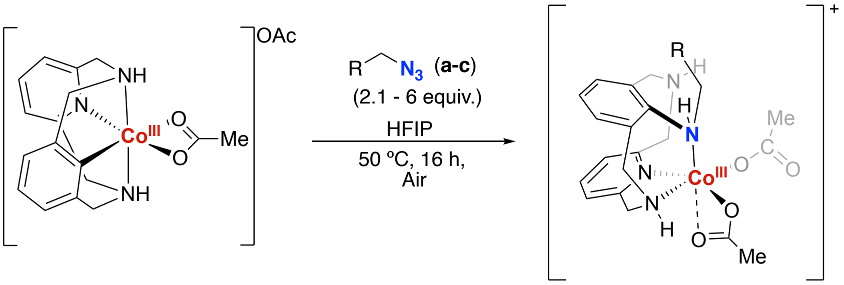

1-OAc **2x-OAc**

Scheme S3. Formation of **2x-OAc** from **1-OAc** and organic azide (**a-c**)

- *Aryl-(NHCH$_2$Ph)-Co^{III} – (**2a-OAc**)*

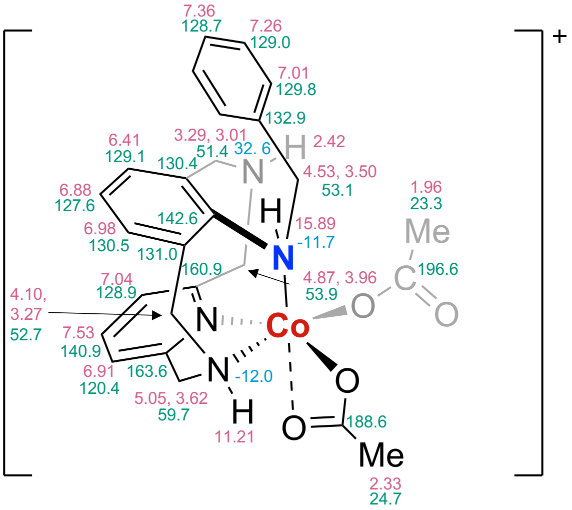

The **2a-OAc** complex was synthetized according to the procedure described above using 2.1 equivalents of benzyl azide (**a**) in a 46 % NMR yield. **^{1}H NMR** (500 MHz, CHCl$_3$, ppm): 15.89 (d, ^3J = 8.9 Hz, 1H), 11.21 (s, 1H), 7.53 (t, ^3J = 7.6 Hz, 1H), 7.36 (1H), 7.26 (1H), 7.04 (d, ^3J = 7.6 Hz, 1H), 7.01 (d, ^3J = 7.6 Hz, 2H), 6.98 (1H), 6.91 (d, ^3J = 8.0 Hz, 1H), 6.88 (t, ^3J = 7.6 Hz, 1H), 6.41 (d, ^3J = 7.6 Hz, 1H). 5.05 (dd, ^2J = 16.4 Hz, ^3J = 6.7 Hz, 1H), 4.87 (d, ^2J = 14.3 Hz, 1H), 4.53 (d, ^2J = 13.0 Hz, 1H), 4.10 (1H), 3.96 (d, ^2J = 14.2 Hz, 1H), 3.62 (d, ^2J = 16.2 Hz, 1H), 3.50 (1H), 3.29 (d, ^2J = 14.8 Hz, 1H), 3.27 (d, ^2J = 14.8 Hz,

1H), 3.01 (d, ^2J = 14.6 Hz, 1H), 2.42 (bs, 1H), 2.33 (s, 3H), 1.96 (s, 3H). **^{13}C {1H} NMR** (125 MHz, $CHCl_3$, ppm): δ 196.6, 188.6, 163.6, 160.9, 142.6, 140.9, 132.9, 131.0, 130.4, 129.8, 129.1, 129.0, 128.9, 128.7, 127.6, 120.4, 59.7, 53.9, 52.7, 51.4, 24.7, 23.3. **^{15}N-NMR** (50.7 MHz): -12.0, -11.7, 32.6. **HRMS** (ESI) calc. for $C_{26}H_{30}CoN_4O_4^+$ [M – OAc]$^+$: 521.1594, found 521.1598.

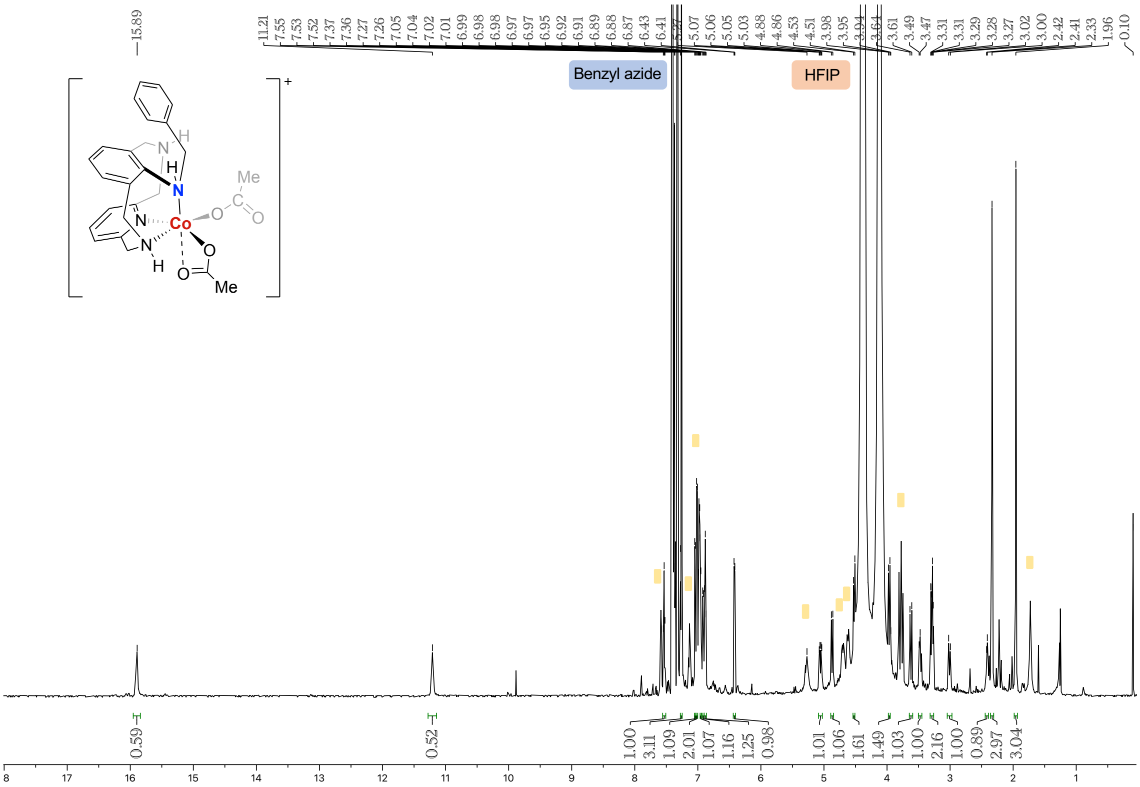

Figure S2. 500 MHz ^{1}H NMR spectra of **2a-OAc** crude mixture in $CHCl_3$, 298 K. In yellow was depicted the unreactive **1-OAc**.

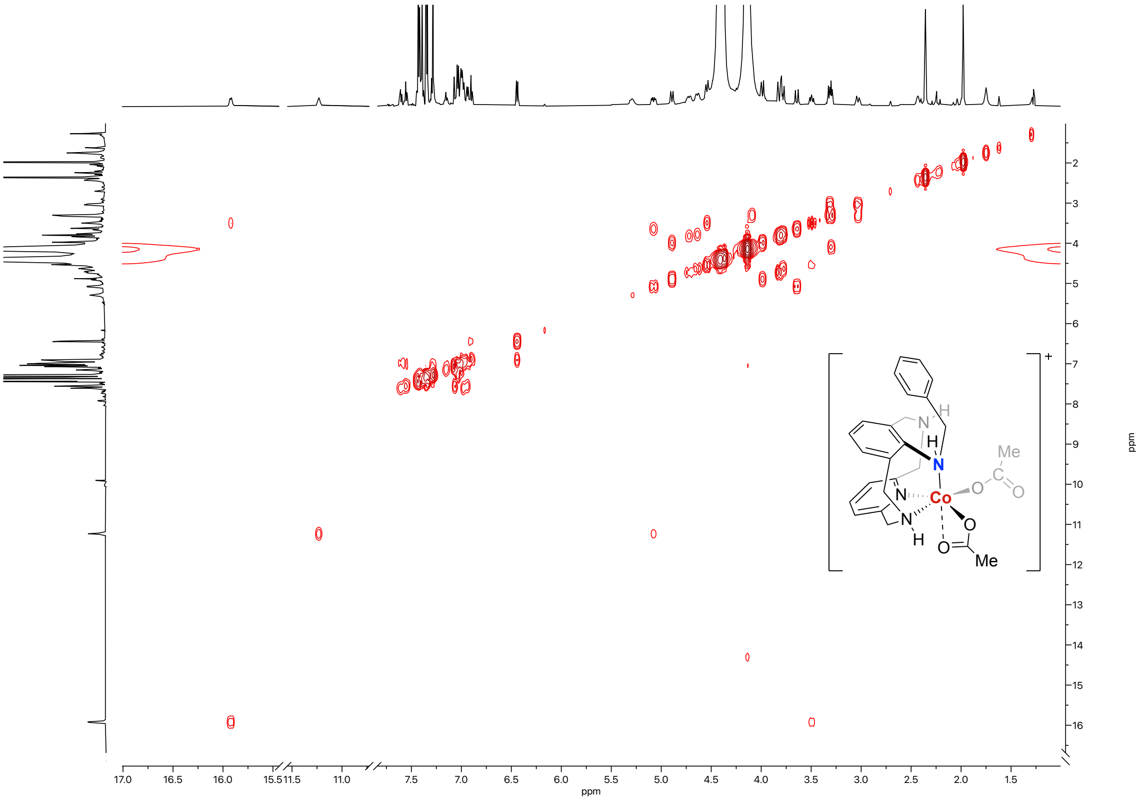

Figure S3. 500 MHz ^{1}H-^{1}H COSY spectrum of **2a-OAc** crude mixture in $CHCl_3$, 298 K.

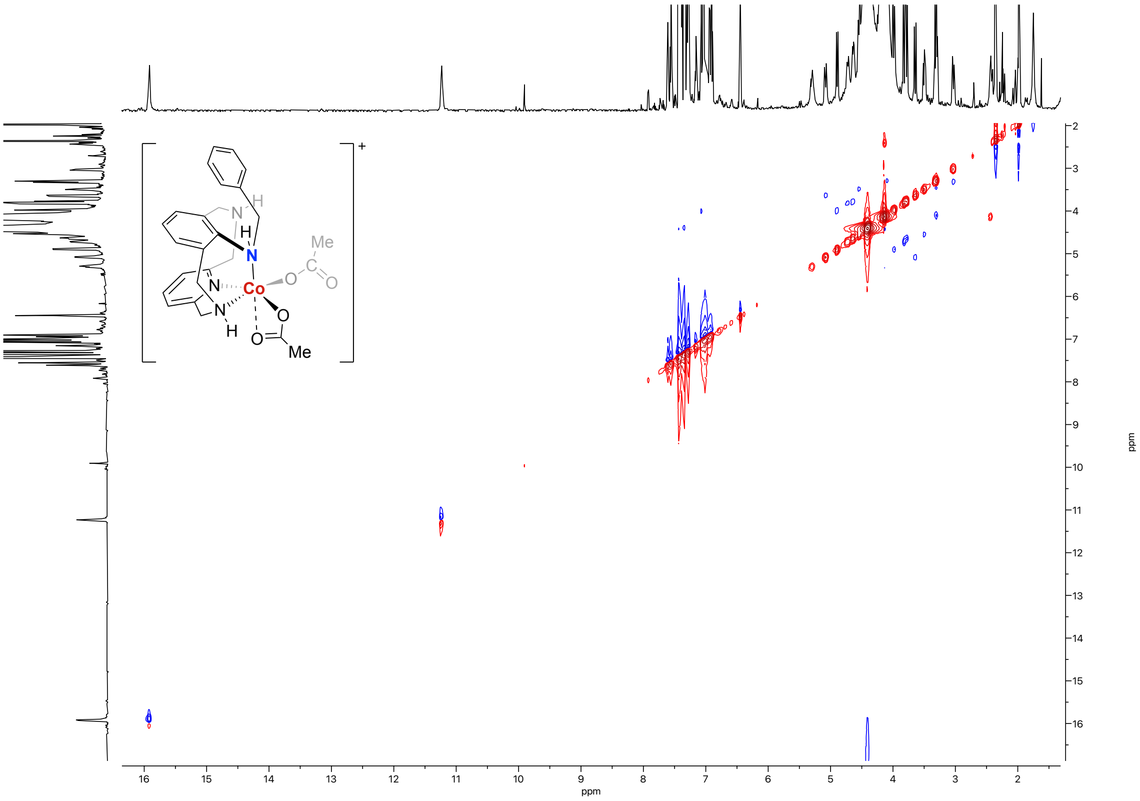

Figure S4. 500 MHz ^{1}H-^{1}H NOESY spectrum of **2a-OAc** crude mixture in $CHCl_3$, 298 K.

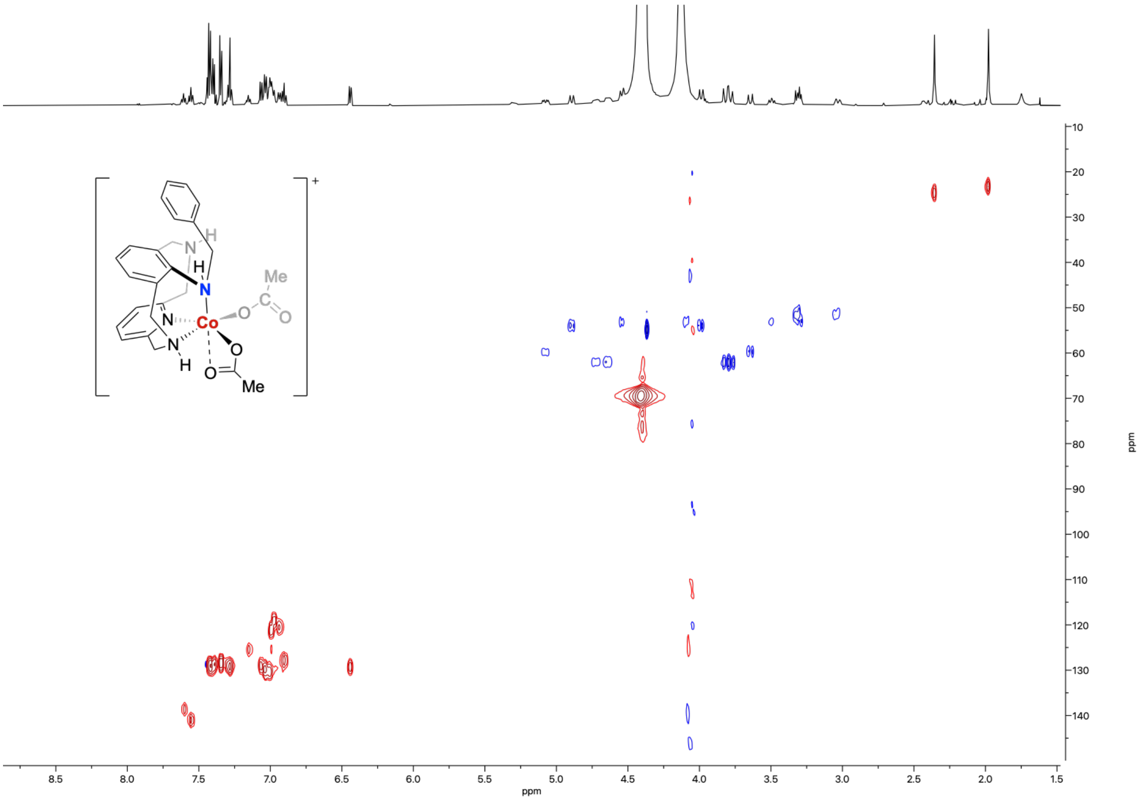

Figure S5. 500 MHz ^{1}H-^{13}C HSQC spectrum of **2a-OAc** crude mixture in $CHCl_3$, 298 K.

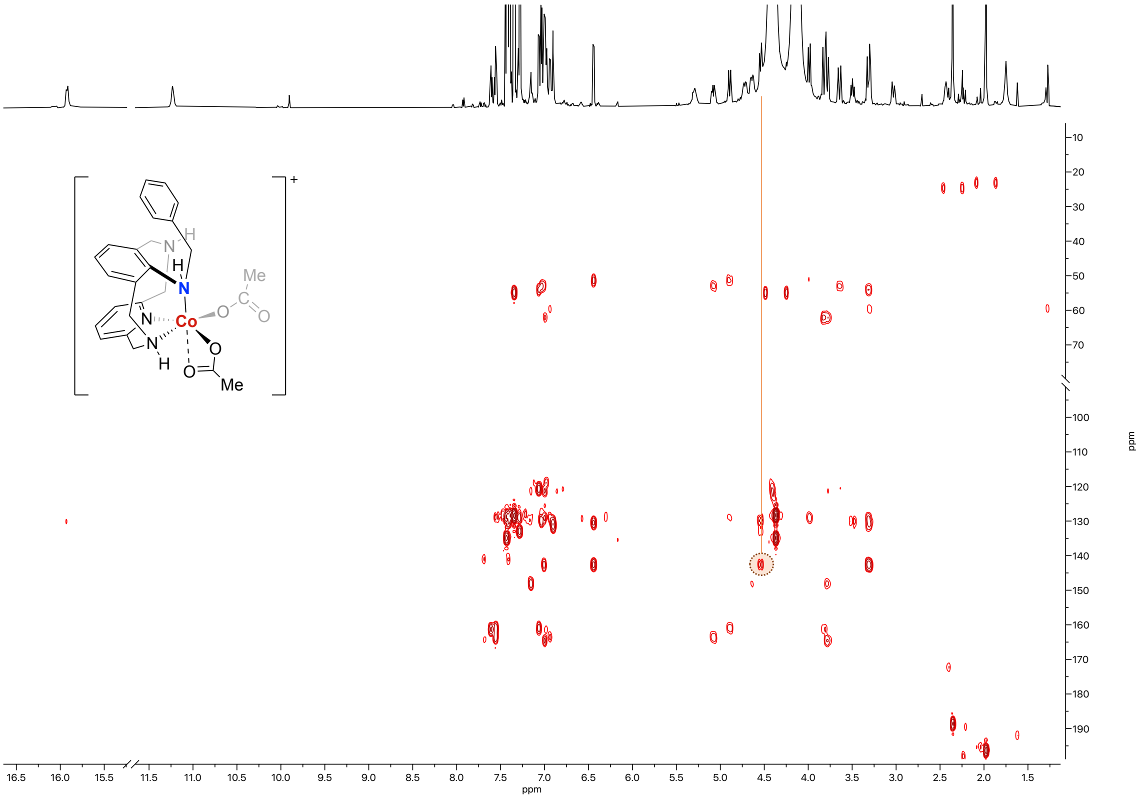

Figure S6. 500 MHz ^{1}H-^{13}C HMBC spectrum of **2a-OAc** crude mixture in $CHCl_3$, 298 K.

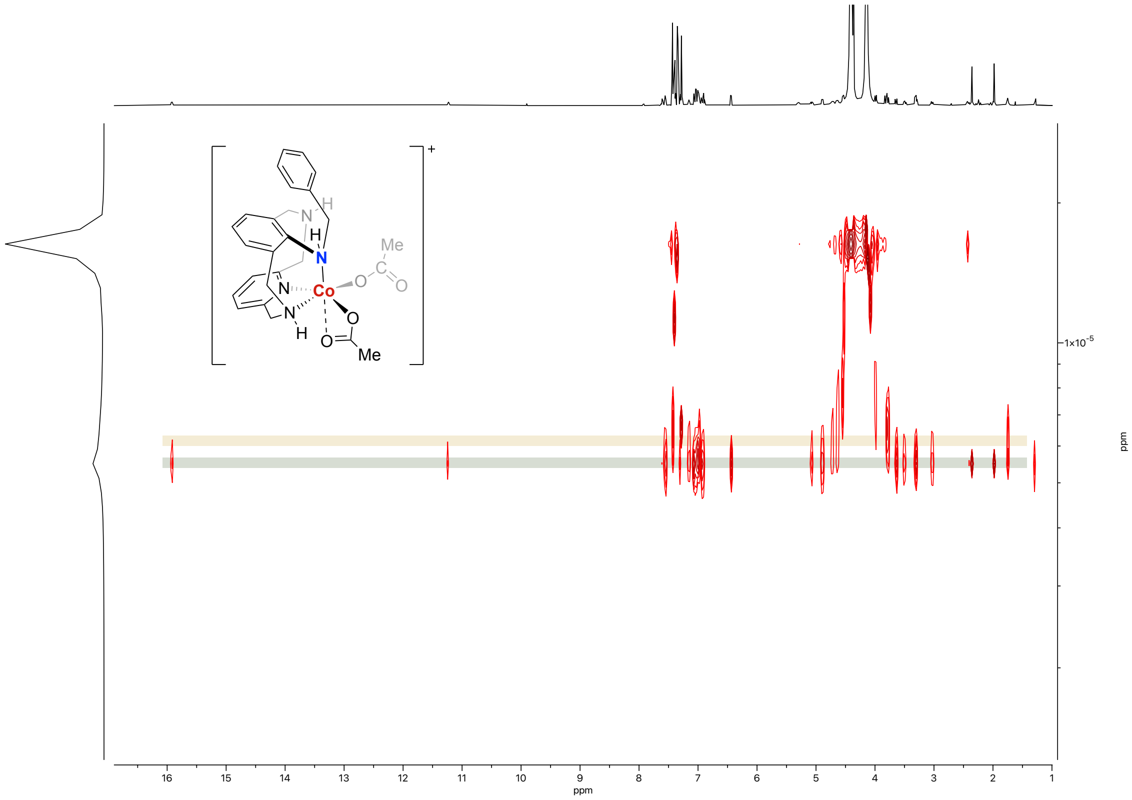

Figure S7. 500 MHz DOSY spectrum of **2a-OAc** crude mixture in $CHCl_3$, 298 K. In yellow was depicted the unreactive **1-OAc**.

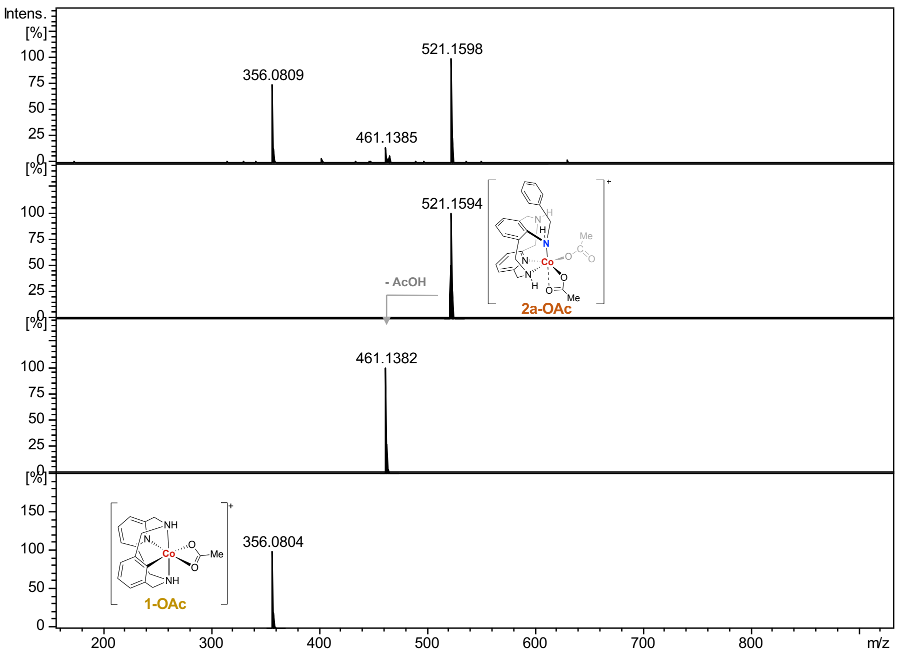

Figure S8. HRMS spectrum of **2a-OAc** crude mixture showing a peak at m/z = 521.1598.

- *Aryl-(NH(CH$_2$)$_2$Ph)-Co^{III}* – ***(2b-OAc)***

The excess of azide (**b**) needed (6 equiv.) in the reaction using **1-OAc**, makes the characterization of the **2b-OAc** complex crude difficult. The –NH and the –OAc peaks were in agreement with the analogous **2a-OAc** complex. Furthermore, the formation of **2b-OAc** was further confirmed by HRMS (see figure S9 and S10).

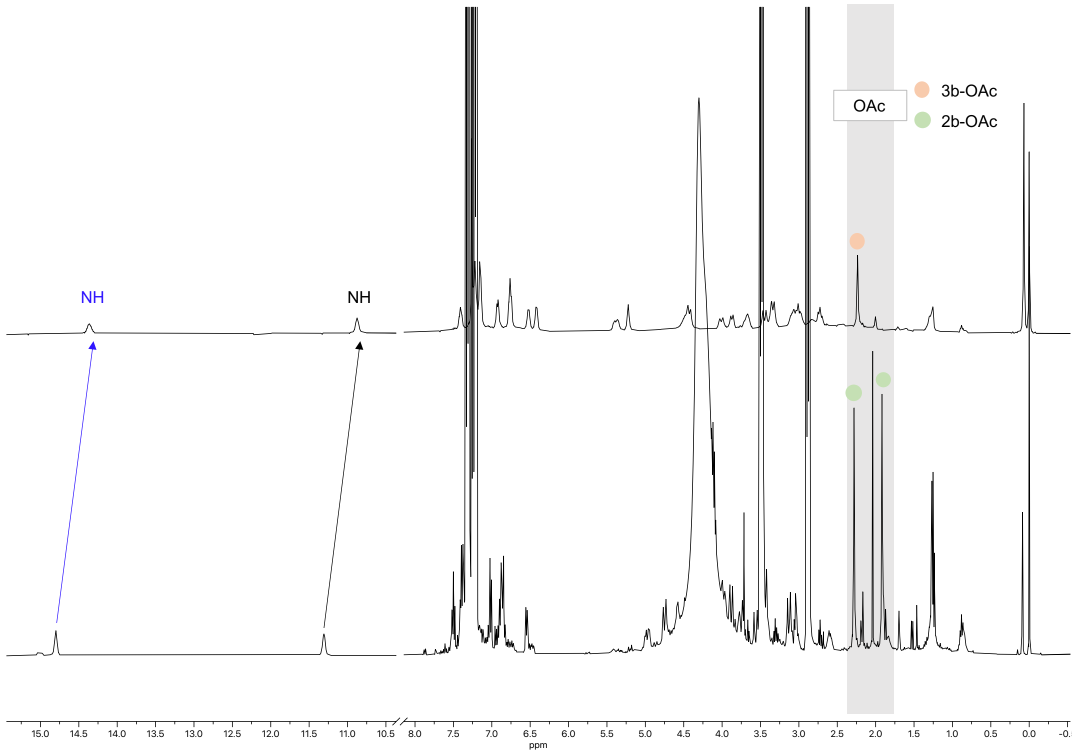

Figure S9. ^{1}H-NMR spectra obtained after reaction of **1-OAc** with organic azide, 400 MHz, 298 K (**b**) showing the formation of **2b-OAc** (bottom) and its evolution to form the **3b-OAc** (top).

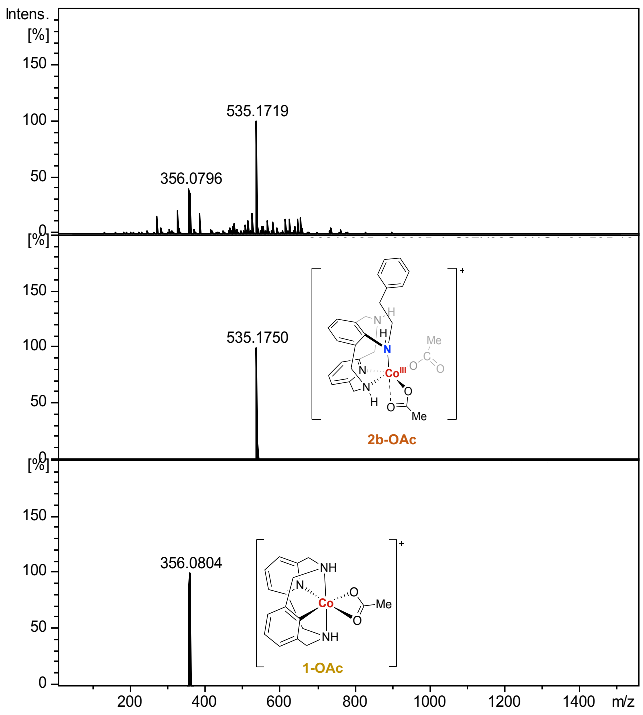

Figure S10. HRMS obtained after reaction of **1-OAc** with organic azide (**b**). Mass analysis show a peak at *m/s* = 535.1719 which corresponds to **2b-OAc**.

- *Aryl-(NH(CH_2)$_3$Ph)-Co^{III} – **(2c-OAc)***

As in the case of using azide (**b**), the excess of azide (**c**) used (6 equiv.) in the reaction using **1-OAc** makes the characterization of the **2c-OAc** complex crude difficult. The –NH and the –OAc peaks were in agreement with the analogous **2a-OAc** complex. Furthermore, the formation of **2c-OAc** was further confirmed by HRMS (see Figure S11 and S12).

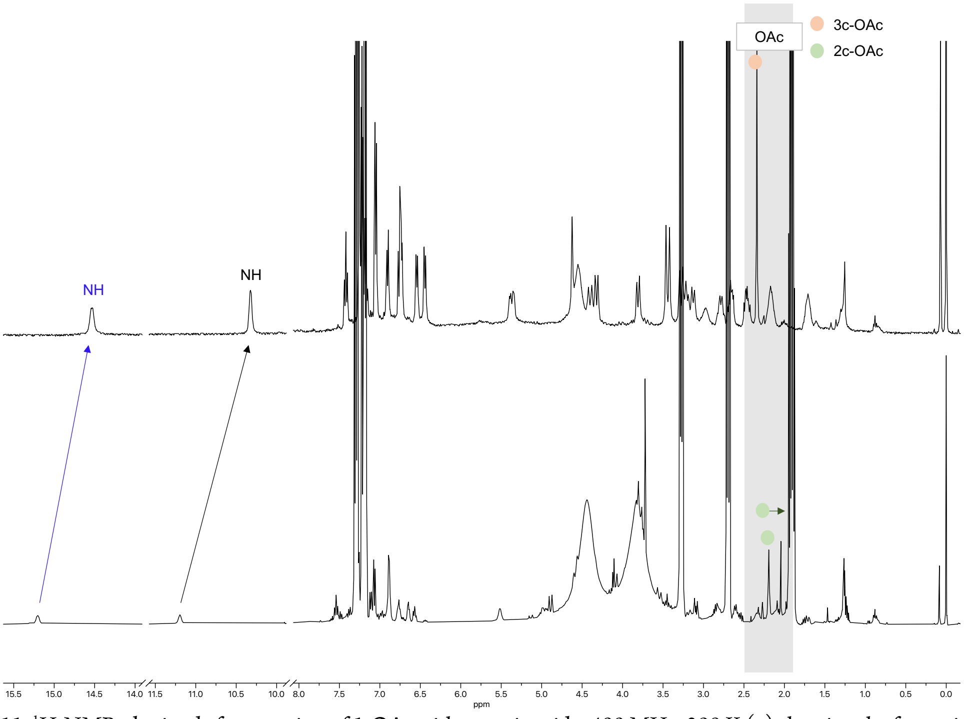

Figure S11. ^{1}H-NMR obtained after reaction of **1-OAc** with organic azide, 400 MHz, 298 K (**c**) showing the formation of **2c-OAc** (bottom) and its evolution to form the **3c-OAc** (top)

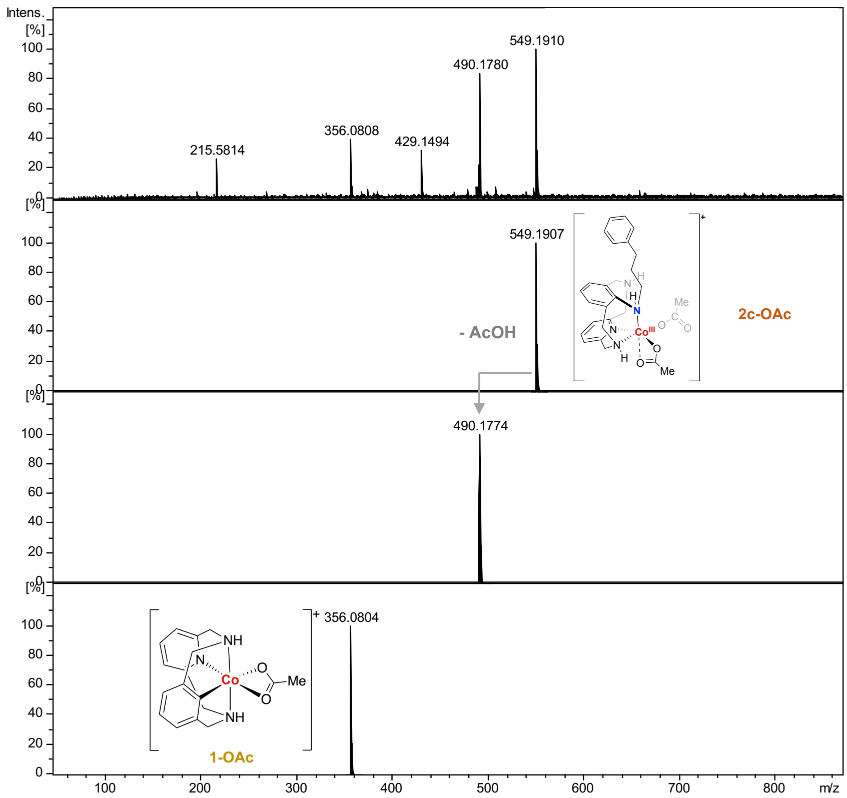

Figure S12. HRMS obtained after reaction of **1-OAc** with organic azide (**c**). Mass analysis show a peak at *m/z* = 549.1910 which corresponds to **2c-OAc**.

*3.2 Characterization of **3x-OAc***

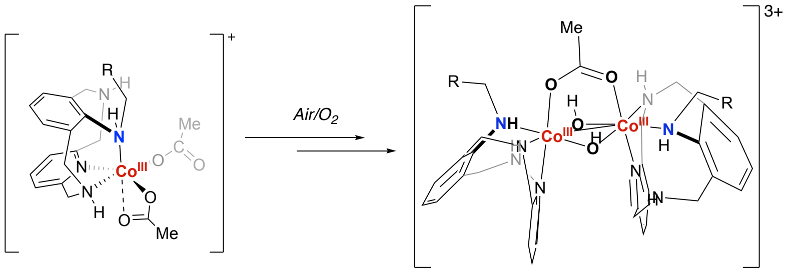

Scheme S4. Formation of **3x-OAc** from **2x-OAc**

- *[Aryl-(NHCH$_2$Ph)-Co^{III}]$_2$* – ***(3a-OAc)***

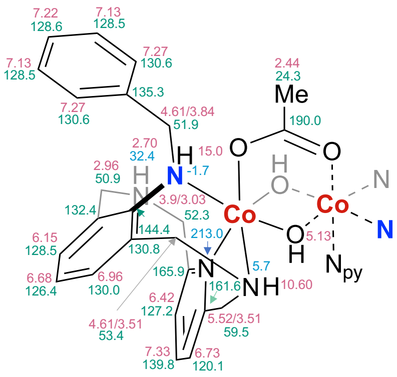

Red oil (9.6 mg, 0.0089 mmol, 81% based on **2a-OAc;** 95% NMR yield based on **2a-OAc**). **^{1}H NMR** (500 MHz, CHCl$_3$, ppm): 15.02 (bs, 2H), 10.60 (bs, 2H), 7.33 (t, ^{3}J = 7.6 Hz, 2H), 7.23-7.22 (m, 6H), 7.13 (m, 4H), 6.96 (d, ^{3}J = 7.2 Hz, 2H), 6.73 (d, ^{3}J

= 7.4 Hz, 2H), 6.68 (t, ^3J = 7.2 Hz, 2H), 6.42 (d, ^3J = 7.6 Hz, 2H), 6.15 (d, ^3J = 7.2 Hz, 2H), 5.52 (dd, ^3J = 6.7 Hz, ^3J = 17.4 Hz, 2H), 5.14 (s, 2H), 6.61 (m, 4H), 3.90 (m, 2H), 3.84 (m, 2H), 3.51 (m, 4H), 3.03 (m, 2H), 2.96 (s, 4H), 2.70 (bs, 2H), 2.44 (s, 3H). **^{13}C {1H} NMR** (125 MHz, $CHCl_3$, ppm): δ 190.3 (2C), 165.9 (2C), 161.6 (2C), 144.4 (2C), 139.8 (2C), 135.3 (2C), 132.4 (2C), 130.8 (2C), 130.6 (4C), 130.0 (2C), 128.6 (2C), 128.5 (6C), 127.2 (2C), 126.4 (2C), 120.1 (2C), 59.5 (2C), 53.4 (2C), 52.3 (2C), 51.9 (2C), 50.9 (2C), 24.6 (1C). **HRMS** (ESI) calc. for $C_{50}H_{59}Co_2N_8O_8^+$ [M-OAc]$^+$: 1017.3119; found: 1017.3121. **EA:** $C_{52}H_{62}Co_2N_8O_{10}$ · 2.5($CHCl_3$)·2.5($C_3H_2F_6O$): calc. C 41.3 N 6.39 H 3.9%, exp. C 41.47 N 6.24 H 3.90%.

- *[Aryl-(NH(CH$_2$)$_2$Ph)- Co^{III}]$_2$* – ***(3b-OAc)***

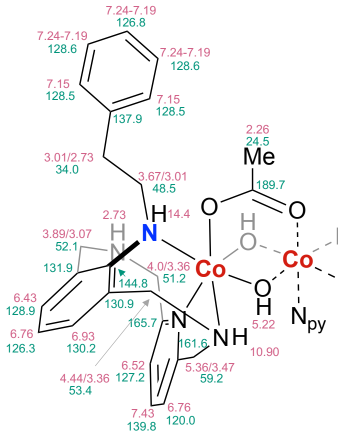

Red oil (4.8 mg, 0.0043 mmol, 70% based on **2b-OAc**; 75% NMR Yield based on **2b-OAc**). **^{1}H NMR** (400 MHz, $CHCl_3$, ppm): 14.36 (bs, 2H), 10.88 (bs, 2H), 7.41 (t, ^3J = 7.1 Hz, 2H), 7.24-7.19 (m, 6H), 7.15 (m, 4H), 6.93 (d, ^3J = 7.1 Hz, 2H), 6.76 (m, 4H), 6.53 (d, ^3J = 7.1 Hz, 2H), 6.43 (d, ^3J = 7.1 Hz, 2H), 5.36 (m, 2H), 5.22 (s, 2H), 4.44 (m, 2H), 4.03 (d, ^3J = 15.2 Hz, 2H), 3.90 (d, ^3J = 12.1 Hz, 2H), 3.67 (m, 2H), 3.47 (d, ^3J = 17.2 Hz, 2H), 3.36 (m, 4H), 3.07-3.01 (m, 6H), 2.83 (bs, 2H), 2.73 (m, 2H), 2.24 (s, 3H). **^{13}C {1H} NMR** (100 MHz, $CHCl_3$, ppm): δ 189.7, 165.7, 161.6, 144.8, 139.8, 137.9, 131.9, 130.9, 130.2, 128.9, 128.6, 128.5, 127.2, 126.8, 126.3, 120.0, 59.2, 53.4, 52.2, 51.2, 48.5, 34.0, 24.5. **HRMS** (ESI) calc. for $C_{52}H_{63}Co_2N_8O_8^+$ [M-OAc]$^+$: 1045.3427; found: 1045.3440. **EA:** $C_{54}H_{66}Co_2N_8O_{10}$ · 2.2($CHCl_3$)·0.3($C_3H_2F_6O$): calc. C 48.36 N 7.90 H 4.89%, exp. C 48.39 N 7.98 H 4.71%.

- *[Aryl-(NH(CH$_2$)$_3$Ph)- Co^{III}]$_2$* – ***(3c-OAc)***

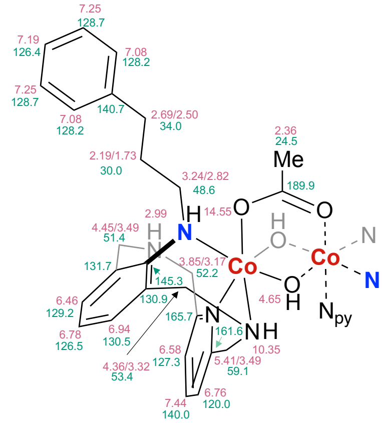

Red oil (5.0 mg, 0.0044 mmol, 73% based on **2c-OAc**; 97% NMR Yield based on **2c-OAc**).**^{1}H NMR** (400 MHz, $CHCl_3$, ppm): 14.55 (bs, 2H), 10.35 (bs, 2H), 7.44 (t, ^3J = 7.2 Hz, 2H), 7.25 (m, 4H), 7.19 (m, 2H), 7.08 (d, ^3J = 7.4 Hz, 4H), 6.94 (d, ^3J = 7.2, 2H), 6.78 (t, ^3J = 7.7 Hz, 2H), 6.76 (d, ^3J = 7.4 Hz, 2H), 6.58 (d, ^3J = 7.2 Hz, 2H), 6.46 (d, ^3J = 7.2 Hz, 2H), 5.40 (dd, ^2J = 17.3 Hz, ^3J = 7.2. Hz, 2H), 4.05 (s, 2H), 4.45 (d, ^2J = 15.6 Hz, 2H), 4.36 (d, ^2J = 14.3 Hz, 2H), 3.85 (d, ^2J = 13.1 Hz, 2H), 3.49 (d, ^2J = 17.3 Hz, 2H), 3.32 (bs, 2H), 3.24 (bs, 2H), 3.17 (d, ^2J = 13.9 Hz, 2H), 2.99 (bs, 2H), 2.82 (bs, 2H), 2.69 (bs, 2H), 2.50 (bs, 2H), 2.36 (s, 3H), 2.19 (bs, 2H). **^{13}C {1H} NMR** (100 MHz, $CHCl_3$, ppm): δ 189.8, 165.7, 145.3, 140.7, 140.0, 131.7, 130.9, 130.5, 129.2, 128.7, 128.2, 127.3, 126.5, 126.4, 120.0, 59.1, 53.4, 52.2, 51.4, 48.6, 34.0, 30.0, 24.5. **HRMS** (ESI) calc. for $C_{54}H_{67}Co_2N_8O_8$ $^+$ [M-OAc]$^+$: 1073.3740, found 1073.3792. **EA:** $C_{56}H_{70}Co_2N_8O_{10}$ · 2.2($CHCl_3$)·1($C_3H_2F_6O$): calc. C 47.00 N 7.17 H 4.78%, exp. C 47.13 N 7.31 H 4.54%.

4. C-N bond formation reactions

*4.1 Reactivity of **1-OAc** with organic azides at 50 ºC*

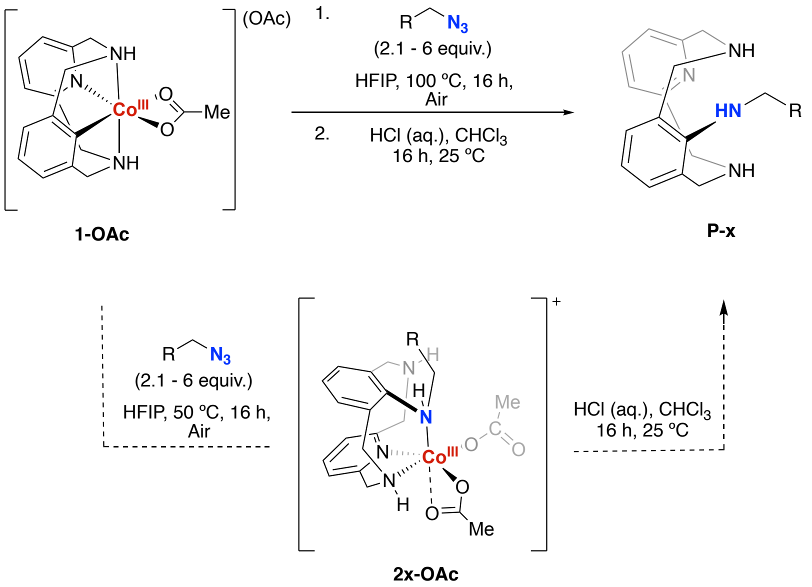

Scheme S5. Formation of lineal **P-a**, **P-b** and **P-c** product.

In a 2 mL vial, **1-OAc** (20 mg, 0,048 mmol) and organic azide **a-c** (2.1 – 6 equiv.) were mixed in HFIP (1 mL) and the vial was sealed. The mixture was heated at 50 ºC overnight. The crude was concentrated under vacuum line until the initial volume was reduced to two-thirds. Then, the crude mixture was dissolved in $CHCl_3$ and HCl (3 mmol, 2M) was added. After stirring overnight, the crude was basified until pH 14 and extracted with $CHCl_3$. Products were purified using neutral alumina column chromatography ($CHCl_3$, then $CHCl_3$/MeOH 8:2) and characterized by NMR techniques.

- ***P-a***

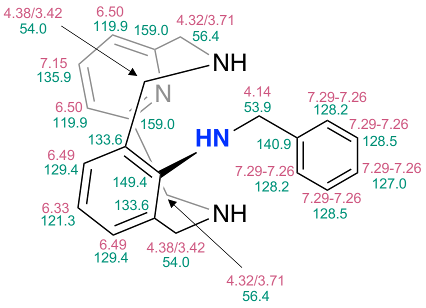

Yellow solid (7.3 mg, 0.021 mmol, 44%). **^{1}H NMR** (400 MHz, $CHCl_3$, ppm): 7.29-7.26 (m, 5H), 7.15 (t, ^3J = 7.7 Hz, 1H), 6.50 (d, ^3J = 7.6 Hz, 2H), 6.49 (d, ^3J = 7.4 Hz, 2H), 6.33 (t, ^3J = 7.4 Hz, 2H), 4.38 (d, ^2J = 14.5 Hz, 2H), 4.32 ((d, ^2J = 15.4 Hz, 2H), 4.14 (s, 2H), 3.71 (d, ^2J = 15.4 Hz, 2H), 3.42 (d, ^2J = 14.5 Hz, 2H). **^{13}C {1H} NMR** (100 MHz, $CHCl_3$, ppm): δ 159.0 (2C), 149.4 (1C), 140.9 (1C), 135.9 (1C), 133.6 (2C), 129.4 (2C), 128.5 (2C), 128.3 (2C), 127.0 (1C), 121.3 (1C), 119.9 (2C), 56.4 (2C), 54.1 (2C), 53.9 (1C). **HRMS** (ESI) calc. for $C_{22}H_{24}N_4$ $[M+H]^+$: 345.2074; found: 345.2075.

- ***P-b***

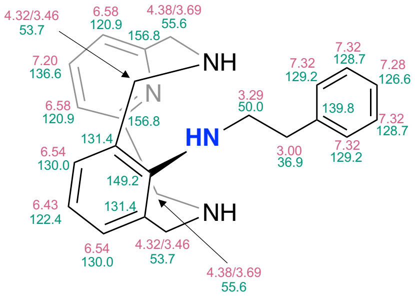

Yellow solid (6.8 mg, 0.019 mmol, 40%). **¹H NMR** (400 MHz, $CHCl_3$, ppm): 7.35-7.28 (m, 5H), 7.20 (t, ^{3}J = 7.7 Hz, 1H), 6.58 (d, ^{3}J = 7.9 Hz, 2H), 6.54 (d, ^{3}J = 7.9 Hz, 2H), 6.43 (t, ^{3}J = 7.30, 1H), 4.38 (d, ^{2}J = 15.3 Hz, 2H), 4.32 (d, ^{2}J = 14.6 Hz, 2H), 3.69 (d, ^{2}J = 3.7 Hz, 2H), 3.46 (d, ^{2}J = 14.6 Hz, 2H), 3.29 (t, ^{3}J = 6.9 Hz, 2H), 3.0 (t, ^{3}J = 6.9 Hz, 2H). **¹³C {1H} NMR** (100 MHz, $CHCl_3$, ppm): δ 156.8 (2C), 149.2, 139.8, 136.6, 131.4 (2C), 130.0 (2C), 129.2 (2C), 128.7 (2C), 126.6, 122.4, 120.9 (2C), 55.6 (2C), 53.7 (2C), 50.0, 36.9. **HRMS** (ESI) calc. for $C_{23}H_{26}N_4$ $[M+H]^+$: 359.2230; found: 359.2234.

- ***P-c***

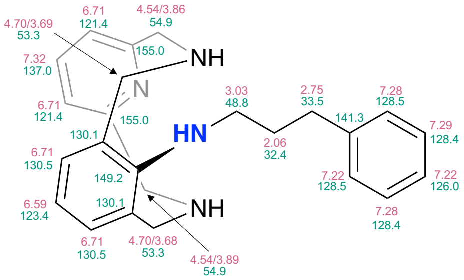

Yellow solid (7.7 mg, 0.020 mmol, 42%). **¹H NMR** (400 MHz, $CHCl_3$, ppm): 7.32-7.28 (m, 3H), 7.23-7.20 (m, 3H), 6.72-6.70 (m, 4H), 6.59 (t, ^{3}J = 7.3 Hz, 1H), 4.70 (d, ^{2}J = 14.4 Hz, 2H), 4.54 (d, ^{2}J = 15.5 Hz, 2H), 3.86 (d, ^{2}J = 15.5 Hz, 2H), 3.69 (d, ^{2}J = 14.4 Hz, 2H), 3.03 (t, ^{3}J = 7.7 Hz, 2H), 2.75 (t, ^{3}J = 7.5 Hz, 2H), 2.06 (quint, ^{3}J = 7.1 Hz, 2H). **¹³C {1H} NMR** (100 MHz, $CHCl_3$, ppm): δ 155.0, 149.2, 141.3, 137.0, 130.5, 130.1, 128.5, 128.4, 126.0, 123.4, 121.5, 54.9, 53.4, 48.8, 33.5, 32.4. **HRMS** (ESI) calc. for $C_{24}H_{28}N_4$ $[M+H]^+$: 373.2387; found: 373.2394.

*4.2 Reactivity of **1-OAc** with organic azides at 100 ºC*

*4.2.1 Using benzyl azide (**a**)*

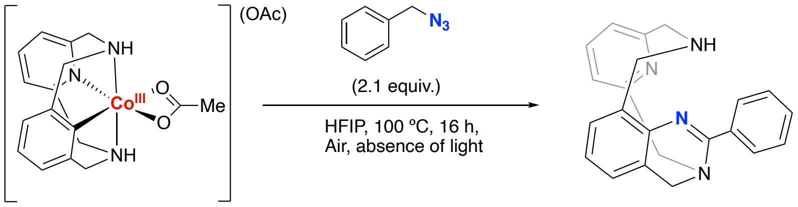

1-OAc **P-a-cyc**

Scheme S6. Reactivity of **1-OAc** and benzyl azide to form **P-a-cyc** product.

In a 2 mL vial, **1-OAc** (20 mg, 0,048 mmol) and organic azide **a** (2.1 equiv.) were mixed in HFIP (1 mL) and the vial was sealed and heated at 100ºC during 16 h in the absence of light. Then, after removal of the solvent, the product **P-a-cyc** was purified using neutral alumina column chromatography ($CHCl_3$, then $CHCl_3$/MeOH 8:2) and characterized by NMR.

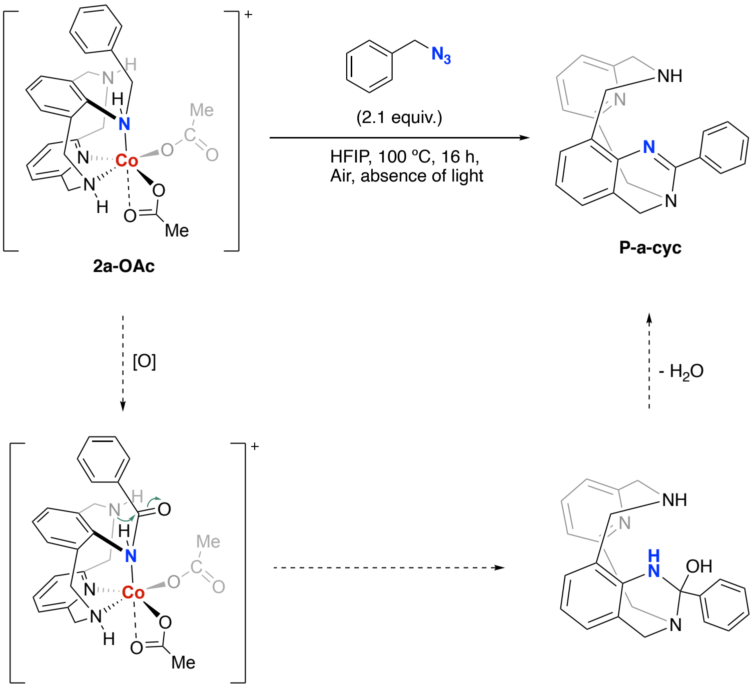

Scheme S7. Proposed mechanism for the intramolecular cyclization to form the **P-a-cyc** product. The absence of air/O_2 (N_2 atmosphere) is detrimental since yield drops from 27% to 5%.

Table S1. Reaction of **1-OAc** with benzyl azide (**a**) using different conditions.

Entry	Solvent	Atmosphere	Yield of P-a-cyc (%)[a]
1	TFE	Air	34% (31 %)
2	HFIP	Air	(27%)
3	HFIP	O_2	28%
4	HFIP	N_2	5%[b]

[a]*Yields are calculated using 1,3,5-trimethoxybenzene as internal standard. Isolated yield in parenthesis.*
[b]*Reaction was performed using Schlenck flask.*

- ***P-a-cyc***

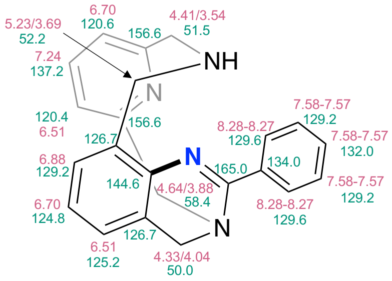

Yellow solid (4.4 mg, 0.013, 27%). **¹H NMR** (400 MHz, $CHCl_3$, ppm): 8.28-8.27 (m, 2H), 7.58-7.57 (m, 3H), 7.24 (t, ^3J = 7.2 Hz, 1H), 6.88 (d, ^3J = 7.3 Hz, 1H), 6.74-6.69 (m, 2H), 6.52-6.50 (m, 2H), 5.23 (d, ^2J = 13.6 Hz, 1H), 4.64 (d, ^2J = 15.1 Hz, 1H), 4.41 (d, ^2J = 16.9 Hz, 1H), 4.33 (d, ^2J = 15.6 Hz, 1H), 4.04 (d, ^2J = 15.6 Hz, 1H), 3.88 (d, ^2J = 15.1 Hz, 1H), 3.69 (d, ^2J = 13.6 Hz, 1H), 3.54 (d, ^2J = 16.9 Hz, 1H). **¹³C {1H} NMR** (100 MHz, $CHCl_3$, ppm): 165.0 (1C), 156.6 (2C), 144.6 (1C), 137.2 (1C), 134.0 (1C), 132.0 (1C), 129.6 (2C), 129.2 (3C), 126.7 (2C), 125.2 (1C), 124.8 (1C), 120.6 (1C), 120.4 (1C), 58.4 (1C), 52.2 (1C), 51.5 (1C), 50.0 (1C). **HRMS** (ESI) calc. for $C_{22}H_{20}N_4$ [M+H]⁺: 341.1761, found 341.1751.

*4.2.2 Using (2-azidoethyl)benzene (**b**)*

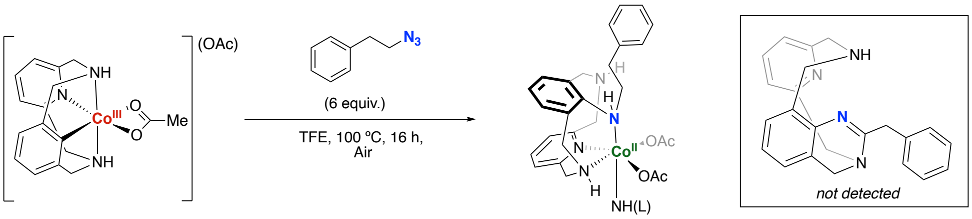

1-OAc **4b-OAc, 30%**

Scheme S8. *Reaction of **1-OAc** with organic azide (**b**) forming the **4b-OAc** complex.*

In a 2 mL vial, **1-OAc** (20 mg, 0,048 mmol) and organic azide **b** (6 equiv.) were mixed in TFE (1 mL) and the vial was sealed and heated at 100 ºC during 16 h. Then, the solvent was removed and suitable crystals of **4b-OAc** were grown by pentane diffusion in a concentrated solution in CH_2Cl_2 anhydrous under inert atmosphere affording **4b-OAc** in 30% isolated yield (7.7 mg, 0.014 mmol).

^{1}H NMR (400 MHz, $CHCl_3$, ppm): 225.98, 204.88, 196.48, 51.77, 49.45, 44.92. **HRMS** (ESI) calc. for $C_{25}H_{29}CoN_4O_2$ $^+$ [M-OAc]$^+$: 476.1617, found 476.1618. **EA:** $C_{27}H_{32}CoN_4O_4 \cdot 0.01(H_2O) \cdot 0.65(CH_2Cl_2)$: calc. C 56.39 N 9.69 H 5.64%, exp. C 56.20 N 9.48 H 5.71%.

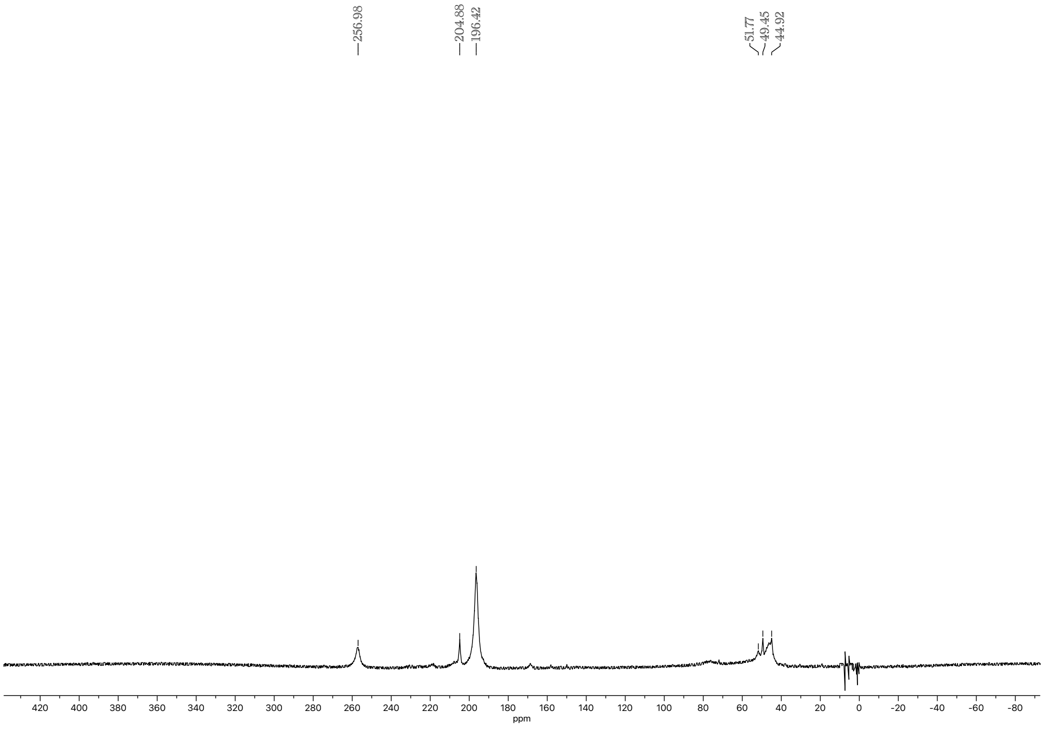

Figure S13. 400 MHz ^{1}H-NMR spectrum of **4b-OAc** in $CDCl_3$, 298 K.

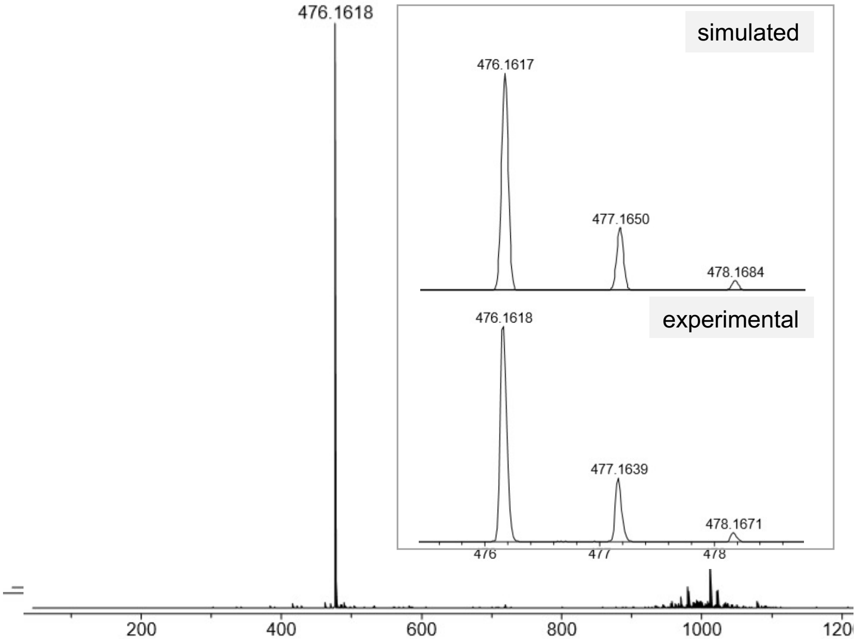

Figure S14. HRMS spectrum of **4b-OAc** showing a peak at m/z = 476.1618.

4.3 Evaluation of different carboxylate anions

*4.3.1 **2a-OOCR** complexes*

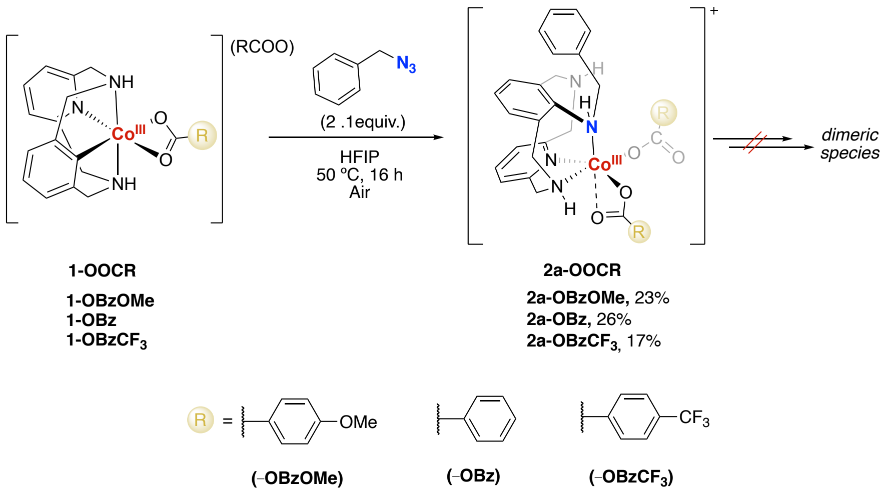

Scheme S9. Formation of **2a-OOCR** complexes. NMR yields are calculated using 1,3,5-trimethoxybenzene as internal standard.

In a 2 mL vial, **1-OOCR** (0.048 mmol) and benzyl azide (**a**) (0.1 mmol, 2.1 equiv.) were mixed in HFIP (1 mL) and the vial was sealed. The mixture was heated at 50 ºC overnight. The crude was concentrated under vacuum line until the initial volume was reduced to two-thirds. The crude mixture was analyzed by ^{1}H-NMR and HRMS observing the formation of the corresponding **2a-OOCR** complex. Faster decomposition was observed compared to the analogous **2a-OAc** complex and no dimeric species was detected.

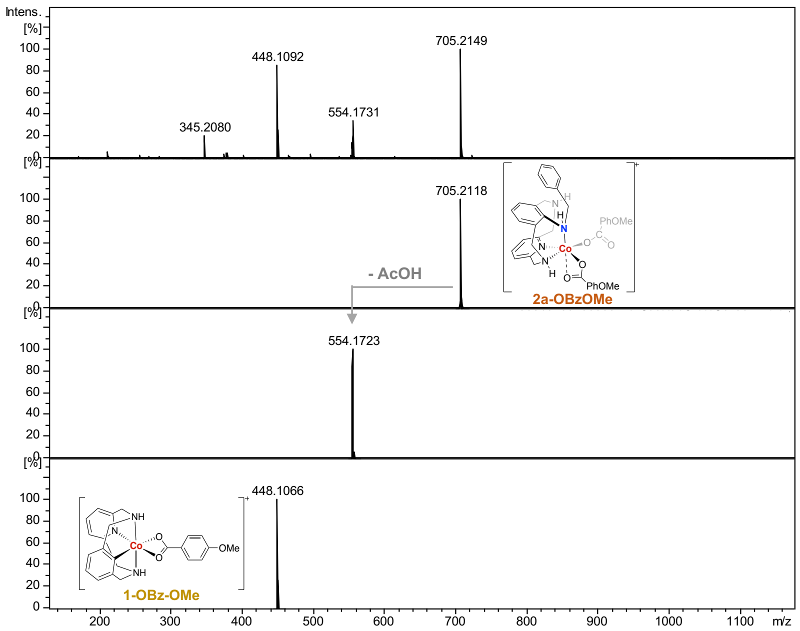

Figure S15. HRMS obtained after reaction of **1-OBzOMe** with benzyl azide (**a**). Mass analysis show a peak at *m/z* = 705.2149 which corresponds to **2a-OBzOMe** complex.

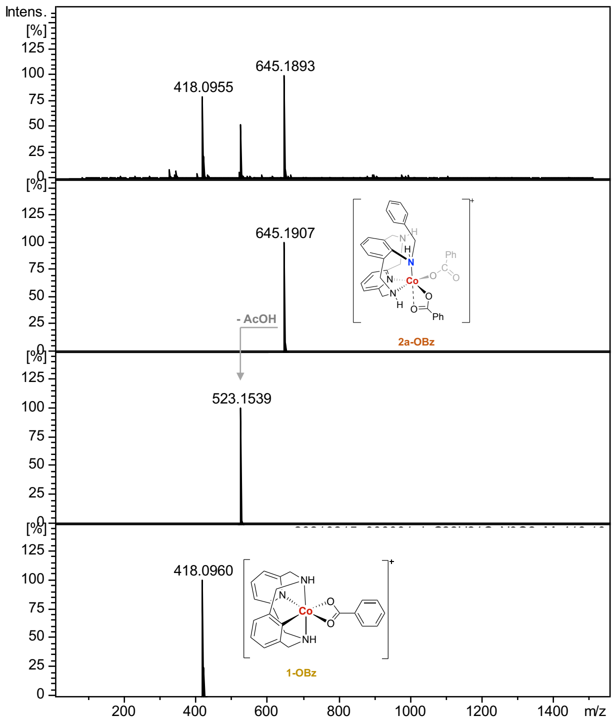

Figure S16. HRMS obtained after reaction of **1-OBz** with benzyl azide (**a**). Mass analysis show a peak at *m/z* = 645.1893 which corresponds to **2a-OBz** complex.

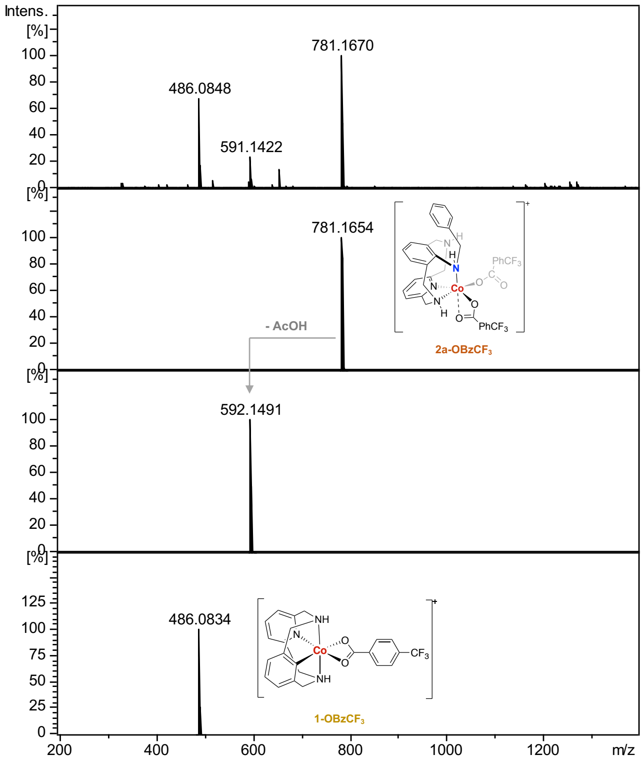

Figure S17. HRMS obtained after reaction of **1-OBzCF$_3$** with benzyl azide (**a**). Mass analysis show a peak at *m/z* = 781.1670 which corresponds to **2a-OBzCF$_3$** complex.

*4.3.2 **P-a** formation using **1-OOCR** complexes*

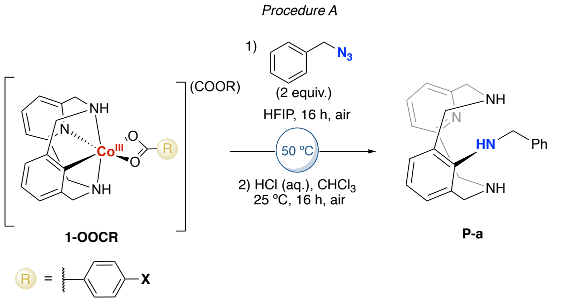

X	Yield P-a (%)
OMe	25%
H	37%
CF$_3$	19%

Scheme S10. Evaluation of different carboxylate anions in **P-a** formation reaction at 50°C

Procedure A: In a 2 mL vial, **1-OOCR** (0.048 mmol) and benzyl azide (**a**) (0.1 mmol, 2.1 equiv.) were mixed in HFIP (1 mL) and the vial was sealed. The mixture was heated at 50 °C overnight. The crude was concentrated under vacuum line until the initial volume was reduced to two-thirds. Then, the crude mixture was dissolved in 1 mL of $CHCl_3$ and HCl (3 mmol, 2M) was added. After stirring overnight, the crude was basified until pH 14 and extracted with $CHCl_3$. Products were purified using neutral alumina column chromatography ($CHCl_3$, then $CHCl_3$/MeOH 8:2) and characterized by NMR techniques.

4.3.3 ***P-a-cyc*** *formation using* ***1-OOCR*** *complexes*

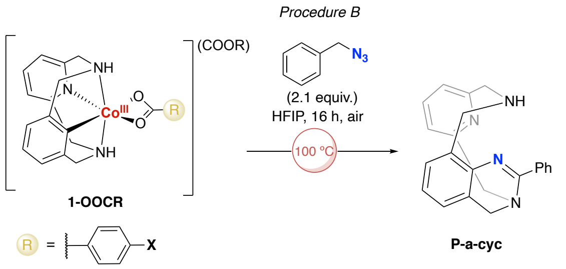

X	Yield P-a-cyc (%)
OMe	42
Me	17
H	34
Cl	49
CF_3	23
CN	24
NO_2	16

Scheme S11. Evaluation of different carboxylate anions in **P-a-cyc** formation.

Procedure B: In a 2 mL vial, **1-OOCR** (0.048 mmol) and benzyl azide (**a**) (0.1 mmol, 2.1 equiv.) were mixed in HFIP (1 mL) and the vial was sealed and heated at 100ºC during 16 h in the absence of light. Then, after removal of the solvent, the product was purified using neutral alumina column chromatography ($CHCl_3$, then $CHCl_3$/MeOH 8:2) and analyzed by NMR using 1,3,5-trimethoxybenzene as internal standard.

5. Mechanistic insight

5.1 Inhibitory effect with TEMPO in ***1-OAc*** *and benzyl azide reaction*

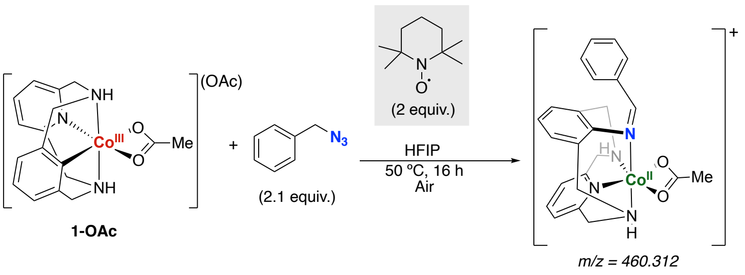

Scheme S12. Addition of TEMPO in the reaction of **1-OAc** with benzyl azide at 50 ºC.

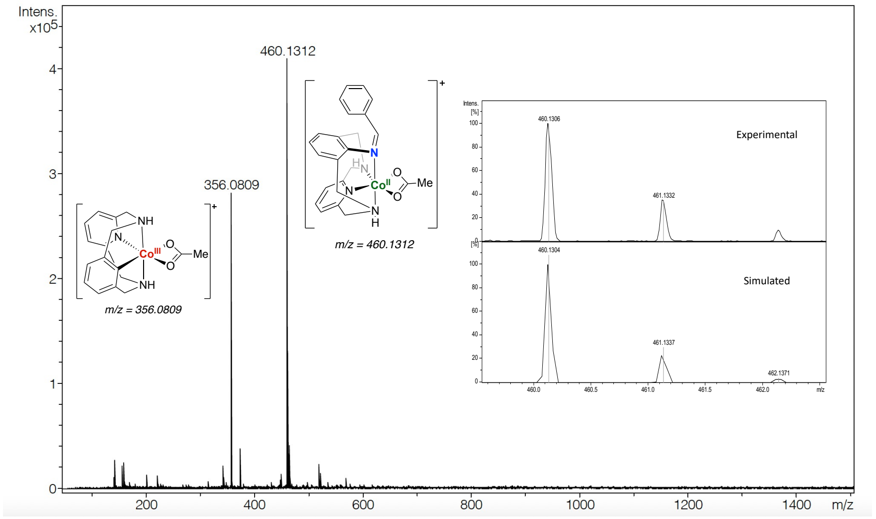

Figure S18. HRMS obtained after reaction of **1-OAc** with organic azide (**a**) and TEMPO. Mass analysis show a peak at *m/z* = 460.1312 which is tentatively assigned to Co^{II} inserted imine complex.

*5.2 Reactivity of **1-OAC** with benzyl amine to form **5-OAc***

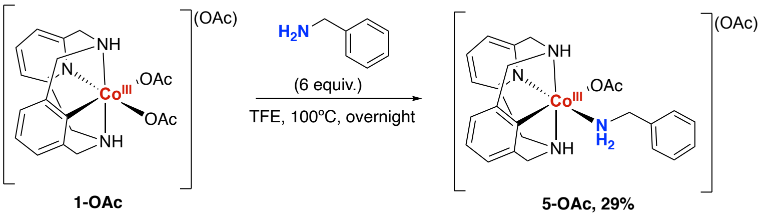

Scheme S13. Formation of **5-OAc** by reacting **1-OAc** complex with benzyl amine.

In a 2 mL vial, **1-OAc** (20 mg, 0.048 mmol) and benzyl amine (6 equiv.) were mixed in TFE and the vial was sealed and heated at 100 ºC. After stirring overnight, the solvent was removed and ether diffusion over $CHCl_3$ give the **5-OAc** organometallic complex, which was characterized by NMR, HRMS and X-Ray spectroscopy.

Mixture of two conformers were observed (6.4 mg, 0.0122 mmol, 26%; 29% NMR yield). **^{1}H NMR** (400 MHz, $CHCl_3$, ppm): 8.35 (bs, 1H), 7.60 (m, 2H), 7.23 (m, 6H), 7.14-7.00 (m, 10H), 6.95 (d, ^3J = 7.7 Hz, 2H), 6.89 (d, ^3J = 7.4 Hz, 2H), 4.82 (m, 3H), 4.70 (d, ^2J = 16.6 Hz, 2H), 4.57 (d, ^{2}J = 16.0 Hz, 2H), 4.33 (bs, 1H), 4.21 (d, ^{2}J = 16.6 Hz, 2H), 3.81-3.77 (m, 4H), 3.71 (d, ^{2}J = 16.9 Hz, 2H), 3.61 (d, ^2J = 16.9 Hz, 2H), 2.89 (bs, 2H), 2.76 (bs, 2H), 2.20 (s, 6H), 1.95 (s, 6H). **^{13}C {1H} NMR** (100 MHz, $CHCl_3$, ppm): δ 207.7, 179.9, 169.0, 168.6, 161.2, 161.1, 161.0, 160.8, 148.0, 147.9, 147.8, 147.7, 138.3, 138.2, 138.1, 138.0, 128.7, 128.6, 128.1, 128.0, 127.8, 127.8, 125.1, 125.0, 121.3, 121.0, 119.2, 119.1, 63.5, 63.4, 63.3, 63.2, 63.1, 63.0, 47.8, 47.4, 47.0, 46.9, 31.2, 25.5. **HRMS** (ESI) calc. for $C_{24}H_{28}CoN_4O_2^+$ [M – OAc]$^+$: 463.1537, found 463.1539. **EA:** $C_{26}H_{31}CoN_4O_2 \cdot 2(H_2O)$: calc. C 55.91 N 10.03 H 6.32, exp. C 55.93 N 10.20 H 6.42%.

*5.3 Reactivity of **1-CH₃CN** with benzyl azide (**a**)*

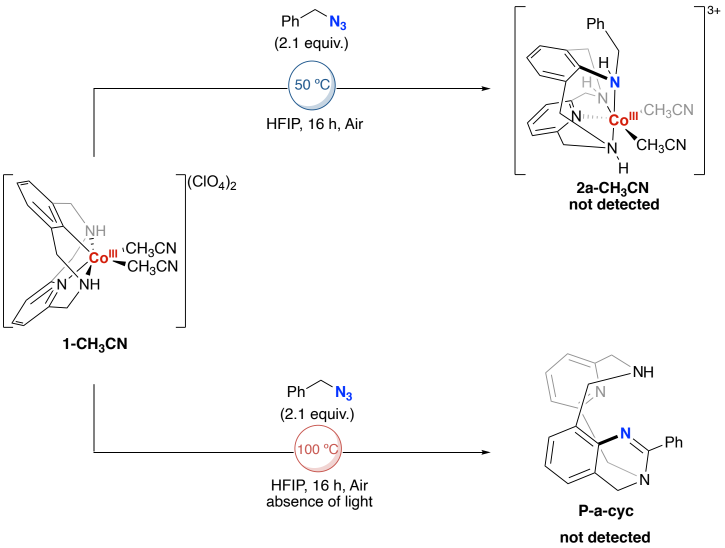

Scheme S14. Unproductive reactivity of **1-CH₃CN** complex with benzyl azide at 50 ºC and 100 ºC.

Procedure A: In a 2 mL vial, **1-CH₃CN** (27.7 mg, 0.048 mmol) and organic azide **a** (2.1 equiv.) were mixed in HFIP (1 mL) and the vial was sealed. The mixture was heated at 50 ºC overnight. The crude was concentrated under vacuum line until the initial volume was reduced to two-thirds. The crude mixture was analyzed by ^{1}H-NMR but no traces of aryl-amine coupling complex **2a-CH₃CN** was observed. The broad ^{1}H-NMR spectrum indicated the presence of unknown paramagnetic species.

Procedure B: In a 2 mL vial, **1-CH₃CN** (27.7 mg, 0.048 mmol) and organic azide **a** (2.1 equiv.) were mixed in HFIP (1 mL) and the vial was sealed and heated at 100ºC during 16 h in the absence of light. Then, after removal of the solvent, the crude mixture was analyzed by ^{1}H-NMR observing the total conversion, however neither the **2a-CH₃CN** nor final **P-a-cyc** were detected. Moreover, 17% of benzaldehyde was observed together with unknown paramagnetic species.

5.4 Kinetic Measurements

A 48 mM solution of **1-OAc** in HFIP was prepared and 0.5 mL of this solution was placed in a UV-vis cuvette. The quartz cell was capped with a septum and placed in the Unisoku cryostat of the UV-vis spectrophotometer and heated at different temperatures. After reaching thermal equilibrium an UV-vis spectrum of the starting complex was recorded. Then, 200 µL of benzyl azide was added.

The value of k were determined at different temperatures and fitted to the Eyring eq 3. to obtain the activation pararmeters $\Delta H^{\ddagger}$ and $\Delta S^{\ddagger}$.

$$\ln(k/T) = 23.75 + \Delta S^{\ddagger}/R - \Delta H^{\ddagger}/RT \qquad (3)$$

$$\Delta G^{\ddagger} = \Delta H^{\ddagger} - T\Delta S^{\ddagger} \qquad (4)$$

Resulting Eyring plot is shown in Figure S20

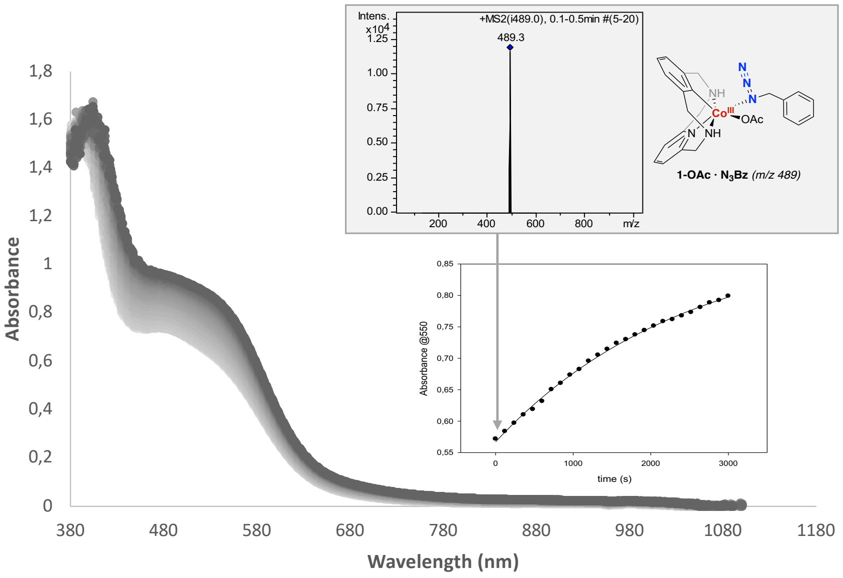

Figure S19. UV-Vis spectral changes of a solution of **1a-OAc** upon reaction with benzyl azide (**a**) in HFIP at 323 K.

T (K)	k (s^{-1})
313,15	1,34E-04
318,15	1,75E-04
320,15	2,66E-04
323,15	4,06E-04
325,15	5,61E-04
328,15	9,76E-04

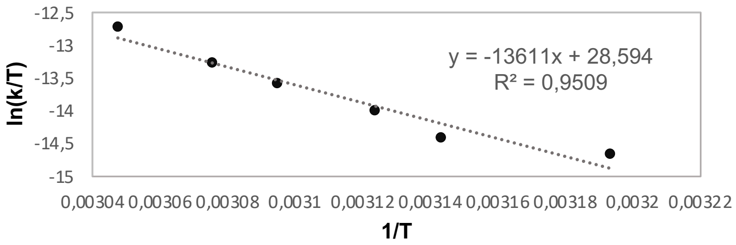

Figure S20. Eyring plot for the reaction between **1a-OAc** and benzyl azide (**a**) in HFIP. Temperature range: from 40 to 55 °C. Activation parameters determined from Erying equation are $\Delta H^{\ddagger} = 27.1$ kcal·mol^{-1}, $\Delta S^{\ddagger} = 9.62 \times 10^{-3}$ kcal·mol^{-1} and $\Delta G^{\ddagger} = 23.94$ kcal·mol^{-1}.

*5.5 Reactivity of **1-OAc** and benzyl azide with xanthene*

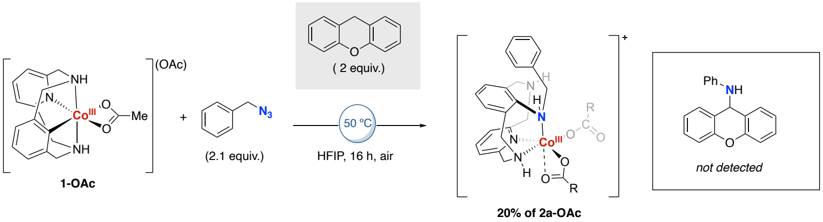

Scheme S15. Reaction of **1-OAc** and benzyl azide (**a**) with xanthene

In a 2 mL vial, **1-OAc** (0.048 mmol), benzyl azide (**a**) (0.1 mmol, 2.1 equiv.) and xanthene (0.096, 2 equiv.) were mixed in HFIP (1 mL). The vial was sealed and stirred under air over 16 h at 50 ºC. The crude mixture was concentrated under vacuum line until the initial volume was reduced to two-thirds. Then, the reaction crude was analyzed by ^{1}H-NMR and HRMS observing the formation of the inserted Co^{III} complex in 20% NMR yield using 1,3,5-trimethoxybenzene as internal standard. The intermolecular imido transfer was not observed using xanthene as an external substrate.

5.6 Evaluation of demetallation sources

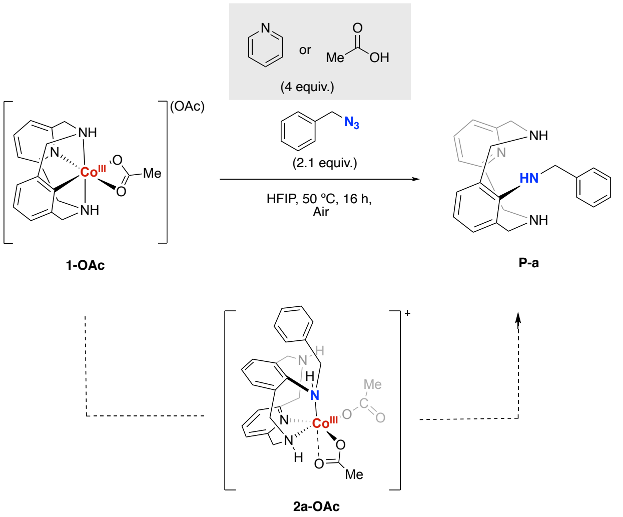

	Yield (%) 2a-OAc	Yield (%) P-a
Standard conditions	46%	n.d.
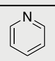	n.d.	n.d.
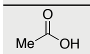	24%	n.d.

Scheme S16. Evaluation of different additive in the **P-a** formation

In a 2 mL vial, **1-OAc** (0.048 mmol), benzyl azide (**a**) (0.1 mmol, 2.1 equiv.) and the corresponding additive (0.192 mmol, 4 equiv.) were mixed in HFIP (1 mL). The vial was sealed and stirred under air over 16 h at 50 ºC. The crude mixture was concentrated under vacuum line until the initial volume was reduced to two-thirds. Then, the reaction crude was analyzed by ^{1}H-NMR and HRMS using 1,3,5-trimethoxybenzene as internal standard.

The presence of pyridine in the reaction quenches the reactivity due to the coordination of the pyridine to the Co^{III} complex starting material. On the other hand, in the presence of acetic acid lower yield of **2a-OAc** (24%) was observed. However, no **P-a** product was detected, and traces amounts of **L-H** ligand were observed in HRMS analysis.

6. Synthesis of aryl-Rh^{III}-X complexes (6_{Me}-OAc)

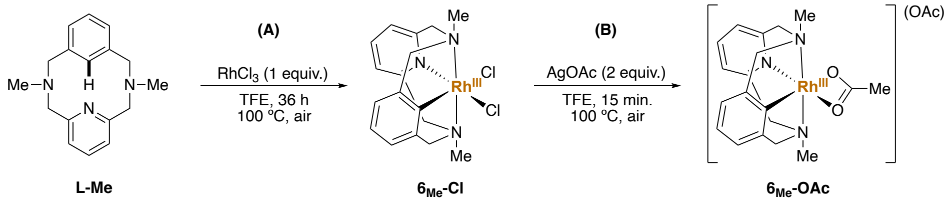

Scheme S17. Synthesis of **6_{Me}-OAc** from **L-Me** ligand.

(A) The **6_{Me}-Cl** complex has been synthesized according to the procedure described in the literature.[2] **L-Me** (100 mg, 0.37 mmol) and $RhCl_3$ (77.4 mg, 0.37 mmol) were mixed in TFE (2.5 mL). The crude mixture was heated up to 100 ºC during 36 h. The solvent was then removed, and the crude mixture was dissolved in a mixture 1:1 CH_3CN/CH_3OH and layered with ether. Yellow crystals of **6_{Me}-Cl** were obtained after 24 h at 4 ºC. The NMR data was in agreement with the previous report.

(B) In a 10 mL vial, **6_{Me}-Cl** (20 mg, 0.045 mmol) and AgOAc (18.4 mg, 0.11 mmol, 2.5 equiv.) were mixed in TFE and heated over 15 min at 100 ºC. Then, the solvent was removed observing a quantitative formation of **6_{Me}-OAc** and was used without purification.

^{1}H NMR (400 MHz, $CHCl_3$, ppm): 7.43 (t, ^3J = 7.6 Hz, 1H), 6.89 (d, ^3J = 7.6 Hz, 2H), 6.72 – 6.67 (m, 3H), 4.95 (d, ^2J = 14.6 Hz, 4H), 3.92 (d, ^2J = 15.7 Hz, 2H), 3.81 (d, ^2J = 14.6 Hz, 2H), 3.12 (s, 6H), 1.95 (s, 3H). **^{13}C {1H} NMR** (100 MHz, $CHCl_3$, ppm): δ 178.3 (1C), 167.3 (1C), 159.2 (2C), 142.2 (2C), 136.9 (1C), 123.0 (1C), 119.7 (2C), 118.4 (1C), 75.4 (2C), 73.8 (2C), 51.7 (2C), 24.6 (1C). **HRMS** (ESI) calc. for $C_{19}H_{23}RhN_3O_2^+$ [M – OAc]$^+$: 428.0840; found: 428.0839.

7. Reactivity of 1_{Me}-OAc (Co^{III}) and 6_{Me}-OAc (Rh^{III}) complex with organic azides

Several attempts to isolate the analogous **6-OAc** (Rh^{III}) using the **L-H** have been unsuccessful. To compare the reactivity of Co and Rh we subject directly de **L-H** ligand and we explored the direct C–H amination reaction using $Co(OAc)_2$ and $Rh(OAc)_3$ under thermal conditions. Using the free **L-H** ligand with $Co(OAc)_2$ the **P-a-cyc** product was observed in 23%. Instead, using $Rh(OAc)_3$, the cyclic aminated **P-a-cyc** product was not observed and only hydrazine was detected as side-product, as depicted in Scheme S18a.

Then, we explored the use of N-methyleted ligand (**L-Me**) and we synthesize the corresponding aryl-M^{III} complexes **1_{Me}-OAc** (Co^{III}) **and 6_{Me}-OAc** (Rh^{III}). No reactivity was observed for the Co^{III} complex using 2 equiv. of benzyl azide in TFE at 50 °C, recovering the starting **1_{Me}-OAc** complex. In contrast, the Rh-based **6_{Me}-OAc** complex led to the formation of **$7a_{Me}$-OAc** complex in 25% yield, featuring an imine moiety directly coordinated to the Rh^{III} center.[27-29] Furthermore, the complex **$7b_{Me}$-OAc** bearing an iminoethyl phenyl was also obtained (35% yield) when an excess of azide **b** (6 equiv.) was used (in TFE, 50 °C).

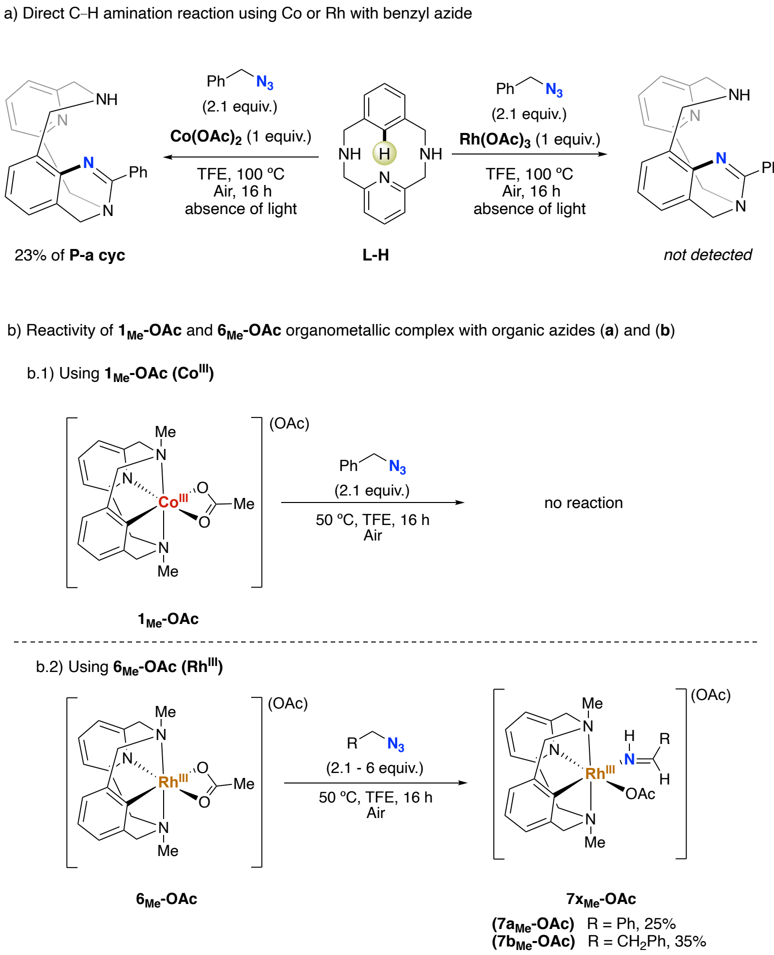

Scheme S18. a) Attempts of direct C–H amination reaction using $Co(OAc)_2$ and $Rh(OAc)_3$ with **L-H** under thermal conditions. b.1) Exploring the reactivity of N-methylated complex with organic azides using **1$_{Me}$-OAc (Co^{III})** and b.2) using **6$_{Me}$-OAc (Rh^{III})** to afford the **7x$_{Me}$-OAc** complexes.

Procedure followed for the formation of 7x$_{Me}$-OAc:

6$_{Me}$-Cl (20 mg, 0.045 mmol) and AgOAc (18.4 mg, 0.11 mmol, 2.5 equiv.) were mixed in TFE and heated over 15 min at 100 ºC. The crude was cooled down to room temperature and filtered and the corresponding organic azide was added. After heating for 16 h at 50 ºC, the crude was concentrated under vacuum line until the initial volume was reduced to two-thirds. The mixture was dissolved in $CHCl_3$ and layered with pentane or ether at 4 ºC giving the corresponding **7x$_{Me}$-OAc** complex which was characterized by NMR and HRMS.

- Aryl-Rh^{III}-(NHCHPh) – (**7a$_{Me}$-OAc**)

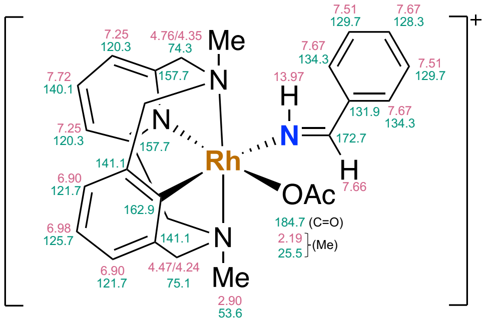

7a$_{Me}$-OAc was synthesized using 2.1 equivalents of azide **a** affording the organometallic complex as yellow oil (5.3 mg, 0.0089 mmol, 20% isolated yield; 25 % NMR Yield). **^{1}H NMR** (400 MHz, $CHCl_3$, ppm): 13.97 (d, ^3J = 21.2 Hz, 1H), 7.72 (t, ^3J = 7.9 Hz, 1H), 6.67 (b, 1H), 7.64-7.58 (m, 3H), 7.51 (m, 2H), 7.25 (d, ^3J = 7.9 Hz, 2H), 6.98 (m, 1H), 6.90 (m, 2H), 4.76 (d, ^2J = 16.0 Hz, 2H), 4.47 (b, 2H), 4.35 (d, ^2J = 16.0 Hz, 2H), 4.24 (d, ^2J = 16.1 Hz, 2H), 2.90 (s, 6H), 2.s9 (s, 3H). **^{13}C {1H} NMR** (100 MHz, $CHCl_3$, ppm): δ 184.6 (1C), 172.7 (1C), 162.9 (1C), 157.7 (2C), 141.1 (2C), 140.1 (1C), 134.3 (2C), 131.9 (1C),

129.7 (2C), 128.3 (1C), 125.7 (1C), 121.7(2C), 120.3 (2C), 75.1 (2C), 74.3 (2C), 53.6 (2C), 25.5 (1C). **HRMS** (ESI) calc. for $C_{26}H_{30}RhN_4O_2^+$ [M – OAc]$^+$: 533.1418, found 533.1424. **EA:** $C_{28}H_{33}RhN_4O_2 \cdot 3.1(H_2O) \cdot 0.5(CHCl_3)$ calc. C 48.35 N 7.91 H 5.65%; exp. C 48.16 N 8.11 H 5.43%.

- Aryl-Rh(III)-(NHCHCH$_2$Ph) – (**7b$_{Me}$-OAc**)

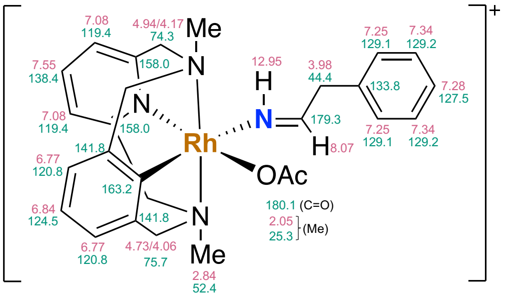

7b$_{Me}$-OAc was synthesized using 6 equivalents of azide **b** affording the organometallic complex as yellow oil (4.0 mg, 0.0066 mmol, 15%; 35 % NMR Yield). **^{1}H NMR** (400 MHz, CHCl$_3$, ppm): 12.95 (d, ^3J = 20.3 Hz, 1H), 8.07 (dt, ^3J = 20.3 Hz, ^3J = 5.6 Hz, 1H), 7.55 (t, ^3J = 7.9 Hz, 1H), 7.34 (m, 2H), 7.28 (m, 1H), 7.25 (m, 2H), 7.08 (d, ^3J = 7.9 Hz, 2H), 6.84 (m, 1H), 6.77 (m, 2H), 4.94 (d, ^2J = 15.6 Hz, 2H), 4.73 (d, ^2J = 15.6 Hz, 2H), 4.17 (d, ^2J = 15.6 Hz, 2H), 4.06 (d, ^2J = 15.6 Hz, 2H), 3.98 (d, d, ^3J = 5.6 Hz, 2H), 2.84 (s, 6H), 2.05 (s, 3H). **^{13}C {1H} NMR** (100 MHz, CHCl$_3$, ppm): δ 180.1 (1C), 179.3 (1C), 163.2 (1C), 158.0 (2C), 141.8 (2C), 138.4 (1C), 133.8 (1C), 129.2 (2C), 129.1 (2C), 124.5 (1C), 120.8 (2C), 119.4 (2C), 75.7 (2C), 74.3 (2C), 52.4 (2C), 44.4 (1C), 25.3 (1C). **HRMS** (ESI) calc. for $C_{27}H_{32}RhN_4O_2^+$ [M – OAc]$^+$: 547.1575, found 547.1567. **EA:** $C_{29}H_{35}RhN_4O \cdot 0.75(H_2O) \cdot 2(CHCl_3)$: calc. C 43.36 N 6.52 H 4.53%, exp. C 43.19 N 6.56 H 4.48%.

8. XAS analysis of 1-OAc, 4b-OAc and 5-OAc complexes

1-OAc and **5-OAc** exhibit rising edges centered around 7720 eV at half-height. Together with the intense pre-edge region with a maximum at 7711.5 eV this is consistent with the previously reported aryl-Co^{III} species having Co-C bonds.[1-2] The slightly lower rising edge energy and pre-edge intensity of **5-OAc** relative to **1-OAc** indicates a more negative effective charge on the metal center and a less covalent interaction with the surrounding ligands. EXAFS analysis shows both species consist of a six-coordinate environment having 2 N/O/C scattering atoms at 1.86 A and 3 N/O scattering atoms centered around 2.0 A, consistent with the reported crystal structures (Fig. S21).[1-2] Therefore the differences in the XANES features must arise from the differences in surrounding ligands, where the pi-accepting nature of the acetonitrile ligands, evidenced in the crystal structure of **1-OAc**[1] result in an ever so slightly effectively more oxidized metal center, leading to a slight increase in the rising edge energy. Furthermore the pi-backbonding in **1-OAc** is also expected to increase metal p-character mixing into the Co d-manifold resulting in a slightly more intense pre-edge feature with respect to **5-OAc**. **4b-OAc** on the other hand has a similar rising edge profile to the previously reported Co^{II} species from the same family of complexes.[1] Coupled with EXAFS analysis of the bond distances, which are significantly larger than for the +3 analogues with 2 N/O scattering atoms at 2.01 A and 4 N/O scattering atoms at 2.14 A, all lead to characterizing this species as having a +2 metal oxidation state. Lastly, the small scattering intensity in the Fourier transformed EXAFS spectra above 2 A, and the need of only including 2 C scattering atoms at 2.95 A for **4b-OAc** suggests, in stark contrast to the Co^{III} analogues, a more disordered metal coordination environment in the bulk sample as opposed to what might be expected from the crystal structure.

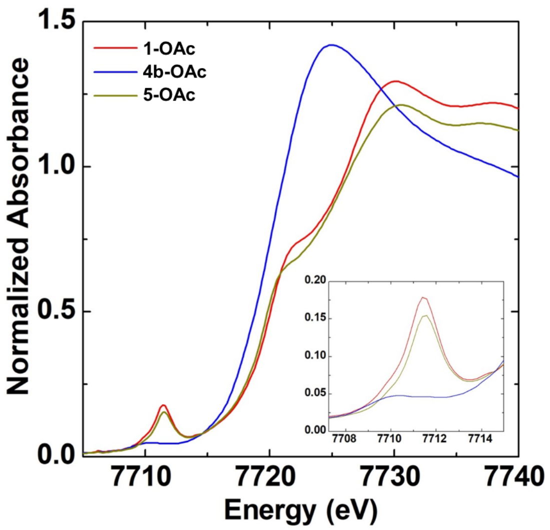

Figure S21: XANES region XAS spectra highlighting the rising edges for **1-OAc**, **4b-OAc** and **5-OAc**, with inset showing the pre-edge region due to 1s-3d transitions.

Table S2: X-ray absorption spectroscopy parameters at the Co K-edge.

SAMPLE	OXIDATION STATE	E_0' (eV)[1]	$E_0^{1/2}$ (eV)[2]	Pre-edge Energy (eV)	Pre-edge Area
3c-OAc[3]	2.0	7719.8	7718.5	7709.8	0.12
1-OAc	3.0	7720.1	7720.0	7711.5	0.30
4b-OAc	2.0	7719.9	7718.8	7710.1	0.11
5-OAc	3.0	7719.6	7719.8	7711.5	0.27

(1) E0' values at were taken as the maximum infection point of the first derivative

(2) $E0_{1/2}$ values were taken at the half-height ie. 0.5 normalized intensity units of the rising edge

(3) J. Am. Chem. Soc. 2016, 138, 14388–14397

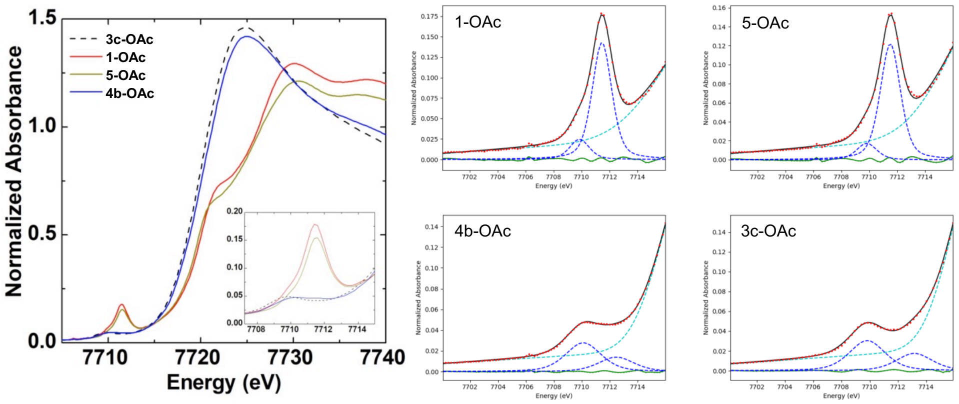

Figure S22: Co K-edge XANES spectra and pre-edge fits.

Panel S1:Summary of EXAFS analysis at the Co^{III}complex 1-OAc: k^3-weighted fits carried out in r-space over a k-range of 3-12 Å using a Hanning window (dk 1), and an $S_0 = 0.9$ was chosen. Bond distances and disorder parameters (Δr_{eff} and σ^2) were allowed to float having initial values of 0.0 Å and 0.003 Å^2 respectively, with a universal E_0 and $\Delta E_0 = 0$ eV. Plotted is the best fit (bottom of the table), σ^2 values reported as x 10^3 Å^2.

							M-N/C/O			M-N/C/O			M-C			M--C-N--M			M--C-N^{MeCN}--M		
SAMPLE	Δk	Δr	*Var.*	R_{FACTOR}	χ^2_v	ΔE_0	N	r(Å)	σ^2	N	r(Å)	σ^2	N	r(Å)	σ^2	N	r(Å)	σ^2	N	r(Å)	σ^2
	2-12	1-3	5	0.0247	116.8	2.2	2	1.88	5	3	1.99	5	6	2.81	4	8	2.99	4	3	3.15	5
	2-12	1-3	5	0.0248	116.6	2.3	1	1.86	6	4	1.97	6	6	2.81	4	8	2.99	4	3	3.16	6
	2-12	1-3	5	0.0139	65.4	2.0	1	1.86	8	5	1.97	8	6	2.81	4	8	2.99	4	3	3.15	8
1-OAc	2-12	1-3	5	0.0174	82.2	2.2	3	1.90	6	3	2.01	6	6	2.81	4	8	2.99	4	3	3.16	6
	2-12	1-3	5	0.0254	119.8	1.8	2	1.87	7	4	1.98	7	6	2.81	4	-	-	-	3	3.15	7
	2-12	1-3	5	0.0246	116.3	3.6	2	1.88	7	4	1.99	7	6	2.82	4	8	3.00	4	-	-	-
	2-12	1-3	5	0.0127	59.6	1.8	2	1.87	7	4	1.98	7	6	2.81	4	8	2.99	4	3	3.16	7

*Although EXAFS can not differentiate between O/N/C scattering paths, chemical intuition was used for description;
M is the absorber element in this case Mn

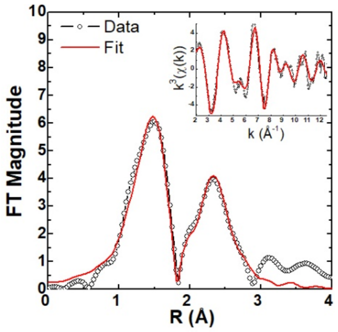

Panel S2: Summary of EXAFS analysis of 4b-OAc: k^3-weighted fits carried out in r-space over a k-range of 3-11.5 Å using a Hanning window (dk 1), and an $S_0 = 0.9$ was chosen. Bond distances and disorder parameters (Δr_{eff} and σ^2) were allowed to float having initial values of 0.0 Å and 0.003 Å^2 respectively, with a universal E_0 and $\Delta E_0 = 0$ eV. Plotted is the best fit (bottom of the table), σ^2 values reported as x 10^3 Å^2.

							M-N/C/O			M-N/C/O			M-N/C/O			M-C		
SAMPLE	Δk	Δr	*Var.*	R_{FACTOR}	χ^2_v	ΔE_0	N	r(Å)	σ^2	N	r(Å)	σ^2	N	r(Å)	σ^2	N	r(Å)	σ^2
	2-11.5	1-3	3	0.031	139.5	1.6	1	2.03	7	4	2.13	7	-	-	-	2		7
	2-11.5	1-3	3	0.028	124.6	1.9	2	2.05	7	3	2.15	7	-	-	-	2	2.97	7
4b-OAc	2-11.5	1-3	3	0.018	82.3	1.7	4	2.07	9	2	2.17	9	-	-	-	2	2.99	9
	2-11.5	1-3	3	0.03	135.0	1.4	2	2.02	5	2	2.13	5	2	2.21	5	-	-	-
	2-11.5	1-3	3	0.018	79.2	1.6	2	2.02	5	2	2.13	5	2	2.21	5	2	2.94	5
	2-11.5	1-3	3	0.0140	62.5	1.3	2	2.04	8	4	2.14	8	-	-	-	2	2.96	8

*Although EXAFS can not differentiate between O/N/C scattering paths, chemical intuition was used for description;
M is the absorber element in this case Mn

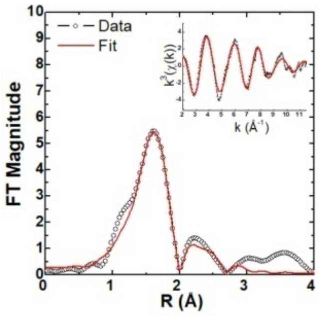

Panel S3: Summary of EXAFS analysis at the 5-OAc: k^3-weighted fits carried out in r-space over a k-range of 3-12 Å using a Hanning window (dk 1), and an $S_0 = 0.9$ was chosen. Bond distances and disorder parameters (Δr_{eff} and σ^2) were allowed to float having initial values of 0.0 Å and 0.003 Å^2 respectively, with a universal E_0 and $\Delta E_0 = 0$ eV. Plotted is the best fit (bottom of the table), σ^2 values reported as x 10^3 Å^2.

							M-N/C/O			M-N/C/O			M-C			M-C			M--C-N--M			M--C-O/N--M		
SAMPLE	Δk	Δr	*Var.*	R_{FACTOR}	χ^2_v	ΔE_0	N	r(Å)	σ^2	N	r(Å)	σ^2	N	r(Å)	σ^2	N	r(Å)	σ^2	N	r(Å)	σ^2	N	r(Å)	σ^2
5-OAc	2-12	1-3	4	0.038	225.7	-1.2	2	1.87	2	3	2.01	2	4	2.81	2	2	2.94	2	4	3.01	2	4	3.16	2
	2-12	1-3	4	0.036	212.6	0.0	1	1.87	2	4	2.01	6	4	2.8	2	2	2.94	2	4	3.00	2	4	3.16	2
	2-12	1-3	4	0.036	214.5	-1.1	2	1.87	3	4	2.01	4	4	2.81	3	2	2.94	3	-	-	-	-	-	-
	2-12	1-3	4	0.039	235.7	-0.1	2	1.87	3	4	2.01	4	4	2.81	3	2	2.94	3	8	3.01	3	-	-	-
	2-12	1-3	4	0.054	322.0	-1.7	2	1.87	5	4	2.02	5	6	2.81	5	-	-	-	4	3.01	5	4	3.16	5
	2-12	1-3	4	0.026	152.0	-1.4	2	1.86	3	4	2.01	4	4	2.8	3	2	2.94	3	4	3.00	3	4	3.16	3

*Although EXAFS can not differentiate between O/N/C scattering paths, chemical intuition was used for description;
M is the absorber element in this case Mn

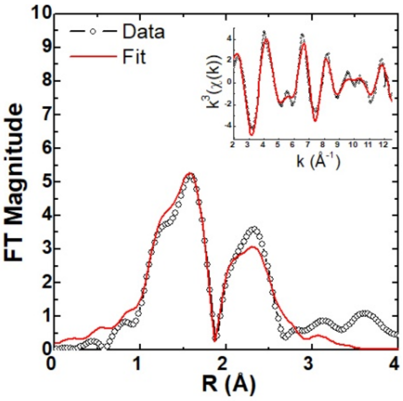

9. Crystallographic data information

*9.1 X-Ray structure of **1-OBzOMe***

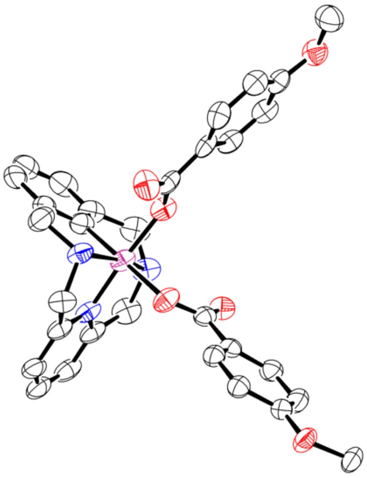

Figure S23. X-Ray crystal structure of **1-OBzOMe** at 50% probability level. H-atoms and solvent molecules have been omitted for clarity.

Orange-pink needle of $(C_{31}H_{28}CoN_3O_6)_2$, $7H_20$, were grown from a $CHCl_3$ solution layered with pentane, and used for low temperature (100(2) K) X-ray structure determination. The X-ray intensity data were measured on a Bruker D8 QUEST ECO system equipped with a doubly curved silicon crystal Bruker Triumph monochromator and a Mo Kalpha sealed X-ray tube (λ = 0.71073 Å). A total of 540 frames were collected. The total exposure time was 12.50 hours. The frames were integrated with the Bruker SAINT software package using a narrow-frame algorithm. The integration of the data using a monoclinic unit cell yielded a total of 43919 reflections to a maximum θ angle of 27.54° (0.77 Å resolution), of which 6956 were independent (average redundancy 6.314, completeness = 99.5%, R_{int} = 10.94 %, R_{sig} = 7.97%) and 4543 (65.31%) were greater than $2\sigma(F^2)$. The final cell constants of a = 12.9753(10) Å, b = 14.8245(14) Å, c = 16.6220(14) Å, β = 108.367(2)°,volume = 3034.4(5) Å^3, are based upon the refinement of the XYZ centroids of 9909 reflections above 20 σ(I) with 5.496° < 2θ < 54.82°.Data were corrected for absorption effects using the MultiScan method (SADABS). The ratio of minimum to maxim um apparent transmission was 0.643. The calculated minimum and maximum transmission coefficients (based on crystal size) are 0.8340 and 0.9810. The structure was solved and refined using the Bruker SHELXTL Software Package, using the space group P 1 21/n 1, with Z = 2 for the formula unit, $C_{62}H_{70}Co_2N_6O_{19}$. The final anisotropic full-matrix least-squares refinement on F^2 with 433 variables converged at R1 = 5.85%, for the observed data and wR2 = 16.10% for all data. The goodness-of-fit was 1.049. The largest peak in the final differences electron density synthesis was 0.606 $e^-/Å^3$ and the largest hole was -0.694 $e^-/Å^3$ with an RMS deviation of 0.087 $e^-/Å^3$. On the basis of the final model, the calculated density was 1.446 g/cm^3 and F(000), 1380 e^-.

Table S3. Crystallographic parameters for **1-OBzOMe**.

**Chemical formula**	$(C_{31}H_{28}CoN_3O_6)_2$, $7H_20$	
**Formula weight**	1321.10 g/mol	
**Temperature**	100(2) K	
**Wavelenght**	0.71073 Å	
**Crystal size**	0.030 x 0.040 x 0.300 mm	
**Crystal system**	Monoclinic	
**Space group**	P 1 21/n 1	
**Unit cell dimensions**	a = 12.9753(10) Å	α = 90°
	b = 14.8245(14) Å	β = 108.367(2)°
	c = 16.6220(14) Å	γ = 90°
**Volume**	3034.4(5) Å^3	
**Density (calculated)**	1.446 g/cm^3	
**Absorption coefficient**	0.627 mm^{-1}	
**Final R indices**	4543 data; I>2σ(I)	R1 = 0.0585, wR2 = 0.1363
	all data	R1 = 0.1062, wR2 = 0.1610

*9.2 X-Ray structure of **1-OBzCF₃***

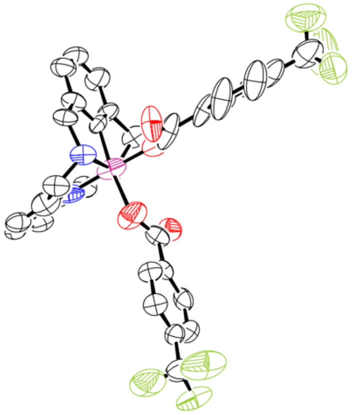

Figure S24. X-Ray crystal structure of **1-OBzCF$_3$** at 50% probability level. H-atoms and solvent molecules have been omitted for clarity.

Red-pink block of $C_{31}H_{24}CoF_6N_3O_4$, $(C_8H_5F_3O_2)$, H_2O were grown from a $CHCl_3$ solution layered with pentane, and used for low temperature (100(2) K) X-ray structure determination. The X-ray intensity data were measured on a Bruker D8 QUEST ECO system equipped with a doubly curved silicon crystal Bruker Triumph monochromator and a Mo Kalpha sealed X-ray tube (λ = 0.71076 Å). A total of 1452 frames were collected. The total exposure time was 21.65 hours. The frames were integrated with the Bruker SAINT software package using a narrow-frame algorithm. The integration of the data using a monoclinic unit cell yielded a total of 84160 reflections to a maximum θ angle of 28.37° (0.75 Å resolution), of which 9346 were independent (average redundancy 9.005, completeness = 99.5%, R_{int} = 3.16 %, R_{sig} = 1.85%) and 7955 (85.12%) were greater than $2\sigma(F^2)$. The final cell constants of a = 14.30(2) Å, b = 14.84(2) Å, c = 17.70(3) Å, β = 93.35(3)°,volume = 3750.(10) Å^3, are based upon the refinement of the XYZ centroids of 9921 reflections above 20 σ(I) with 5.945° < 2θ < 56.39°.Data were corrected for absorption effects using the MultiScan method (SADABS). The ratio of minimum to maxim um apparent transmission was 0.921. The calculated minimum and maximum transmission coefficients (based on crystal size) are 0.9210 and 0.9460. The structure was solved and refined using the Bruker SHELXTL Software Package, using the space group P 1 21/c 1, with Z = 4 for the formula unit, $C_{39}H_{31}CoF_9N_3O_7$. The final anisotropic full-matrix least-squares refinement on F^2 with 813 variables converged at R1 = 7.33%, for the observed data and wR2 = 17.75% for all data. The goodness-of-fit was 1.092. The largest peak in the final differences electron density synthesis was 0.685 e$^-$/Å^3 and the largest hole was -1.074 e$^-$/Å^3 with an RMS deviation of 0.066 e$^-$/Å^3. On the basis of the final model, the calculated density was 1.565 g/cm^3 and F(000), 1800 e$^-$.

Table S4. Crystallographic parameters for **1-OBzCF$_3$**.

**Chemical formula**	$C_{31}H_{24}CoF_6N_3O_4$, $(C_8H_5F_3)$, H_2O	
**Formula weight**	883.60 g/mol	
**Temperature**	100(2) K	
**Wavelenght**	0.71076 Å	
**Crystal size**	0.100 x 0.100 x 0.150 mm	
**Crystal system**	Monoclinic	
**Space group**	P 1 21/c 1	
**Unit cell dimensions**	a = 14.30(2) Å	α = 90°
	b = 14.84(2) Å	β = 93.35(3)°
	c = 17.70(3) Å	γ = 90°
**Volume**	3750.(10) Å^3	
**Density (calculated)**	1.565 g/cm^3	
**Absorption coefficient**	0.558 mm^{-1}	
**Final R indices**	7955 data; I>2σ(I)	R1 = 0.0733, wR2 = 0.1694
	all data	R1 = 0.0850, wR2 = 0.1775

*9.3 X-Ray structure of **3a-OAc***

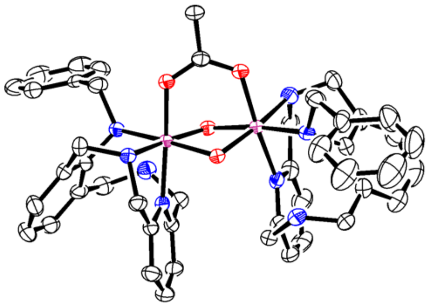

Figure S25. X-Ray crystal structure of **3a-OAc** at 50% probability level. H-atoms and solvent molecules have been omitted for clarity.

Needle-like of $C_{46}H_{53}Co_2N_8O_4$, $(C_3H_2F_6O)_3$, $(C_2H_3O_2)_4$ were grown from slow $CHCl_3$ solution layered with pentane at -4 °C , and used for low temperature (100(2) K) X-ray structure determination. The X-ray intensity data were measured on a Bruker D8 QUEST ECO system equipped with a doubly curved silicon crystal Bruker Triumph monochromator and a Mo Kalpha sealed X-ray tube (λ = 0.71076 Å). A total of 277 frames were collected. The total exposure time was 9.23 hours. The frames were integrated with the Bruker SAINT software package using a narrow-frame algorithm. The integration of the data using a monoclinic unit cell yielded a total of 39050 reflections to a maximum θ angle of 18.79° (1.10 Å resolution), of which 5512 were independent (average redundancy 7.085, completeness = 99.5%, R_{int} = 36.10 %, R_{sig} = 20.13%) and 3026 (54.90%) were greater than $2\sigma(F^2)$. The final cell constants of a = 21.20(4) Å, b = 11.96(2) Å, c = 29.59(4) Å, β = 108.90(4)°,volume = 7098.(19) $Å^3$, are based upon the refinement of the XYZ centroids of 1009 reflections above 20 σ(I) with 6.502° < 2θ < 37.65°.Data were corrected for absorption effects using the MultiScan method (SADABS). The ratio of minimum to maxim um apparent transmission was 0.727. The calculated minimum and maximum transmission coefficients (based on crystal size) are 0.9180 and 0.9880. The structure was solved and refined using the Bruker SHELXTL Software Package, using the space group P 1 21/c 1, with Z = 4 for the formula unit, $C_{63}H_{71}Cl_0Co_2F_{18}N_8O_{15}$. A considerable amount of electron density attributable to three $C_3H_2F_6O$ and two $C_2H_3O_2$ heavyly disordered solvent molecules was removed with the SQUEEZE option of PLATON.[30] Those solvent molecules are, however, included in the reported chemical formula and derived values (e.g. formula weight, F(000), etc). The final anisotropic full-matrix least-squares refinement on F^2 with 406 variables converged at R1 = 8.84%, for the observed data and wR2 = 24.85% for all data. The goodness-of-fit was 1.024. The largest peak in the final differences electron density synthesis was 0.41 $e^-/Å^3$ and the largest hole was -0.46 $e^-/Å^3$ with an RMS deviation of 0.231 $e^-/Å^3$. On the basis of the final model, the calculated density was 1.534 g/cm^3 and F(000), 2132 e^-.

Table S5. Crystallographic parameters for **3a-OAc**.

**Chemical formula**	$C_{46}H_{53}Co_2N_8O_4$, $(C_3H_2F_6O)_3$, $(C_2H_3O_2)_4$	
**Formula weight**	1640.13 g/mol	
**Temperature**	100(2) K	
**Wavelenght**	0.71076 Å	
**Crystal size**	0.020 x 0.020 x 0.150 mm	
**Crystal system**	Monoclinic	
**Space group**	P 1 21/c 1	
**Unit cell dimensions**	a = 21.20(4) Å	α = 90°
	b = 11.96(2) Å	β = 108.90(4)°
	c = 29.59(4) Å	γ = 90°
**Volume**	7098.(19) $Å^3$	
**Density (calculated)**	1.534 g/cm^3	
**Absorption coefficient**	0.583 mm^{-1}	
**Final R indices**	3026 data; I>2σ(I)	R1 = 0.0884, wR2 = 0.2073
	all data	R1 = 0.1530 wR2 = 0.2485

9.4 X-Ray structure of ***4b-OAc***

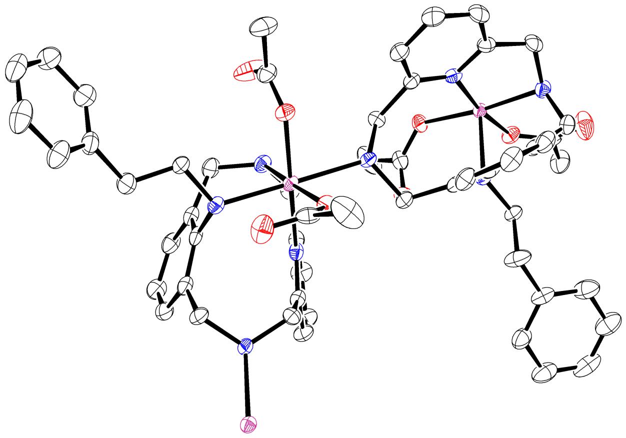

Figure S26. X-Ray crystal structure of **4b-OAc** at 50% probability level. H-atoms and solvent molecules have been omitted for clarity.

Pink needle of $(C_{27}H_{32}CoN_4O_4)_2$, $(CH_2Cl_2)_2$, H_2O were grown from pentane diffusion in a concentrated solution in CH_2Cl_2 anhydrous under inert atmosphere and used for low temperature (100(2) K) X-ray structure determination. The X-ray intensity data were measured on a Bruker D8 QUEST ECO system equipped with a doubly curved silicon crystal Bruker Triumph monochromator and a Mo Kalpha sealed X-ray tube (λ = 0.71073 Å). A total of 910 frames were collected. The total exposure time was 22.75 hours. The frames were integrated with the Bruker SAINT software package using a narrow-frame algorithm. The integration of the data using a monoclinic unit cell yielded a total of 76818 reflections to a maximum θ angle of 27.55° (0.77 Å resolution), of which 6608 were independent (average redundancy 11.625, completeness = 99.7%, R_{int} = 4.94%, R_{sig} = 2.34%) and 5442 (82.35%) were greater than $2\sigma(F^2)$. The final cell constants of a = 14.830(12) Å, b = 11.908(10) Å, c = 16.675(12) Å, β = 103.06(2)°,volume = 2869.(4) Å^3, are based upon the refinement of the XYZ centroids of 9796 reflections above 20 σ(I) with 6.068° < 2θ < 54.93°.Data were corrected for absorption effects using the MultiScan method (SADABS). The ratio of minimum to maxim um apparent transmission was 0.880. The calculated minimum and maximum transmission coefficients (based on crystal size) are 0.7890 and 0.9140. The structure was solved and refined using the Bruker SHELXTL Software Package, using the space group P 1 21/n 1, with Z = 2 for the formula unit, $C_{56}H_{70}Cl_4Co_2N_8O_9$. The final anisotropic full-matrix least-squares refinement on F^2 with 375 variables converged at R1 = 7.78%, for the observed data and wR2 = 24.18% for all data. The goodness-of-fit was 1.087. The largest peak in the final differences electron density synthesis was 2.371 e$^-$/Å^3 and the largest hole was -2.035 e$^-$/Å^3 with an RMS deviation of 0.167 e$^-$/Å^3. On the basis of the final model, the calculated density was 1.457 g/cm^3 and F(000), 1312 e$^-$.

Table S6. Crystallographic parameters for **4b-OAc.**

**Chemical formula**	$(C_{27}H_{32}CoN_4O_4)_2$, $(CH_2Cl_2)_2$, H_2O	
**Formula weight**	1258.86 g/mol	
**Temperature**	100(2) K	
**Wavelenght**	0.71073 Å	
**Crystal size**	0.110 x 0.110 x 0.300 mm	
**Crystal system**	Monoclinic	
**Space group**	P 1 21/n 1	
**Unit cell dimensions**	a = 14.830(12) Å	α = 90°
	b = 11.908(10) Å	β = 103.06(2)°
	c = 16.675(12) Å	γ = 90°
**Volume**	2869.(4) Å^3	
**Density (calculated)**	1.457 g/cm^3	
**Absorption coefficient**	0.828 mm^{-1}	
**Final R indices**	5442 data; I>2σ(I)	R1 = 0.0778, wR2 = 0.2251
	all data	R1 = 0.0932, wR2 = 0.2418

*9.5 X-Ray structure of **5-OAc***

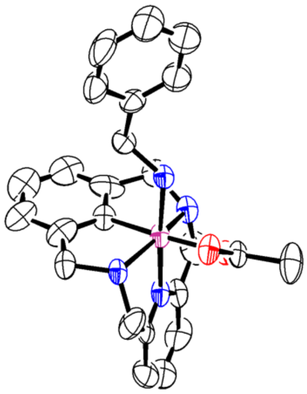

Figure S27. X-Ray crystal structure of **5-OAc** at 50% probability level. H-atoms and solvent molecules have been omitted for clarity.

Pink-red prism-like specimen of $(C_{24}H_{28}CoN_4O_4)_2$, $(CH_2Cl_2)_2$, $(C_2H_3O_2)_2$, $(H_2O)_3$ were grown from slow CH_2Cl_2 solution layered with pentane and used for low temperature (100(2) K) X-ray structure determination. The X-ray intensity data were measured on a Bruker D8 QUEST ECO system equipped with a doubly curved silicon crystal Bruker Triumph monochromator and a Mo Kalpha sealed X-ray tube (λ = 0.71073 Å). A total of 707 frames were collected. The total exposure time was 19.64 hours. The frames were integrated with the Bruker SAINT software package using a narrow-frame algorithm. The integration of the data using a triclinic unit cell yielded a total of 113544 reflections to a maximum θ angle of 26.48° (0.80 Å resolution), of which 12127 were independent (average redundancy 9.363, completeness = 99.6%, R_{int} = 9.33%, R_{sig} = 5.37%) and 8266 (68.16%) were greater than $2\sigma(F^2)$. The final cell constants of a = 11.4024(5) Å, b = 13.8632(6) Å, c = 18.7203(8) Å, α = 87.4460(10)°, β = 86.1440(10)°, γ = 84.8130(10)°, volume = 2938.2(2) Å^3, are based upon the refinement of the XYZ centroids of 9994 reflections above 20 σ(I) with 5.906° < 2θ < 52.70°.
Data were corrected for absorption effects using the MultiScan method (SADABS). The ratio of minimum to maximum appar ent transmission was 0.906. The calculated minimum and maximum transmission coefficients (based on crystal size) are 0.8140 and 0.9570. The structure was solved and refined using the Bruker SHELXTL Software Package, using the space group P -1, with Z = 2 for the formula unit, $C_{54}H_{70}Cl_6Co_2N_8O_{11}$. The final anisotropic full-matrix least-squares refinement on F^2 with 706 variables converged at R1 = 9.61%, for the observed data and wR2 = 26.67% for all data. The goodness-of-fit was 1.028. The largest peak in the final differences electron density synthesis was 1.684 e$^-$/Å^3 and the largest hole was -1.319 e$^-$/Å^3 with an RMS deviation of 0.137 e$^-$/Å^3. On the basis of the final model, the calculated density was 1.512 g/cm^3 and F(000), 1388 e$^-$.

Table S7. Crystallographic parameters for **5-OAc.**

**Chemical formula**	$(C_{24}H_{28}CoN_4O_4)_2$, $(CH_2Cl_2)_2$, $(C_2H_3O_2)_2$, $(H_2O)_3$	
**Formula weight**	1337.74 g/mol	
**Temperature**	100(2) K	
**Wavelenght**	0.71073 Å	
**Crystal size**	0.050 x 0.100 x 0.240 mm	
**Crystal system**	Monoclinic	
**Space group**	P -1	
**Unit cell dimensions**	a = 11.4024(5) Å	α = 87.4460(10)°
	b = 13.8632(6) Å	β = 86.1440(10)°
	c = 18.7203(8) Å	γ = 84.8130(10)°
**Volume**	2938.2(2) Å^3	
**Density (calculated)**	1.512 g/cm^3	
**Absorption coefficient**	0.896 mm^{-1}	
**Final R indices**	8266 data; I>2σ(I)	R1 = 0.0961, wR2 = 0.2308
	all data	R1 = 0.1408, wR2 = 0.2667

10. Computational studies

10.1 EOS (Effective oxidation state) analysis

Nitrene (INT-N)

The occupation of the EFOs showed in Figure S28 and in the EOS analysis presented in Table S8 clearly stablish that **INT-N** can be described as an aryl-Co^{III}=N-R (R = -CH_2Ph) nitrene species (Fischer-type). The low R(%) value of the OS assignation is due to highly covalent character of the Co-aryl bond.

Table S8. For the nitrene (**INT-N**), effective oxidation state of each fragment, as well as occupations of the last occupied and first unoccupied EFOs.

Fragment	EOS	Last occ.	First unocc.
1 (Co)	3	0.618	0.483
2 (N_2)	0	0.969	0.007
3 (Carboxylate)	-1	0.858	0.017
4 ($PhCH_2N$)	0	0.655	0.343
5 (Aryl)	-1	0.485	0.063

Reliability Index R(%) = 50.2%

Masked nitrene (INT-MaskN)

The results of the EOS analysis indicating that **INT-MaskN** can be describe as a Co^{III} nitrene:

Table S9. For the masked nitrene (**INT-MaskN**), effective oxidation state of each fragment, as well as occupations of the last occupied and first unoccupied EFOs.

Fragment	EOS	Last occ.	First unocc.
1 (Co)	3	0.817	0.419
2 (Ring)	-1	0.741	0.045
3 (Aryl)	-1	0.542	0.095

Reliability Index R(%) = 62.21%

10.2. Analysis of the EFOs (Effective fragment orbitals)

For the nitrene (**INT-N**), a pair of EFOs can be found corresponding to the Co-N π bond. These two EFOs (one for the Co, and one on the N atom in the $PhCH_2N$ fragment) are obtained separately and independently. The former has an occupation of 0.62, and the latter an occupation of 0.34, so they agree with the description of **INT-N** as an aryl-Co^{III}=N-R nitrene with a significant back-donation from the Co to the N.

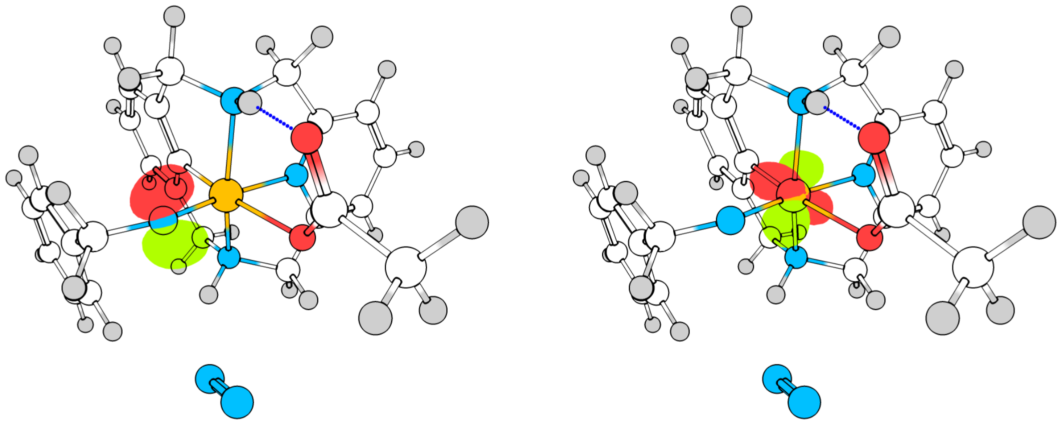

$PhCH_2N$ EFO (occupation = 0.34) *Co EFO (occupation = 0.62)*

Figure S28. The complementary EFOs of Co and N corresponding to the Co-N π bond.

A pair of EFOs describing the sigma bond can also be identified. The Co has an occupation of 0.40, with the N having an occupation of 0.66:

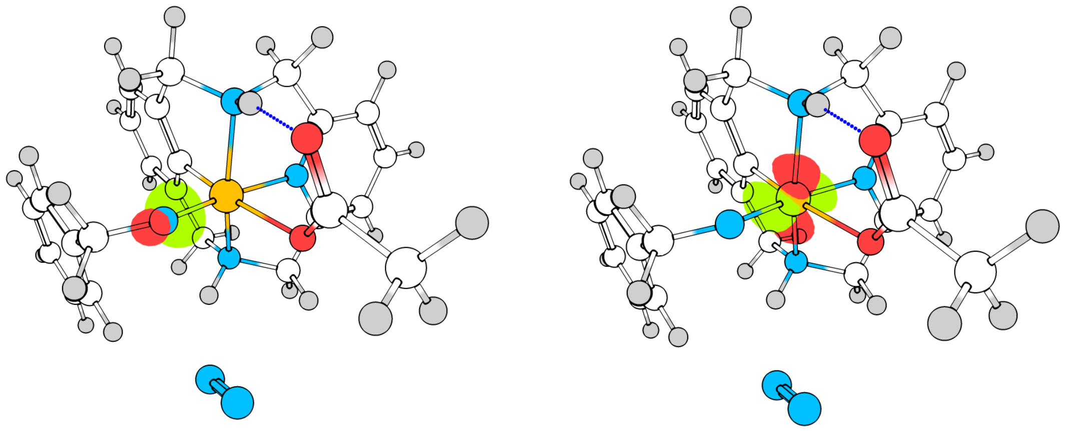

$PhCH_2N$ EFO (occupation = 0.66) *Co EFO (occupation = 0.40)*

Figure S29. The complementary EFOs of Co and N corresponding to the Co-N sigma bond.

For the masked nitrene (**INT-MaskN**), the sigma Co-N bond can easily be identified, with an occupation of 0.31 for the Co, and 0.74 for the N, which agrees with the description of **INT-MaskN** as a masked Co^{III} nitrene:

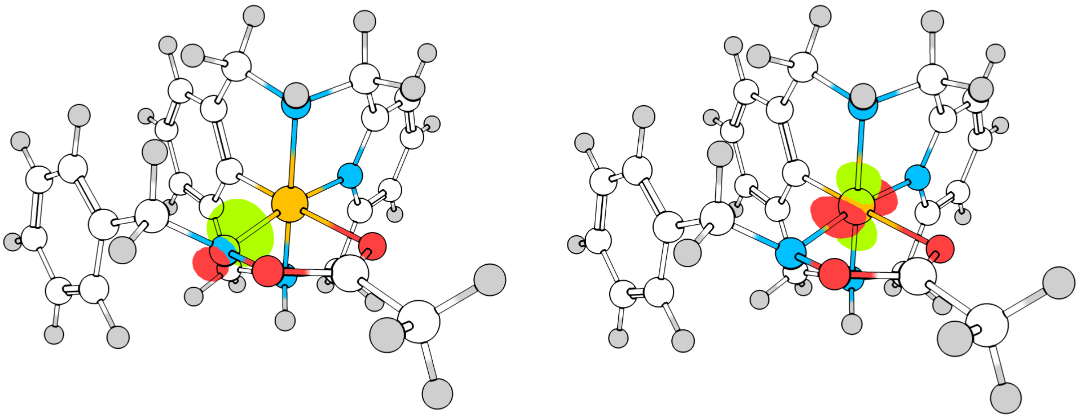

$PhCH_2N$ EFO (occupation = 0.31) *Co EFO (occupation = 0.74)*

Figure S30. The complementary EFOs of Co and N corresponding to the Co-N sigma bond.

In this case there's no π bond (the bond order is only 0.51), but we can find the lone pair on the nitrogen, with an occupation of 0.96:

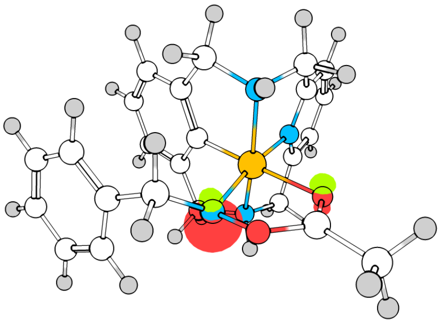

Occupation = 0.96

Figure S31. EFO of the ring fragment corresponding mostly to the lone pair on N.

As for the EFOs of the aryl ligand, for each of the two geometries we can find the aryl contribution to the sigma Co-aryl bond. The occupations of this EFOs are useful to compare the nucleophilic character of the aryl in the *nitrene* ***(INT-N)*** and *masked*

*nitrene (**INT-MaskN**)* (Figure S32). The former has an occupation of 0.49, while for the latter it's 0.54, indicating that the nucleophilic character of the aryl increases when the masked nitrene (**INT-MaskN**) is formed.

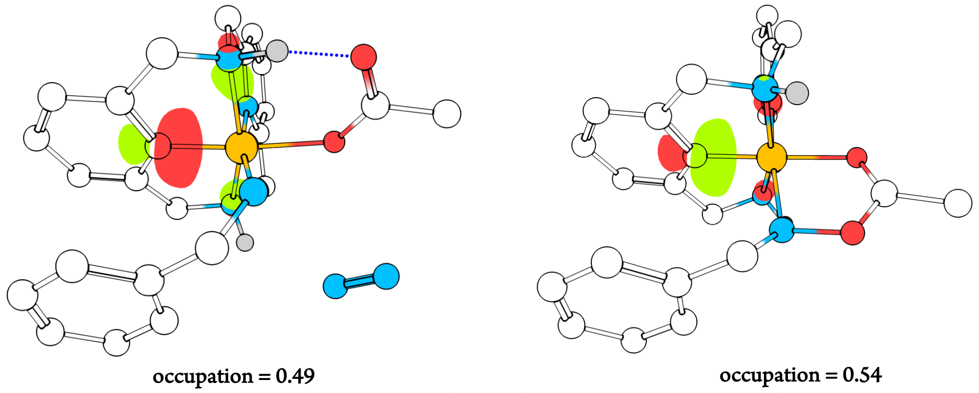

Figure S32. Comparison of the aryl EFO in the nitrene (**INT-N**) (left) and masked nitrene (**INT-MaskN**) (right) geometries.

*10.3 Analysis of the MO-LCAO coefficients for nitrene (**INT-N**)*

Visual representation of the MOs

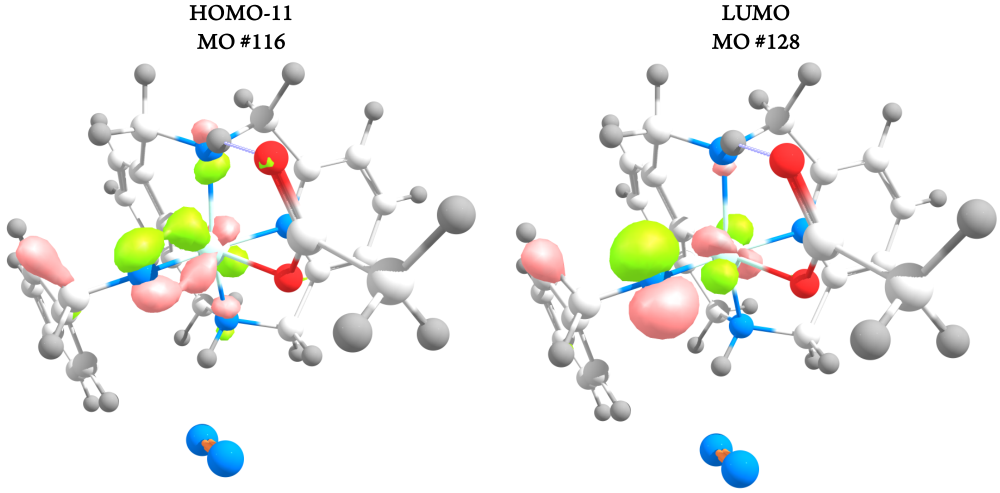

Figure S33. BP86-D3BJ(SMD)/Def2SVP MOs (level of theory used for the geometry optimizations).

The **HOMO-11** is a π bonding molecular orbital formed by the overlapping of the d_{yz} orbital of the metal, and the p_z of the nitrogen (bonding interaction). As it can be seen in Table S10, the major contribution to the **HOMO-11** is due to the *d* orbitals of Co, which agrees with the fact that for very high values of the isosurfaces the **HOMO-11** is only localized on Co.[31] The **LUMO,** on the other hand, is the corresponding π anti-bonding molecular orbital. The major contribution to the **LUMO** is given by the p_z orbital of the N, which agrees with the fact that for very high values of the isosurfaces the **LUMO** is only localized on the N. Therefore, the analysis of the canonical MOs is in complete agreement with the EOS method description of **INT-N** as aryl-Co^{III}=N-R nitrene.

Table S10. MO-LCAO coefficients of **HOMO-11** and **LUMO** for the Co *d* atomic orbitals and the N of the nitrenoid *p* atomic orbitals (left), and the corresponding squared (right) for nitrene (**INT-N**).

		**MO #**	**116**	**128**	**116**	**128**
	AO #		**HOMO-11**	**LUMO**	**HOMO-11**	**LUMO**
**Cobalt *d* atomic orbitals**	43	9D 0	0.317	-0.278	0.100	0.077
	44	9D+1	0.246	-0.141	0.060	0.020
	45	9D-1	-0.060	0.215	0.004	0.046

	46	9D+2	0.125	-0.262	0.016	0.069
	47	9D-2	0.139	-0.171	0.019	0.029
	48	10D 0	0.132	-0.100	0.017	0.010
	49	10D+1	0.104	-0.051	0.011	0.003
	50	10D-1	-0.022	0.077	0.000	0.006
	51	10D+2	0.048	-0.095	0.002	0.009
	52	10D-2	0.055	-0.062	0.003	0.004
Nitrenoid *p* atomic orbitals	438	4PX	0.007	0.042	0.000	0.002
	439	4PY	0.116	0.265	0.014	0.070
	440	4PZ	0.218	0.410	0.047	0.168
	441	5PX	-0.004	-0.025	0.000	0.001
	442	5PY	-0.088	-0.209	0.008	0.044
	443	5PZ	-0.165	-0.312	0.027	0.097

10.4 Study of the S=1 and S=2 states

Table S11. Electronic Energy differences for the triplet and quintuplet BP86-D3BJ/Def2SVP single point calculations at the singlet equilibrium geometry with respect to the singlet electronic energy (in kcal/mol).

	ΔE (Triplet – Singlet)	ΔE (Quintuplet – Singlet)
**Reactant complex**	32.75	79.59
**TS1 (N_2 release)**	14.35	54.15
**Nitrene**	4.93	47.49
**TS2 (Nitrene to Ring)**	1.61	45.16
**Ring**	31.66	80.45
**TS3 (C-N coupling)**	18.34	48.85
**Coupled**	17.12	44.03

*10.5 Analysis of the triplet Nitrene **(INT-N)***

For the nitrene ***(INT-N)***, the singlet → triplet spin-crossing involves a beta electron of the lone pair of the N atom in the nitrene. This is evidenced by the spin density, which is located around this nitrogen, as can be seen from its isosurface plot, and the integrated atomic values:

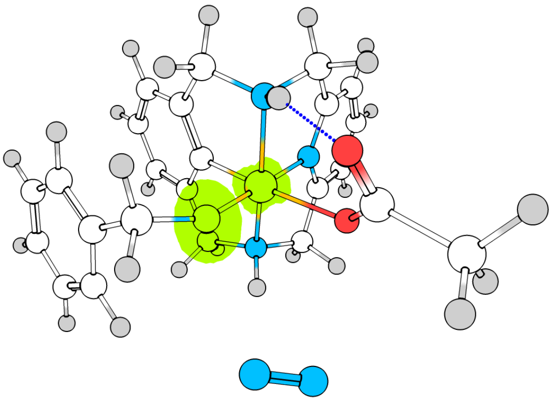

Figure S34. Spin density isosurface (isovalue = 0.03). It can be seen that the major contribution is located around the nitrogen atom.

Table S12. Atomic spin densities for this triplet nitrene ***(INT-N)***. For both the real-space partitioning, and the Hilbert-space one, the spin density is mostly located on the nitrogen.

	**Co spin density**	**N spin density**
**TFVC (Real space)**	0.5617	1.1976
**Mulliken (Hilbert space)**	0.5119	1.3348

The results of the EOS analysis for the triplet also shed light into the electronic structure of the triplet state. For the Co-N sigma interaction (see figure S35), the picture is similar to that of the singlet species. The sigma-type EFO on the N atom has a much larger occupation than the complementary d-type EFO on Co. Some spin polarization can be observed (the occupation of the alpha and beta EFOs differ), but both alpha and beta electrons (essentially the electron pair) is assigned to the sigma-donating N moiety.

The Co-N π interaction is the one affected by the singlet → triplet transition. The EOS analysis for the singlet species revealed the presence of a lone pair on N and a single π Co-N bond polarized towards the Co. For the S=0 state, the overall electron count for the N moiety results in an electron pair for the sigma part and a lone electron pair, thus four valence electrons.

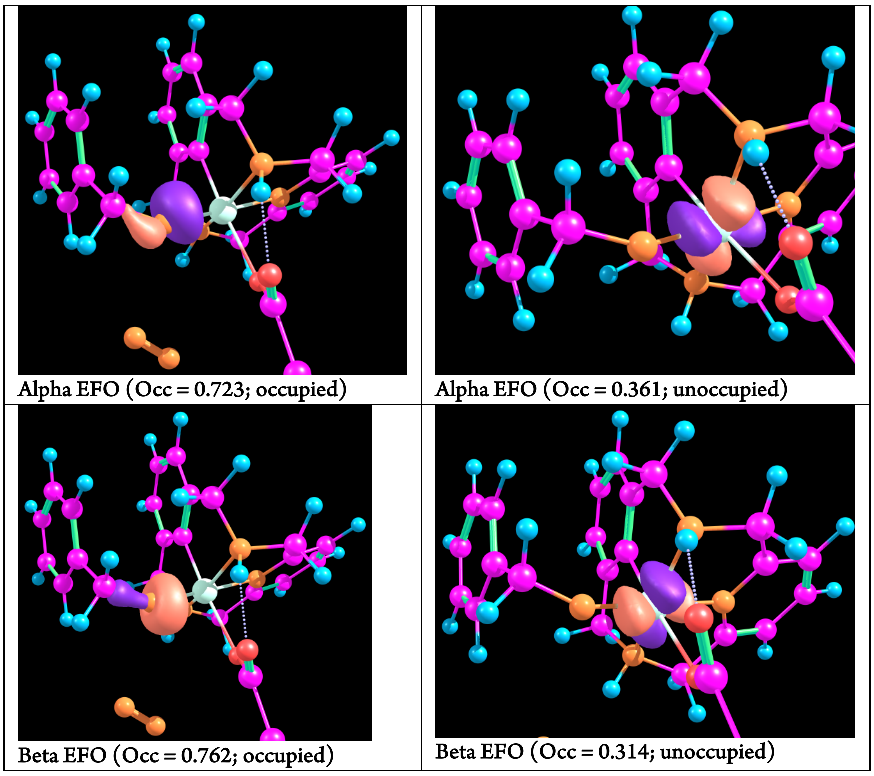

Figure S35. EFOs involved in Co-N sigma interaction in triplet Nitrene ***(INT-N)***.

The beta electron of the lone pair of the singlet is transferred (as alpha) to a formally empty p-type EFO on the N, resulting in a triplet state with two alpha p-type nonbonding electrons (see figure S36). At the same time a second π bond is established between Co-N involving the beta electrons (see figure S37). Both π bonds of the beta spin channels are polarized towards the Co moiety, as evidenced by the EFO occupations of Figure S37. The overall electron count for the N moiety results in an electron pair for the sigma part and two alpha non-bonding pi electrons, giving a total of four valence electrons, like for the singlet state. Hence, the nitrene character of the species is retained for both the singlet and triplet states.

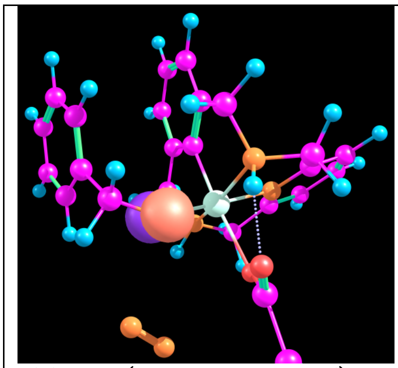

Alpha EFO (Occ = 0.943; occupied)

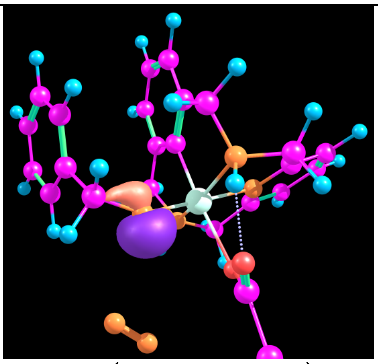

Alpha EFO (Occ = 931, occupied)

Figure S36. Alpha EFOs involved in Co-N π interaction in triplet Nitrene ***(INT-N)***.

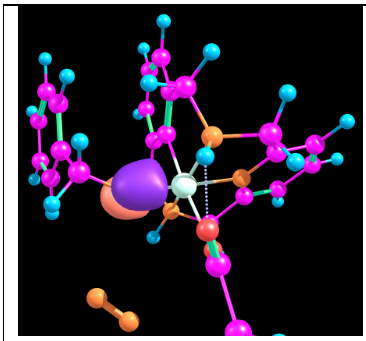

Beta EFO (Occ = 0.237; unoccupied)

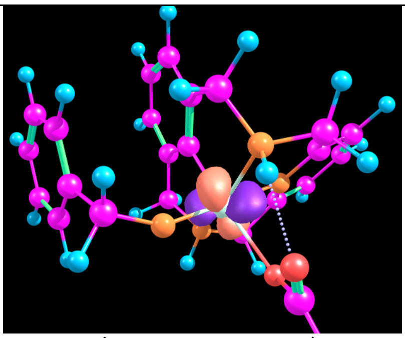

Beta EFO (Occ = 0.685; occupied)

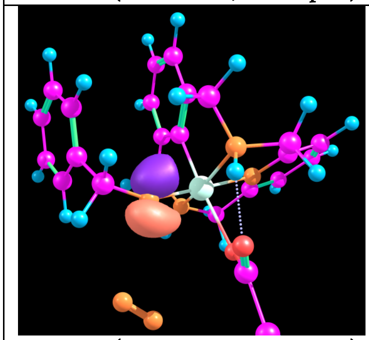

Beta EFO (Occ = 0.255; unoccupied)

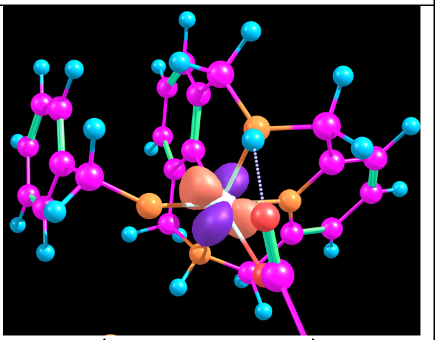

Beta EFO (Occ = 0.674; occupied)

Figure S37. Beta EFOs involved in Co-N π interaction in triplet Nitrene ***(INT-N)***.

*10.6 Attempts to find a stable penta-coordinated intermediate for Nitrene **(INT-N)***

Several relaxed PES scans were performed to look for a penta-coordinated intermediate for singlet Nitrene ***(INT-N)***. Reference image for the numbered atoms, which will be relevant for the scans below:

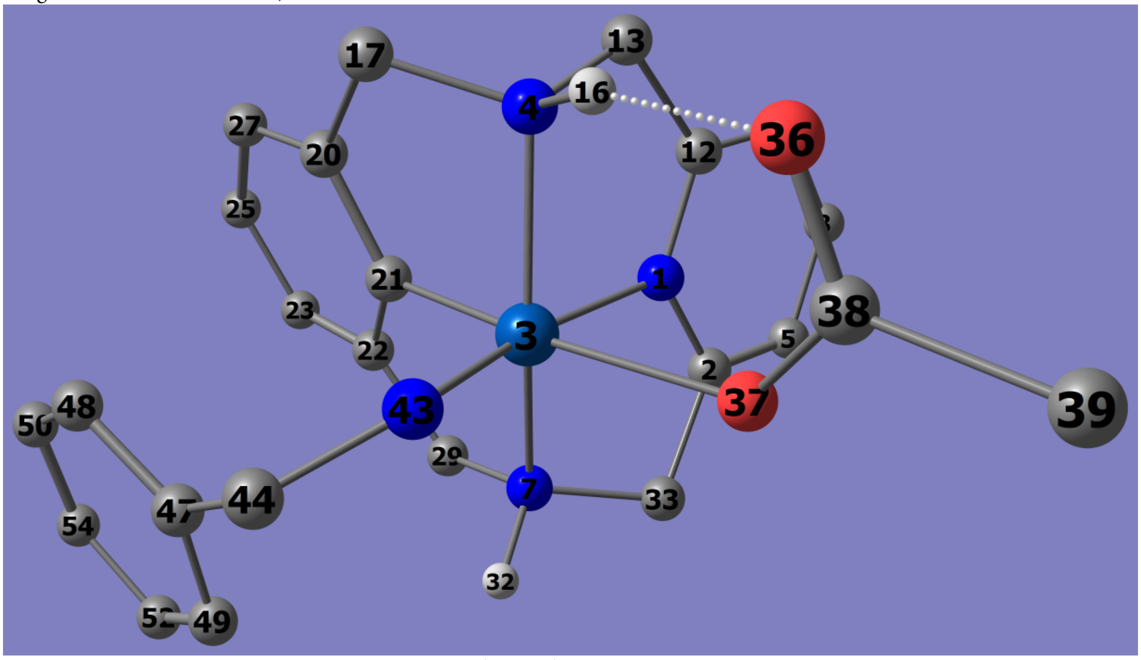

Figure S38. Numbered atoms for the Nitrene ***(INT-N)*** geometry. The labels are used in the following discussion.

a. Scanning along Co-N distances

Bond 3-1: energy increase.

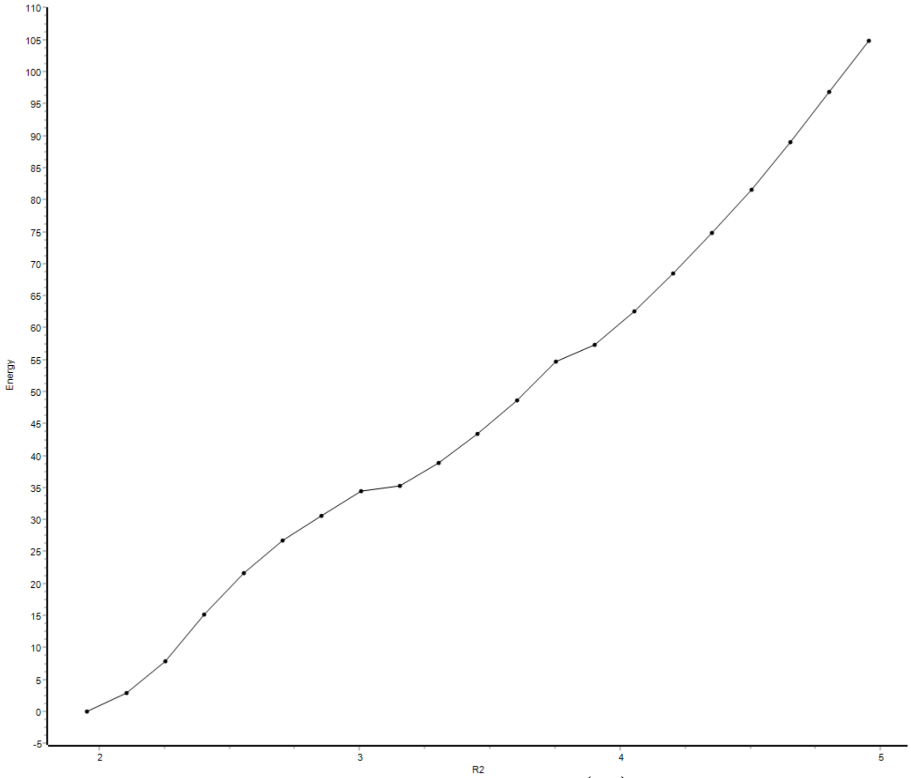

Figure S39. Relaxed PES scan along the Co-N (3,1) bond length.

Bond 3-4: energy increase.

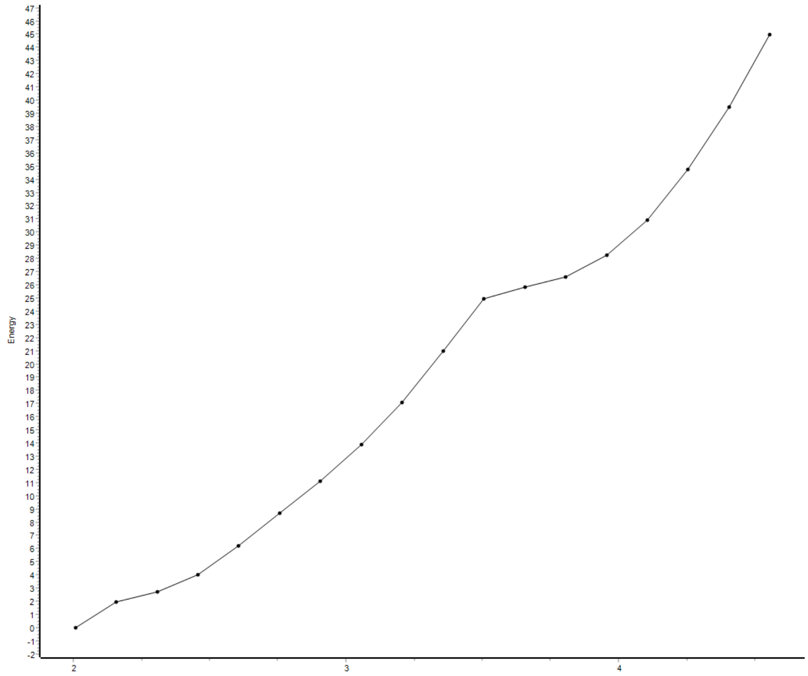

Figure S40. Relaxed PES scan along the Co-N (3,4) bond length.

Bond 3-7: energy increase. After some steps, the geometry is distorted to the point that the final product is formed (C-N bond coupling), leading to a decrease in energy. The barrier, of course, is much higher than the one of the mechanism shown in the manuscript.

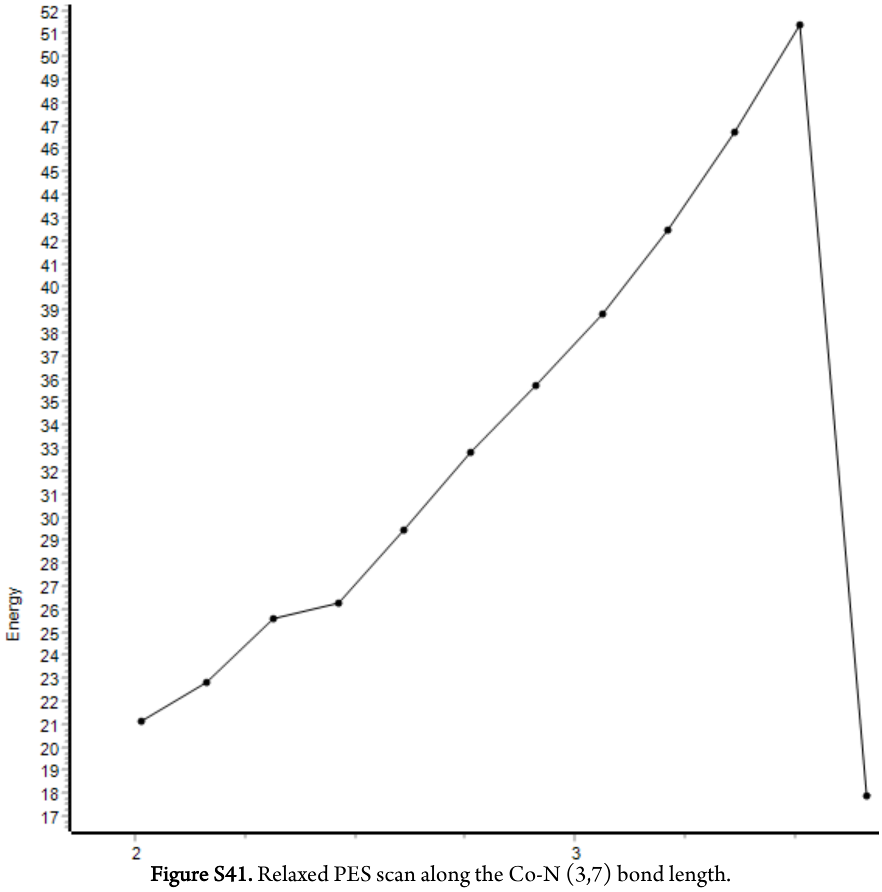

Figure S41. Relaxed PES scan along the Co-N (3,7) bond length.

b. Scans along N-C-C-N (4-13-12-1) dihedral

Both the positive and negative directions for the change of this dihedral were explored:
Positive direction: energy increase.

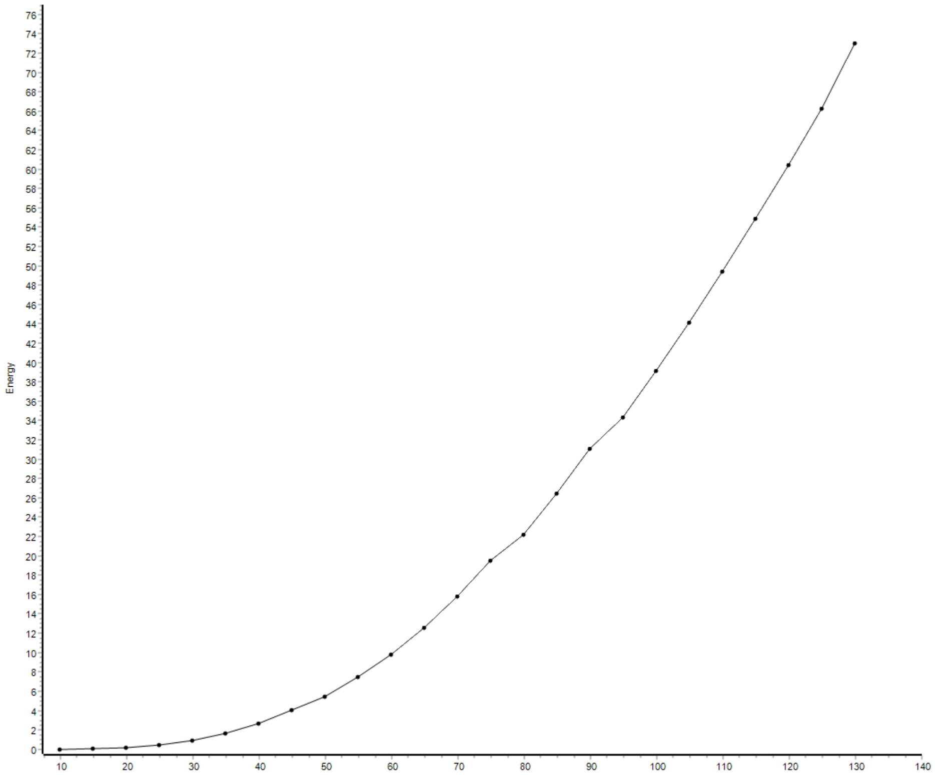

Figure S42. Relaxed PES scan along the N-C-C-N (4,13,12,1) dihedral angle.

Negative direction: energy increase. After some steps, the O-N bond (36-43) is formed, leading to the masked nitrene ***(INT-MaskN)***. Thus, the search is stopped because it's not going to lead to possible alternative stable nitrene ***(INT-N)*** intermediates. Once again, the barrier observed here is much higher than the barrier for the ring formation in our proposed mechanism.

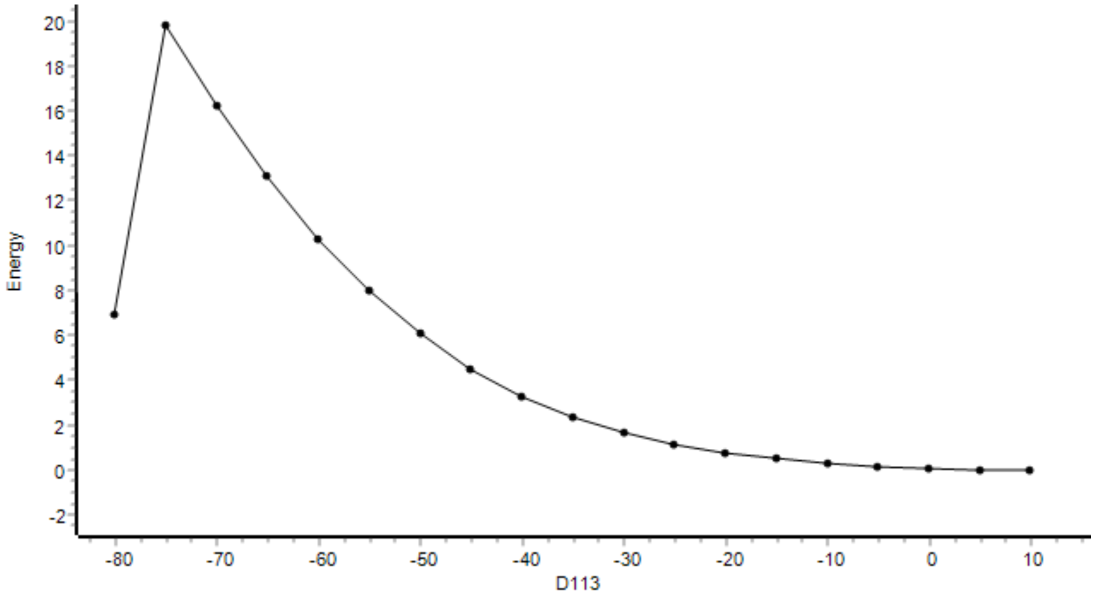

Figure S43. Relaxed PES scan along the N-C-C-N (4,13,12,1) dihedral, negative direction.

c. Designs of penta-coordinated intermediates

Penta-coordinated geometries were designed where the pyridine moiety is no longer coordinating (what we would have expected from the scans in subsections *a* and *b*, if such an intermediate existed).
Since the disconnected pyridine could rotate toward two different directions, two geometries were designed. Both geometries were optimized using two approaches: direct optimization, or step-wise optimization (first relaxing everything while the 3-1 bond was frozen, and then optimizing the resulting geometry). In all cases, the final optimized geometry was the same as Figure S38. For the triplet state, the geometries also optimized back to the original geometry, not leading to new alternative penta-coordinated intermediates.

11. DFT XYZ coordinates of geometry optimized structures

The optimized XYZ Cartesian coordinates for all the structures can be found in the following database link, in a very convenient format and allowing easy visualization and extraction of the XYZ file if needed: https://doi.org/10.19061/iochem-bd-4-29

12. Original NMR data

12.1 Cobalt complexes

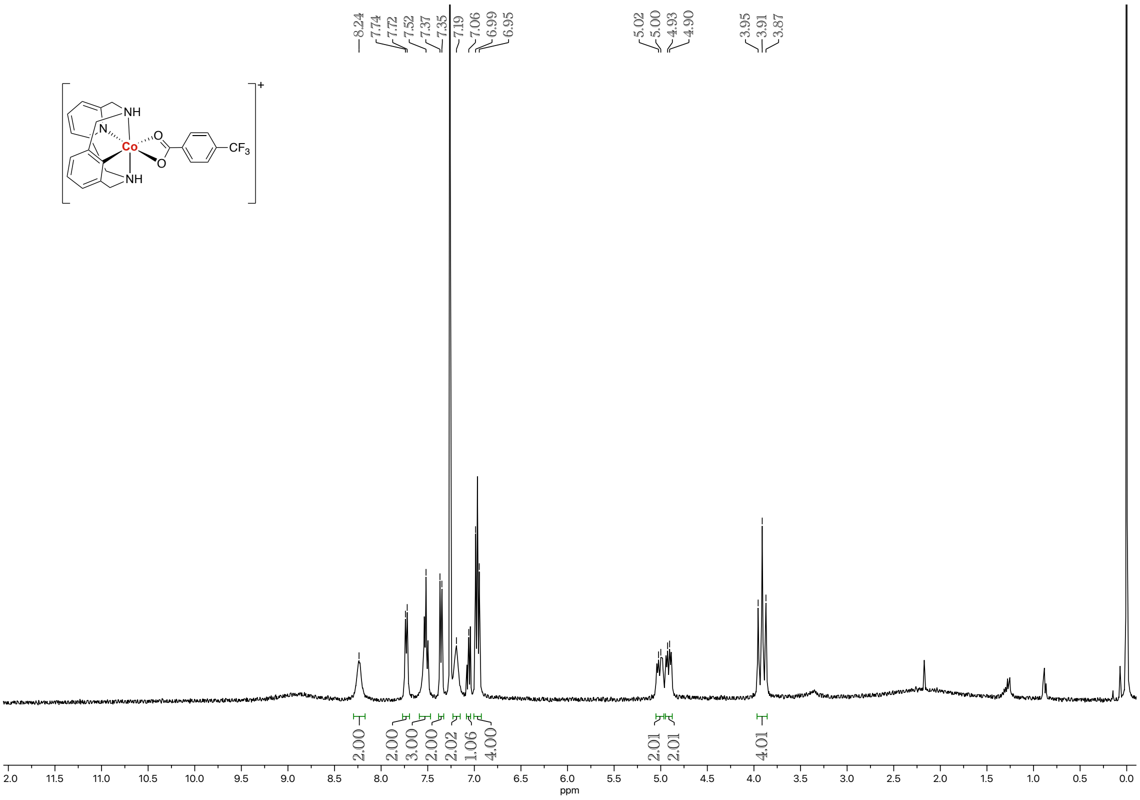

Figure S44. 400 MHz, ^{1}H-NMR spectrum of **1-OBzCF$_3$** in $CDCl_3$, 298 K.

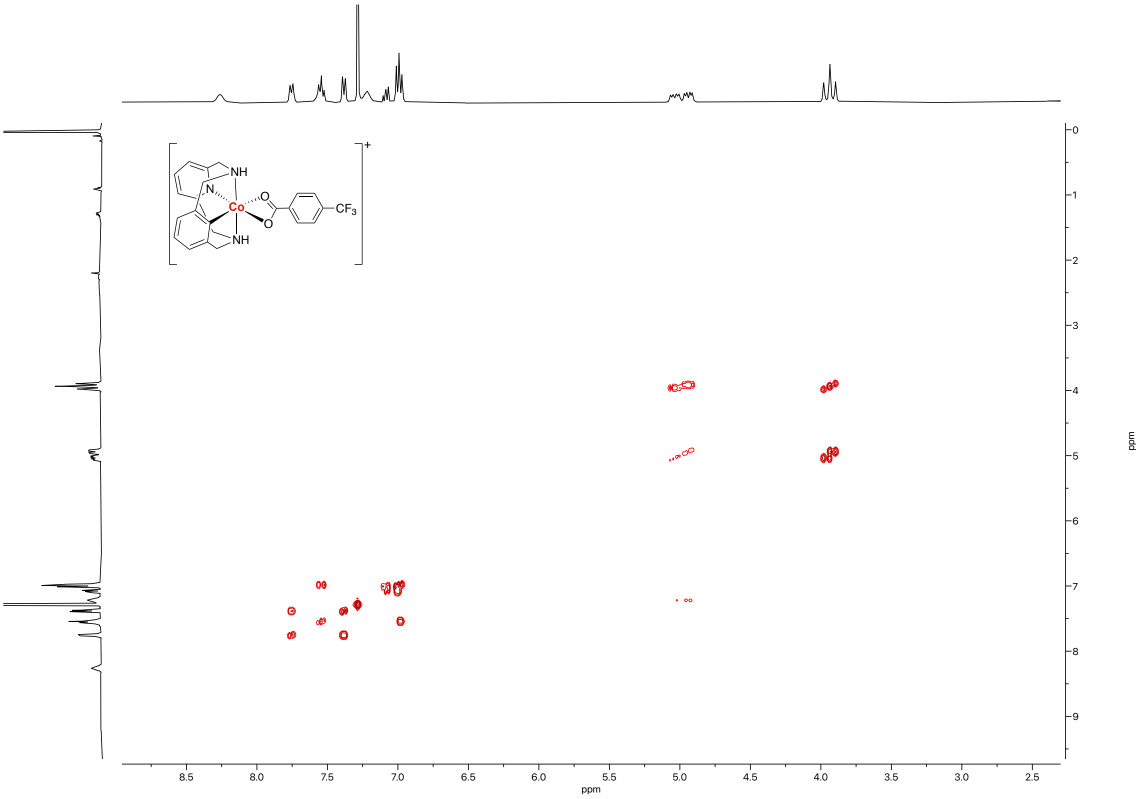

Figure S45. 400 MHz, ^{1}H-^{1}H COSY NMR spectrum of **1-OBzCF$_3$** in $CDCl_3$, 298 K.

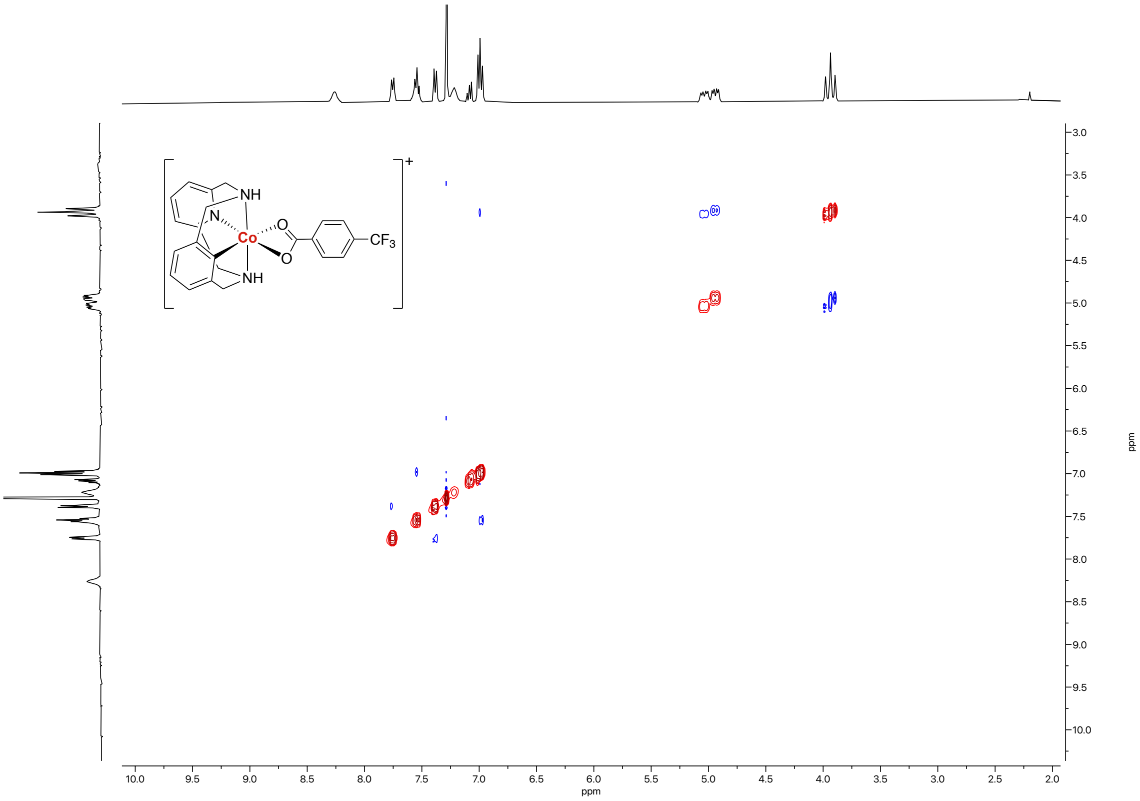

Figure S46. 400 MHz, ^{1}H-^{1}H NOESY NMR spectrum of **1-OBzCF$_3$** in $CDCl_3$, 298 K.

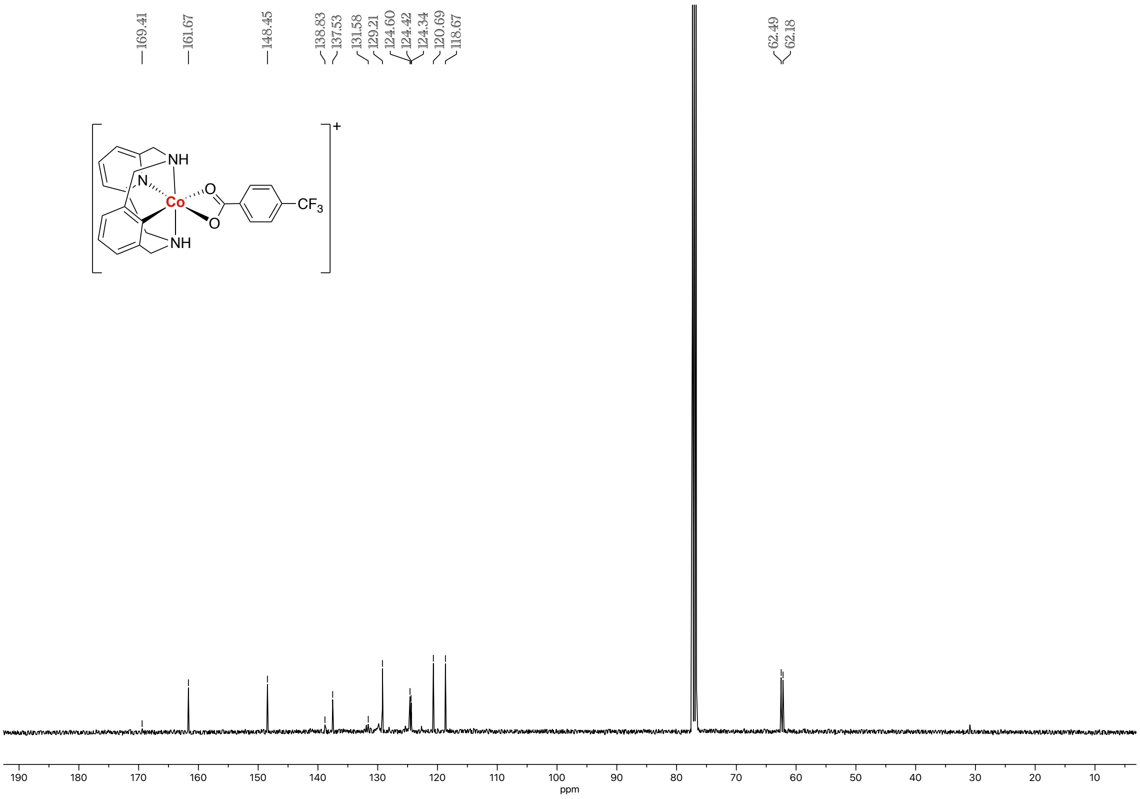

Figure S47. 100 MHz, ^{13}C {^{1}H} NMR spectrum of **1-OBzCF$_3$** in $CDCl_3$, 298 K.

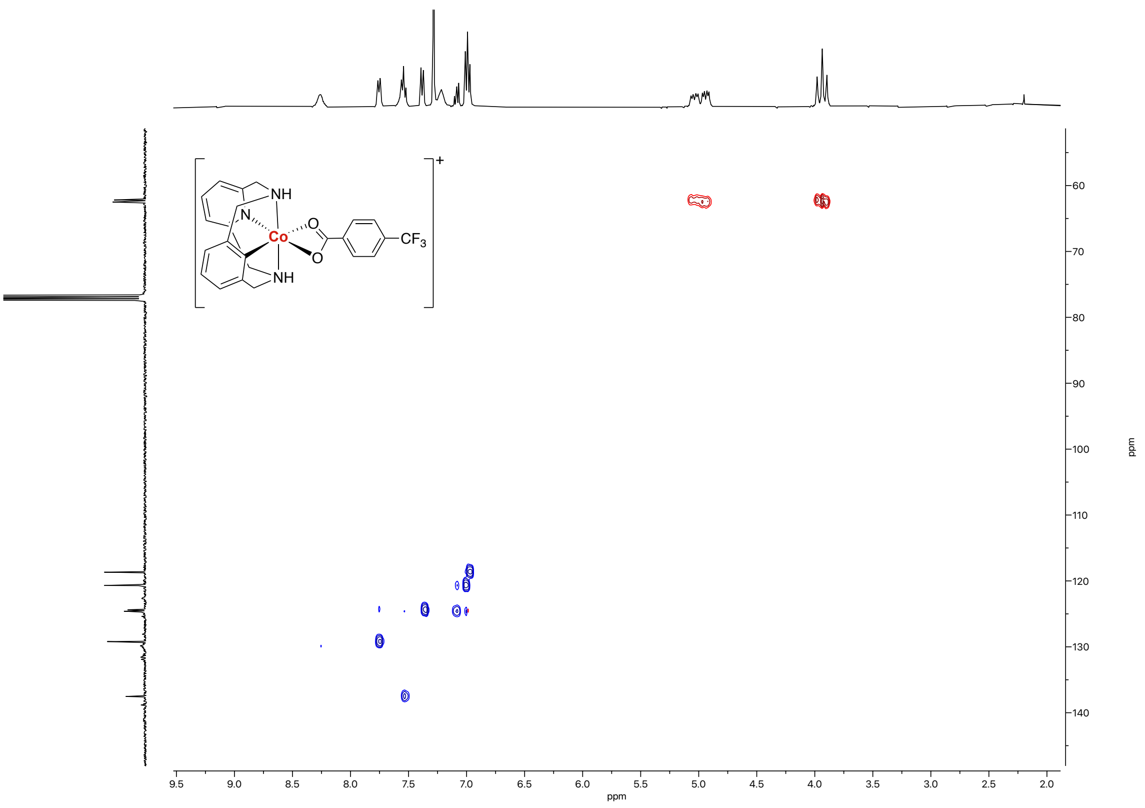

Figure S48. 400 MHz, ^{1}H-^{13}C HSQC NMR spectrum of **1-OBzCF$_3$** in $CDCl_3$, 298 K.

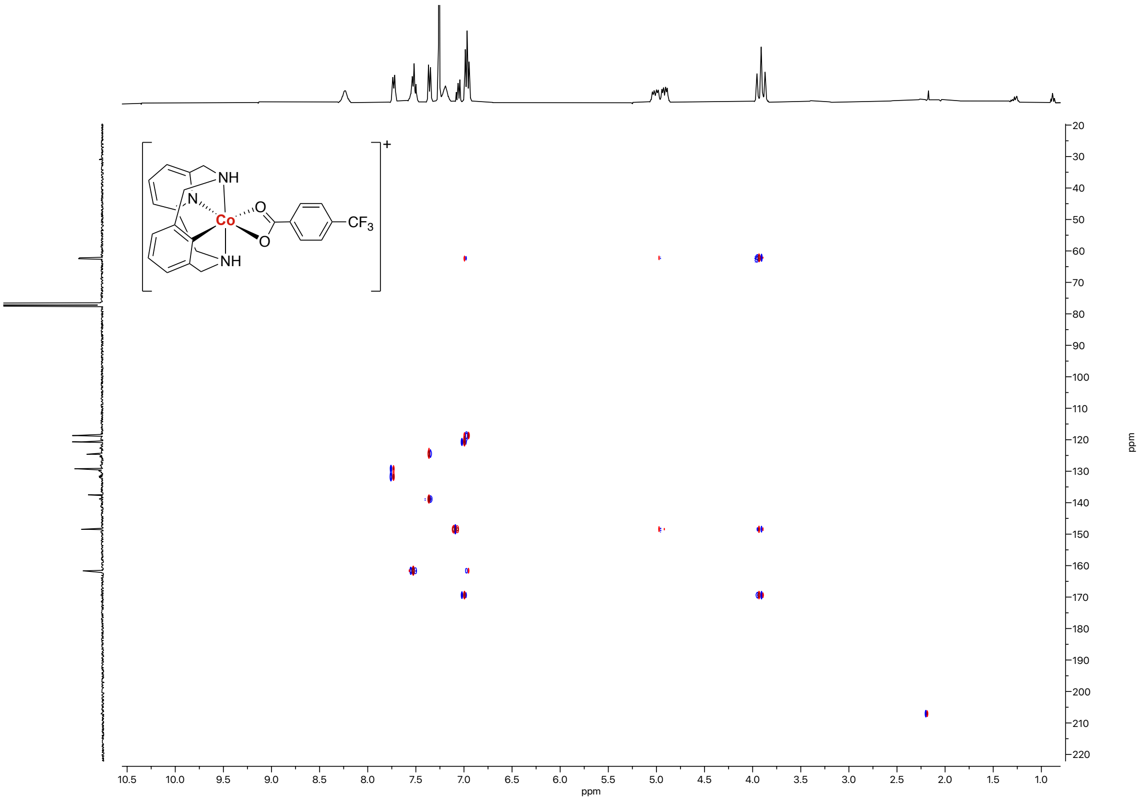

Figure S49. 400 MHz, ^{1}H-^{13}C HMBC NMR spectrum of **1-OBzCF$_3$** in $CDCl_3$, 298 K.

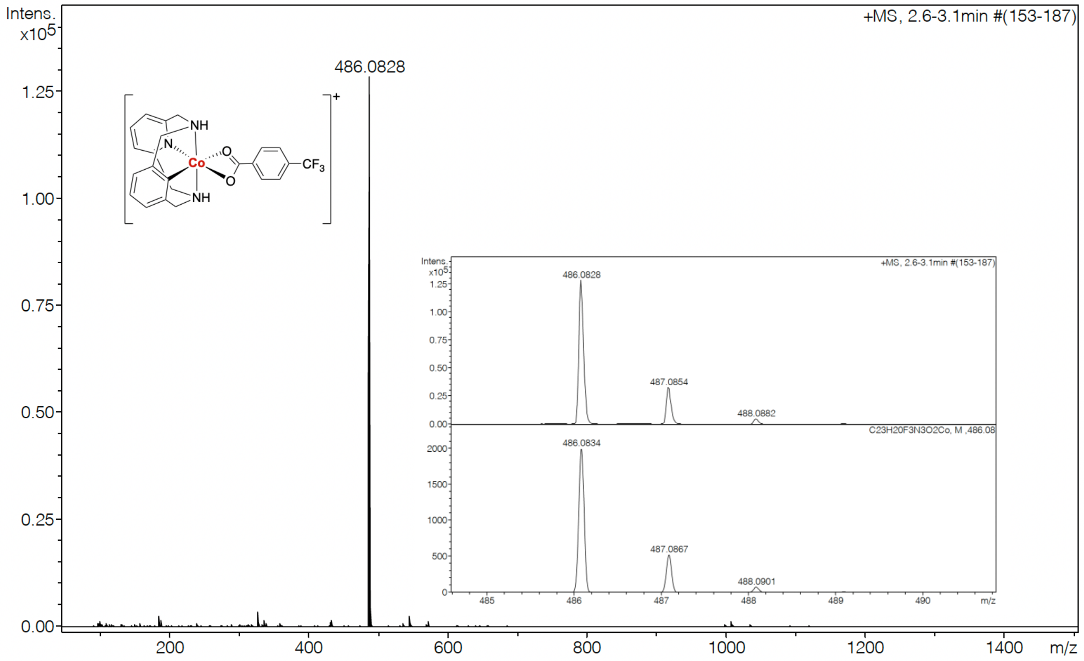

Figure S50. HRMS spectrum of **1-OBzCF₃** showing a peak at m/z = 486.0828. Inset: up, experimental spectrum; down, simulated spectrum.

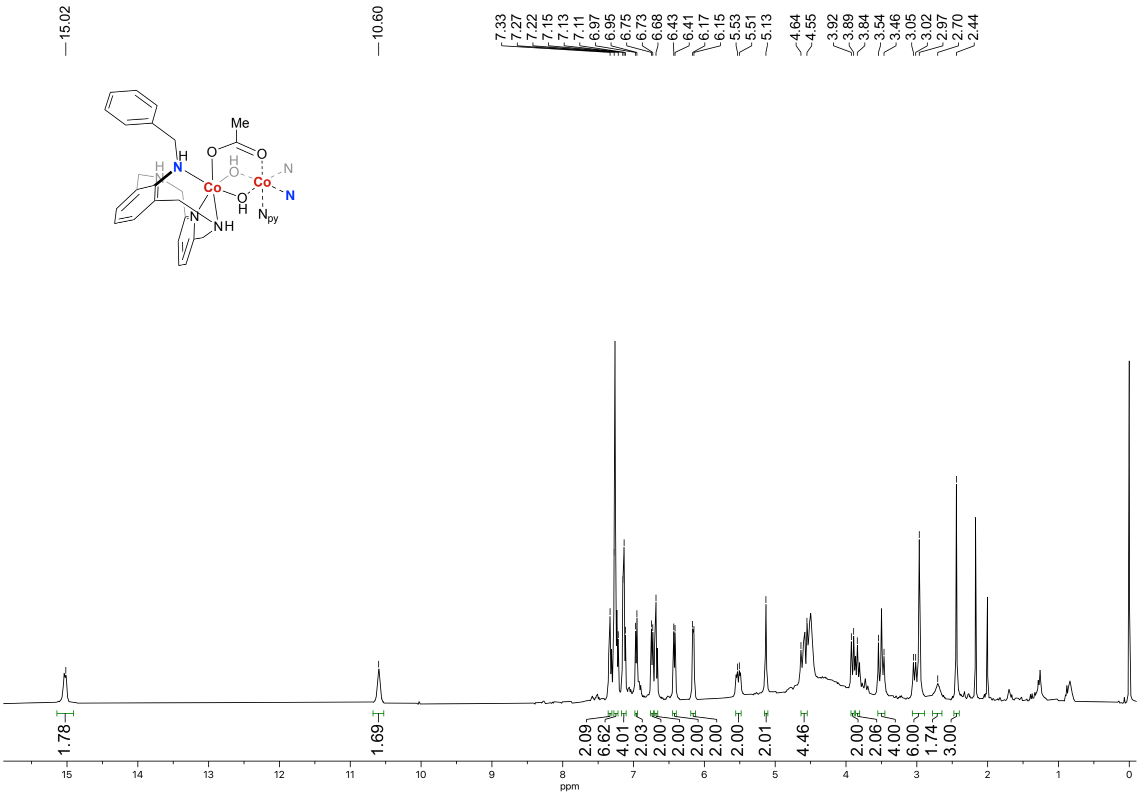

Figure S51. 500 MHz, ¹H NMR spectrum of **3a-OAc** in $CDCl_3$, 298 K.

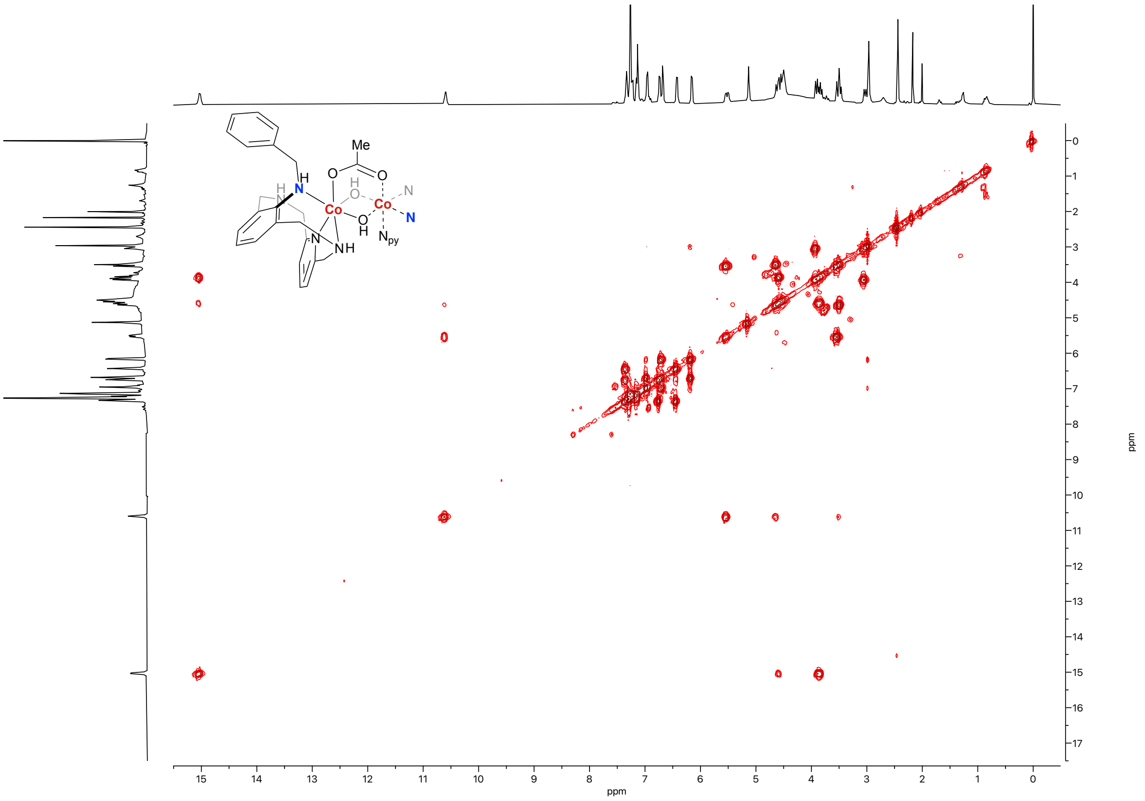

Figure S52. 500 MHz, ^{1}H-^{1}H COSY NMR spectrum of **3a-OAc** in $CDCl_3$, 298 K.

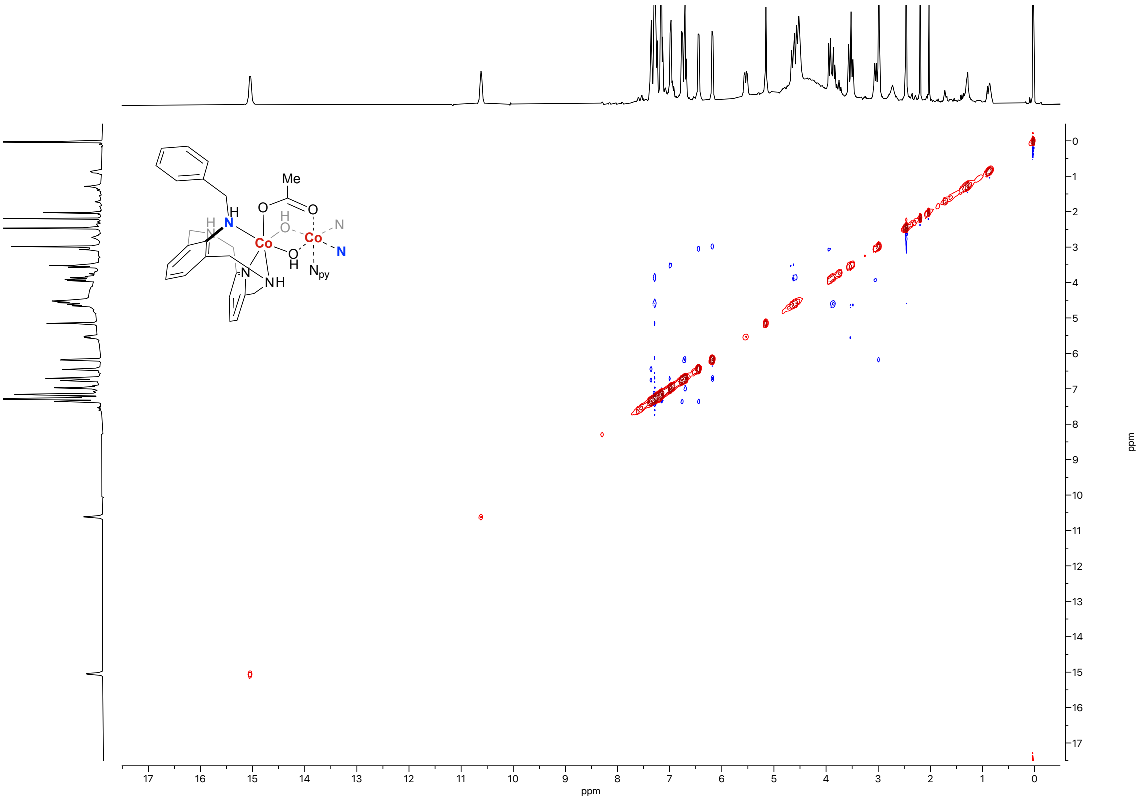

Figure S53. 500 MHz, ^{1}H-^{1}H NOESY NMR spectrum of **3a-OAc** in $CDCl_3$, 298 K.

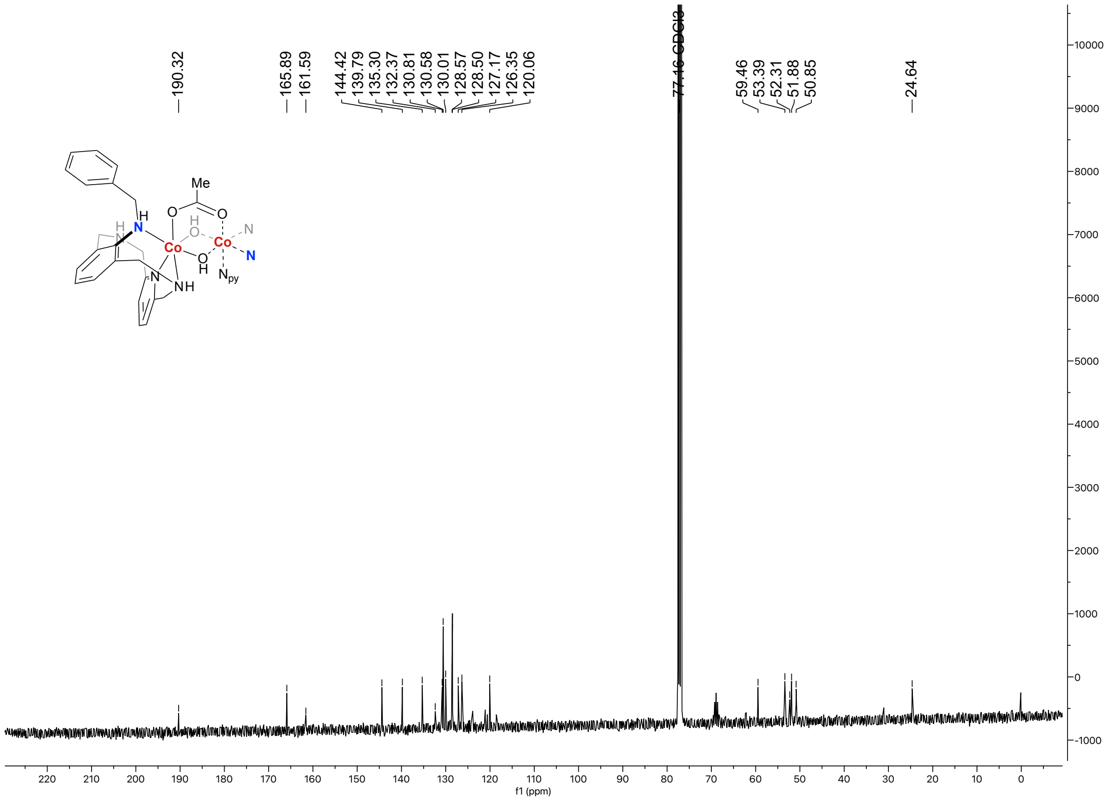

Figure S54. 125 MHz, $^{13}C\{^1H\}$ NMR spectrum of **3a-OAc** in $CDCl_3$, 298 K.

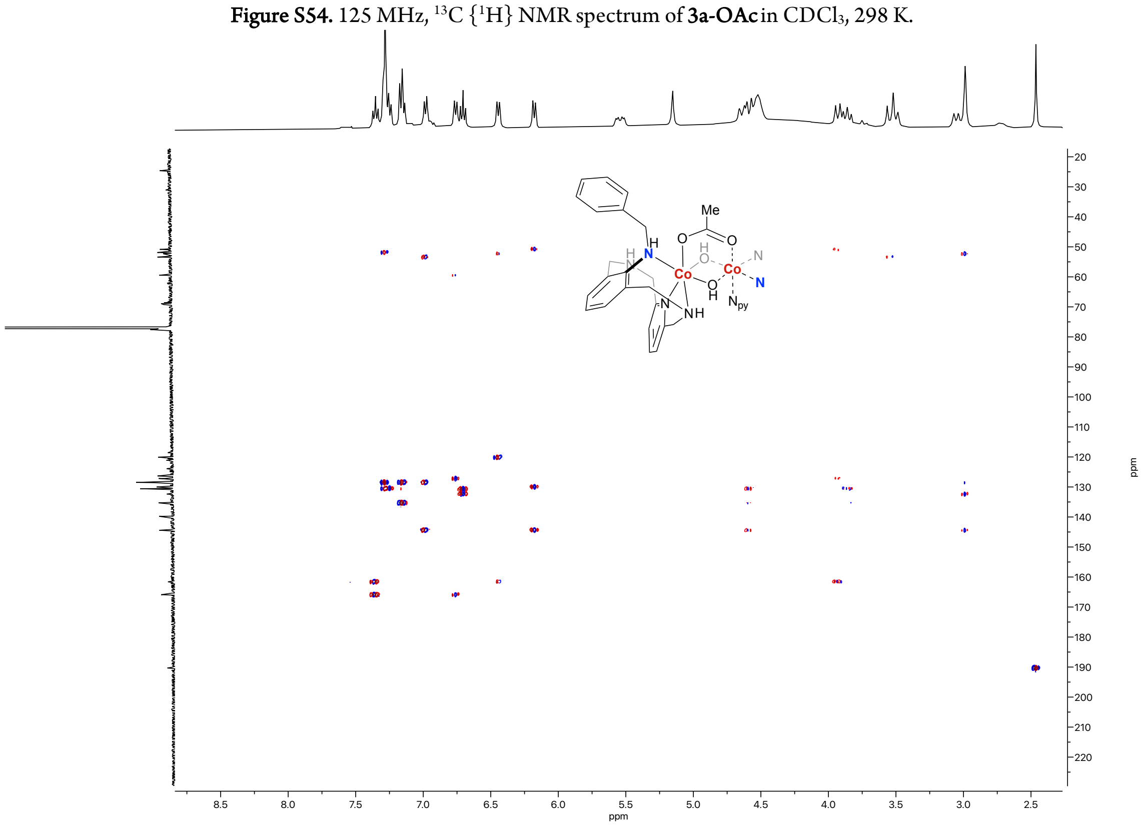

Figure S55. 500 MHz, ^1H-^{13}C HMBC NMR spectrum of **3a-OAc** in $CDCl_3$, 298 K.

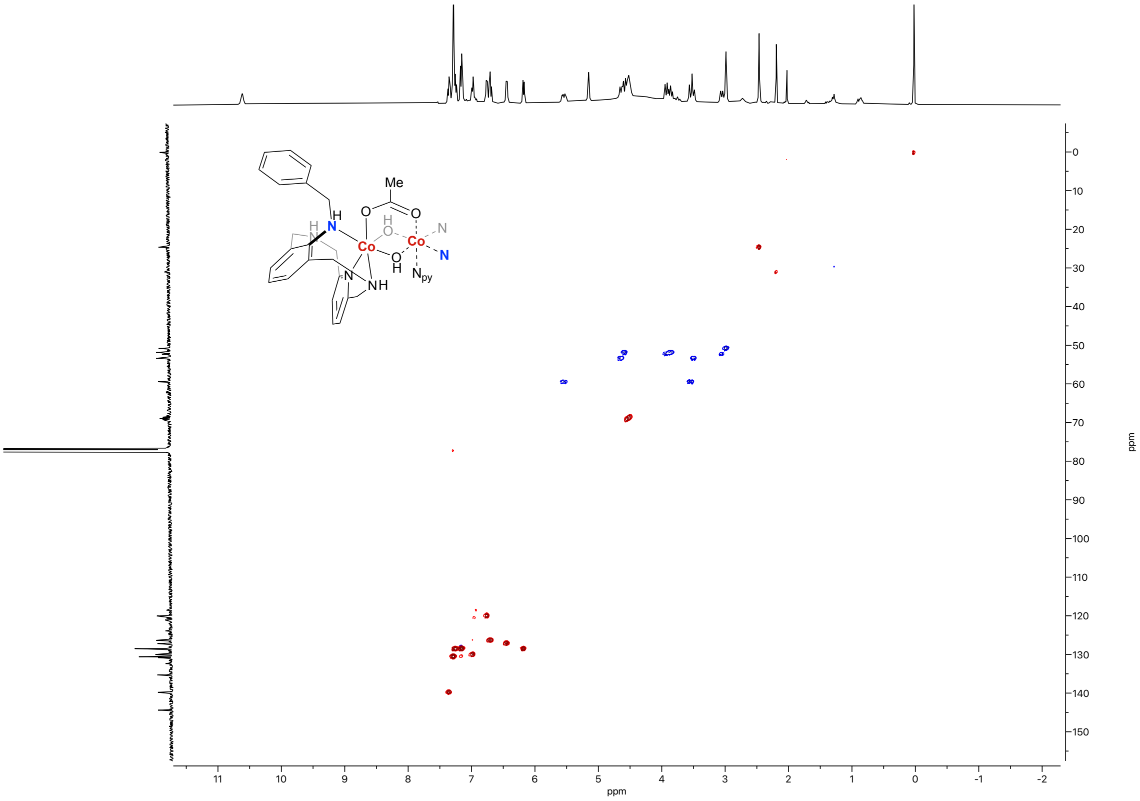

Figure S56. 500 MHz, ^{1}H-^{13}C HSQC NMR spectrum of **3a-OAc** in $CDCl_3$, 298 K.

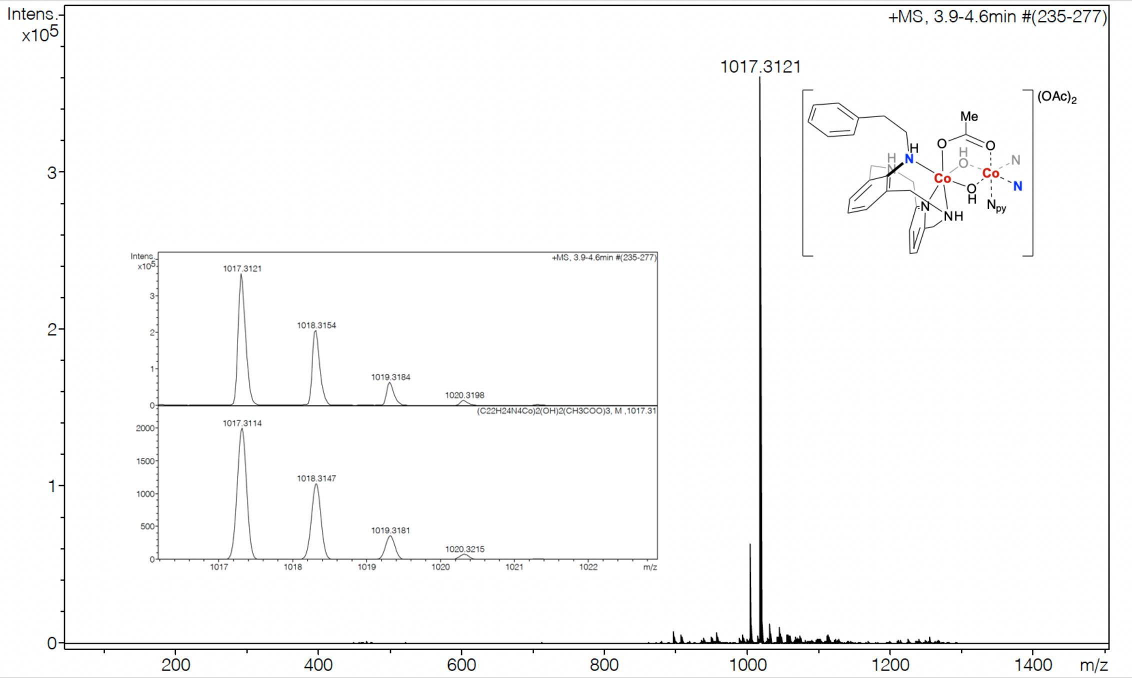

Figure S57. HRMS spectrum of **3a-OAc** showing a peak at $m/z = 1017.3121$. Inset: up, experimental spectrum; down, simulated spectrum.

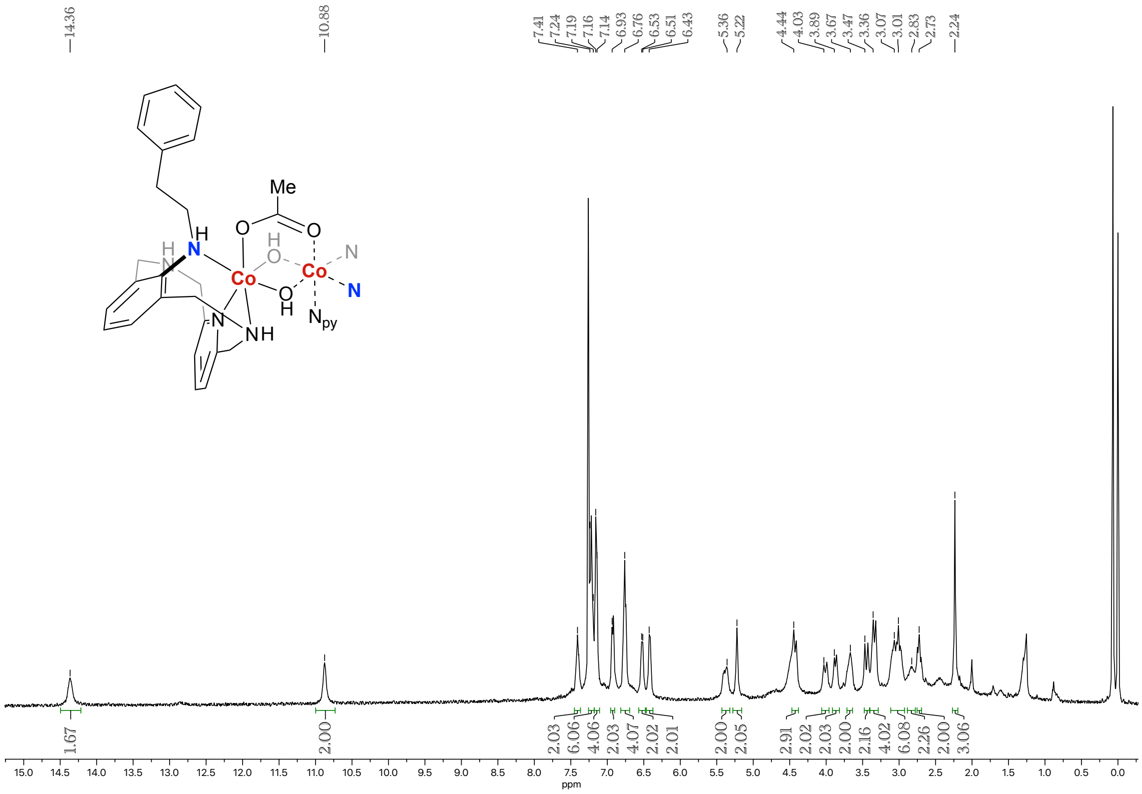

Figure S58. 400 MHz, ^{1}H NMR spectrum of **3b-OAc** in $CDCl_3$, 298 K.

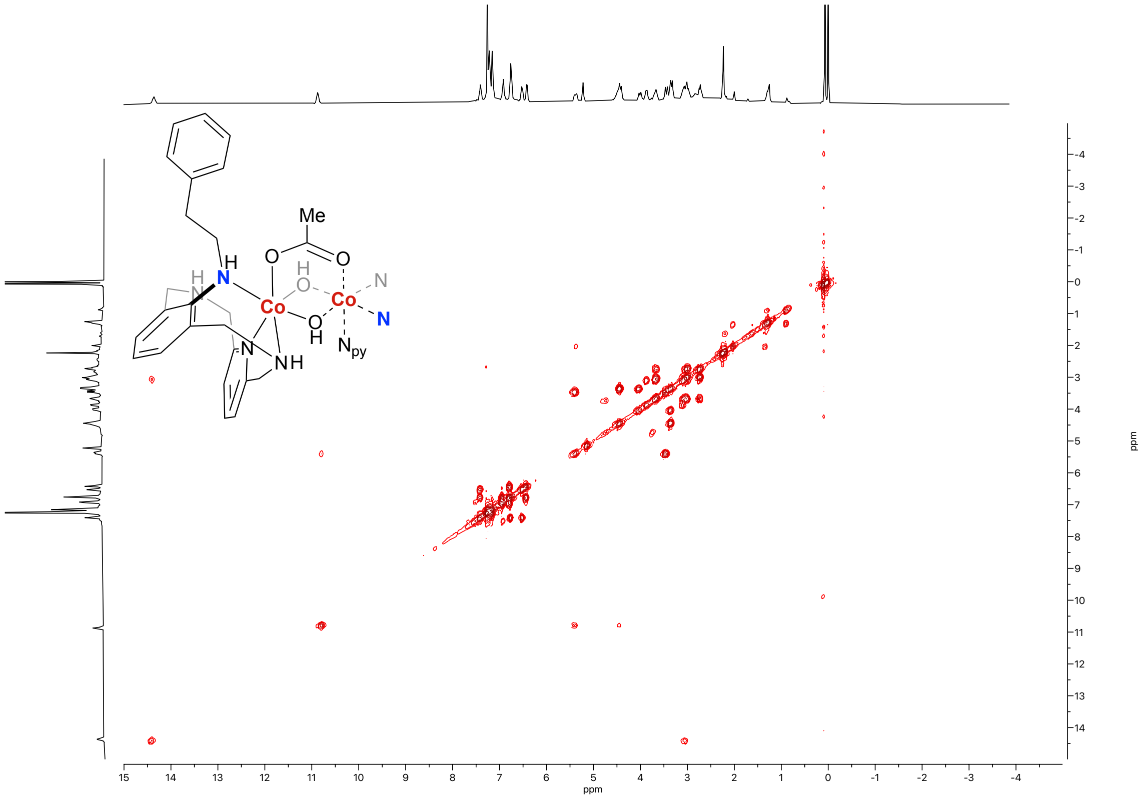

Figure S59. 400 MHz, ^{1}H-^{1}H COSY NMR spectrum of **3b-OAc** in $CDCl_3$, 298 K.

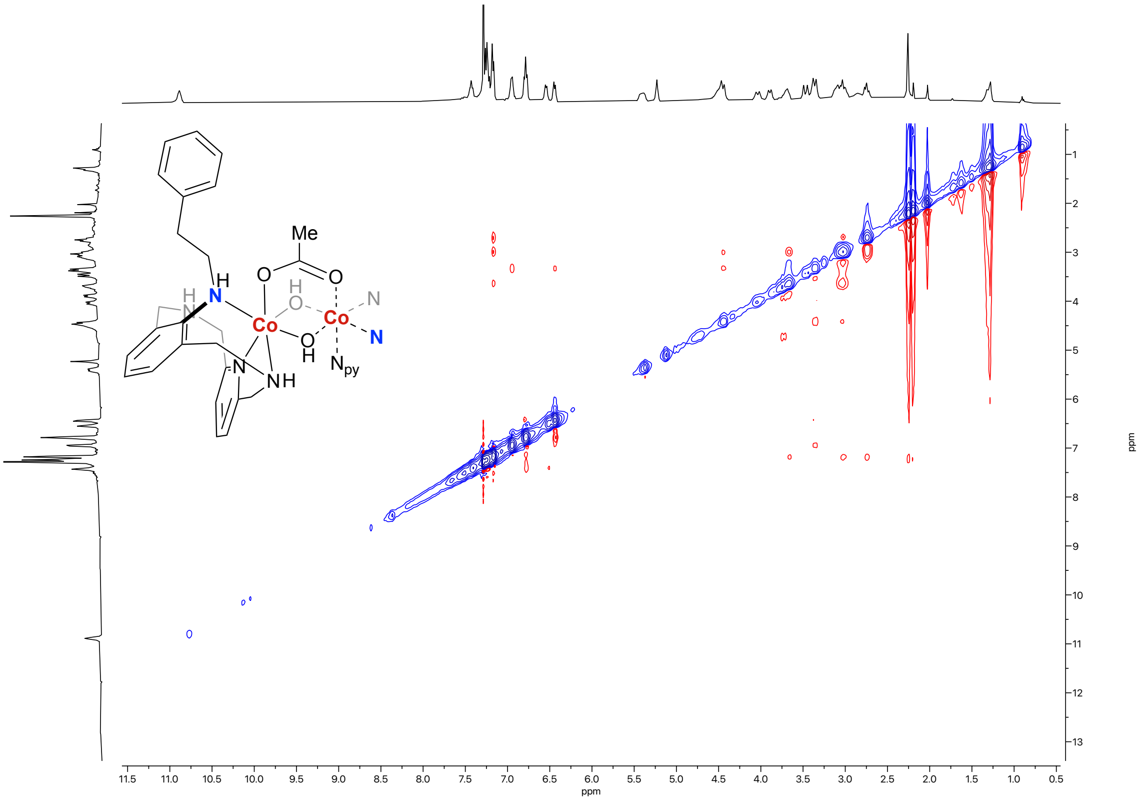

Figure S60. 400 MHz, ^{1}H-^{1}H NOESY NMR spectrum of **3b-OAc** in $CDCl_3$, 298 K.

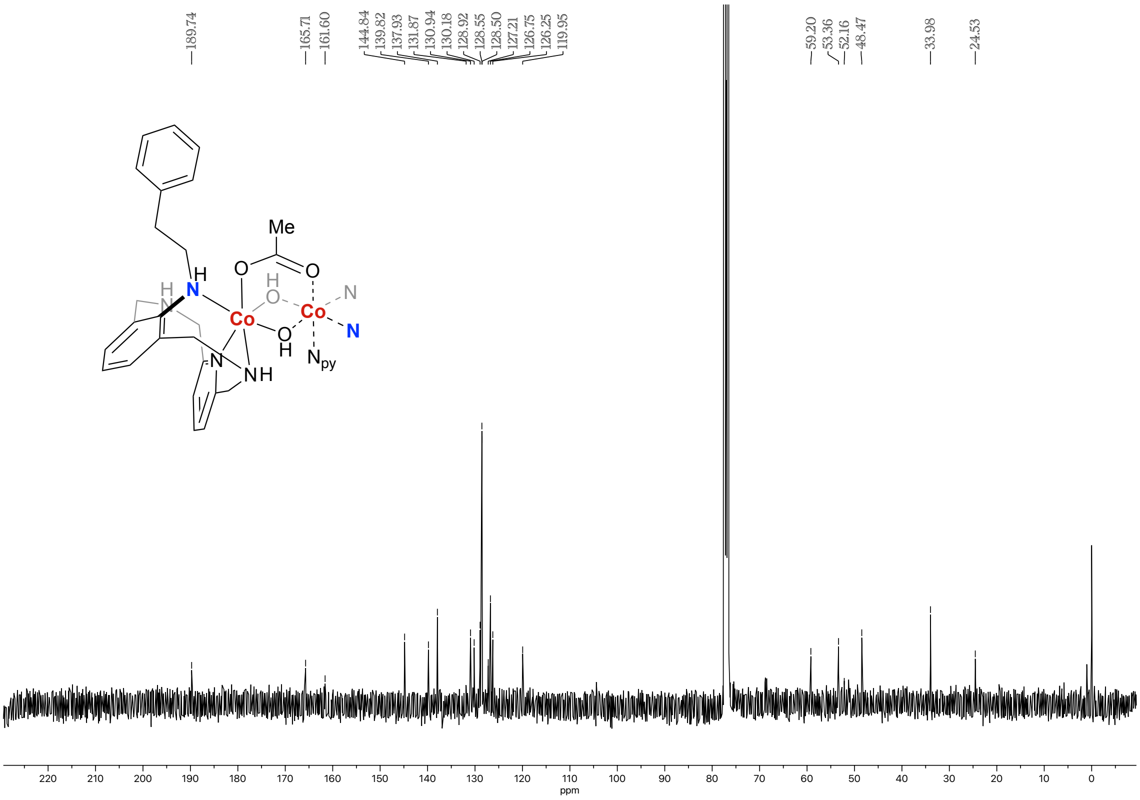

Figure S61. 100 MHz, ^{13}C {^{1}H} NMR spectrum of **3b-OAc** in $CDCl_3$, 298 K.

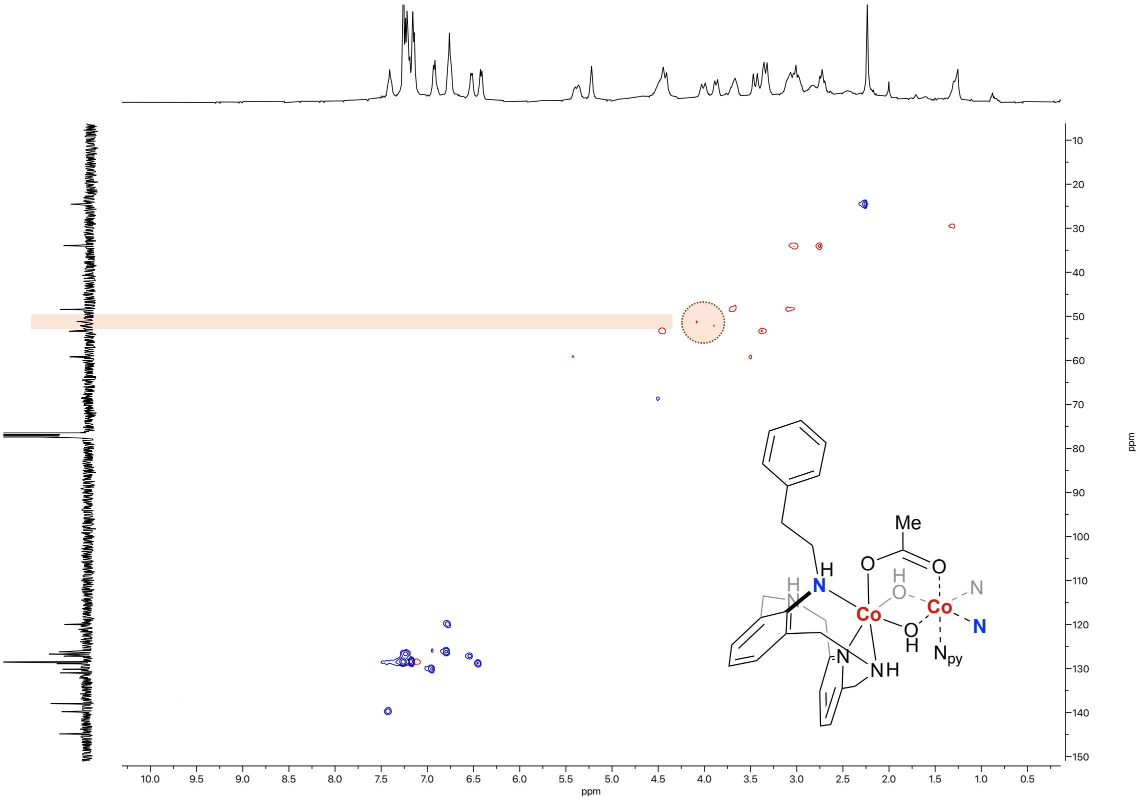

Figure S62. 400 MHz, ^{1}H-^{13}C HSQC NMR spectrum of **3b-OAc** in $CDCl_3$, 298 K.

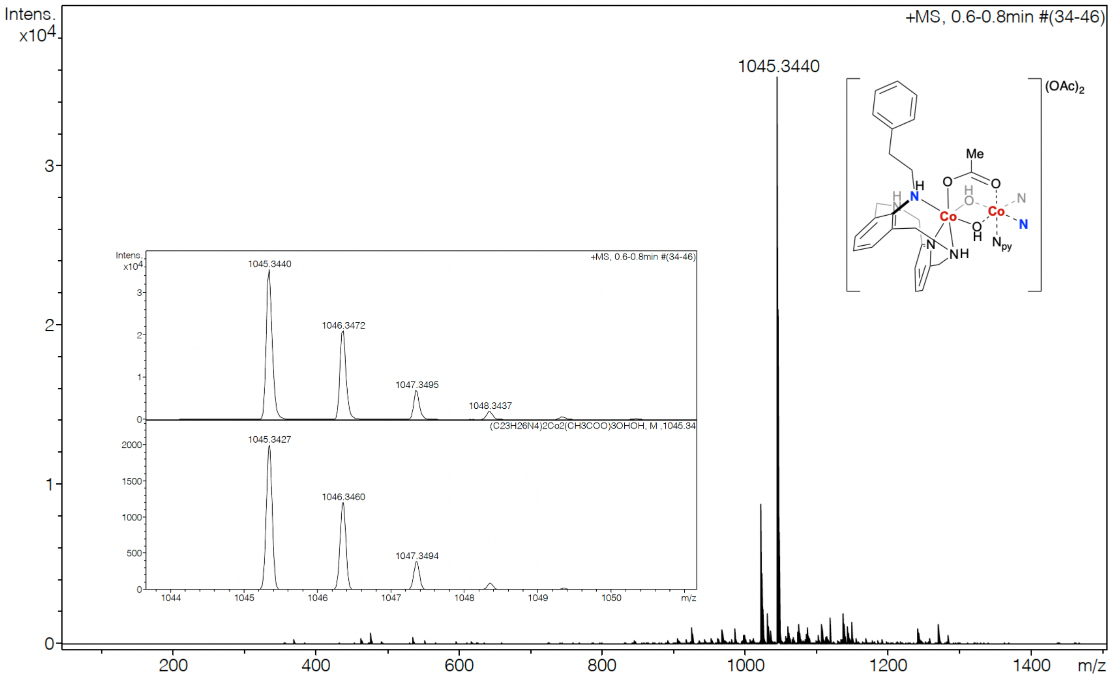

Figure S63. HRMS spectrum of **3b-OAc** showing a peak at m/z = 1045.3440. Inset: up, experimental spectrum; down, simulated spectrum.

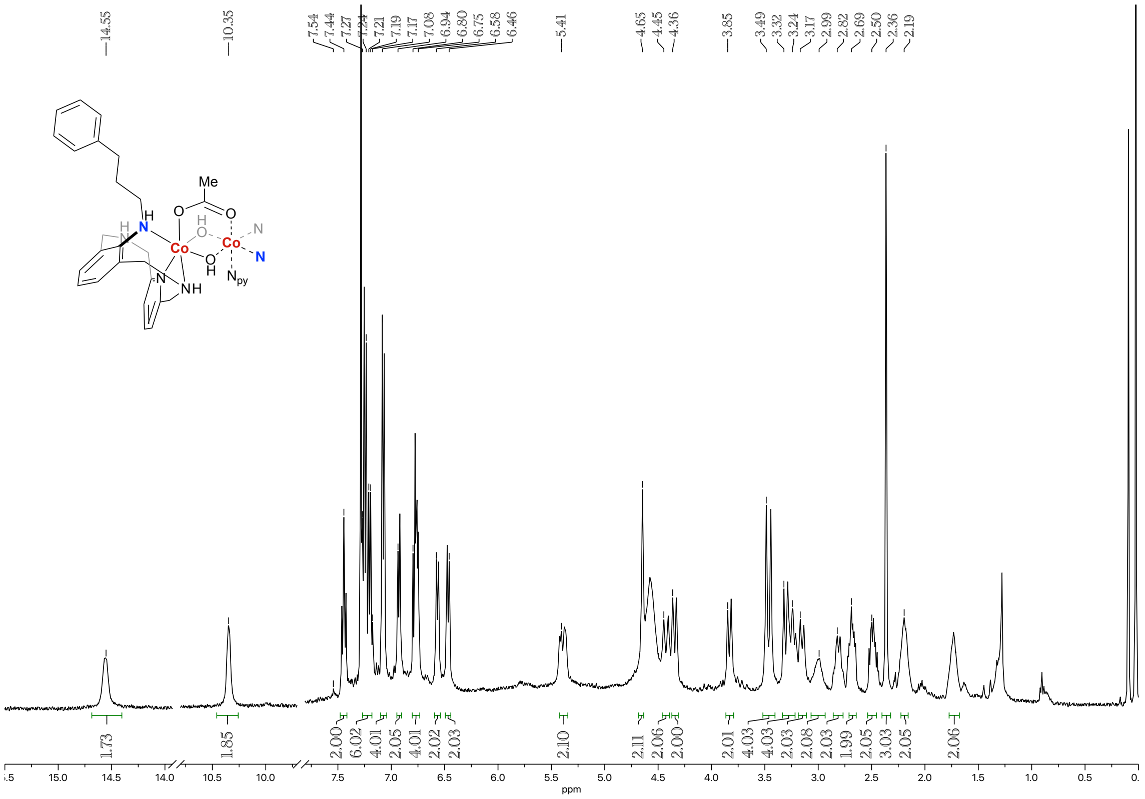

Figure S64. 400 MHz, ^{1}H NMR spectrum of **3c-OAc** in $CDCl_3$, 298 K.

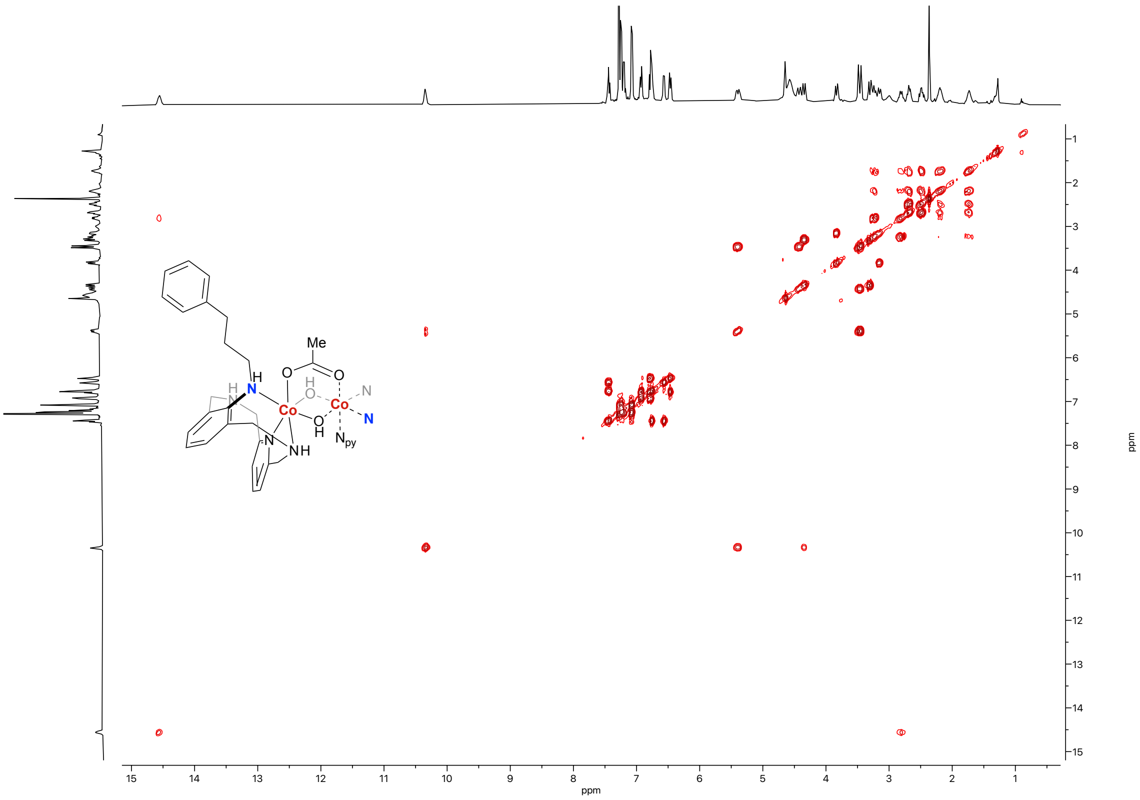

Figure S65. 400 MHz, ^{1}H-^{1}H COSY NMR spectrum of **3c-OAc** in $CDCl_3$, 298 K.

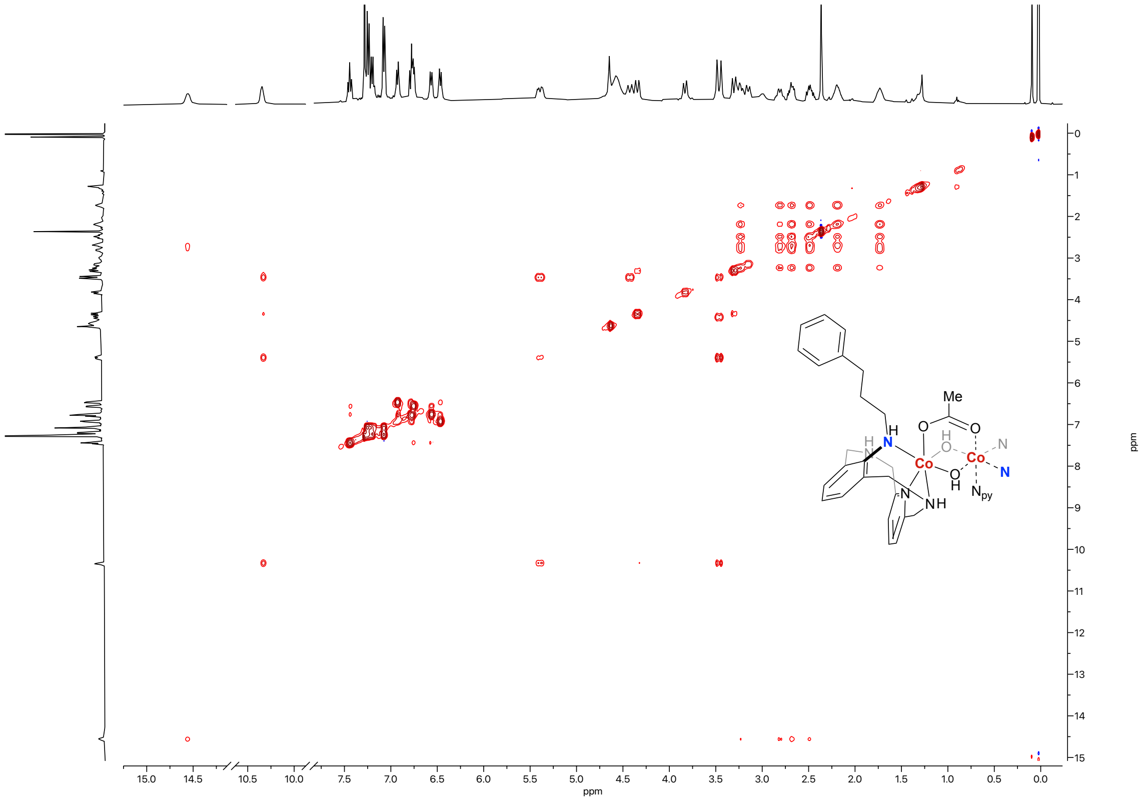

Figure S66. 400 MHz, ^1H-^1H TOCSY NMR spectrum of **3c-OAc** in $CDCl_3$, 298 K.

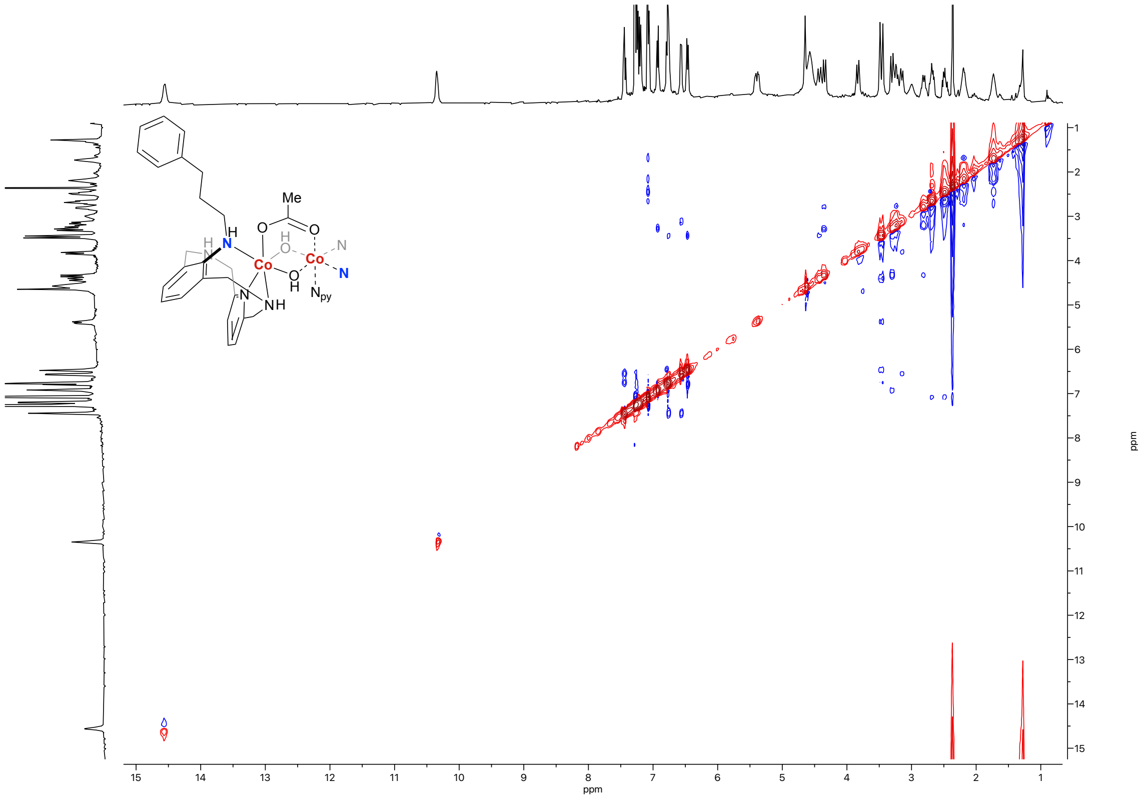

Figure S67. 400 MHz, ^1H-^1H NOESY NMR spectrum of **3c-OAc** in $CDCl_3$, 298 K.

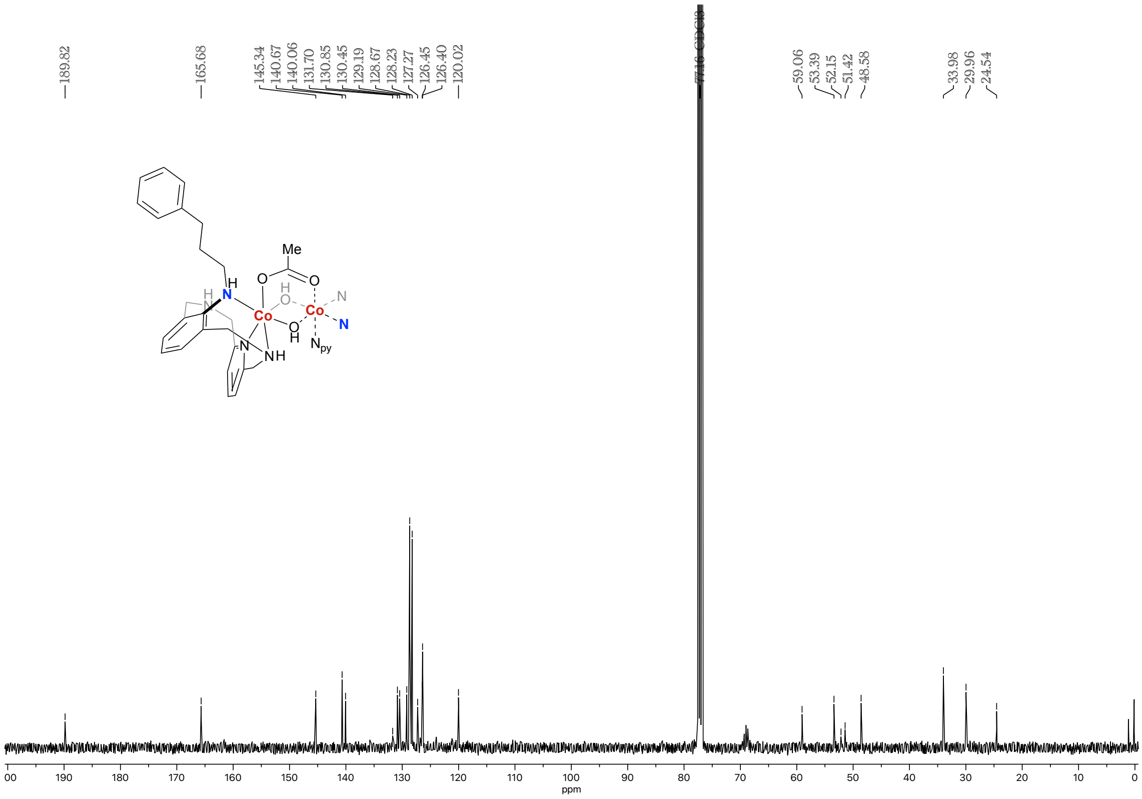

Figure S68. 100 MHz, $^{13}C\{^{1}H\}$ NMR spectrum of **3c-OAc** in $CDCl_3$, 298 K.

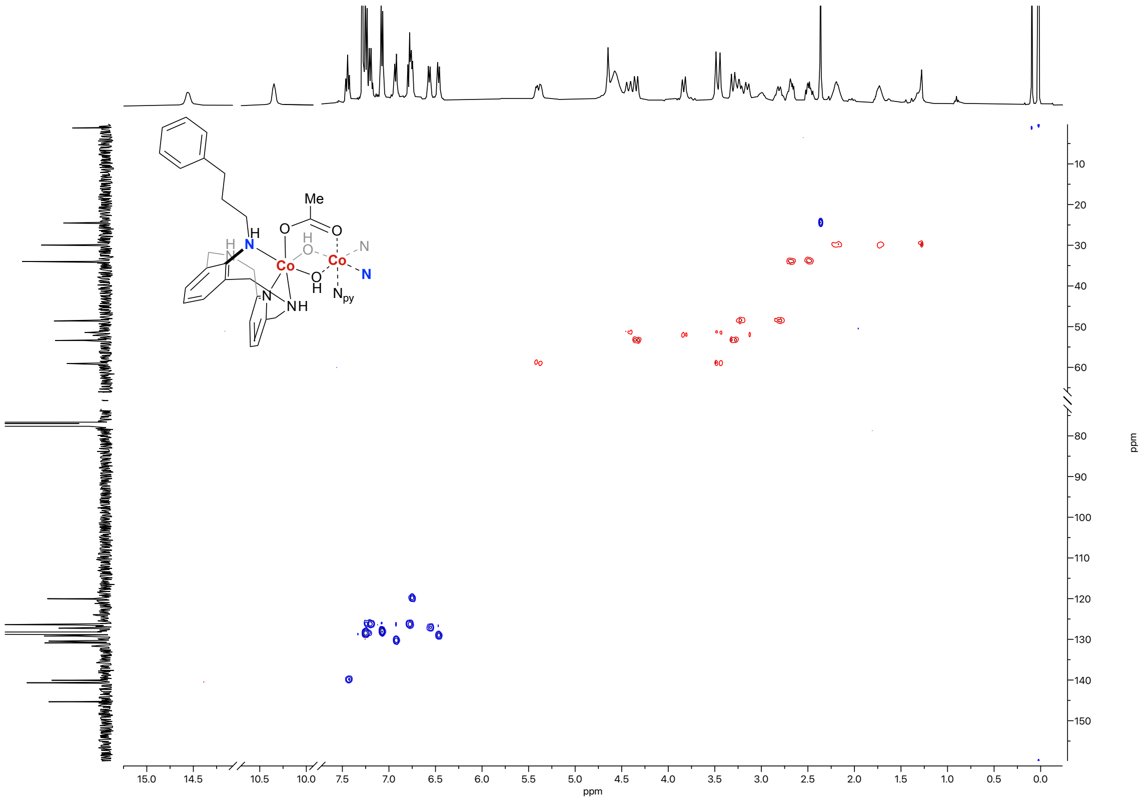

Figure S69. 400 MHz, ^{1}H-^{13}C HSQC NMR spectrum of **3c-OAc** in $CDCl_3$, 298 K.

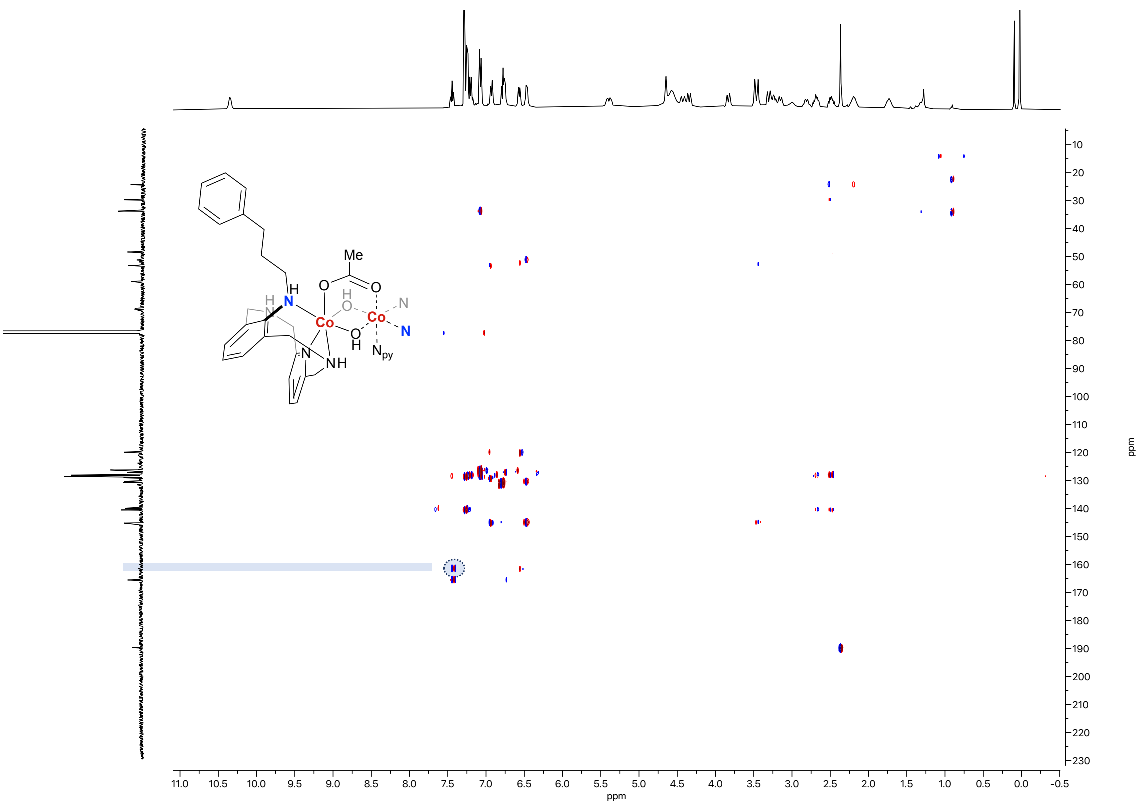

Figure S70. 400 MHz, ^{1}H-^{13}C HMBC NMR spectrum of **3c-OAc** in $CDCl_3$, 298 K.

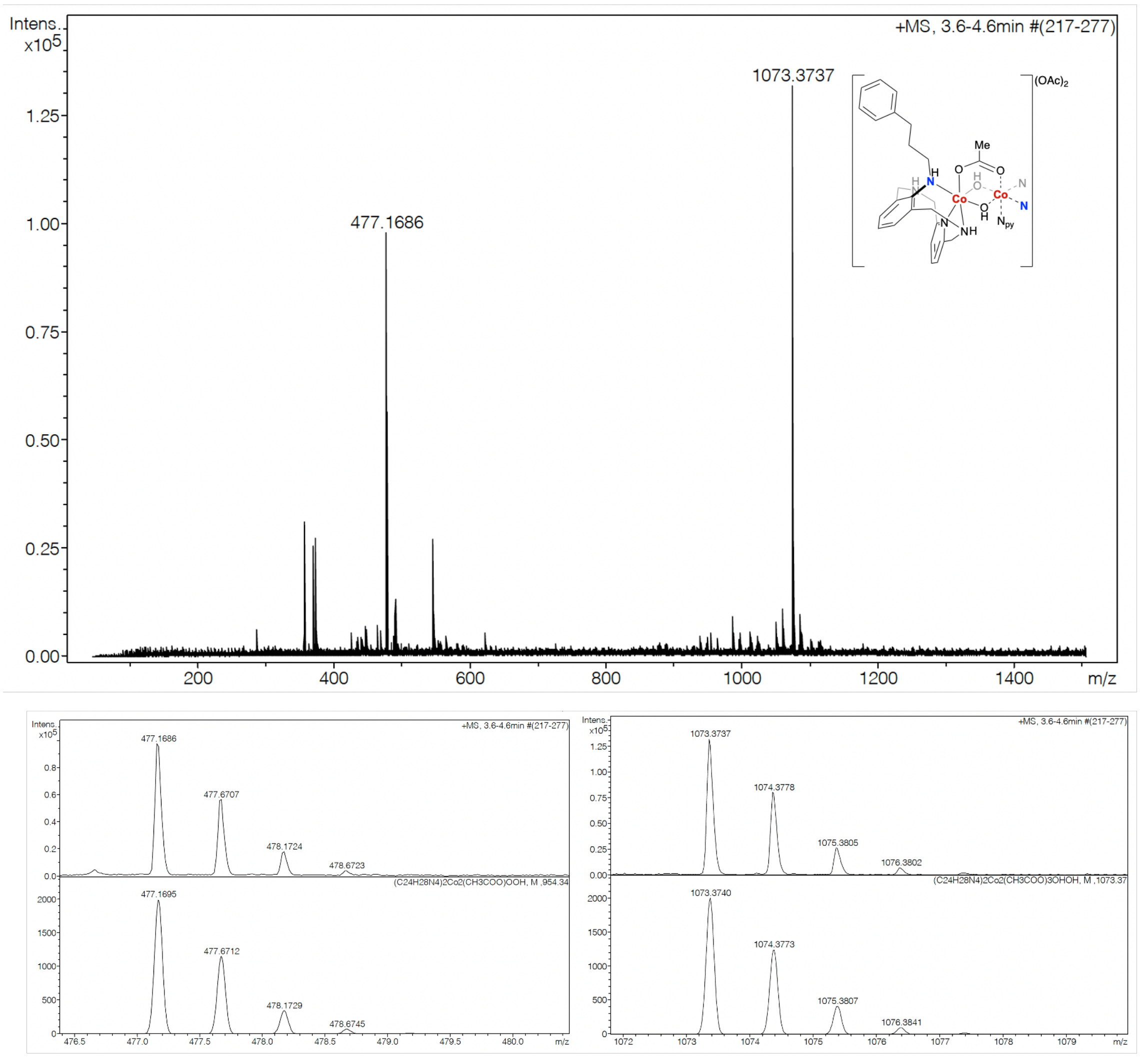

Figure S71. HRMS spectrum of **3c-OAc** showing the main peak at m/z = 1073.3737.

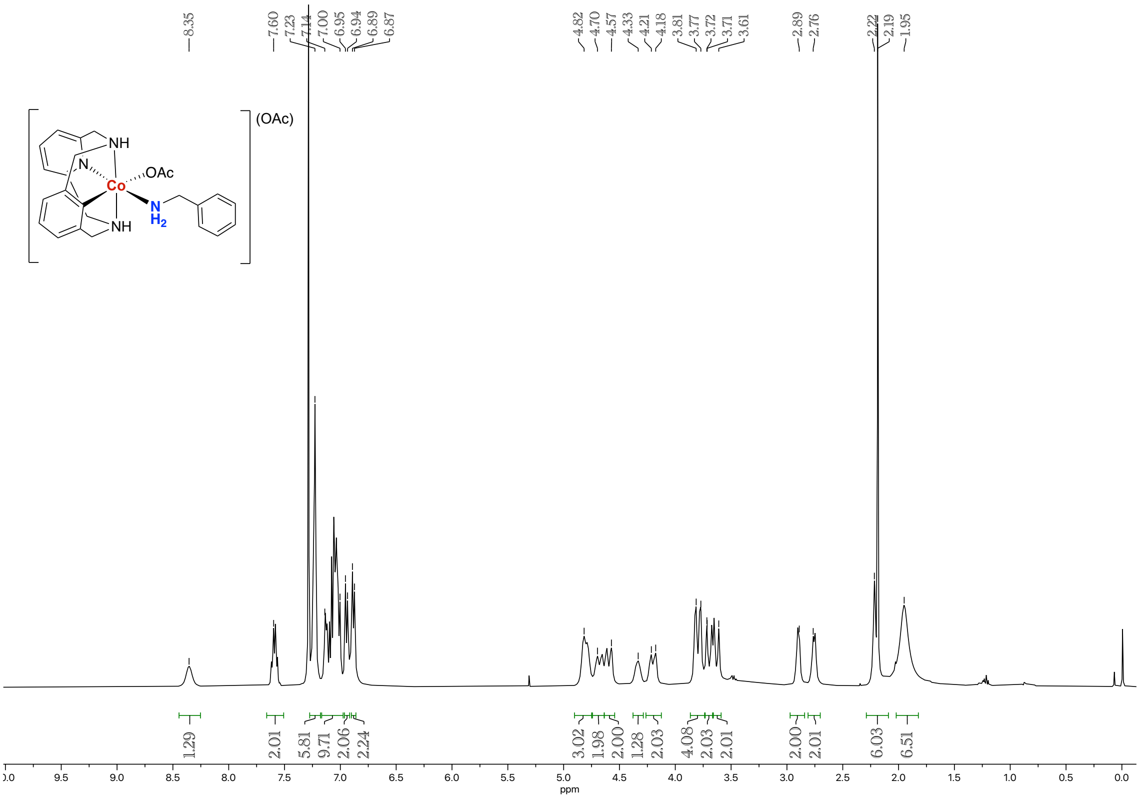

Figure S72. 400 MHz, ^{1}H NMR spectrum of **5-OAc** in $CDCl_3$, 273 K.

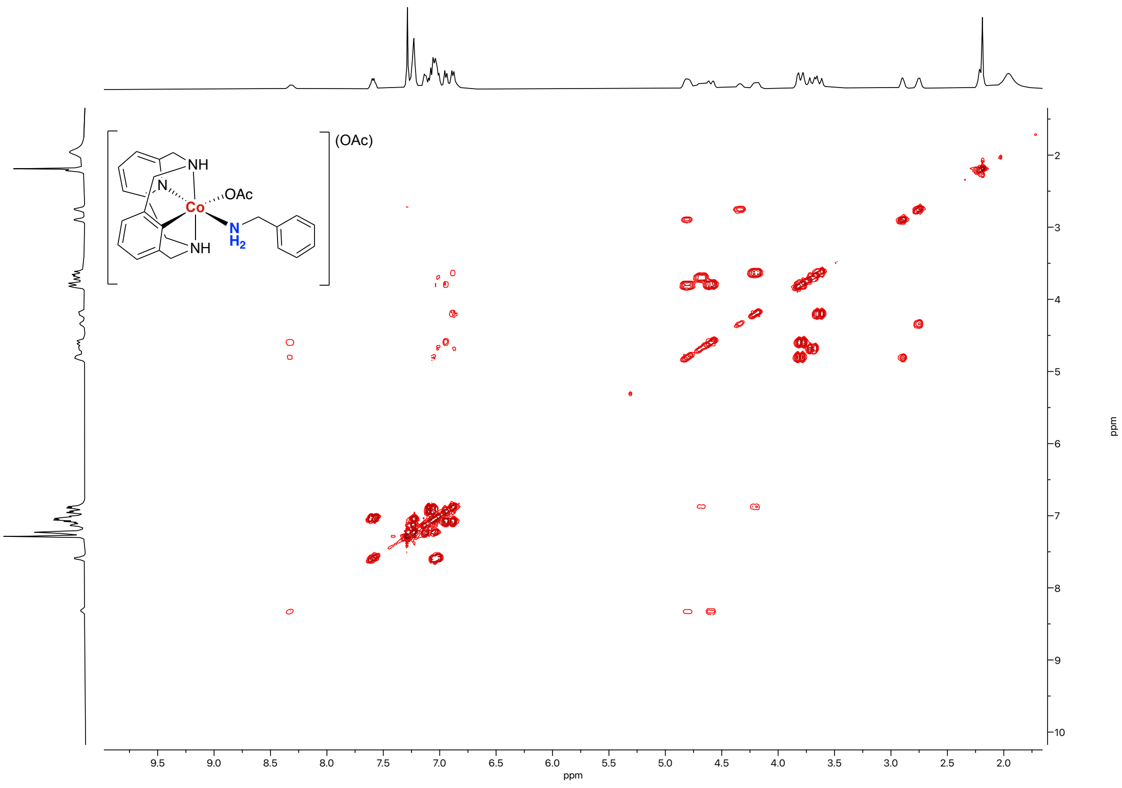

Figure S73. 400 MHz, ^{1}H-^{1}H COSY NMR spectrum of **5-OAc** in $CDCl_3$, 273 K.

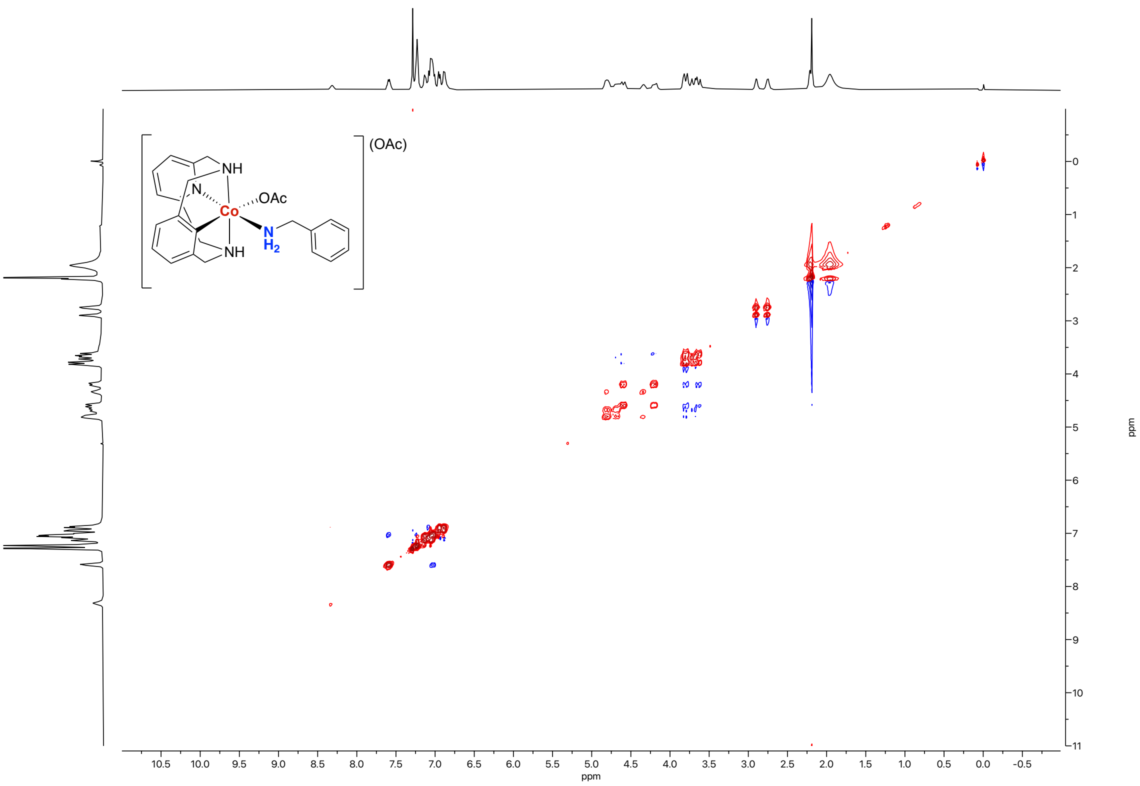

Figure S74. 400 MHz, ^{1}H-^{1}H NOESY NMR spectrum of **5-OAc** in $CDCl_3$, 273 K.

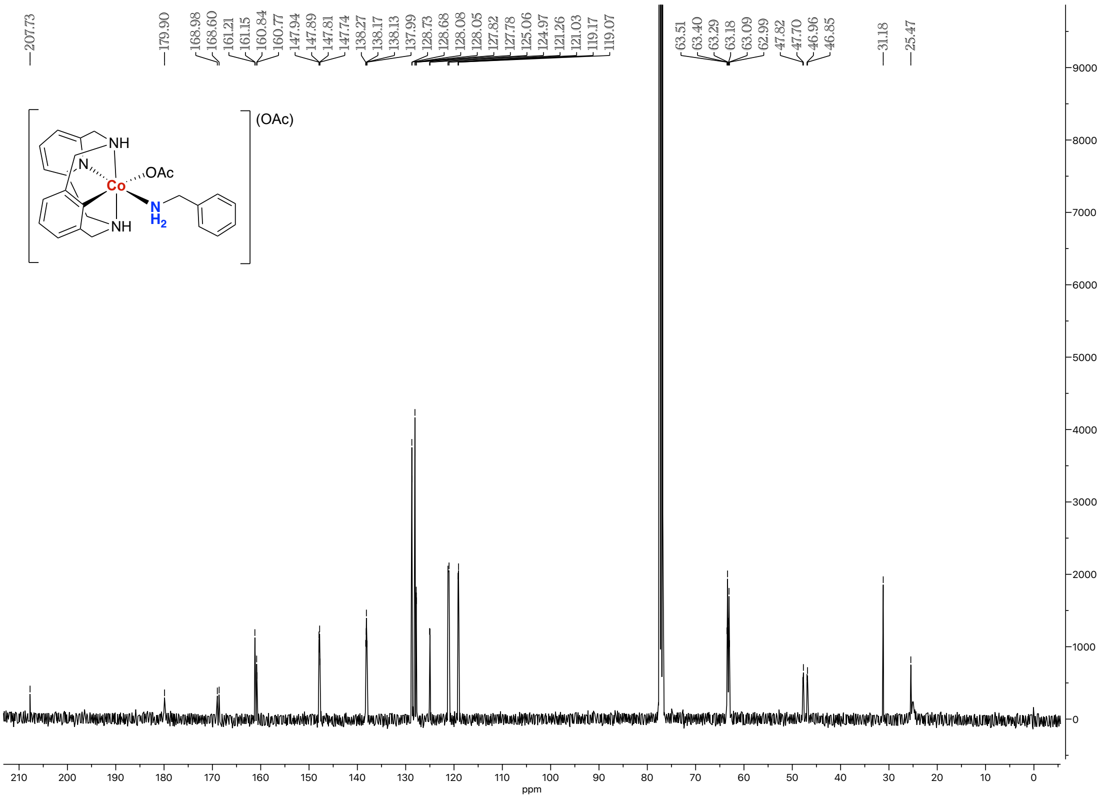

Figure S75. 100 MHz, $^{13}C\{^{1}H\}$ NMR spectrum of **5-OAc** in $CDCl_3$, 273 K

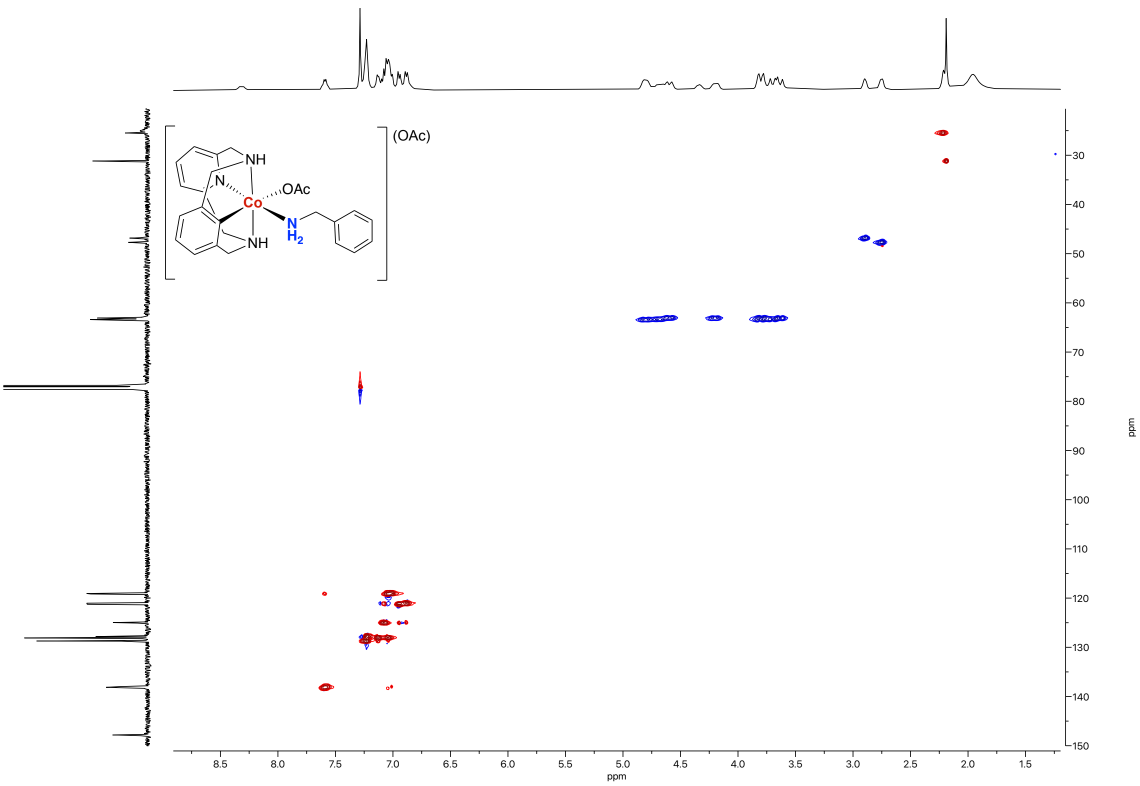

Figure S76. 400 MHz, ^{1}H-^{13}C HSQC NMR spectrum of **5-OAc** in $CDCl_3$, 273 K.

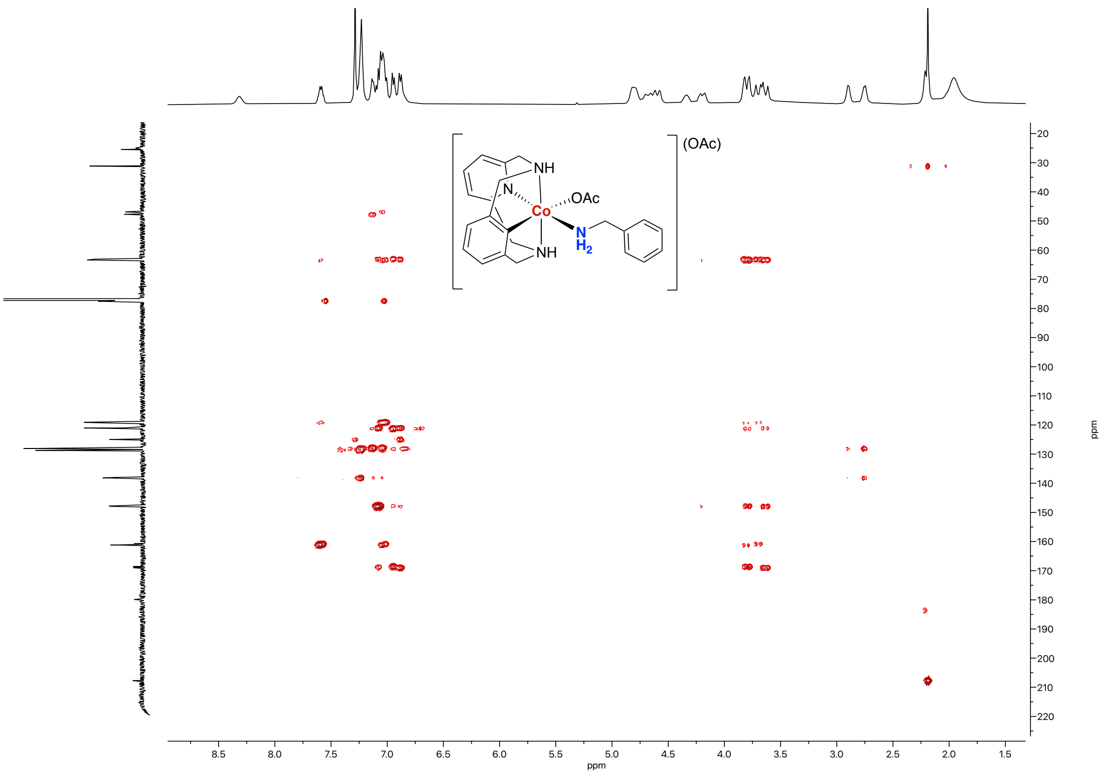

Figure S77. 400 MHz, ^{1}H-^{13}C HMBC NMR spectrum of **5-OAc** in $CDCl_3$, 273 K

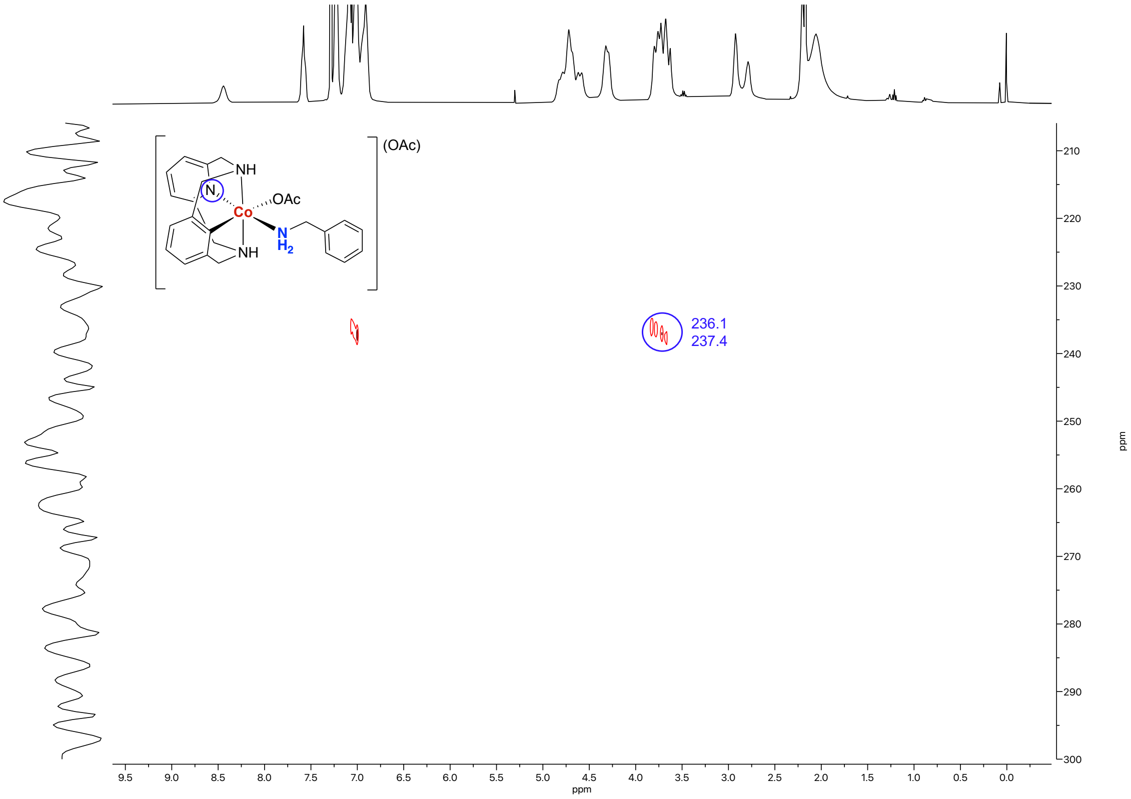

Figure S78. ^{1}H-^{15}N HMBC NMR spectrum of **5-OAc** in $CDCl_3$, 273 K.

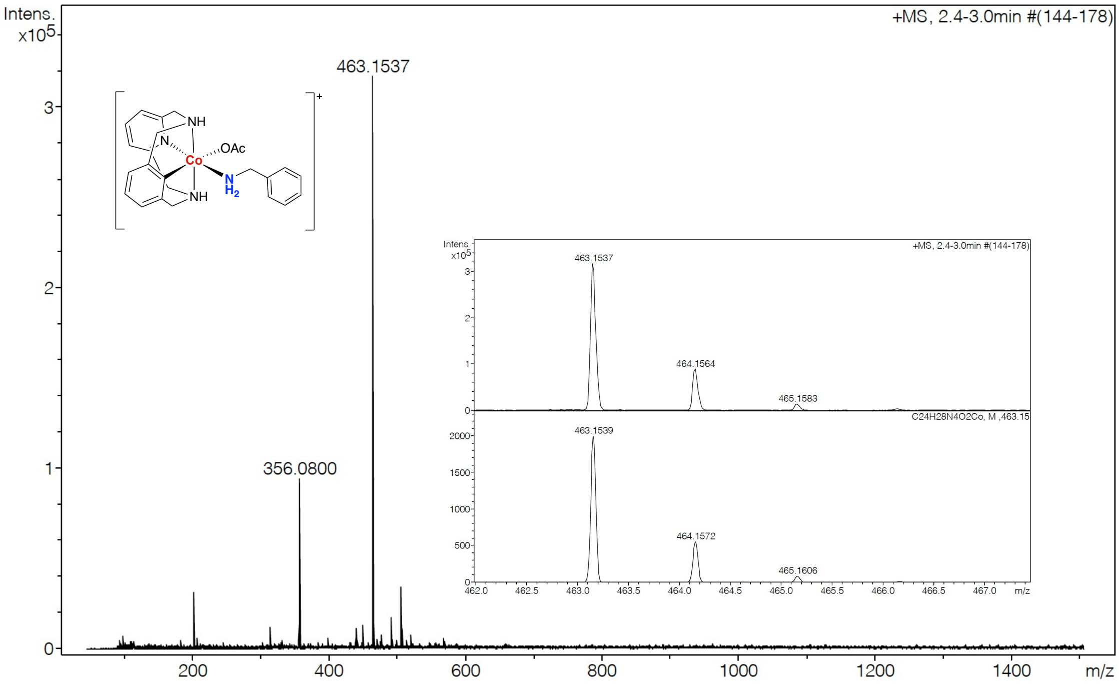

Figure S79. HRMS spectrum of **5-OAc** showing a peak at $m/z = 463.1537$. Inset: up, experimental; down, simulated.

12.2 Rh complexes

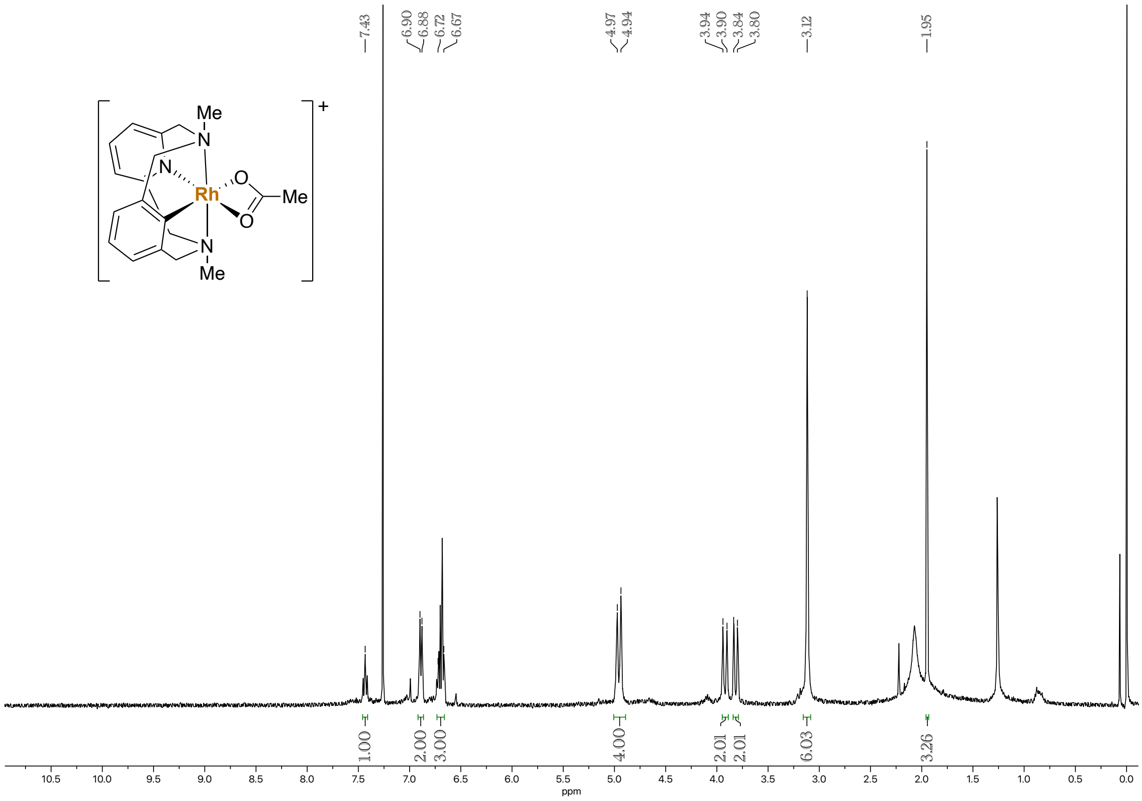

Figure S80. 400 MHz, ^{1}H NMR spectrum of $\mathbf{6_{Me}}$**-OAc** in $CDCl_3$, 298 K.

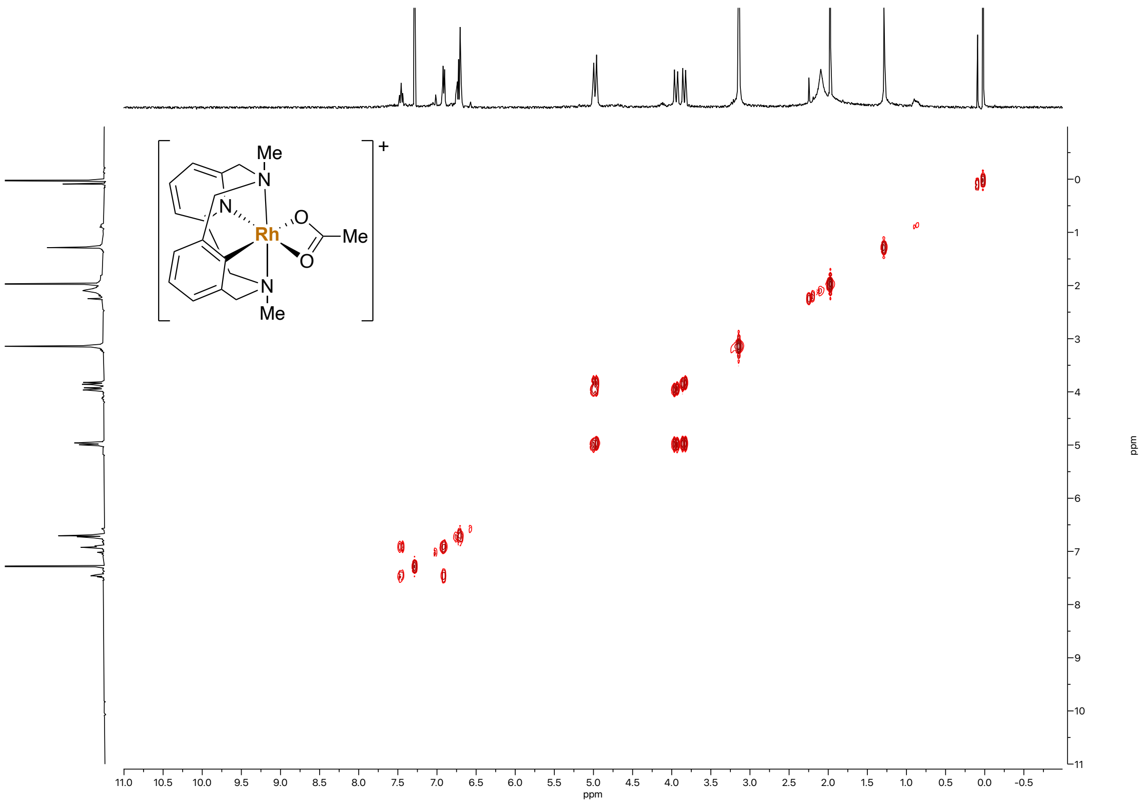

Figure S81. 400 MHz, ^{1}H-^{1}H COSY NMR spectrum of $\mathbf{6_{Me}}$**-OAc** in $CDCl_3$, 298 K.

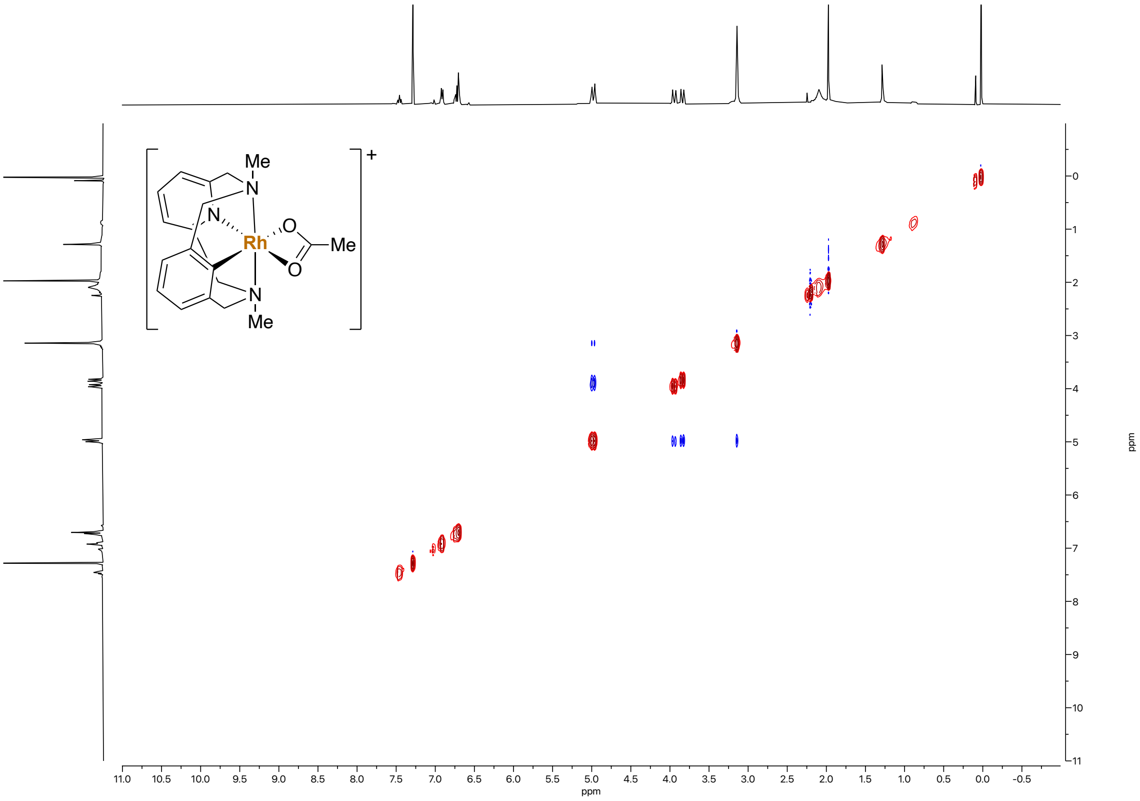

Figure S82. 400 MHz, ^{1}H-^{1}H NOESY NMR spectrum of **6$_{Me}$-OAc** in $CDCl_3$, 298 K.

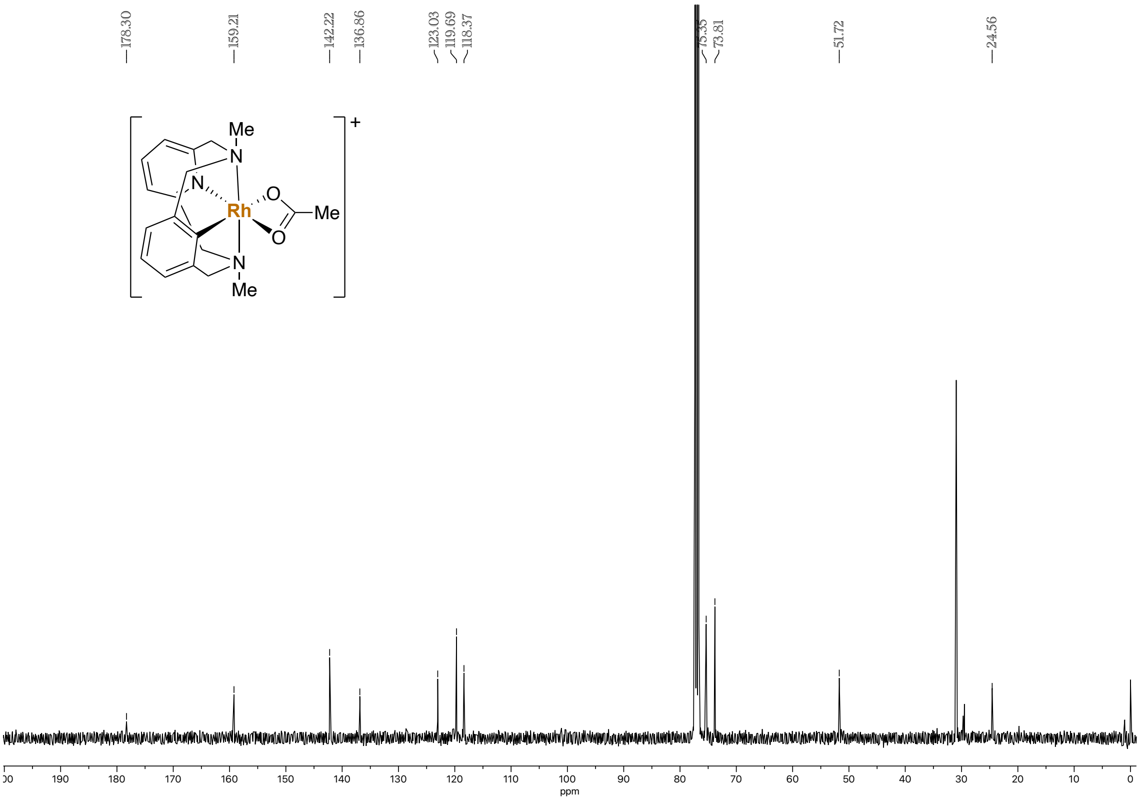

Figure S83. 100 MHz, ^{13}C {^{1}H} NMR spectrum of **6$_{Me}$-OAc** in $CDCl_3$, 298 K.

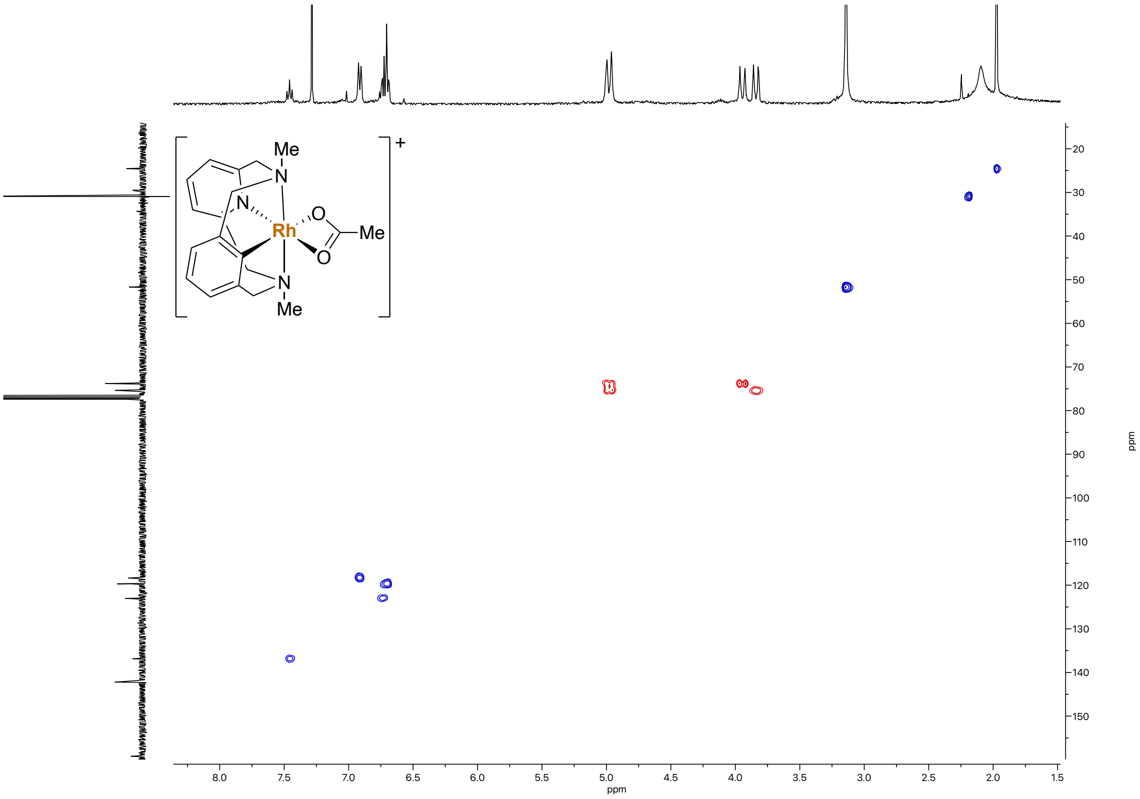

Figure S84. 400 MHz, ^{1}H-^{13}C HSQC NMR spectrum of **6$_{Me}$-OAc** in $CDCl_3$, 298 K.

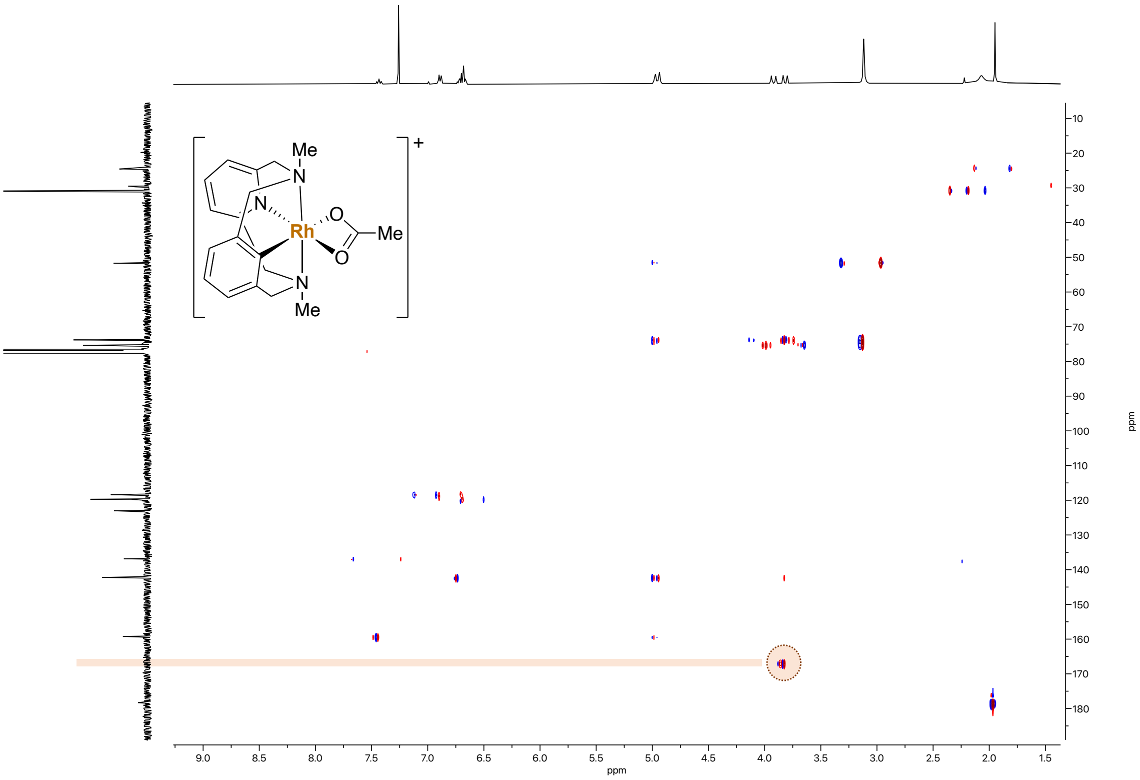

Figure S85. 400 MHz, ^{1}H-^{13}C HMBC NMR spectrum of **6$_{Me}$-OAc** in $CDCl_3$, 298 K.

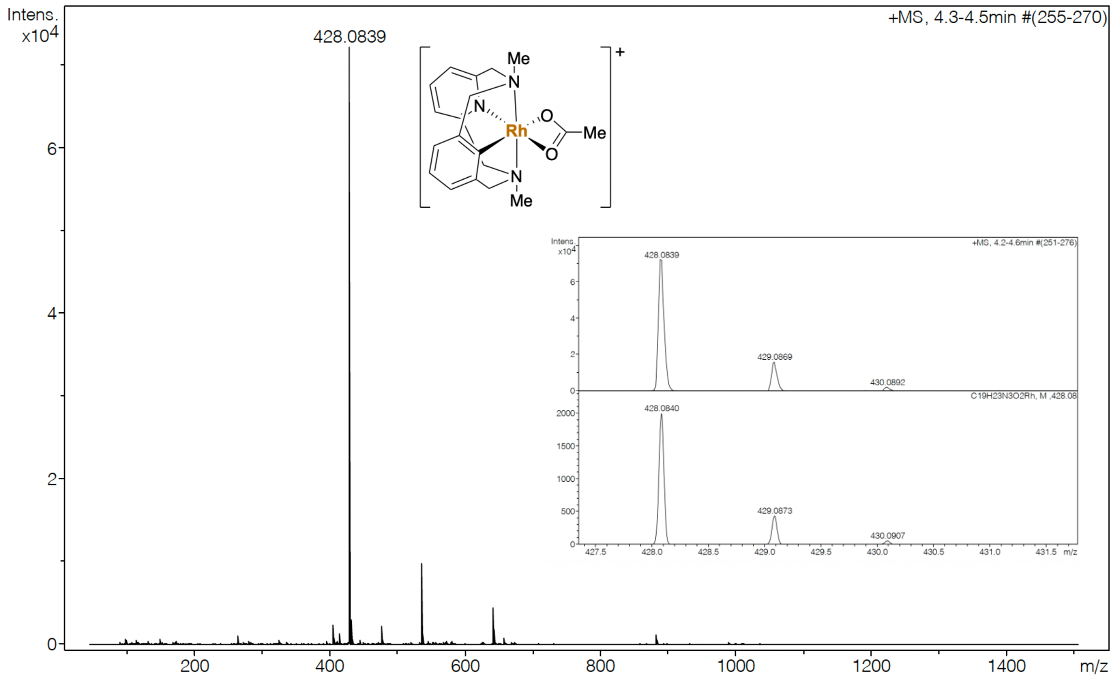

Figure S86. HRMS spectrum of **6$_{Me}$-OAc** showing a peak at m/z = 428.0839. Inset: up, experimental; down, simulated spectrum.

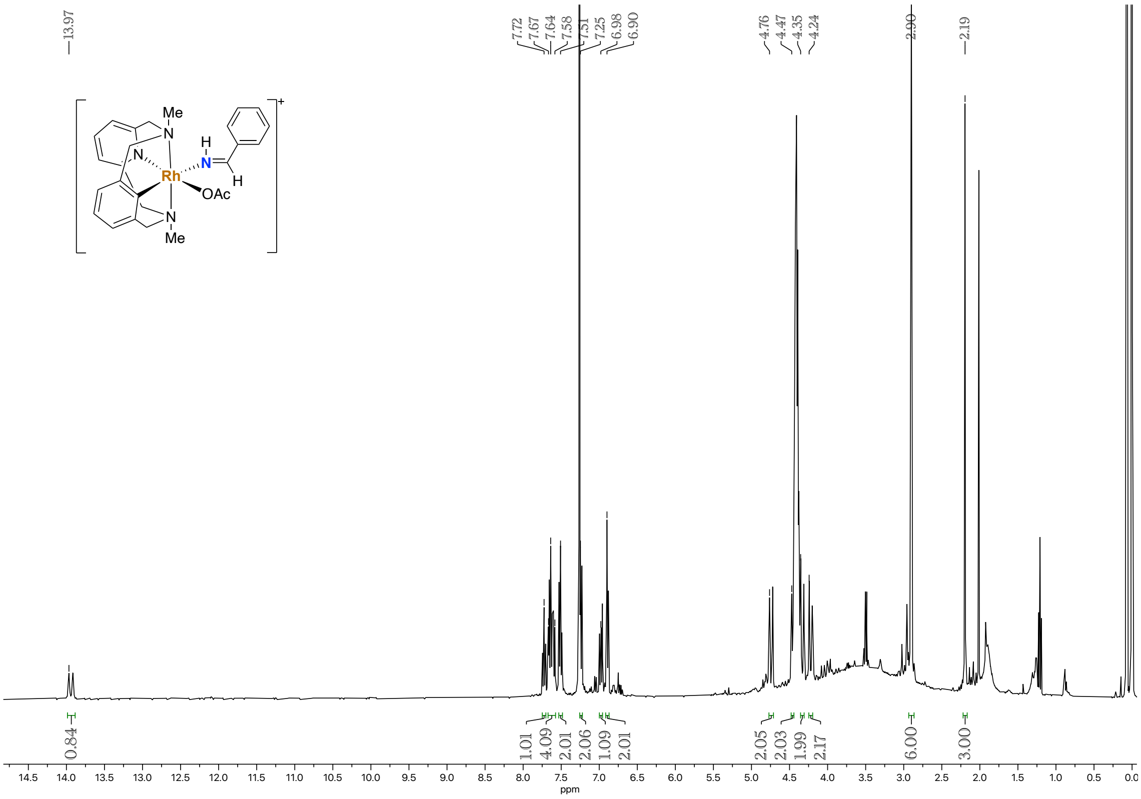

Figure S87. 400 MHz, ^{1}H NMR spectrum of **7a$_{Me}$-OAc** in $CDCl_3$, 298 K.

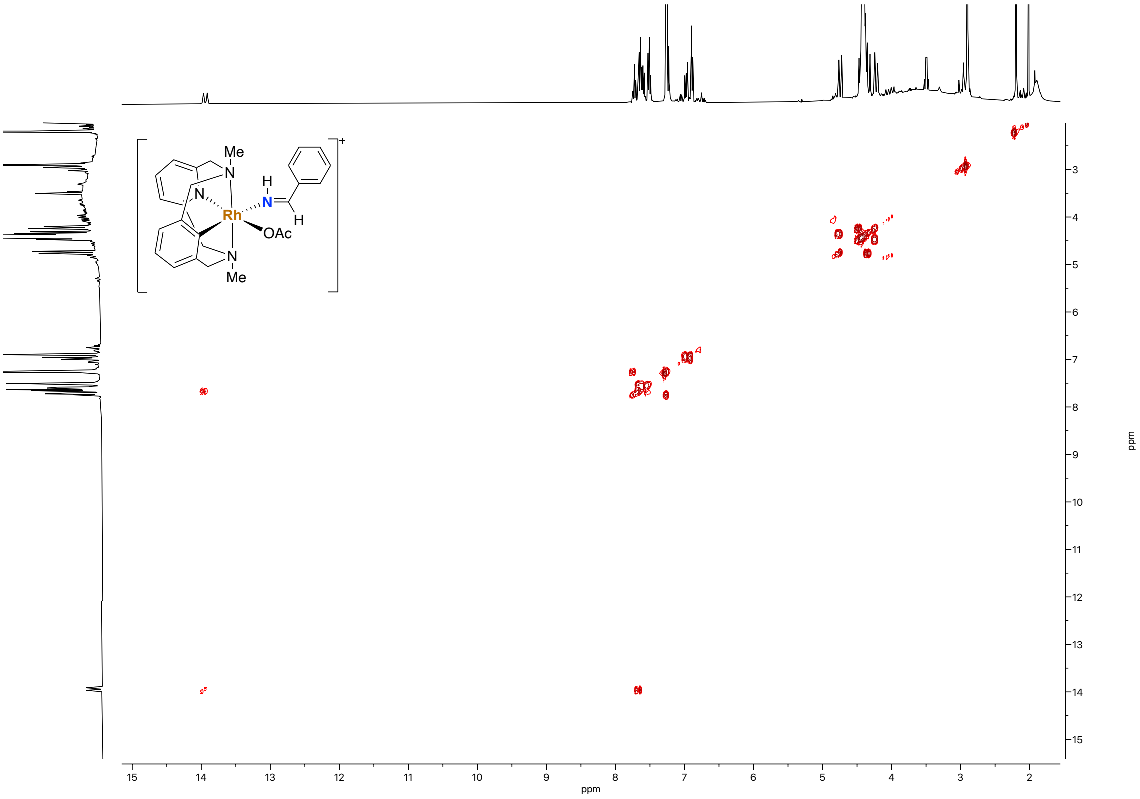

Figure S88. 400 MHz, ^{1}H-^{1}H COSY NMR spectrum of **7a$_{Me}$-OAc** in $CDCl_3$, 298 K.

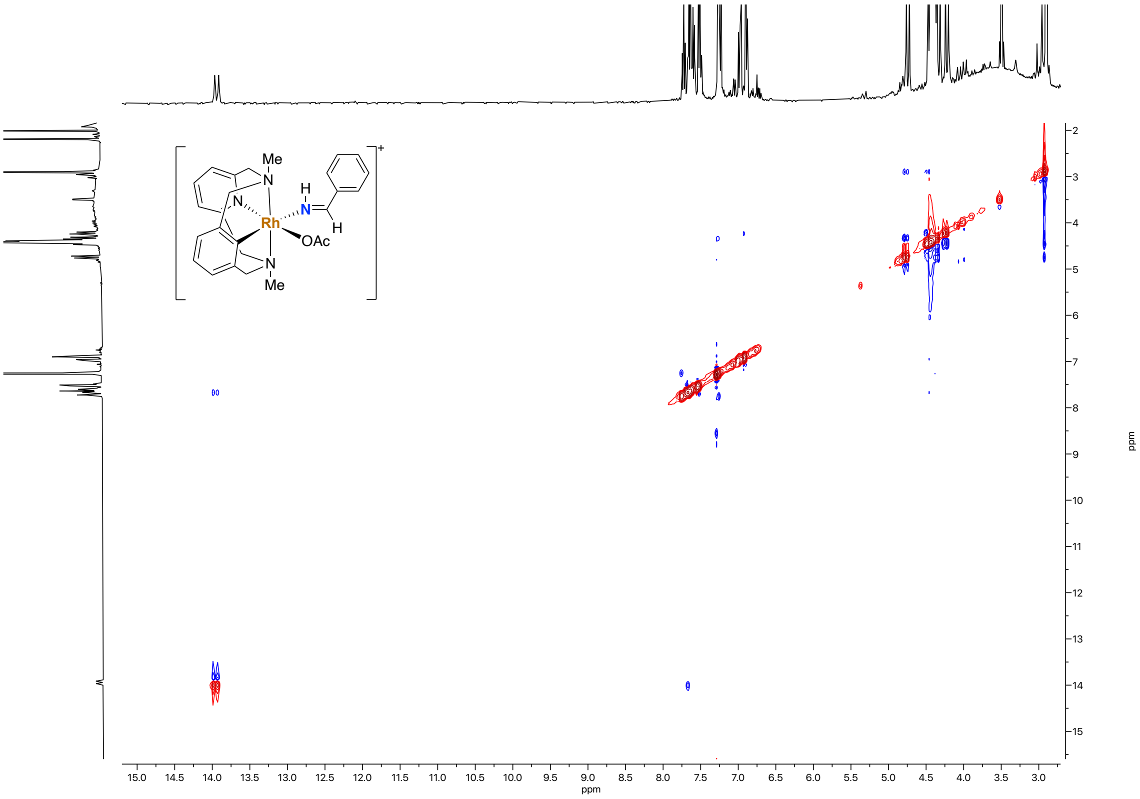

Figure S89. 400 MHz, ^{1}H-^{1}H. NOESY NMR spectrum of **7a$_{Me}$-OAc** in $CDCl_3$, 298 K.

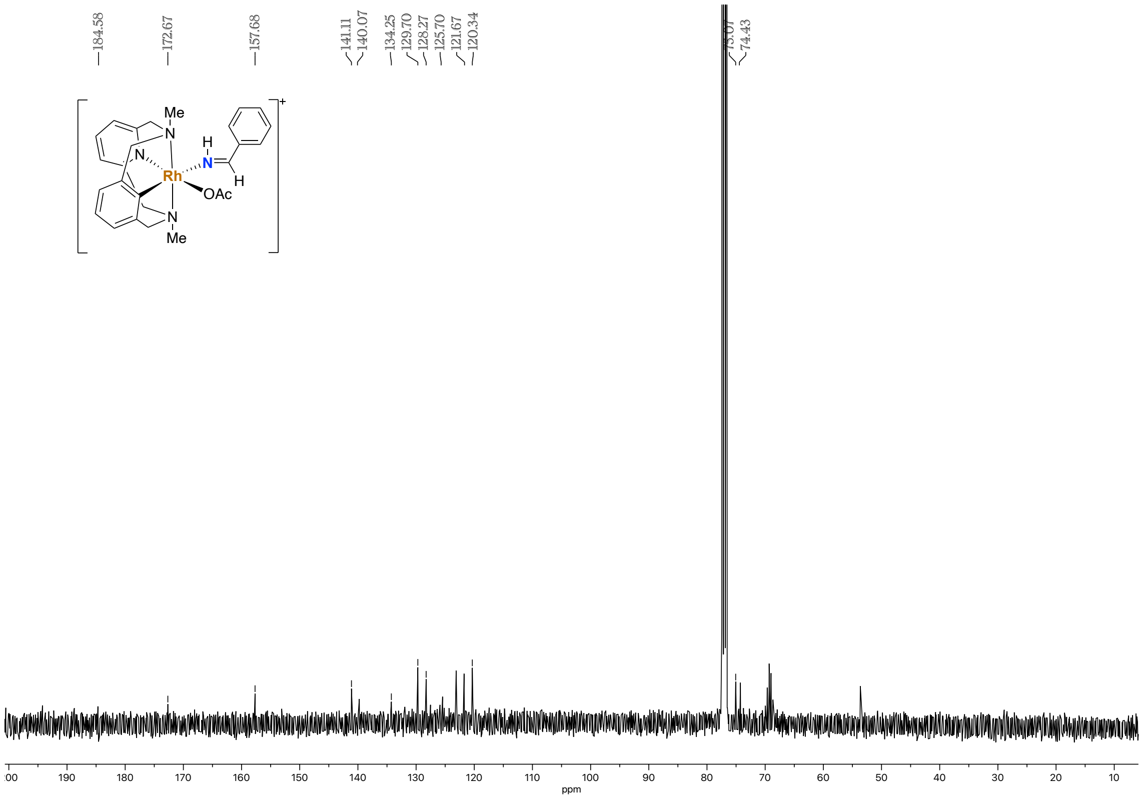

Figure S90. 100 MHz, ^{13}C NMR spectrum of **7a$_{Me}$-OAc** in $CDCl_3$, 298 K.

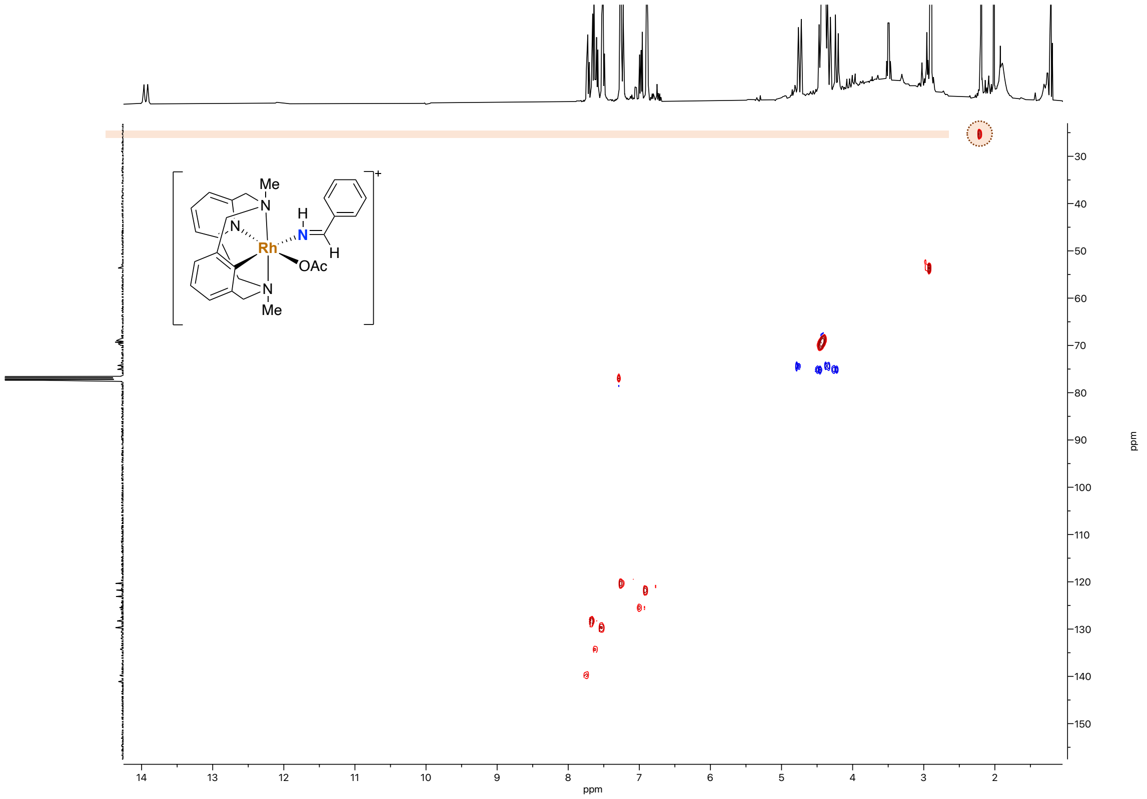

Figure S91. 400 MHz, ^1H-^{13}C HSQC NMR spectrum of **7a$_{Me}$-OAc** in $CDCl_3$, 298 K.

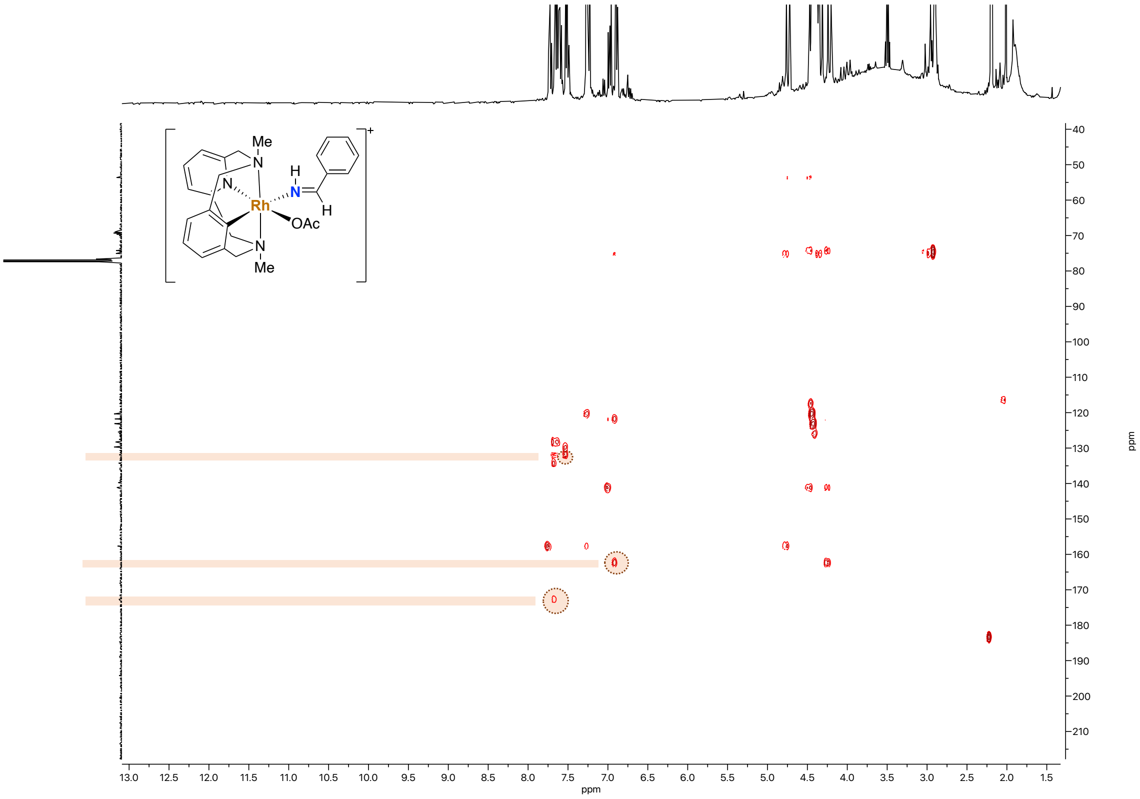

Figure S92. 400 MHz, ^{1}H-^{13}C HMBC NMR spectrum of **7a$_{Me}$-OAc** in $CDCl_3$, 298 K.

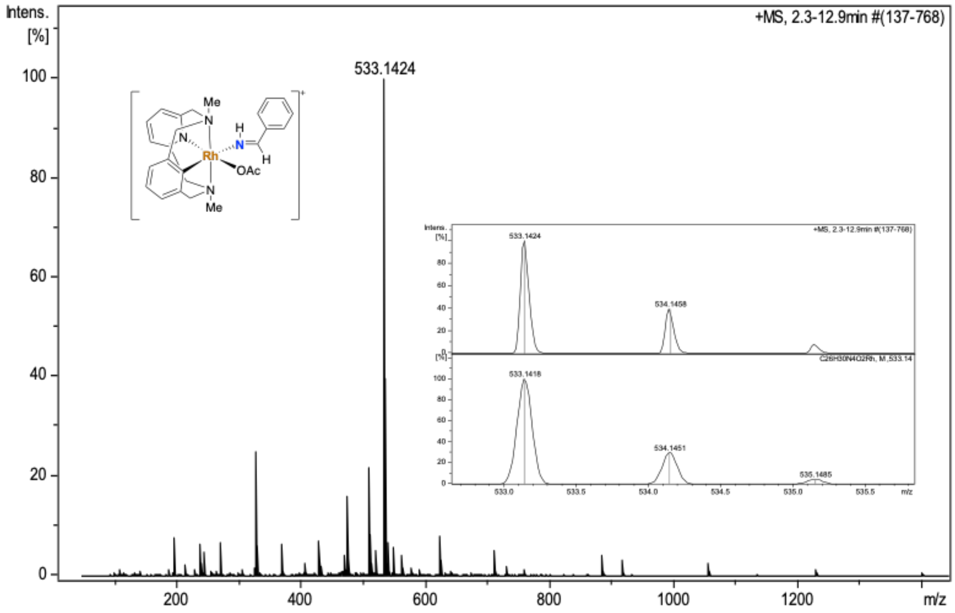

Figure S93. HRMS spectrum of **7a$_{Me}$-OAc** showing a peak at m/z = 533.1424. Inset: up, experimental; down, simulated.

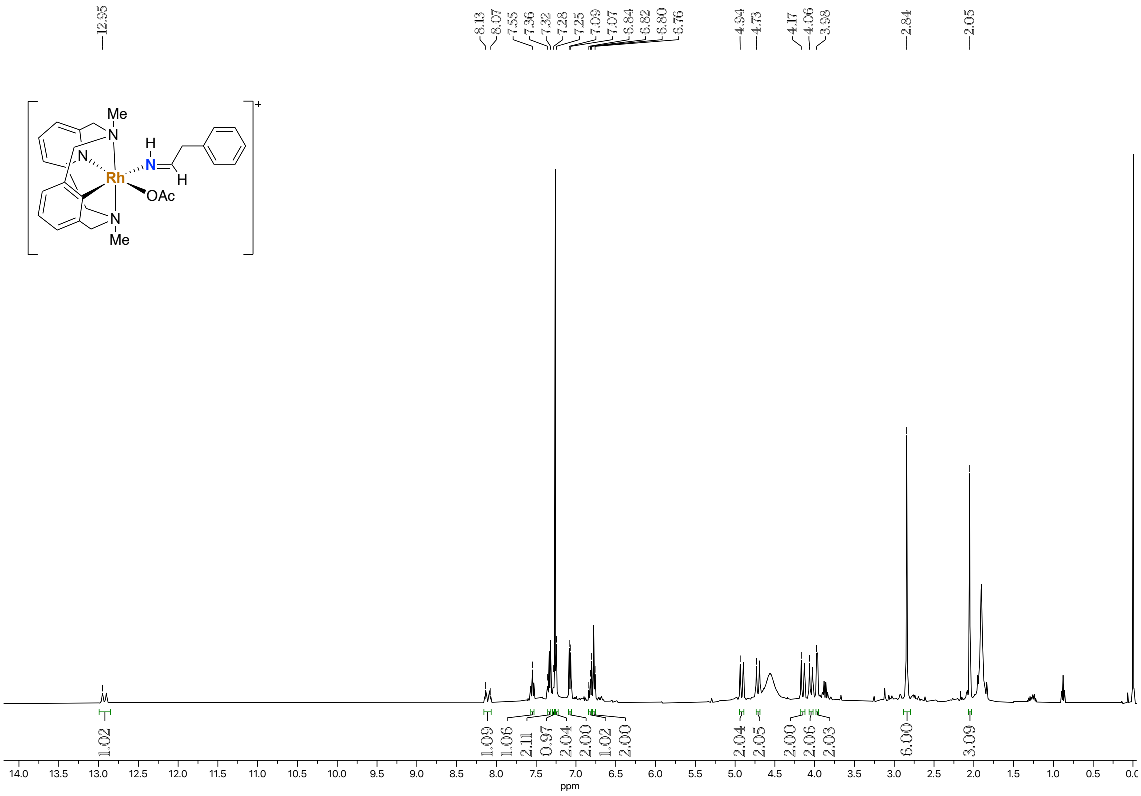

Figure S94. 400 MHz, ^{1}H NMR spectrum of **7b$_{Me}$-OAc** in $CDCl_3$, 298 K.

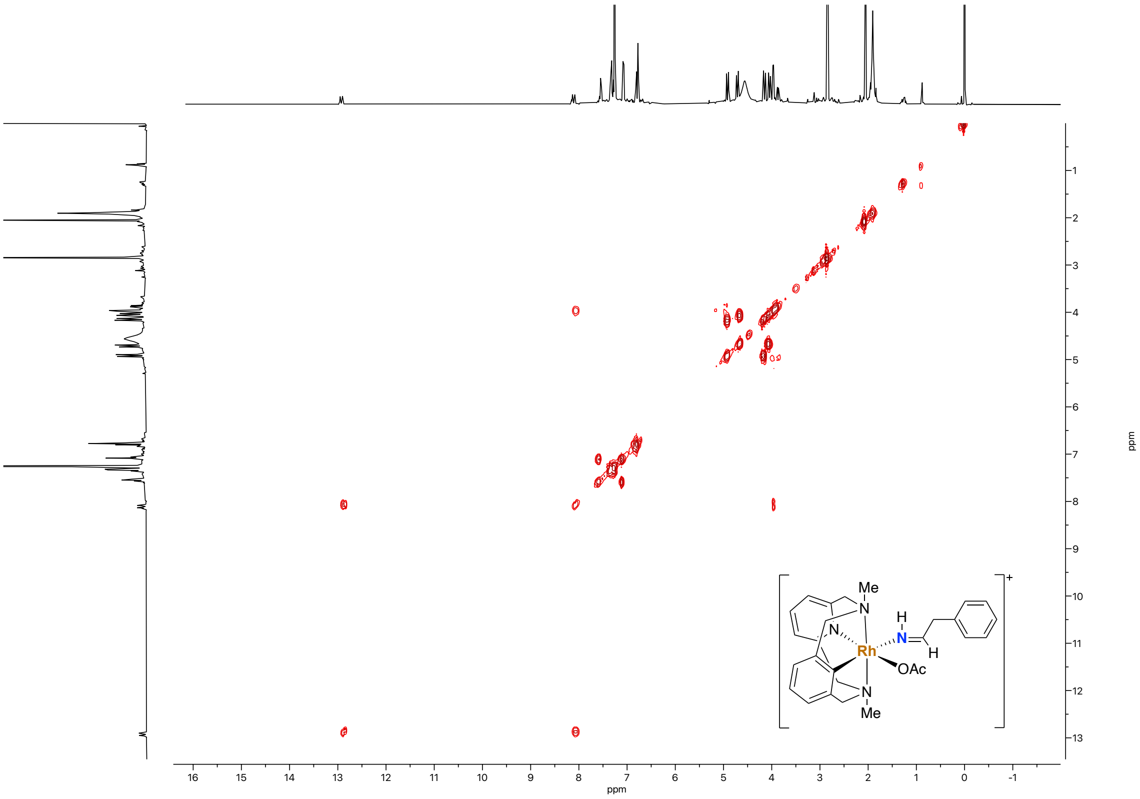

Figure S95. 400 MHz, ^{1}H-^{1}H COSY NMR spectrum of **7b$_{Me}$-OAc** in $CDCl_3$, 298 K.

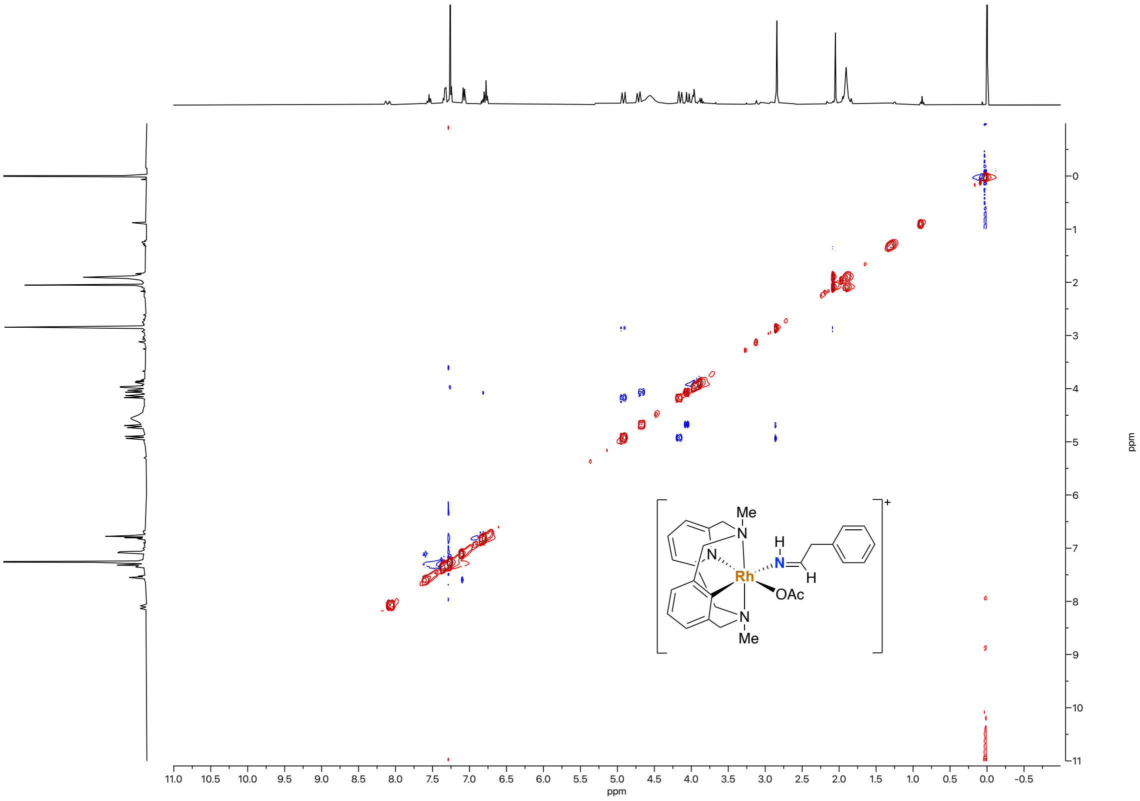

Figure S96. 400 MHz, ¹H-¹H NOESY NMR spectrum of **7b$_{Me}$-OAc** in $CDCl_3$, 298 K.

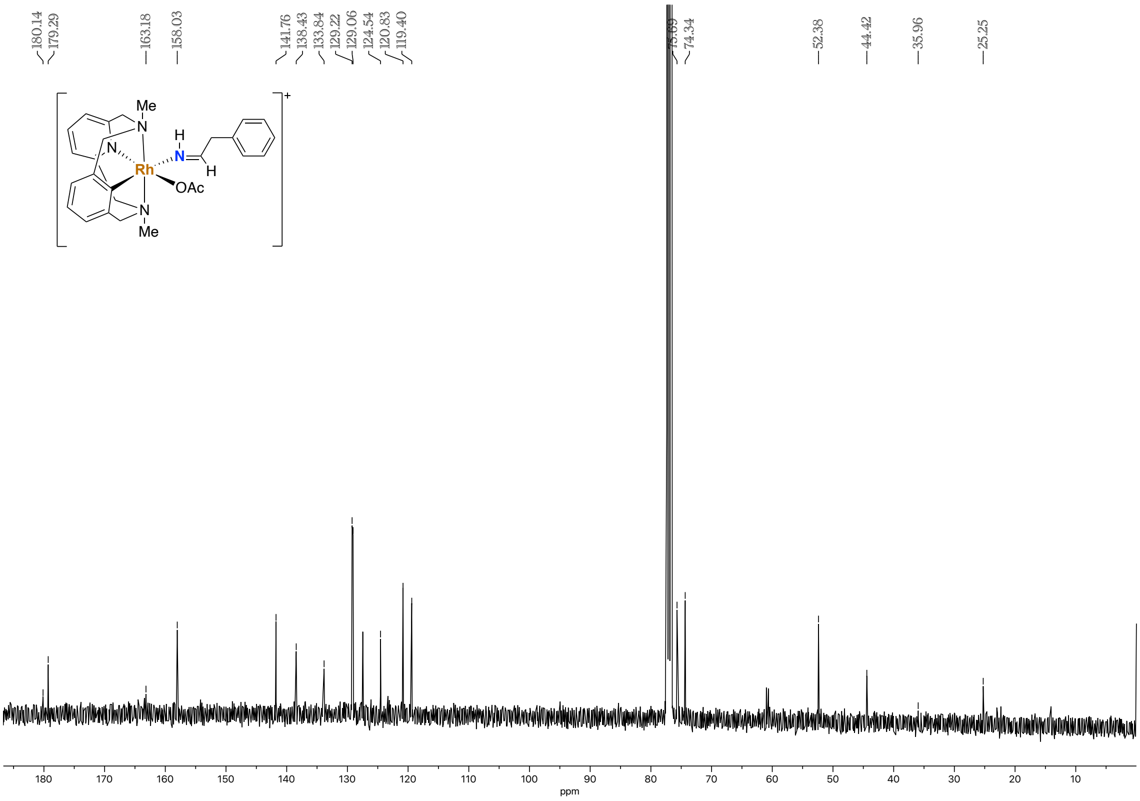

Figure S97. 100 MHz, ^{13}C NMR spectrum of **7b$_{Me}$-OAc** in $CDCl_3$, 298 K.

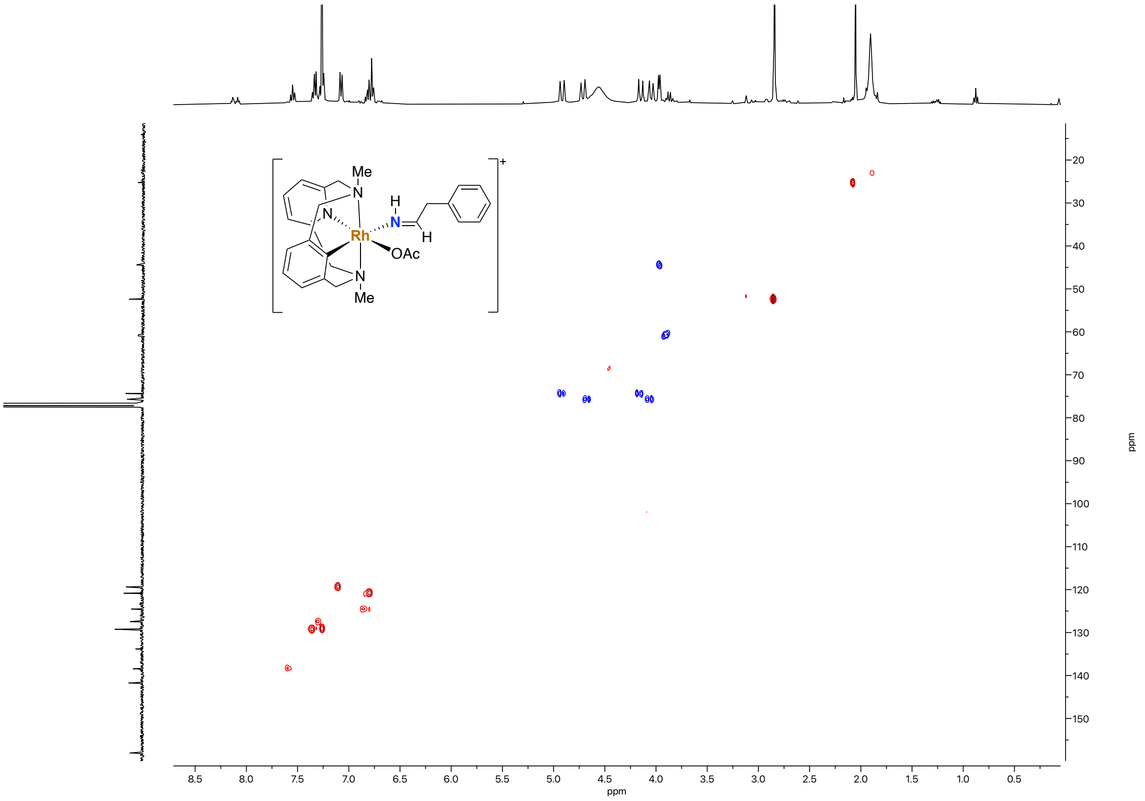

Figure S98. 400 MHz, ^{1}H-^{13}C HSQC NMR spectrum of **7b$_{Me}$-OAc** in $CDCl_3$, 298 K.

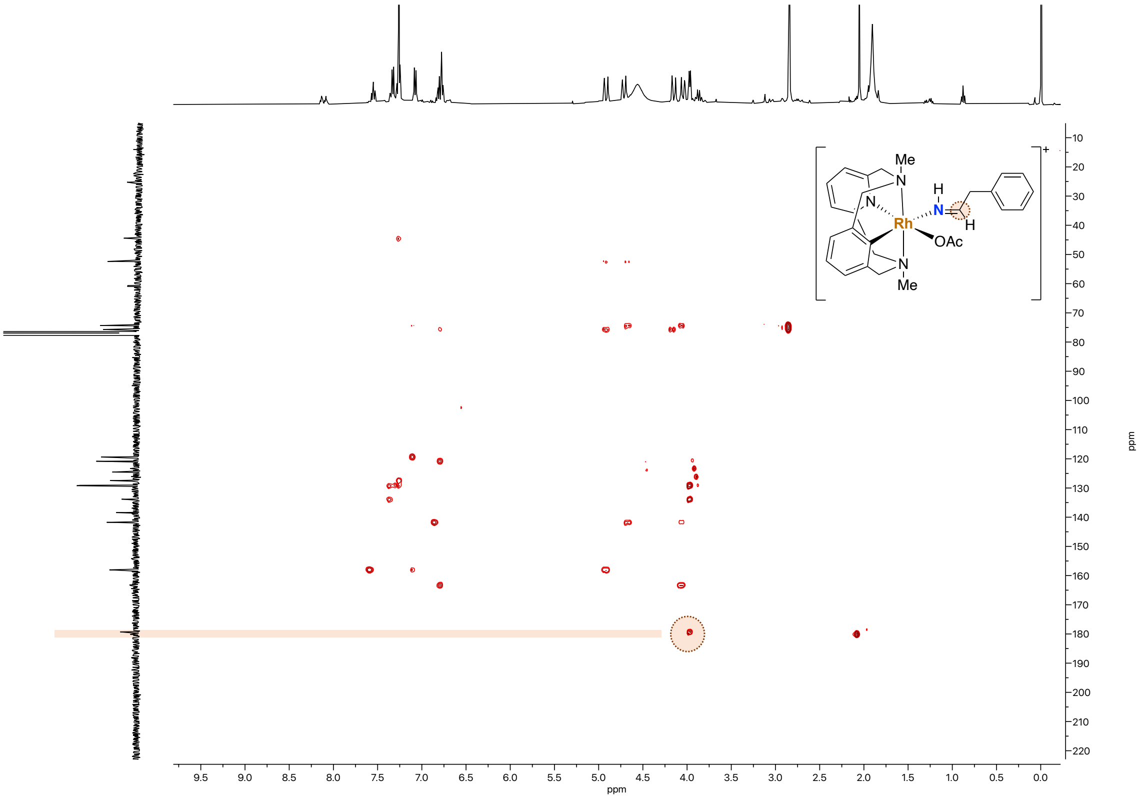

Figure S99. 400 MHz, ^{1}H-^{13}C HMBC NMR spectrum of **7b$_{Me}$-OAc** in $CDCl_3$, 298 K.

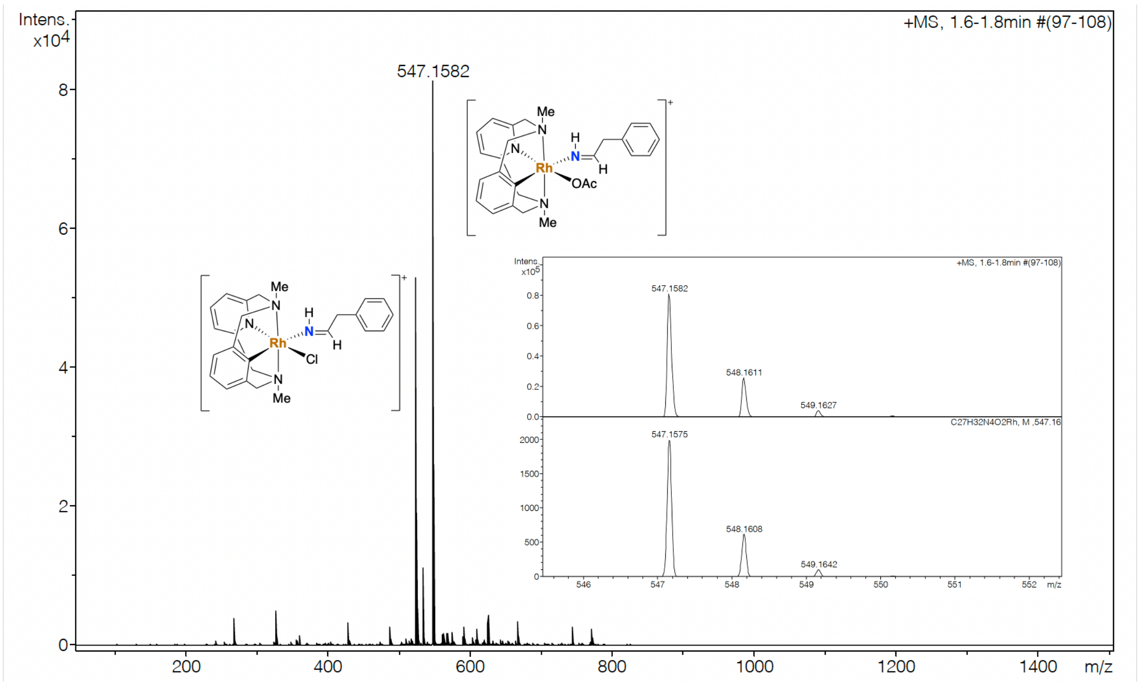

Figure S100. HRMS spectrum of **$7b_{Me}$-OAc** showing a peak at m/z = 547.1582. Inset: up, experimental spectrum; down, simulated spectrum.

12.3 Organic C-N coupling products

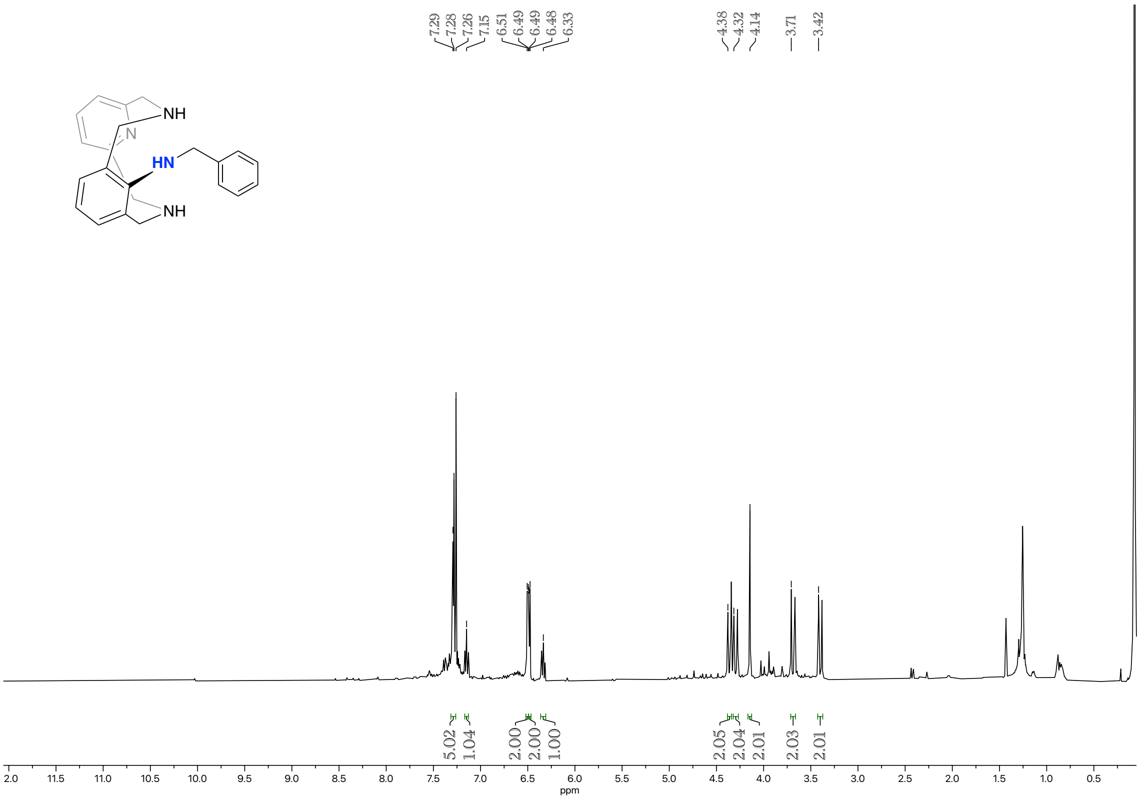

Figure S101. 400 MHz, ^{1}H NMR spectrum of **P-a** in $CDCl_3$, 298 K.

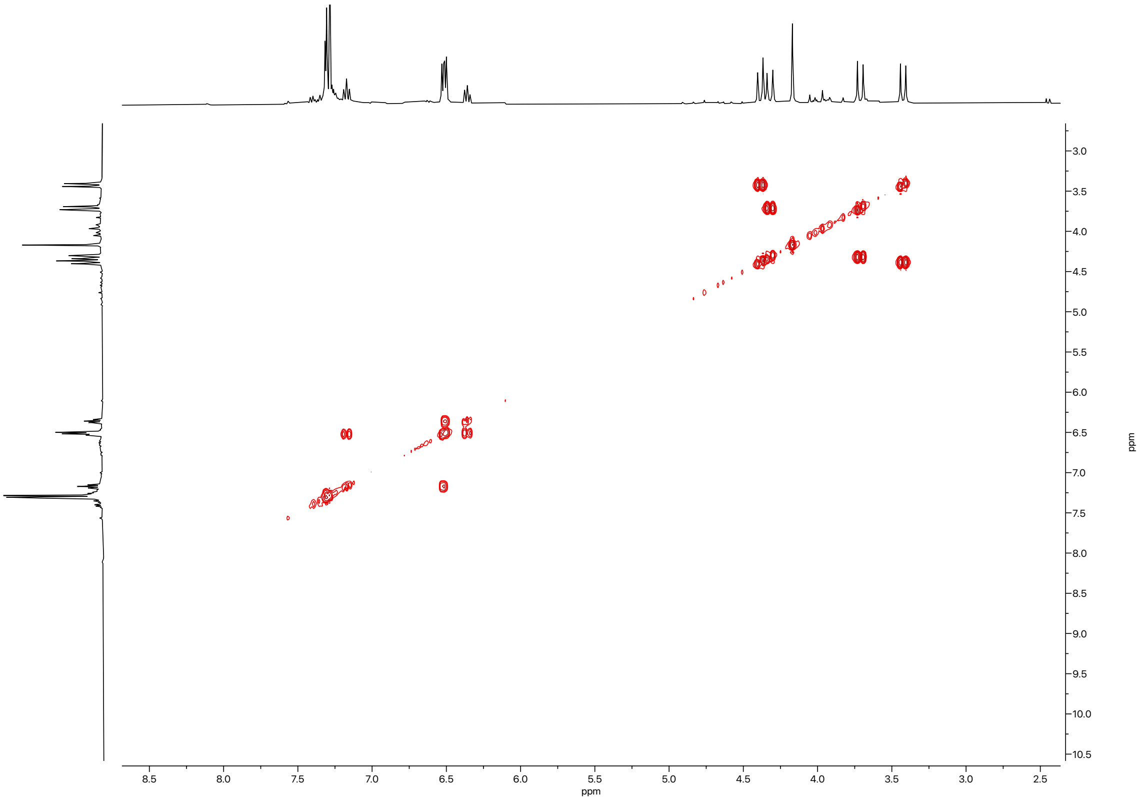

Figure S102. 400 MHz, ^{1}H-^{1}H COSY NMR spectrum of **P-a** in $CDCl_3$, 298 K.

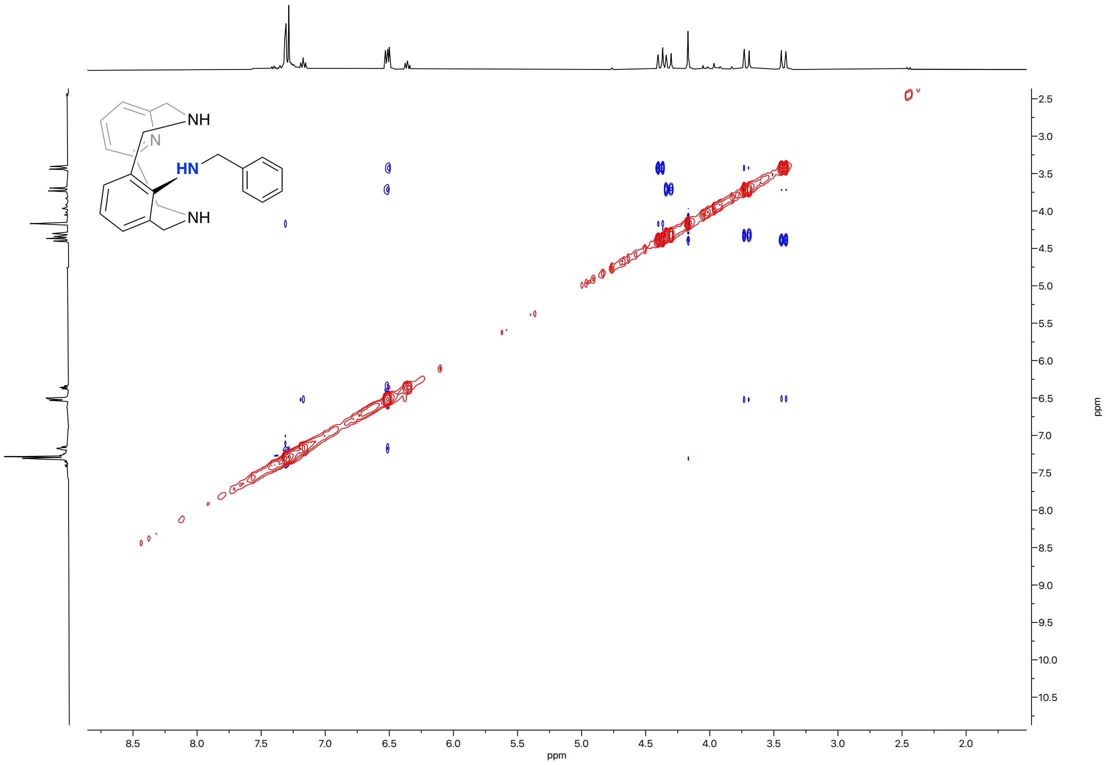

Figure S103. 400 MHz, ^{1}H-^{1}H NOESY NMR spectrum of **P-a** in $CDCl_3$, 298 K.

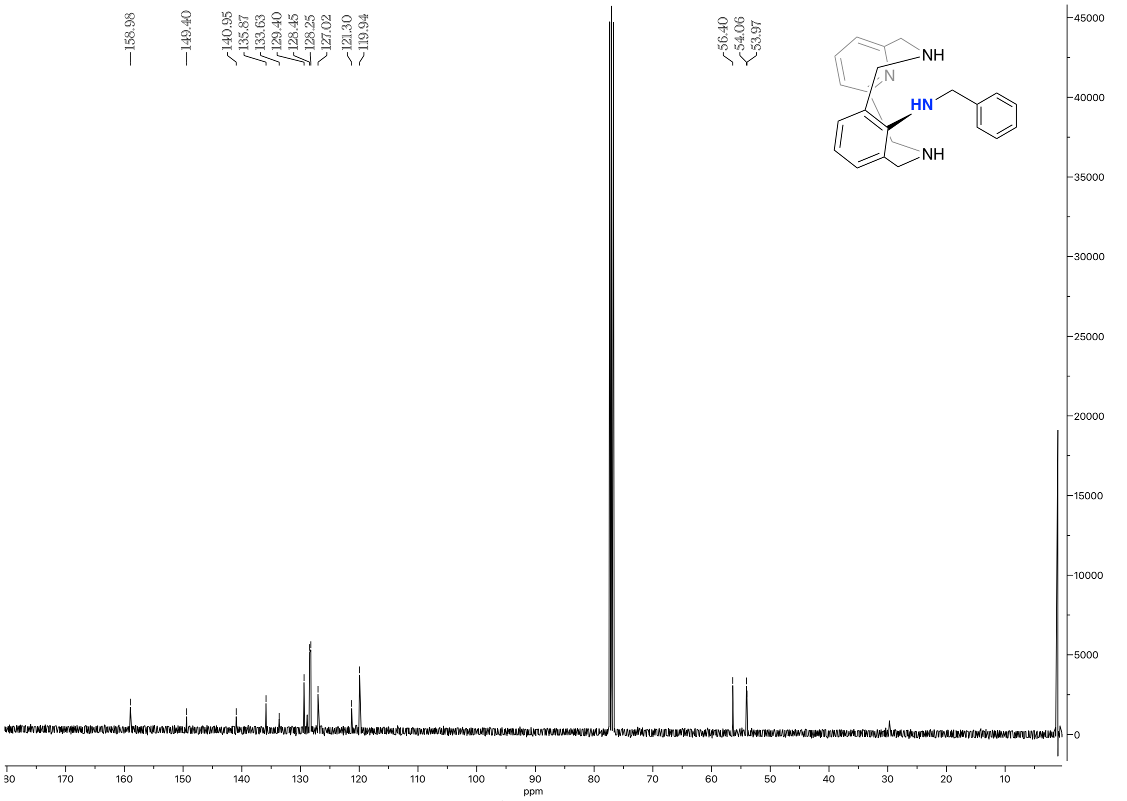

Figure S104. 100 MHz, $^{13}C\ \{^{1}H\}$ NMR spectrum of **P-a** in $CDCl_3$, 298 K.

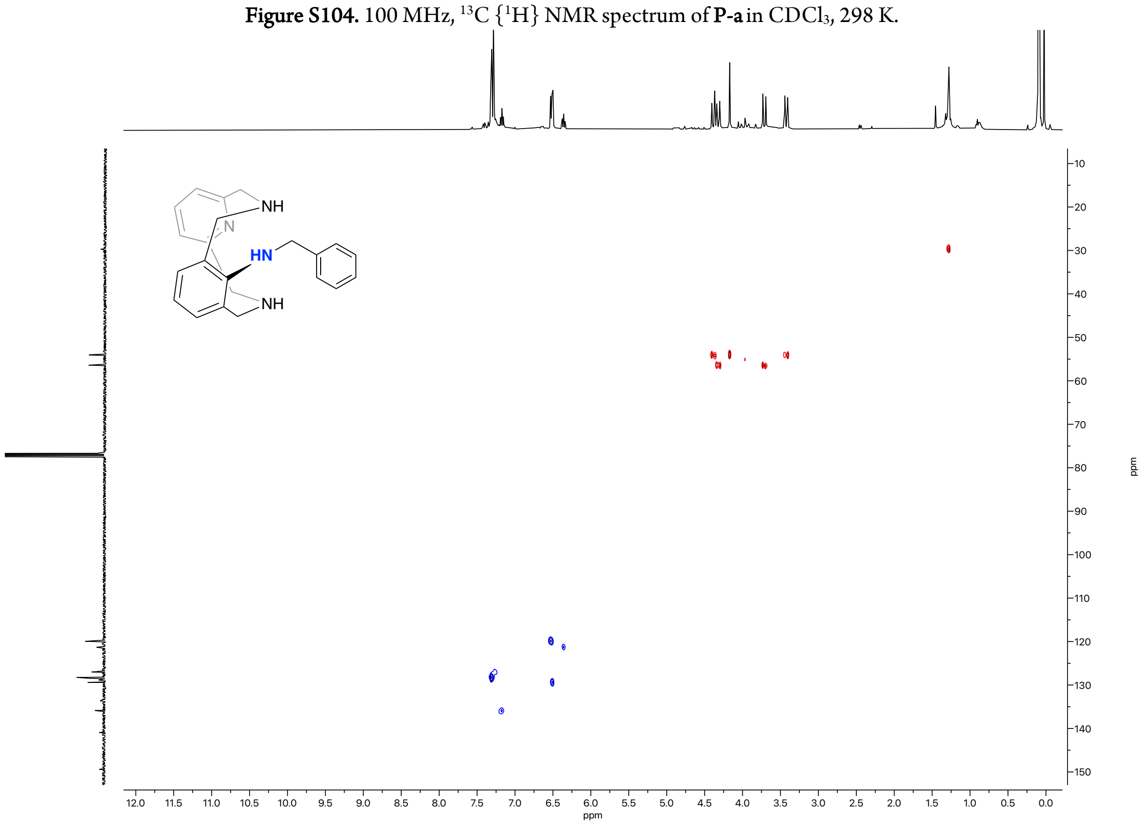

Figure S105. 400 MHz, ^{1}H-^{13}C HSQC NMR spectrum of **P-a** in $CDCl_3$, 298 K.

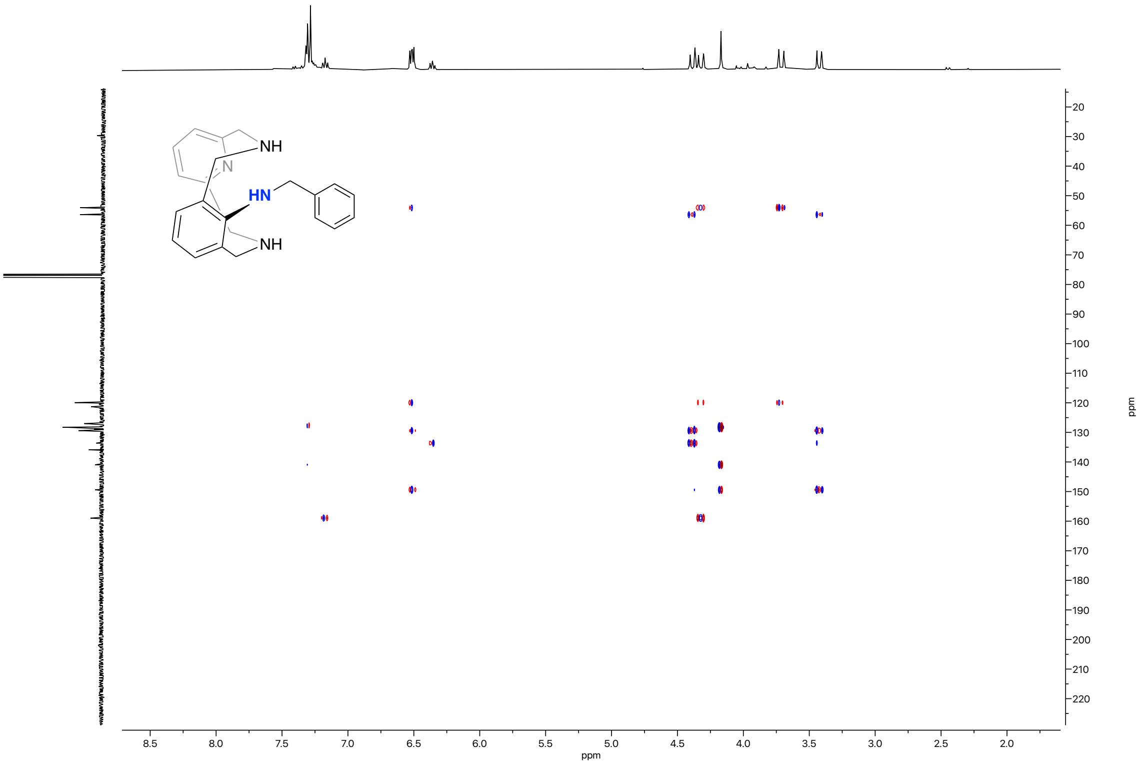

Figure S106. 400 MHz, ^{1}H-^{13}C HMBC NMR spectrum of **P-a** in $CDCl_3$, 298 K.

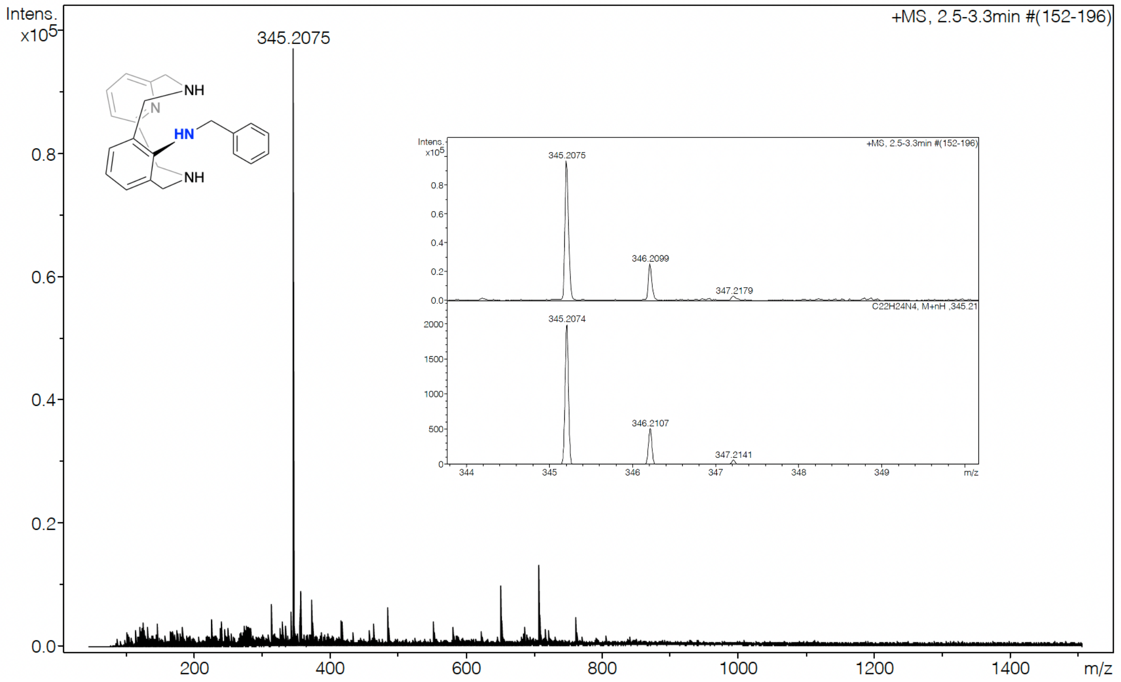

Figure S107. HRMS spectrum of **P-a** showing a peak at m/z = 345.2075. Inset: up, experimental spectrum; down, simulated spectrum.

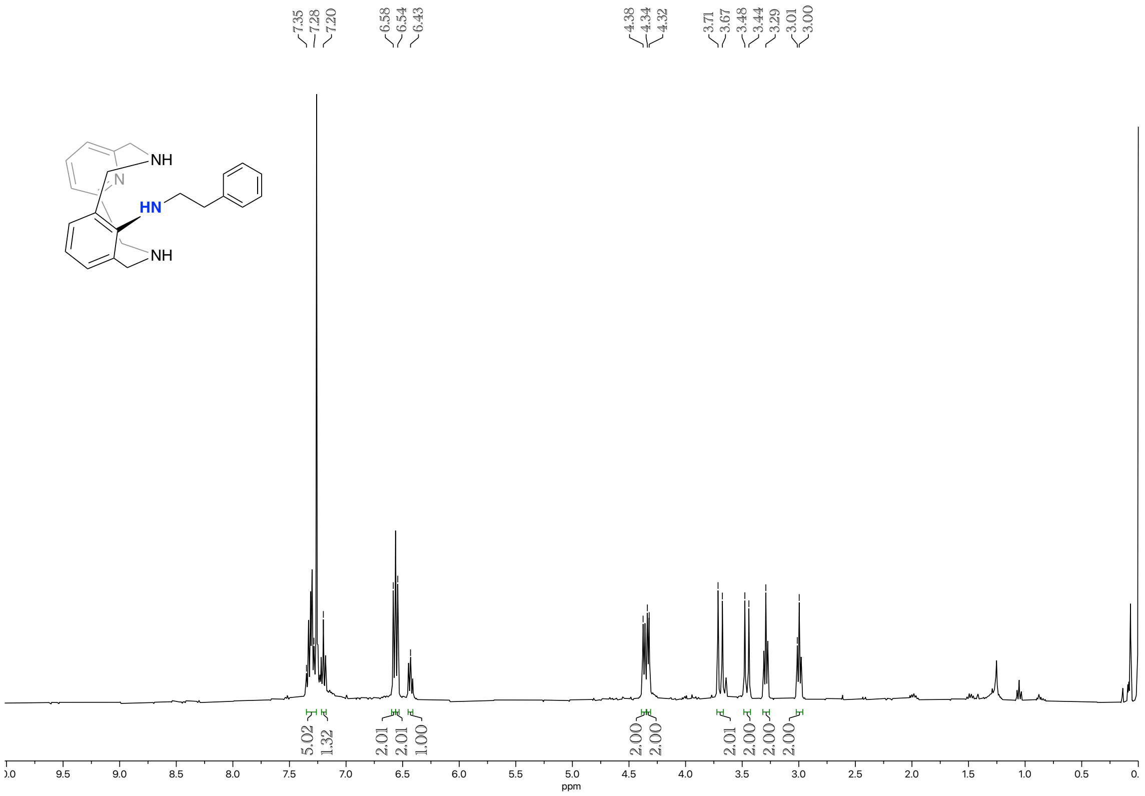

Figure S108. 400 MHz, ^{1}H NMR spectrum of **P-b** in $CDCl_3$, 298 K.

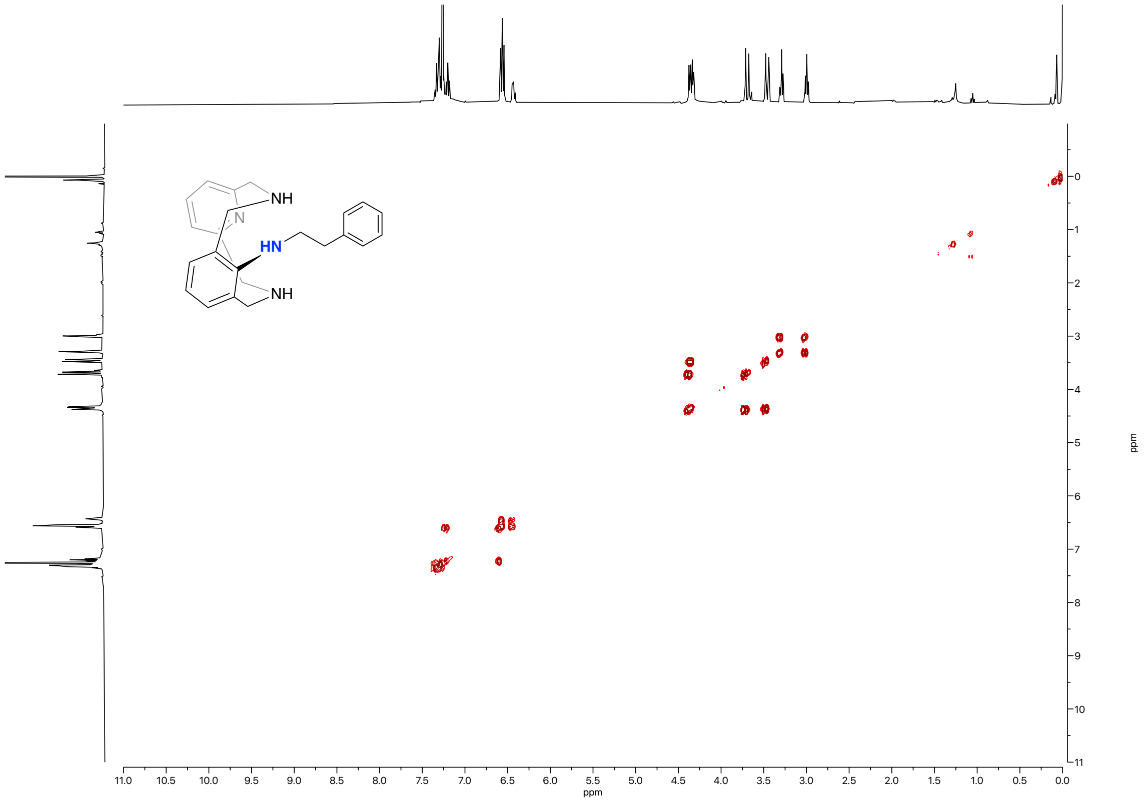

Figure S109. 400 MHz, ^{1}H-^{1}H COSY NMR spectrum of **P-b** in $CDCl_3$, 298 K.

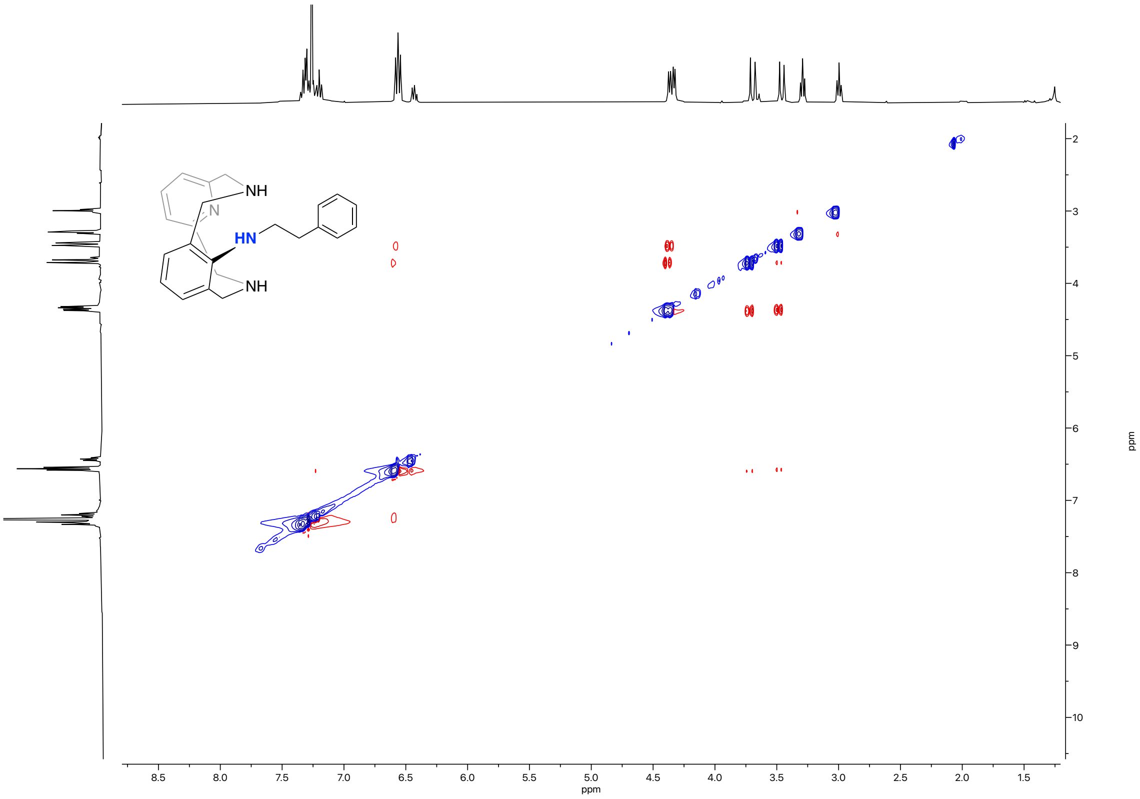

Figure S110. 400 MHz, ^{1}H-^{1}H NOESY NMR spectrum of **P-b** in $CDCl_3$, 298 K.

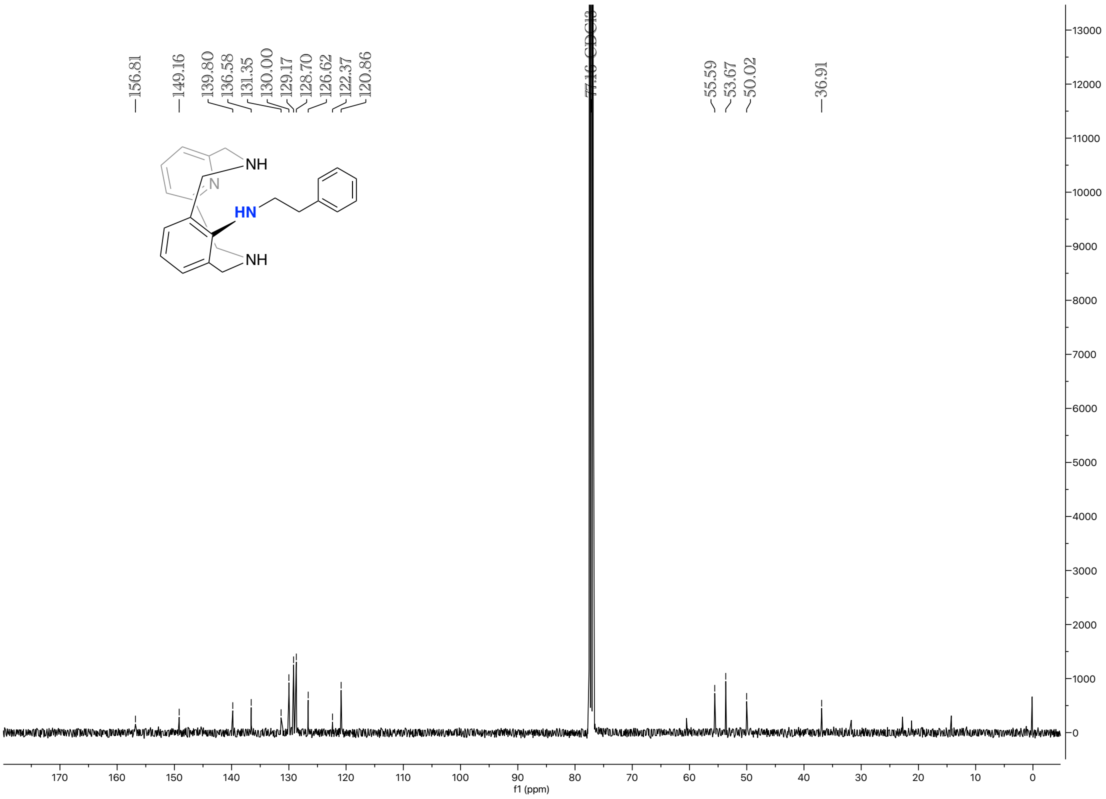

Figure S111. 100 MHz, ^{13}C {^{1}H} NMR spectrum of **P-b** in $CDCl_3$, 298 K.

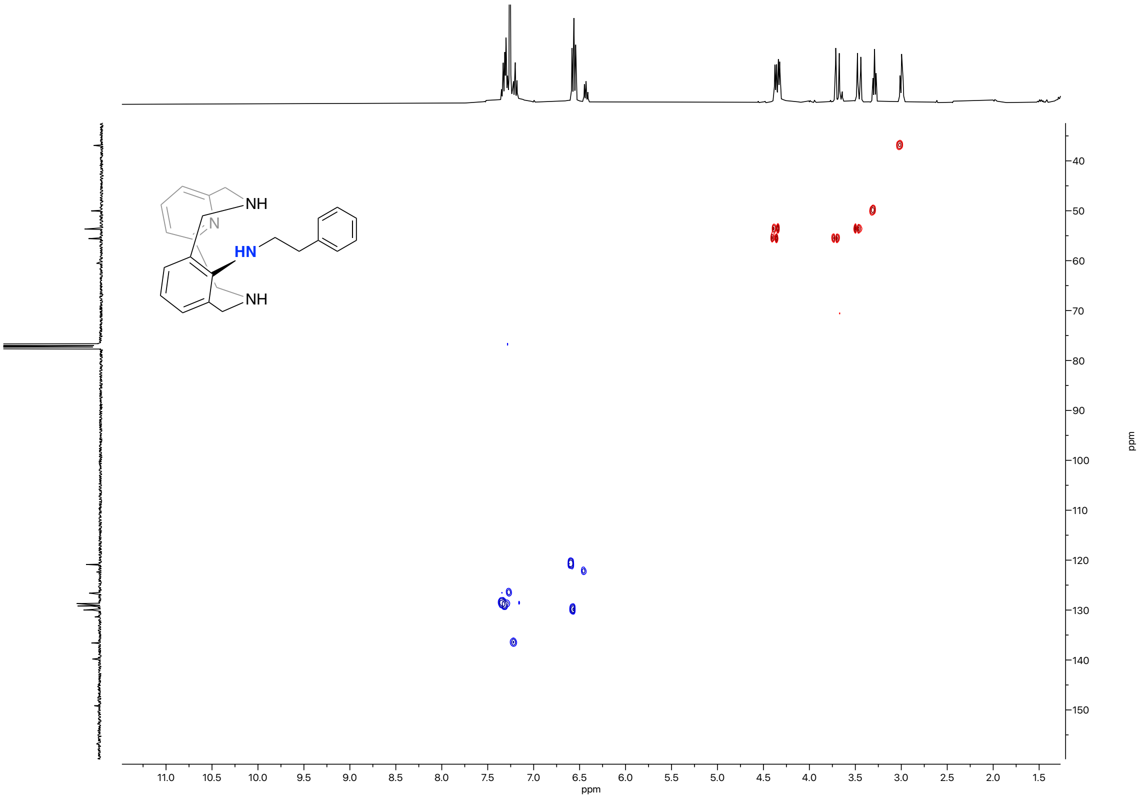

Figure S112. 400 MHz, ^{1}H-^{13}C HSQC NMR spectrum of **P-b** in $CDCl_3$, 298 K.

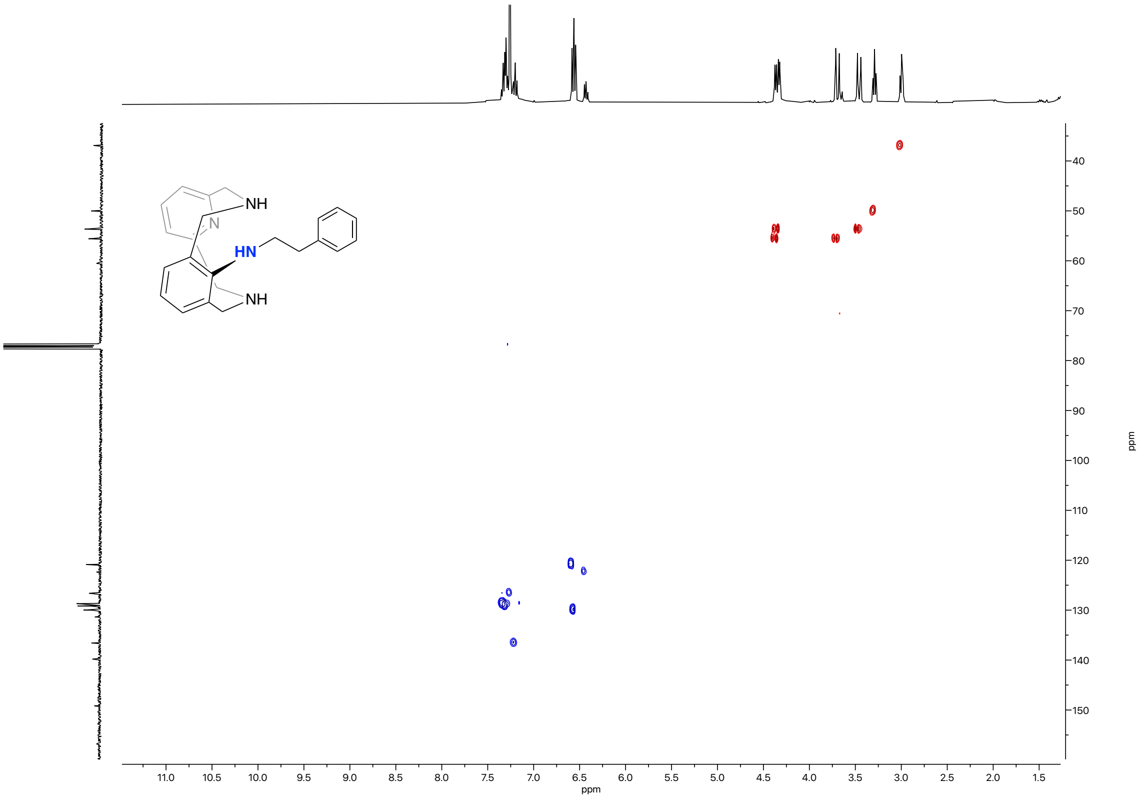

Figure S113. 400 MHz, ^{1}H-^{13}C HMBC NMR spectrum of **P-b** in $CDCl_3$, 298 K.

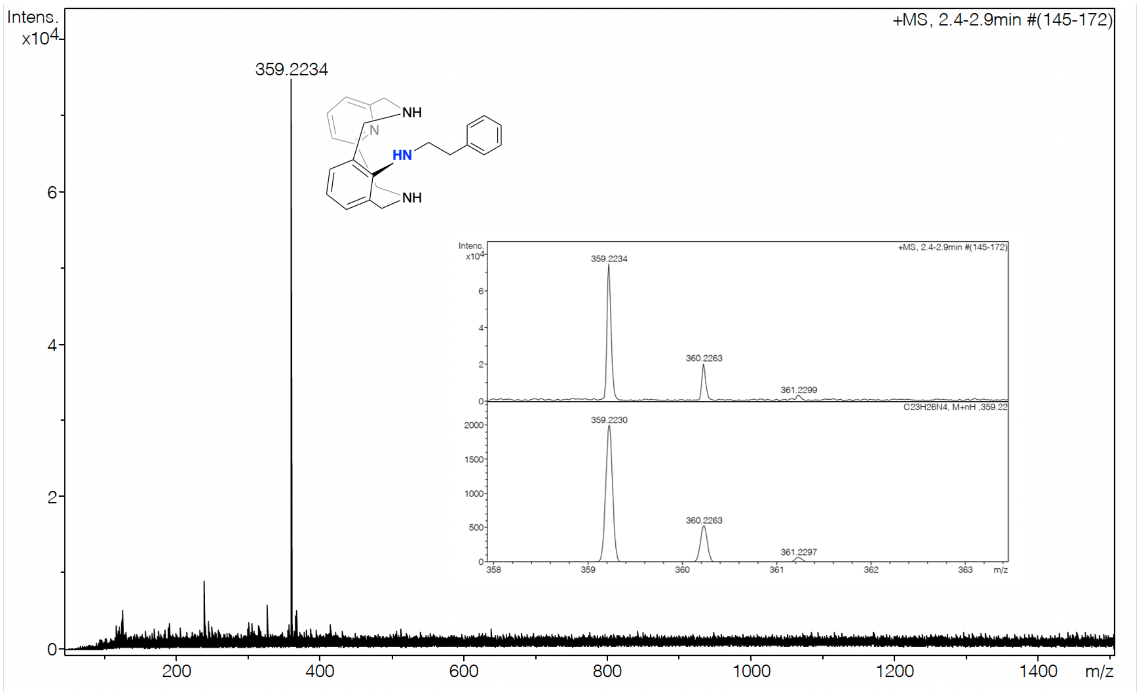

Figure S114. HRMS spectrum of **P-b** showing a peak at $m/z = 359.2234$. Inset: up, experimental spectrum; down, simulated spectrum.

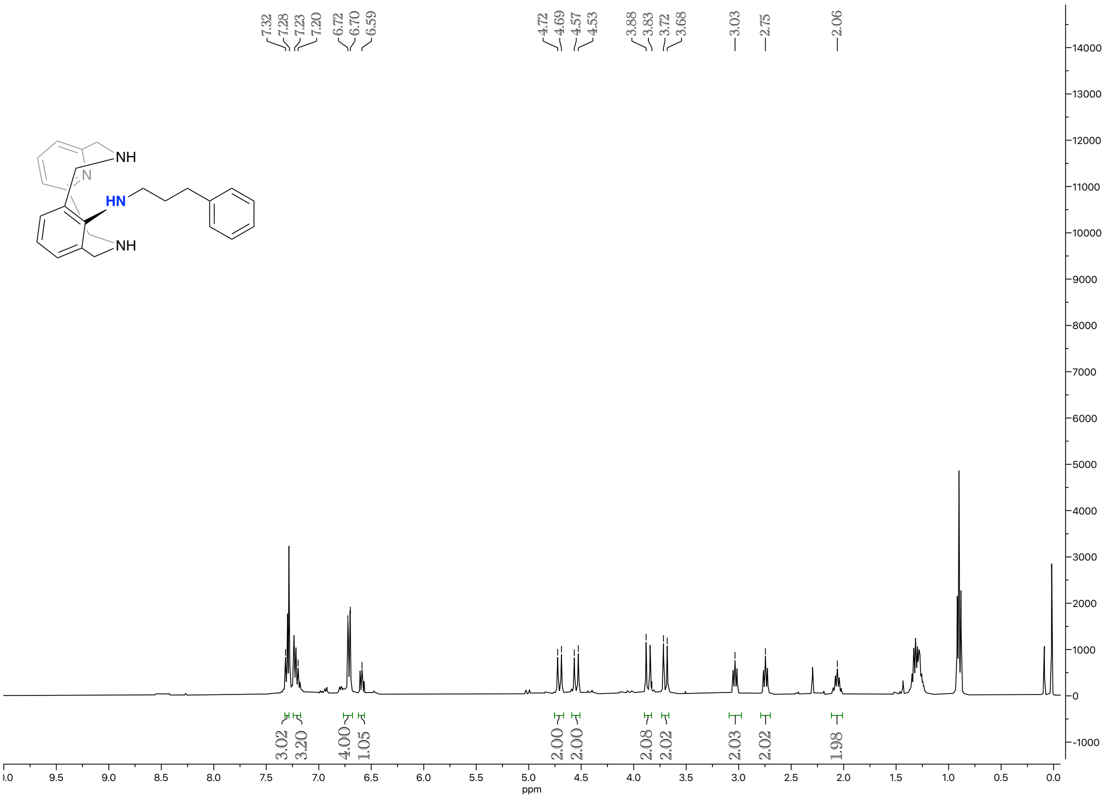

Figure S115. 400 MHz, ^{1}H NMR spectrum of **P-c** in $CDCl_3$, 298 K.

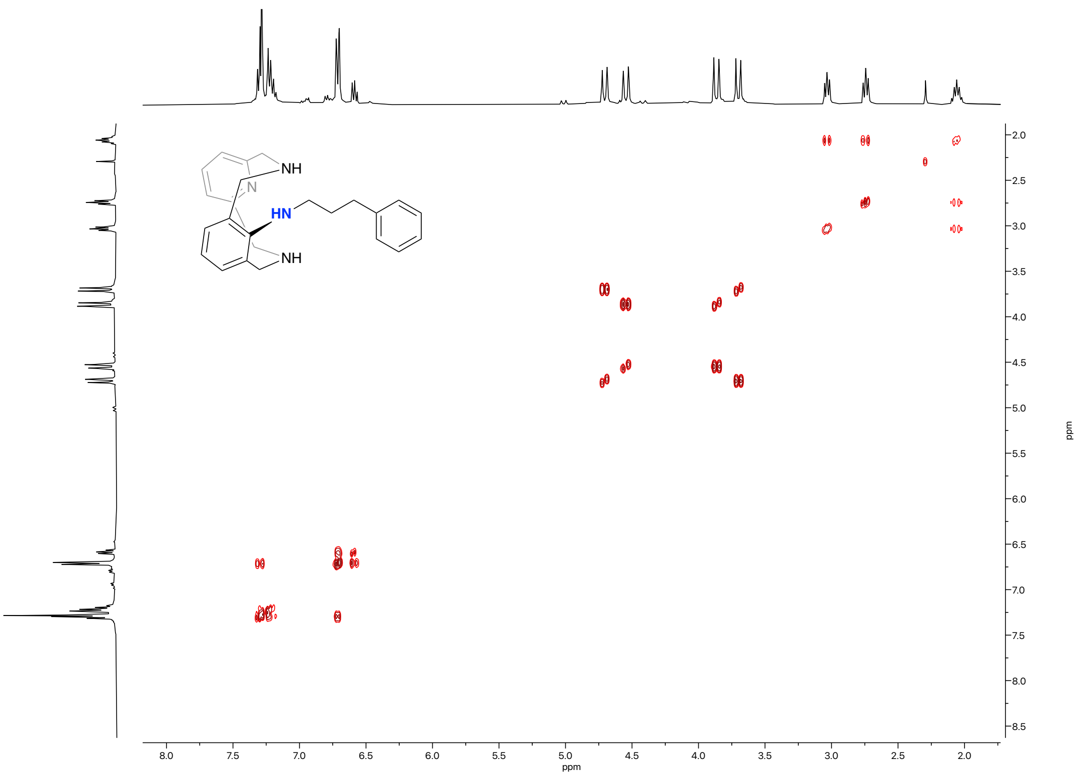

Figure S116. 400 MHz, ^{1}H-^{1}H COSY NMR spectrum of **P-c** in $CDCl_3$, 298 K.

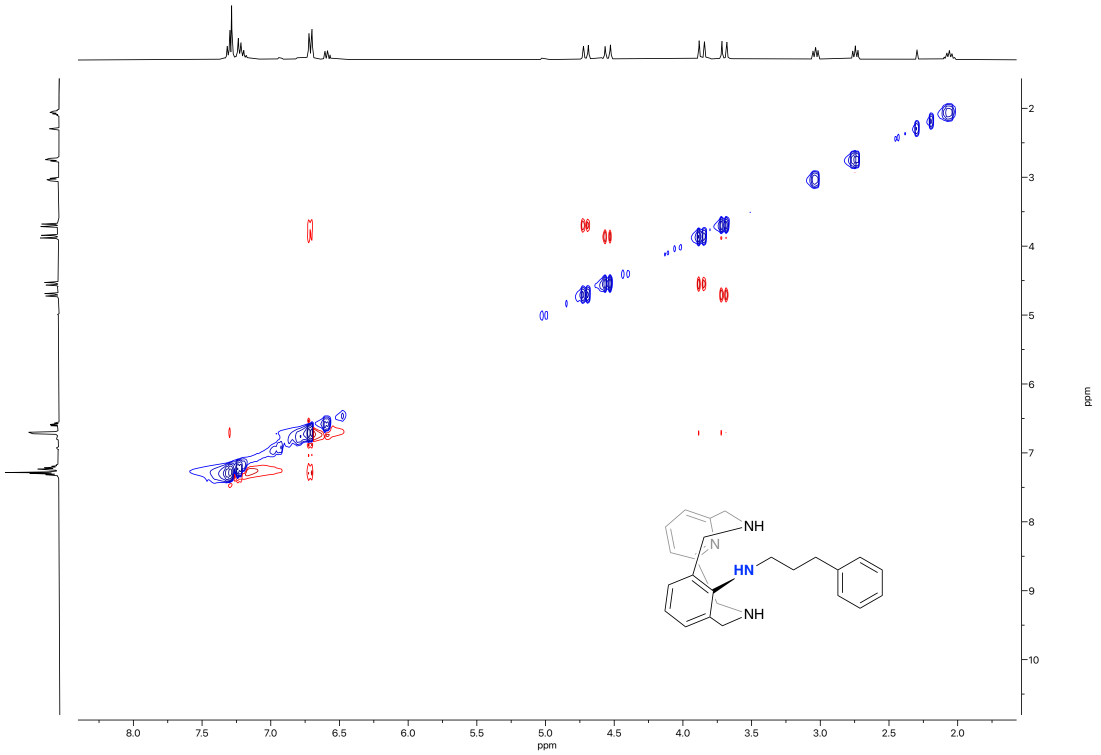

Figure S117. 400 MHz, ^{1}H-^{1}H NOESY NMR spectrum of **P-c** in $CDCl_3$, 298 K.

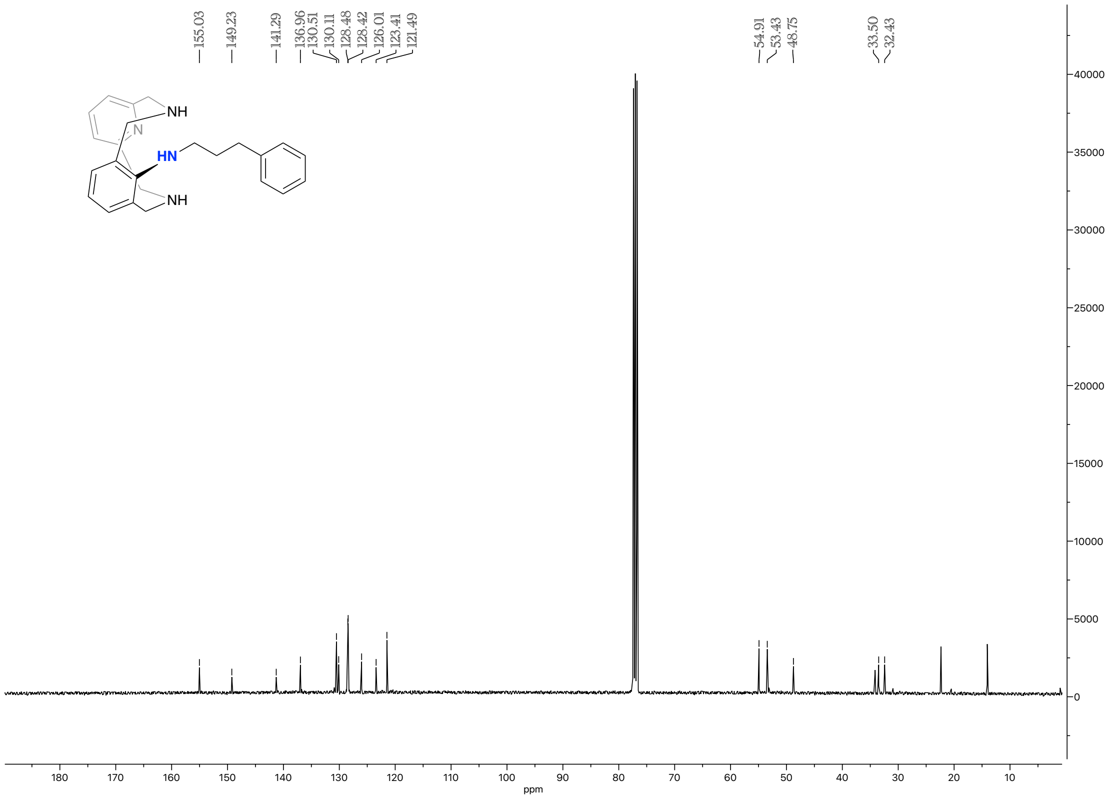

Figure S118. 400 MHz, $^{13}C\{^{1}H\}$ NMR spectrum of **P-c** in $CDCl_3$, 298 K.

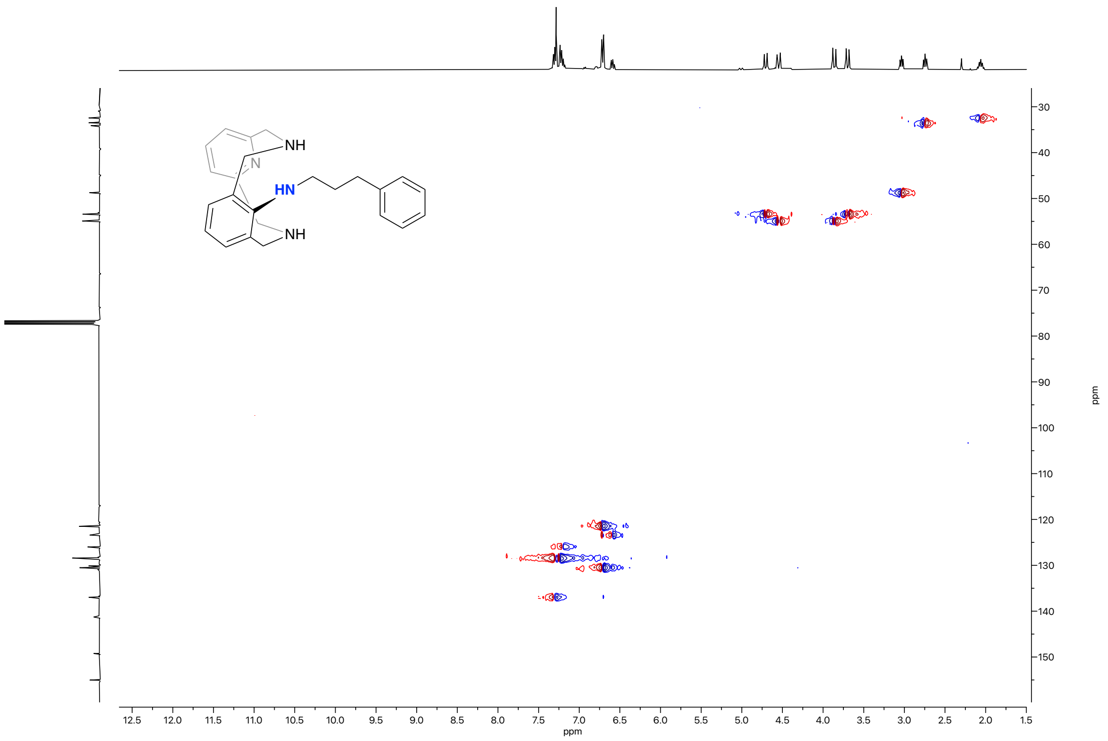

Figure S119. 400 MHz, ^{1}H-^{13}C HSQC NMR spectrum of **P-c** in $CDCl_3$, 298 K.

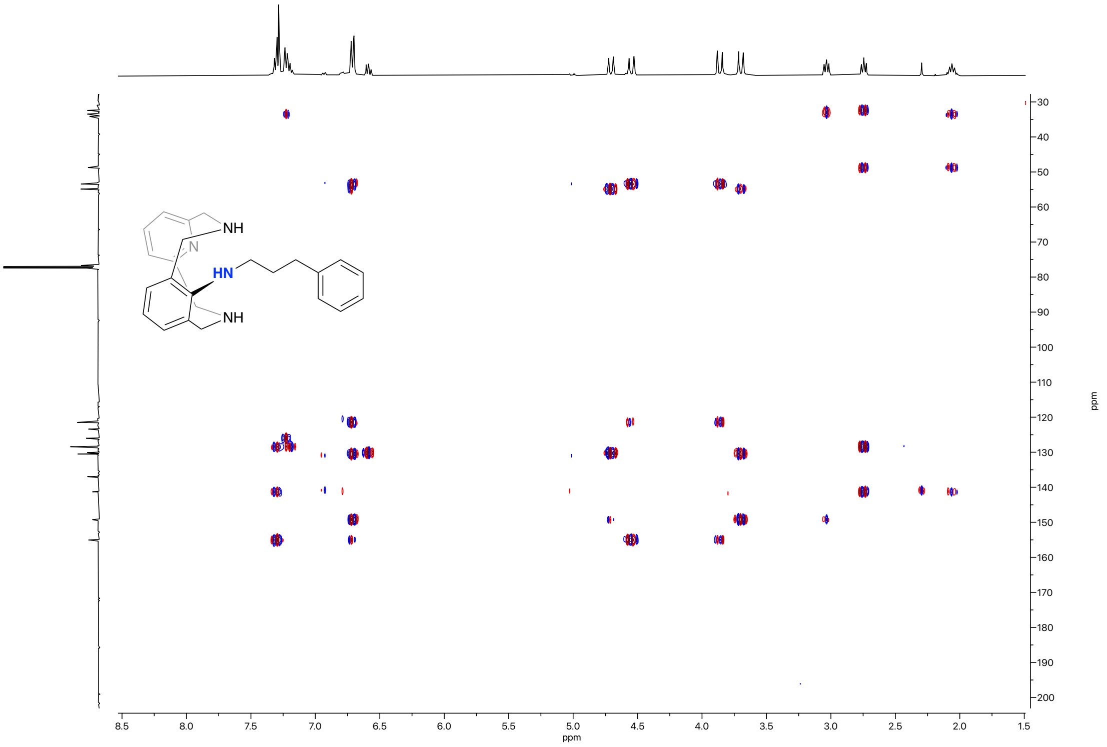

Figure S120. 400 MHz, ^{1}H-^{13}C HMBC NMR spectrum of **P-c** in $CDCl_3$, 298 K.

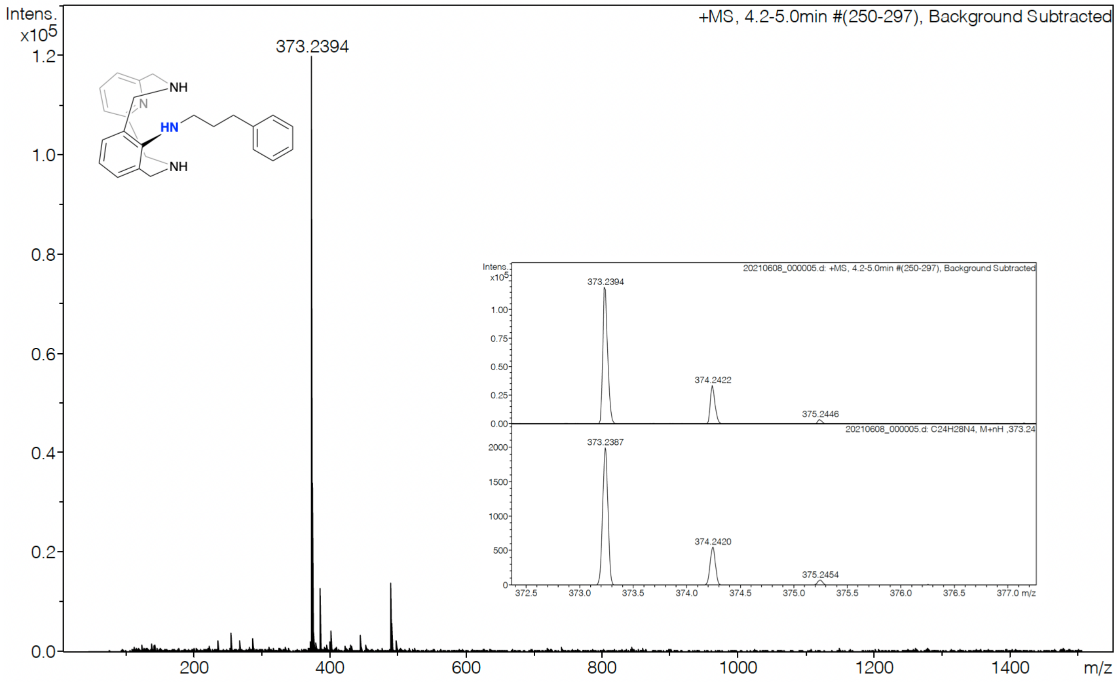

Figure S121. HRMS spectrum of **P-c** showing a peak at m/z = 373.2394. Inset: up, experimental; down, simulated.

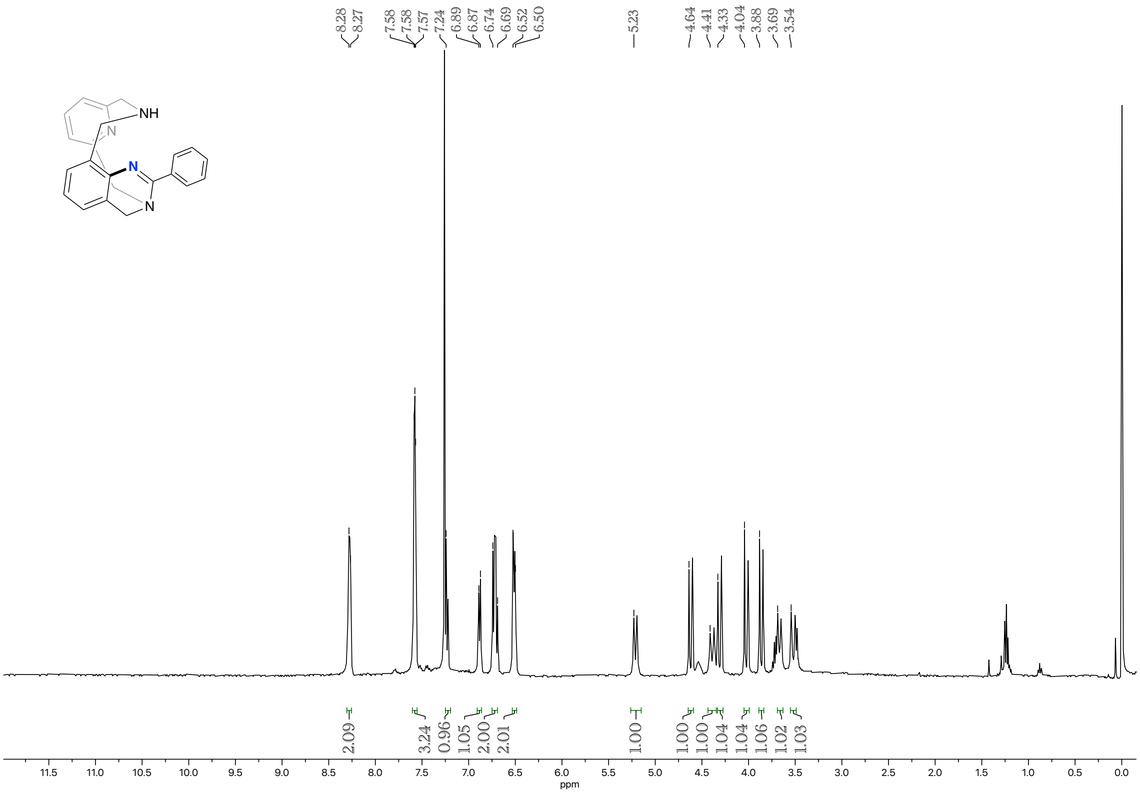

Figure S122. 400 MHz, ^{1}H NMR spectrum of **P-a-cyc** in $CDCl_3$, 298 K.

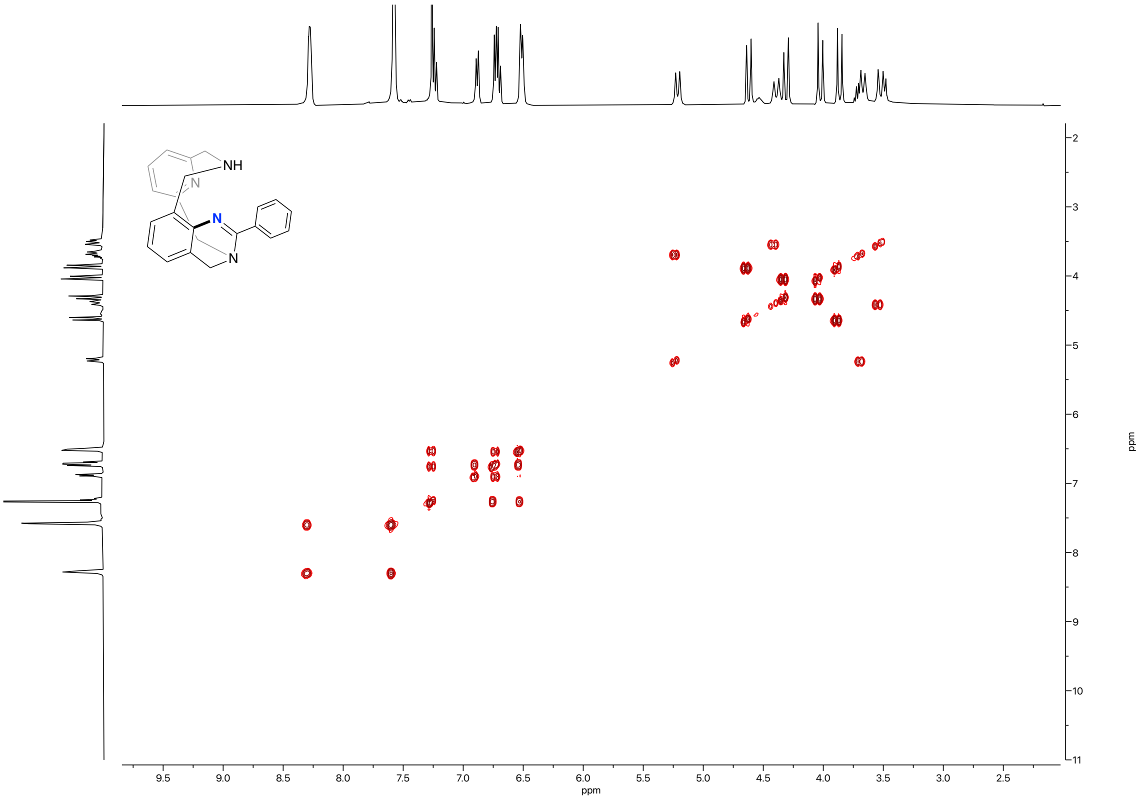

Figure S123. 400 MHz, ^{1}H-^{1}H COSY NMR spectrum of **P-a-cyc** in $CDCl_3$, 298 K.

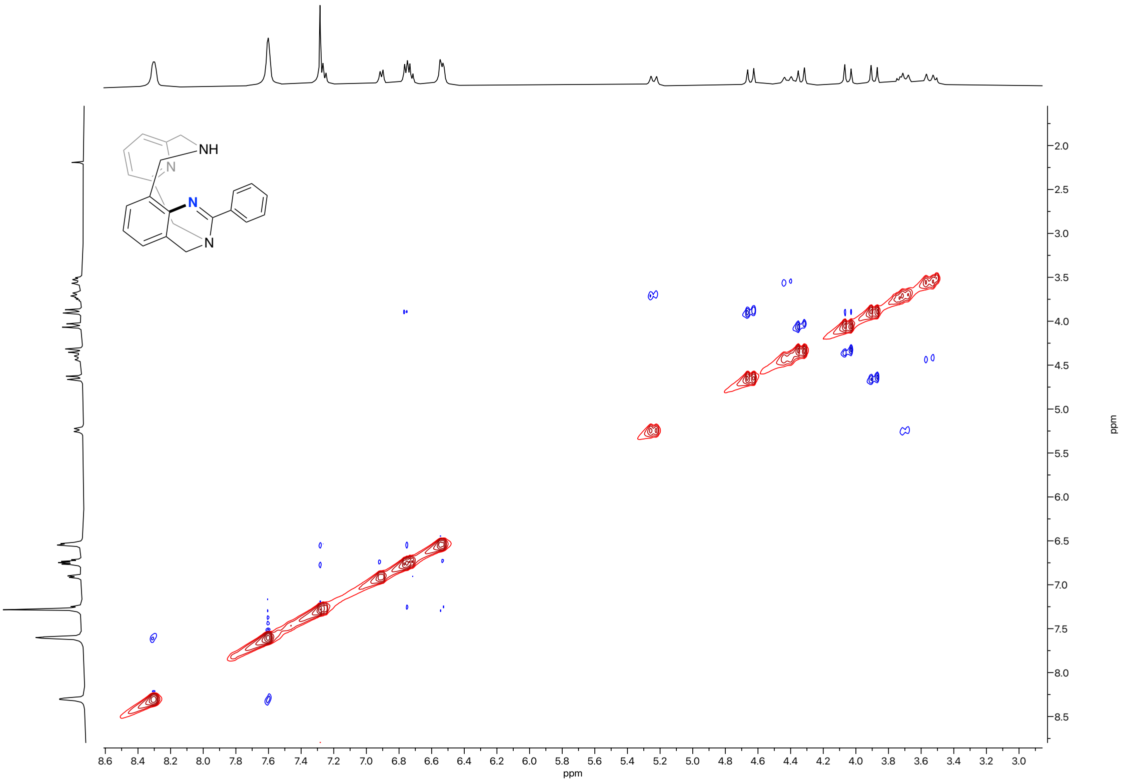

Figure S124. 400 MHz, ^{1}H-^{1}H NOESY NMR spectrum of **P-a-cyc** in $CDCl_3$, 298 K.

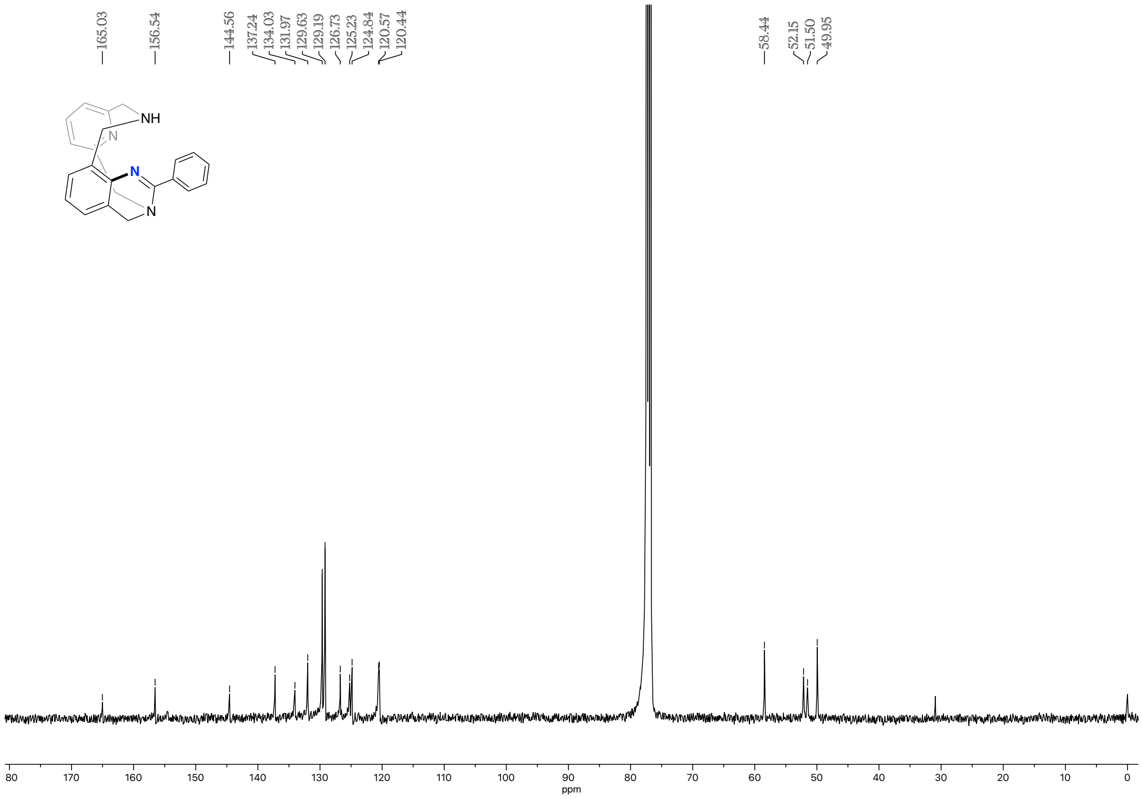

Figure S125. 100 MHz, ^{13}C {^{1}H} NMR spectrum of **P-a-cyc** in $CDCl_3$, 298 K.

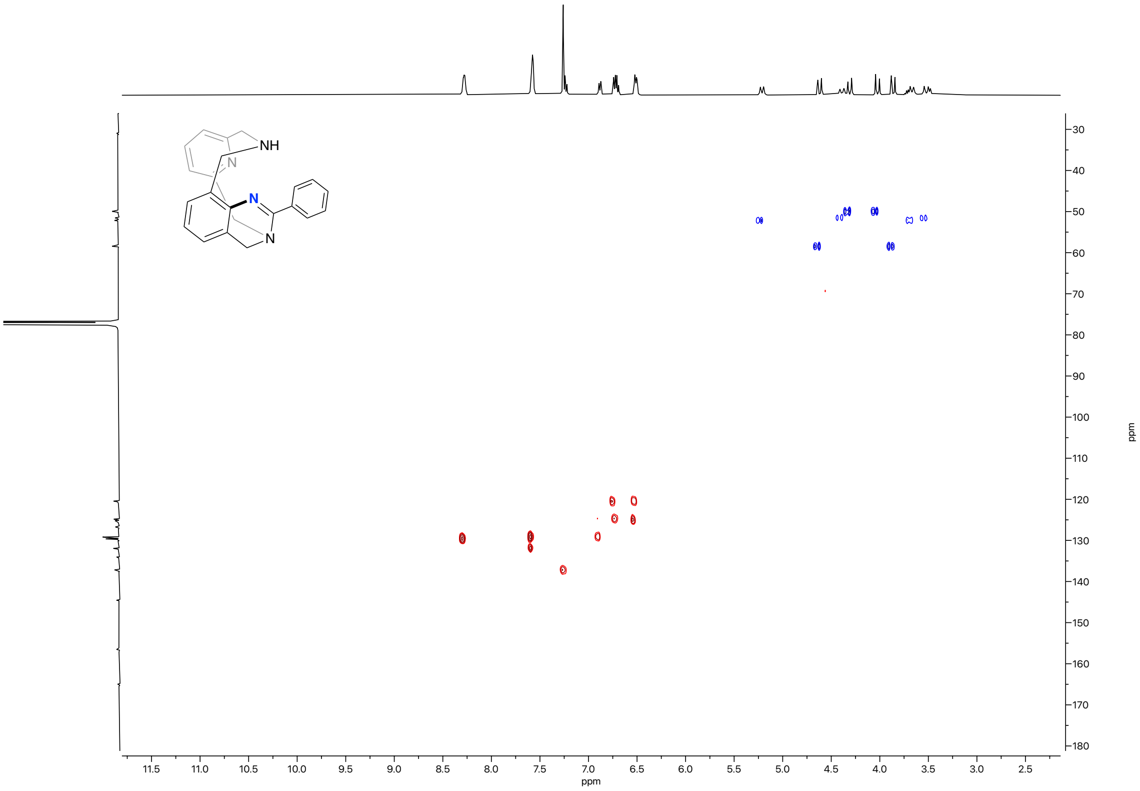

Figure S126. 400 MHz, ^{1}H-^{13}C HSQC NMR spectrum of **P-a-cyc** in $CDCl_3$, 298 K.

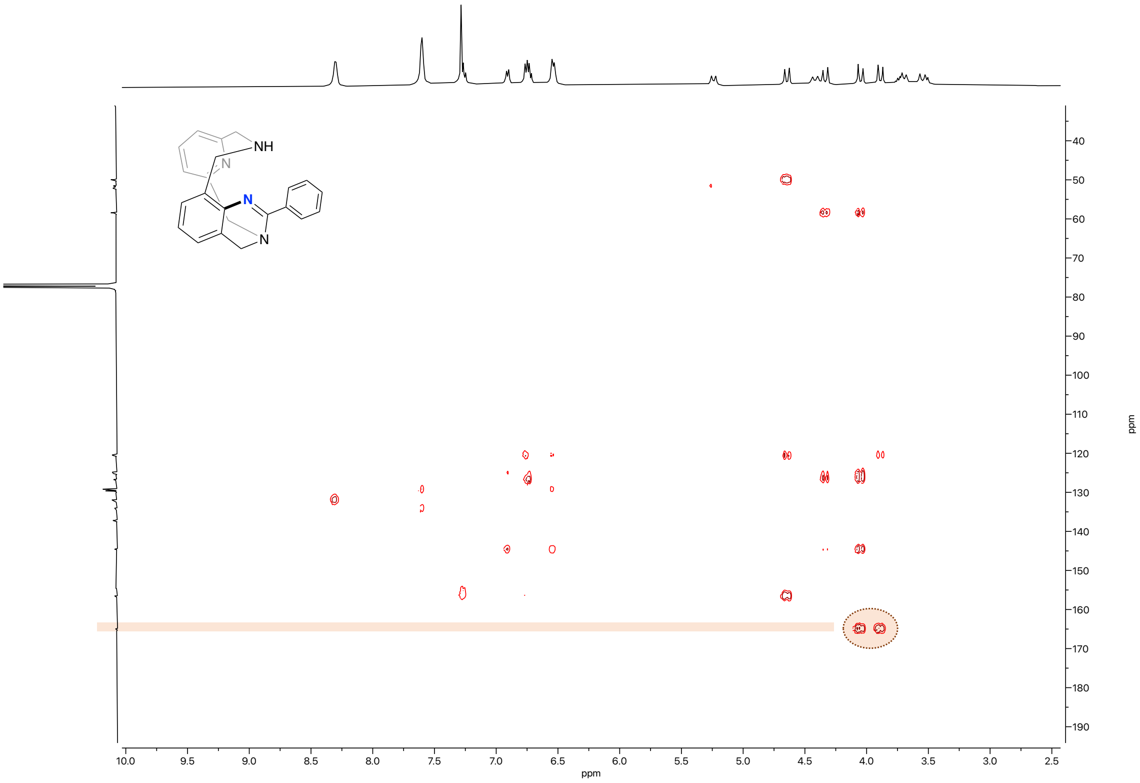

Figure S127. 400 MHz, ^{1}H-^{13}C HMBC NMR spectrum of **P-a-cyc** in $CDCl_3$, 298 K.

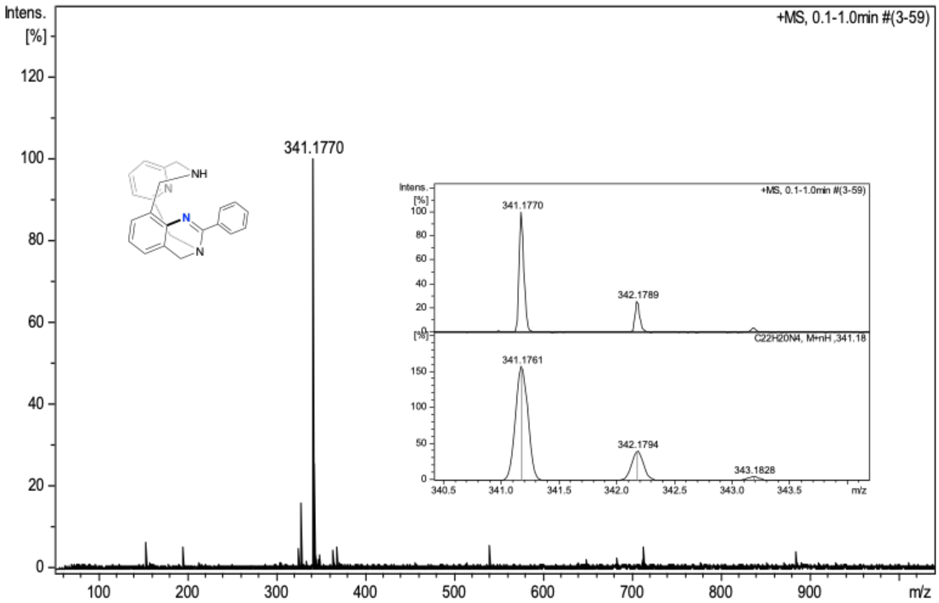

Figure S128. HRMS spectrum of **P-a-cyc** showing a peak at *m/z* = 341.1770. Inset: up, experimental spectrum; down, simulated spectrum.

13. References

1. Planas, O.; Whiteoak, C. J.; Martin-Diaconescu, V.; Gamba, I.; Luis, J. M.; Parella, T.; Company, A.; Ribas, X., Isolation of Key Organometallic Aryl-Co(III) Intermediates in Cobalt-Catalyzed C(sp2)–H Functionalizations and New Insights into Alkyne Annulation Reaction Mechanisms. *J. Am. Chem. Soc.* **2016,** *138*, 14388-14397.
2. Planas, O.; Roldán-Gómez, S.; Martin-Diaconescu, V.; Parella, T.; Luis, J. M.; Company, A.; Ribas, X., Carboxylate-Assisted Formation of Aryl-Co(III) Masked-Carbenes in Cobalt-Catalyzed C–H Functionalization with Diazo Esters. *J. Am. Chem. Soc.* **2017,** *139*, 14649-14655.
3. Planas, O.; Roldán-Gómez, S.; Martin-Diaconescu, V.; Luis, J. M.; Company, A.; Ribas, X., Mechanistic insights into the SN2-type reactivity of aryl-Co(iii) masked-carbenes for C–C bond forming transformations. *Chem. Sci.* **2018,** *9*, 5736-5746.
4. Ravel, B.; Newville, M., ATHENA, ARTEMIS, HEPHAESTUS: data analysis for X-ray absorption spectroscopy using IFEFFIT. *J. Synchrotron Rad.* **2005,** *12*, 537-541.
5. Rehr, J. J.; Albers, R. C., Theoretical approaches to x-ray absorption fine structure. *Rev. Modern Phys.* **2000,** *72*, 621-654.
6. Newville, M., EXAFS analysis using FEFF and FEFFIT. *J. Synchrotron Rad.* **2001,** *8*, 96-100.
7. Frisch, M. J. T., G. W.; Schlegel, H. B.; Scuseria, G. E.; Robb, M. A.; Cheeseman, J. R.; Scalmani, G.; Barone, V.; Petersson, G. A.; Nakatsuji, H.; Li, X.; Caricato, M.; Marenich, A. V.; Bloino, J.; Janesko, B. G.; Gomperts, R.; Mennucci, B.; Hratchian, H. P.; Ortiz, J. V.; Izmaylov, A. F.; Sonnenberg, J. L.; Williams-Young, D.; Ding, F.; Lipparini, F.; Egidi, F.; Goings, J.; Peng, B.; Petrone, A.; Henderson, T.; Ranasinghe, D.; Zakrzewski, V. G.; Gao, J.; Rega, N.; Zheng, G.; Liang, W.; Hada, M.; Ehara, M.; Toyota, K.; Fukuda, R.; Hasegawa, J.; Ishida, M.; Nakajima, T.; Honda, Y.; Kitao, O.; Nakai, H.; Vreven, T.; Throssell, K.; Montgomery, J. A., Jr.; Peralta, J. E.; Ogliaro, F.; Bearpark, M. J.; Heyd, J. J.; Brothers, E. N.; Kudin, K. N.; Staroverov, V. N.; Keith, T. A.; Kobayashi, R.; Normand, J.; Raghavachari, K.; Rendell, A. P.; Burant, J. C.; Iyengar, S. S.; Tomasi, J.; Cossi, M.; Millam, J. M.; Klene, M.; Adamo, C.; Cammi, R.; Ochterski, J. W.; Martin, R. L.; Morokuma, K.; Farkas, O.; Foresman, J. B.; Fox, D. J. *Gaussian 16, Revision A.03*, Gaussian Inc.: Wallingford CT, 2016.
8. Marenich, A. V.; Cramer, C. J.; Truhlar, D. G., Universal solvation model based on solute electron density and on a continuum model of the solvent defined by the bulk dielectric constant and atomic surface tensions. *J. Phys. Chem. B* **2009,** *113*, 6378-6396.
9. Grimme, S.; Ehrlich, S.; Goerigk, L., Effect of the damping function in dispersion corrected density functional theory. *J. Comput. Chem.* **2011,** *32*, 1456-1465.
10. Grimme, S.; Antony, J.; Ehrlich, S.; Krieg, H., A consistent and accurate ab initio parametrization of density functional dispersion correction (DFT-D) for the 94 elements H-Pu. *J. Chem. Phys.* **2010,** *132*, 154104.
11. Becke, A. *D.*, *Phys. Rev. A* **1988,** *38*, 3098-3100.
12. Perdew, J. P., *Phys. Rev. B* **1986,** *33*, 8822-8824.
13. Weigend, F.; Ahlrichs, R., Balanced basis sets of split valence, triple zeta valence and quadruple zeta valence quality for H to Rn: Design and assessment of accuracy. *Phys. Chem. Chem. Phys.* **2005,** *7*, 3297-3305.
14. Schäfer, A.; Huber, C.; Ahlrichs, R., Fully optimized contracted Gaussian basis sets of triple zeta valence quality for atoms Li to Kr. *J. Chem. Phys.* **1994,** *100*, 5829-5835.
15. https://comp.chem.umn.edu/solvation/mnsddb.pdf.
16. Hawkins, G. D.; Cramer, C. J.; Truhlar, D. G., Universal Quantum Mechanical Model for Solvation Free Energies Based on Gas-Phase Geometries. *J. Phys. Chem. B* **1998,** *102*, 3257-3271.
17. https://www.chemicalbook.com/ChemicalProductProperty_EN_CB3251829.htm.
18. Abraham, M. H., Hydrogen bonding. 31. Construction of a scale of solute effective or summation hydrogen-bond basicity. *J. Phys. Org. Chem.* **1993,** *6*, 660-684.
19. Berkessel, A.; Adrio, J. A., Dramatic Acceleration of Olefin Epoxidation in Fluorinated Alcohols: Activation of Hydrogen Peroxide by Multiple H-Bond Networks. *J. Am. Chem. Soc.* **2006,** *128*, 13412-13420.
20. Mayans, E.; Ballano, G.; Sendros, J.; Font-Bardia, M.; Campos, J. L.; Puiggalí, J.; Cativiela, C.; Alemán, C., Effect of Solvent Choice on the Self-Assembly Properties of a Diphenylalanine Amphiphile Stabilized by an Ion Pair. *ChemPhysChem* **2017,** *18*, 1888-1896.
21. Goerigk, L.; Hansen, A.; Bauer, C.; Ehrlich, S.; Najibi, A.; Grimme, S., A look at the density functional theory zoo with the advanced GMTKN55 database for general main group thermochemistry, kinetics and noncovalent interactions. *Phys. Chem. Chem. Phys.* **2017,** *19*, 32184-32215.
22. Perdew, J. P.; Ruzsinszky, A.; Csonka, G. I.; Constantin, L. A.; Sun, J., Workhorse Semilocal Density Functional for Condensed Matter Physics and Quantum Chemistry. *Phys. Rev. Lett.* **2009,** *103*, 026403.
23. Luchini, G.; Alegre-Requena, J.; Funes-Ardoiz, I.; Paton, R., GoodVibes: automated thermochemistry for heterogeneous computational chemistry data [version 1; peer review: 2 approved with reservations]. *F1000Research* **2020,** *9*.
24. Grimme, S., Supramolecular Binding Thermodynamics by Dispersion-Corrected Density Functional Theory. *Chem. Eur. J.* **2012,** *18*, 9955-9964.
25. Kelly, C. P.; Cramer, C. J.; Truhlar, D. G., Single-Ion Solvation Free Energies and the Normal Hydrogen Electrode Potential in Methanol, Acetonitrile, and Dimethyl Sulfoxide. *J. Phys. Chem. B* **2007,** *111*, 408-422.
26. Ramos-Cordoba, E.; Postils, V.; Salvador, P., Oxidation States from Wave Function Analysis. *J. Chem. Theor. Comput.* **2015,** *11*, 1501-1508.
27. Lee, J. H.; Gupta, S.; Jeong, W.; Rhee, Y. H.; Park, J., Characterization and Utility of N-Unsubstituted Imines Synthesized from Alkyl Azides by Ruthenium Catalysis. *Angew. Chem. Int. Ed.* **2012,** *51*, 10851-10855.

28. Albertin, G.; Antoniutti, S.; Baldan, D.; Castro, J.; García-Fontán, S., Preparation of Benzyl Azide Complexes of Iridium(III). *Inorg. Chem.* **2008,** *47*, 742-748.
29. Kim, H.; Chang, S., Intramolecular Amido Transfer Leading to Structurally Diverse Nitrogen-Containing Macrocycles. *Angew. Chem. Int. Ed.* **2017,** *56*, 3344-3348.
30. Spek, A., PLATON SQUEEZE: a tool for the calculation of the disordered solvent contribution to the calculated structure factors. *Acta Cryst. C* **2015,** *71*, 9-18.
31. Kuijpers, P. F.; van der Vlugt, J. I.; Schneider, S.; de Bruin, B., Nitrene Radical Intermediates in Catalytic Synthesis. *Chem. Eur. J.* **2017,** *23*, 13819-13829.
